# Dual binding in cohesin-dockerin complexes: the energy landscape and the role of short, terminal segments of the dockerin module

**DOI:** 10.1038/s41598-018-23380-9

**Published:** 2018-03-22

**Authors:** Michał Wojciechowski, Bartosz Różycki, Pham Dinh Quoc Huy, Mai Suan Li, Edward A. Bayer, Marek Cieplak

**Affiliations:** 1 Institute of Physics, Polish Academy of Sciences, Al. Lotników 32/46, PL-02668 Warsaw, Poland; 2 Institute for Computational Sciences and Technology, SBI building, Quang Trung Software city, Tan Chanh Hiep Ward, District 12, Ho Chi Minh City, Vietnam; 3 Department of Biomolecular Sciences, The Weizmann Institute of Science, 234 Herzl Street, Rehovot, 7610001 Israel

## Abstract

The assembly of the polysaccharide degradating cellulosome machinery is mediated by tight binding between cohesin and dockerin domains. We have used an empirical model known as FoldX as well as molecular mechanics methods to determine the free energy of binding between a cohesin and a dockerin from *Clostridium thermocellum* in two possible modes that differ by an approximately 180° rotation. Our studies suggest that the full-length wild-type complex exhibits dual binding at room temperature, i.e., the two modes of binding have comparable probabilities at equilibrium. The ability to bind in the two modes persists at elevated temperatures. However, single-point mutations or truncations of terminal segments in the dockerin result in shifting the equilibrium towards one of the binding modes. Our molecular dynamics simulations of mechanical stretching of the full-length wild-type cohesin-dockerin complex indicate that each mode of binding leads to two kinds of stretching pathways, which may be mistakenly taken as evidence of dual binding.

## Introduction

The fibrous plant cell walls are the major source of carbon and energy on Earth. They are made primarily of cellulose and hemicellulose, which are difficult to degrade into simple sugars. The simple sugars can be transformed to ethanol, to produce biofuels, through fermentation. In nature, the degradation process takes place as a result of the catalytic action of microbially produced enzymes. In particular, many anaerobic organisms have developed special extracellular organelles, known as cellulosomes^1–9^, which are very efficient at performing degradation. The efficiency is accomplished through binding of many enzymes to a large noncatalytic protein known as scaffoldin so that the enzymes can act in one location together instead of being dispersed.

Scaffoldin consists of a number of covalently linked cohesins (Coh) and carbohydrate binding modules (CBM) that anchor to polysaccharide chains. The cohesins bind tightly and specifically to dockerin-bearing cellulosomal subunits, which comprise a dockerin domain (Doc), one or more enzymatic domains, and sometimes CBMs. These domains are typically connected by disordered polypeptide segments, which are often termed linkers. The amino-acid sequences of the linkers differ from one dockerin to another. In general, the longer the linker, the less restrictive the tethering constraints, which thus facilitates the interaction of the catalytic domain with the substrate^10–12^.

A number of atomic structures of cellulosome-constituing domains and subunits have been solved by X-ray crystallography and NMR. However, there is no single method that could be used to solve structures of the cellulosomes: they are not directly accessible to X-ray crystallography due to the presence of the disordered linkers (although their constituent domains can be crystallized separately); they are not accessible to protein NMR because of their large sizes; and their inherent flexibility makes them still practically inaccessible to cryoEM. Therefore, to delineate conformations of such protein assemblies as the cellulosomes, various complementary methods need to be combined^13,14^. In particular, small angle X-ray scattering (SAXS) in solution is increasingly used to complement protein crystallography in structural studies on multi-protein complexes, including the cellulosomal complexes^15–22^. In addition, these structural methods combined with molecular dynamics simulations or other modeling methods can lead to insights into dynamic properties and conformational heterogeneity of the protein complexes under study^14,22,23^.

**Figure 1:**
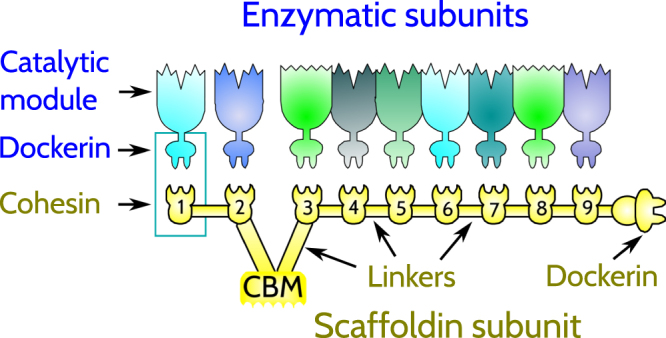
Schematic representation of cellulosome organization in *C. thermocellum*. The cellulosomal structural and enzymatic subunits comprise modular components. The cellulosomal enzymes each carry one or more catalytic modules and a single dockerin module. The scaffoldin is multifunctional, whereby the nine cohesin modules (enumerated) integrate nine dockerin-bearing enzymes into the complex, the carbohydrate-binding module (CBM) binds the complex to the cellulosic substrate, and the C-terminal dockerin module is involved in anchoring the cellulosome complex to the bacterial cell surface. The modules are separated from each other by defined linker segments.

The prototypic cellulosome-producing bacterium is *Clostridium thermocellum*. The typical architecture of the cellulosome that it makes is shown in Fig. 1. Its major scaffoldin, commonly denoted by CipA^24^, consists of nine type-I Cohs that show large sequence similarity. The corresponding Docs also exhibit large sequence similarity. However, the similarity is specific to a given organism. Also, the numbers of the Coh modules in various organisms are distinct and range from only two Cohs in the scaffoldin of *Clostridium saccharoperbutylacetonicum*^25^ to eleven Cohs in the major scaffoldin of *Bacteroides cellulosolvens*^26,27^. The scaffoldins in many mesophilic bacteria commonly bear five or six Cohs^25^.

The question we address here is whether binding of a given Doc to Coh occurs in a variety of ways or just one. Structural analyses of the Coh-Doc complex (derived from three organisms: *C. thermocellum*^28,29^, *C. cellulolyticum*^30^ and *Ruminococcus flavefaciens*^31^) indicate a possibility of a dual binding that would reduce the conformational constraints within dockerins, facilitating access to the substrate and, thus, enhancing the efficiency of degradation. These structural analyses are further supported by computational and biochemical studies on the contributions of distinct amino acid residues to the Coh-Doc binding affinity^32^.

The dual binding^33^ bears some similarities to domain swapping^34–36^. It should be noted, however, that these two phenomena are distinct. Firstly, the free energy difference between the monomers and the domain-swapped oligomers are affected by such factors as conformational changes at the inter-domain linker, or hinge^36^. In contrast, the dual binding implies equal free energies in the two binding modes, which precludes crystallization. Secondly, the nature of dual binding is dynamical–the two binding modes may exchange in time. On the other hand, domain swapping is essentially fixed just after two ribosome-borne chains combine^34,35^.

**Figure 2:**
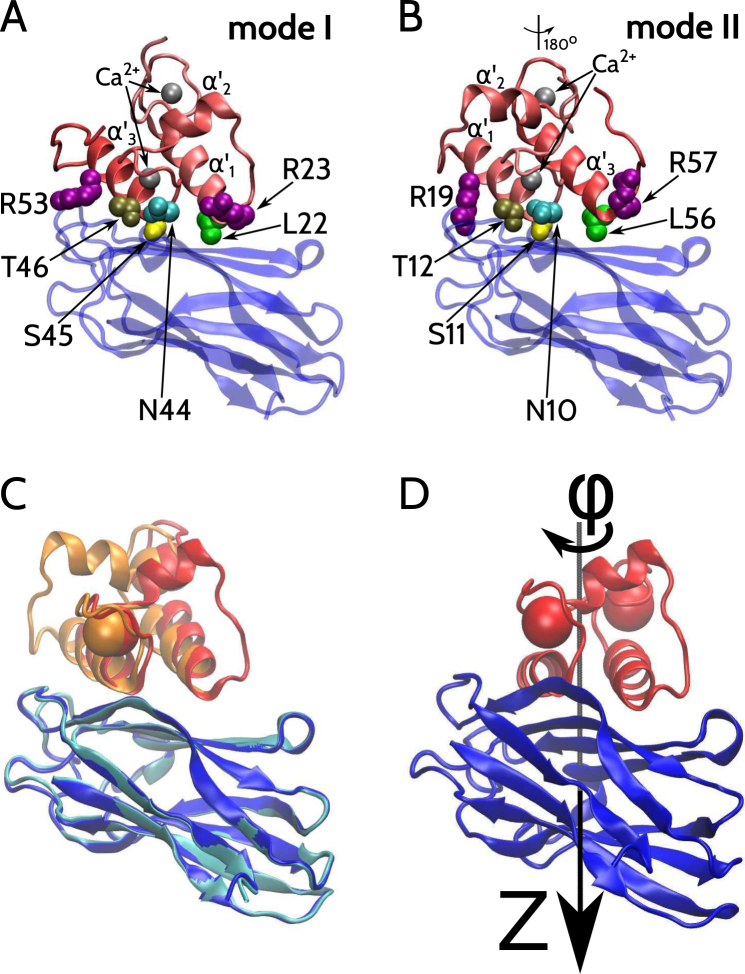
The top panels show structures of the Coh-Doc complex bound in mode I (**A**) and II (**B**). Coh and Doc are shown in blue and red, respectively. The spatial orientation of Coh is the same on both panels. The structure of Doc on panel B (mode II) is rotated by 180° relative to the structure of Doc on panel (A) (mode I). The three *α*-helices forming the Doc domain are labeled as ${\alpha }_{1}^{\prime}$, ${\alpha }_{2}^{\prime}$ and ${\alpha }_{3}^{\prime}$. The Doc-bound Ca^2+^ ions are shown as gray spheres. The Doc residues making contacts with the Coh in the binding mode I include R23, L22, N44, S45, T46 and R53. They are shown in the van der Waals representation. The Doc residues making contacts with the Coh in mode II include R57, L56, N10, S11, T12 and R19. The locations of R57, L56, N10, S11, T12 and R19 in mode II are analogous, respectively, to the positions of R23, L22, N44, S45, T46 and R53 in mode I. Panel (C) shows structures of the Coh-Doc complex given by the PDB:1OHZ (Coh in blue, Doc in red) and PDB:2CCL (Coh in cyan, Doc in orange). The Coh structures are superimposed. The spheres represent the dockerin-bound Ca^2+^ ions. The Doc helices are not aligned, indicating the existence of the two different binding modes. Panel D shows the PDB structure 1OHZ. Coh and Doc are shown in blue and red, respectively. The black line shows the derived axis of the symmetry, denoted here as the *Z*-axis. The sense of the rotation is indicated at the top.

The structure of Doc involves three *α*-helices, which are denoted here as ${\alpha }_{1}^{\prime}$, ${\alpha }_{2}^{\prime}$ and ${\alpha }_{3}^{\prime}$, as shown in panels A and B of Fig. 2. The idea of dual binding stems from a doubled loop-helix sequence in the Doc, wherein both sequence pieces contain a calcium-binding motif and repeated amino acid residues that can interact with the Coh in a similar manner, as illustrated in panels A and B of Fig. 2. As a result of this doubled loop-helix sequence, Doc may bind to Coh through the first and third of its helices but there are two ways in which this could happen, which differ by a rotation of approximately 180°, as shown in Fig. 2. For *C. thermocellum*, the first way, denoted here as mode I, is evidenced by the structure PDB:1OHZ, which has been derived for the wild-type (WT) complex^28^ involving the second Coh (c2A) of CipA^37^. The second way, denoted here as mode II, is exemplified for the same Coh by the structure PDB:2CCL, which has been derived for the complex in which Doc has undergone two single-site mutations (S45A and T46A) at the C-terminal helix^29^. The two structures are shown superimposed in panel C of Fig. 2. Despite the existence of the mutations, the two molecules are structurally very close. When the Docs of 1OHZ and 2CCL are sequence-aligned and superimposed, their *α*-C RMSD is equal to 0.42 Å. A similar alignment and superposition of the Cohs yields the RMSD of 0.40 Å. However, the whole complexes differ in RMSD by about 9 Å.

Two distinct binding modes, which resemble those observed in the crystal structures PDB:1OHZ and PDB:2CCL, have been identified in coarse-grained molecular dynamics simulations of the Coh-Doc association process^38^. An experimental demonstration of the dual binding has been reported by Jobst *et al*.^39^. It involved single molecule force spectroscopy (SMFS) of a Coh-Doc exocellulase Cel48S complex from *C. thermocellum* flanked by other proteins and polymers. The experiment indicated existence of two kinds of stretching trajectories which was interpreted as evidence for the dual binding. However, our theoretical analysis^40^ has found a possible alternative explanation of the stretching data: for a given binding mode there are two kinds of stretching trajectories that reflect different structural transformations in Coh and Doc. It may also happen that the dual binding phenomenon does exist, but in each mode there are two kinds of the stretching trajectories. The two effects are mixed in the SMFS experiment since it is done without any control of the starting situation.

Thus we consider the existing evidence for the dual binding mode to be inconclusive and requiring further studies. In this paper, we provide another theoretical approach to the binding problem by analyzing the energy landscapes involved by using an empirical model known as FoldX^41,42^. In particular, we investigate the influence of the two-site mutation (S45A and T46A) on the energy of Coh-Doc interactions in the two binding modes. We also perform comparative all-atom simulations.

Recent experiments on the ScaA Doc module from *R. flavefaciens* show that physical interactions between an N-terminal Trp and a C-terminal Pro in Doc confer stability on it^43^. However, neither PDB:1OHZ nor PDB:2CCL, encompasses the corresponding residues at the N- and C-terminus of the *C. thermocellum* Doc. Both of these Coh-Doc structures lack the the N- and C-terminal tails in Doc. We modeled these terminal segments and, interestingly, discovered that they indeed have a substantial influence on the energy landscapes of the Coh-Doc complex. In the presence of the full-length N- and C-terminal tails in the WT Doc, the free energy of binding in modes I and II are found to be comparable, which is consistent with the fact that–despite widespread efforts–no crystal structure of the full-length *C. thermocellum* Coh-Doc complex has been ever resolved. In the absence of the terminal tails in the WT Doc, on the other hand, mode I is found to have a substantially lower free energy than mode II, which is consistent with the Coh-Doc structure PDB:1OHZ. The two-site mutation–independent of the presence or absence of the terminal tails in Doc–is found to shift the equilibrium significantly towards mode II, as exemplified by the crystal structure PDB:2CCL.

In addition to the free energy calculations, we also simulate an AFM-like stretching of the tail-extended Coh-Doc complex using a structure-based coarse-grained model^44–49^. Our particular implementation of the structure-based model is related conceptually to several other approaches^50–53^. It has been selected optimally out of 62 variants of structure-based models considered in ref.^54^ by making comparisons to experimental data on stretching. For a given binding mode, we still observe two types of force-extension patterns which reflect different scenarios of structural rearrangements during the stretching. Interestingly, the two terminal tails in Doc make the Coh-Doc complex more stable mechanically. In particular, the presence of the tails in Doc affects the probabilities of observing the different types of the force-extension patterns.

## Methods

### Structural models of Coh and Doc

The Docs present in the PDB:1OHZ and PDB:2CCL correspond to the C-terminal docking domain of endo-1,4-*β*-xylanase Y from *C. thermocellum*. According to the Uniprot database, entry P51584, this Doc contains 69 amino acid residues. In keeping with the convention used in the entries PDB:1OHZ and PDB:2CCL, these residues are numbered from −5 to 64. However, the Doc structures given in PDB:1OHZ and PDB:2CCL have different sequential lengths: PDB:1OHZ contains the Doc residues with numbers from 1 to 56 whereas PDB:2CCL–residues from -2 to 59. When analyzing the Docs without the terminal tails, we truncate the Doc structure given in PDB:2CCL to retain only the residues with the sequential numbers ranging from 1 to 56. Otherwise, the residues at sites -5, -4, -3 are P, P, and V respectively; at sites 60, 61, 62, 63, and 64–D, K, F, P, and V respectively. Also the sequences of Cohs given in the two PDB entries have different lengths: PDB:1OHZ contains the Coh residues numbered from 5 to 144 whereas PDB:2CCL comprises the Coh residues from 5 to 153. We thus discard the Coh residues 145 through 153 from PDB:2CCL (when dealing with or without the tails in Doc) so that both Coh structures have identical sequences in our analysis.

We consider eight Coh-Doc complexes that are defined in Table 1. Here, we introduce the following notation: The capital letter *C* denotes the structure of Coh with the amino acid sequence comprising residue numbers from 5 to 144. The small letter *d* denotes the truncated Doc with the sequence comprising residue numbers 1 through 56. The asterisk denotes the Doc sequence with the two single-point mutations, i.e., S45A and T46A. The subscripts I and II indicate that the protein structures taken as an initial state for energy calculations are PDB:1OHZ and PDB:2CCL, respectively. This convention implies, for example, that the arrangement denoted by *C*_*I*_*d*_*I*_ corresponds to the Coh-Doc corresponding to PDB:1OHZ, where the WT Doc makes a complex with Coh in mode I. Similarly, in the arrangement *C*_*II*_*d*_*I*_ the Coh of 2CCL is bound to the Doc of PDB:1OHZ.

**Table 1:** The Coh-Doc complexes without the terminal tails in the Doc. *C*_*I*_ and *C*_*II*_ denote the Coh structures derived from PDB:1OHZ and PDB:2CCL, respectively. Both *C*_*I*_ and *C*_*II*_ comprise residues with sequential numbers from 5 to 153. *d*_*I*_ and *d*_*II*_ denote the Doc structures derived from PDB:1OHZ and PDB:2CCL, respectively; they both comprise residues with sequential numbers from 1 to 56. The asterisk denotes the two single-point mutations (S45A and T46A) in Doc. The fifth column provides the index, *k*, associated with the complex. The sixth column lists the values of the minimal free energy, *G*_*min*_, corresponding to binding mode I whereas the seventh column–to mode II.

complex	Coh	Doc	Definition	Index *k*	Δ*G*_*min*_ [kcal/mol] mode I	Δ*G*_*min*_ [kcal/mol] mode II
*C*_*I*_ *d*_*I*_	1OHZ	1OHZ	1OHZ	1	−38.2	−28.7
*C*_*I*_ *d*_*II*_	1OHZ	2CCL (A45S,A46T)	Doc from 2CCL with reverse mutations	2	−35.1	−31.2
*C*_*II*_ *d*_*I*_	2CCL	1OHZ	Doc from 1OHZ	3	−35.6	−30.7
*C*_*II*_ *d*_*II*_	2CCL	2CCL (A45S,A46T)	Doc from 2CCL with reverse mutations	4	−37.2	−35.5
${C}_{I}{d}_{I}^{\ast }$	1OHZ	1OHZ (S45A,T46A)	1OHZ with two mutations in Doc	5	−36.0	−28.5
${C}_{I}{d}_{II}^{\ast }$	1OHZ	2CCL	Doc from 2CCL	6	−35.0	−31.9
${C}_{II}{d}_{I}^{\ast }$	2CCL	1OHZ (S45A,T46A)	Doc from 1OHZ with two mutations	7	−34.1	−29.2
${C}_{II}{d}_{II}^{\ast }$	2CCL	2CCL	2CCL	8	−31.0	−34.0

We also study systems in which the Doc of PDB:1OHZ undergoes the two-site mutation to make it sequentially identical to the Doc of PDB:2CCL. Such Doc is denoted in Table 1 by ${d}_{I}^{\ast }$. In addition, we consider systems with the Doc derived from PDB:2CCL in which the original mutation is reverted by implementing the substitutions A45S and A46T. Such Doc is denoted in Table 1 as *d*_*II*_. Therefore, *C*_*I*_*d*_*II*_ corresponds to the Coh-Doc complex in which the Coh of PDB:1OHZ is bound to the Doc of PDB:2CCL in which the reverse mutations A45S and A46T have been made. Finally, the arrangement ${C}_{II}{d}_{II}^{\ast }$ corresponds to PDB:2CCL, where the mutated Doc makes a complex with Coh in mode II.

Since neither PDB:1OHZ nor PDB:2CCL contains the N- and C-terminal tails of the Docs, we model these terminal segments in two ways: by using the Swiss Model^55^ and iTASER^56^. Because of the uncertainty involved in protein structure modeling, in addition to using the two methods, we also consider three different PDB entries as template structures. They are listed in Table 2. The twelve resulting models of the Coh-Doc complex with the tails are summarized in Table 2. We use the convention in which the capital letter *D* denotes the full-length Doc which comprises the amino acid residues with the sequential numbers from −5 to 64. As before, the asterisk denotes the Doc sequence with the two single-point mutations.

**Table 2:** The Coh-Doc complexes comprising the full-length Doc. As in Table 1, *C*_*I*_ and *C*_*II*_ denote the Coh structures derived from PDB:1OHZ and PDB:2CCL, respectively. *D*_*I*_ and *D*_*II*_ denote the full-length Doc structures associated with PDB:1OHZ and PDB:2CCL, respectively. They both comprise residues with sequential numbers from -5 to 64. The asterisk denotes the two single-point mutations (S45A and T46A) in Doc. The PDB-unavailable tail parts of the structures are derived either by using the Swiss Model or iTaser server, as listed in the fourth column. The fifth column indicates the PDB structure code of the templates used. The sixth column provides the index, *k*, associated with the complex. The seventh column lists the values of the minimal free energy corresponding to mode I of binding whereas the eighth column–to mode II.

complex	Coh	Doc	Server	Template	Index *k*	Δ*G*_*min*_ [kcal/mol] mode I	Δ*G*_*min*_ [kcal/mol] mode II
*C*_*I*_ *d*_*I*_	1OHZ	1OHZ	Swiss Model	4DH2	9	−25.2	−14.3
*C*_*II*_ *d*_*II*_	2CCL	2CCL (A45S,A46T)	Swiss Model	4DH2	10	−26.4	−28.4
*C*_*I*_ *d*_*I*_	1OHZ	1OHZ	iTASER	4DH2	11	−29.5	−18.4
*C*_*I*_ *d*_*I*_	1OHZ	1OHZ	iTASER	1OHZ	12	−23.6	−15.5
*C*_*II*_ *d*_*II*_	2CCL	2CCL (A45S,A46T)	iTASER	4DH2	13	−28.0	−28.7
*C*_*II*_ *d*_*II*_	2CCL	2CCL (A45S,A46T)	iTASER	2CCL	14	−26.0	−29.9
${C}_{I}{D}_{I}^{\ast }$	1OHZ	1OHZ	Swiss Model	4DH2	15	−24.2	−13.5
${C}_{II}{D}_{II}^{\ast }$	2CCL	2CCL	Swiss Model	4DH2	16	−29.6	−29.1
${C}_{I}{D}_{I}^{\ast }$	1OHZ	1OHZ	iTASER	4DH2	17	−28.0	−18.5
${C}_{I}{D}_{I}^{\ast }$	1OHZ	1OHZ	iTASER	1OHZ	18	−22.8	−13.0
${C}_{II}{D}_{II}^{\ast }$	2CCL	2CCL	iTASER	4DH2	19	−29.0	−30.4
${C}_{II}{D}_{II}^{\ast }$	2CCL	2CCL	iTASER	2CCL	20	−26.7	−32.1

As we explain in the subsequent paragraph, it is possible to determine a symmetry axis, *Z*, such that a rotation of Doc around *Z* transforms the Coh-Doc structure between the two binding modes. The geometry involved is illustrated in panel D of Fig. 2. Here, *φ* is the rotation angle around the *Z*-axis and the coordinates of Coh-Doc at *Z* = 0 and *φ* = 0 correspond to PDB:1OHZ. Similarly $Z\approx 0$ and $\phi \approx \pi$ correspond to PDB:2CCL. We use the convention in which positive values of *Z* correspond to shifting the two molecules closer together and negative values–to shifting them further away. The rotations and forward shifts are not implemented when prohibited by steric constraints.

The secondary structure of Doc consist of three helices: ${\alpha }_{1}^{\prime}$ constituted by the residues with numbers from 11 to 23, ${\alpha }_{2}^{\prime}$ formed by the residues ranging from 28 to 36, and ${\alpha }_{3}^{\prime}$ comprising residues from 45 to 56. Here, in keeping with the convention used in refs^40,57^ the primed and unprimed symbols indicate the secondary structures belonging to Doc and Coh, respectively. In each of the binding modes only the first and third of these helices couple to Coh, which implies that the symmetry axis can be determined based only on ${\alpha }_{1}^{\prime}$ and ${\alpha }_{1}^{\prime}$. To determine the symmetry axis we use the following procedure: (i) We superimpose the Cohs of PDB:1OHZ and PDB:2CCL. (ii) We introduce a set of vectors formed by pairs of equivalent *α*-C atoms in helices ${\alpha }_{1}^{\prime}$ and ${\alpha }_{3}^{\prime}$ as described in PDB:1OHZ and PDB:2CCL. (iii) We rotate the coordinate system in such a way that the *Z*-components of these vectors are brought closer to zero. (iv) We iterate step (iii) to bring the *Z*-components of these vectors to zero. The *Z*-axis of the final, rotated coordinate system gives the symmetry axis. We find that switching from mode I to mode II requires a rotation around the *Z*-axis by *φ* = 174°. Rotating by 360° brings the system back to mode I.

### Free energy calculations using FoldX

The purpose of our calculations is to determine the free energy of the Coh-Doc system as a function of parameters *Z* and *φ*, which describe the location of Doc relative to Coh, as depicted in panel D of Fig. 2. One way to determine the energy landscape associated with the protein complex would be by using all-atom simulations and averaging over classes of conformations. However, this procedure would be way too costly numerically when repeating it for various values of *Z* and *φ*, even when adopting an implicit solvent approach. Instead, we use an empirical force field known as FoldX^41,42^ which has been designed primarily for predicting free energy differences between a WT protein and its mutant. Here, however, the distinct structures correspond to different positions of Doc relative to Coh. We predict the structures and their free energies as a function of *Z* and *φ*. The prediction also applies to the Coh-Doc complexes with the mutated (S45A and T46A) or reverse-mutated (A45S and A46T) Doc. We consider the systems both with and without the tails in the Doc. The effects of the dockerin-bound calcium ions are included in the calculations. FoldX employs an energy function that consists of ten terms that are listed in Supplementary Information (SI).

We perform the free energy calculations for two temperatures: *T* = 298 K, which is the FoldX default parameter, and *T* = 308 K, i.e., the maximum temperature for which the FoldX energy function has been parametrized. The free energy is optimized with respect to the side-chain conformations while the backbone atoms are kept at fixed positions. When not considering the sequence tails in Doc, the initial structures for the free energy minimization are as in the PDB structure files, as listed in Table 1. Otherwise, the structures are predicted by iTaser or the Swiss Model, as specified in Table 2. In all cases, the dockerin-bound calcium ions are included in the structural models and taken into account in the FoldX energy calculations^58^.

The resulting Δ*G*, minimized with respect to the side-chain orientation, is the free energy subject to the constraints on the positions of the backbone atoms of Coh and Doc. Therefore, Δ*G* depends not only on the coordinates *Z* and *φ* but also on the structural model taken as input for the calculations. In particular, selecting the lowest free energy is subject to a substantial uncertainty and, perhaps more importantly, does not provide a proper estimate of the binding strenght because it does not involve any information about the lateral extension of the minimal basins.

In order to estimate the free energy of binding in a more robust way, we perform a second stage of calculations in which Δ*G* determined for various complexes corresponding to the same sequence (mutated or not) serve as an input. In the case of Docs with tails, we also include the various of determining the structure. Specifically, we define the free energy of Coh-Doc binding in mode I as

1 $${F}_{I}=-{k}_{B}T\,\mathrm{log}[\sum _{k}\sum _{Z}\sum _{-\pi \mathrm{/2 < }\phi  < \pi \mathrm{/2}}\exp (-{\rm{\Delta }}{G}_{k}(Z,\phi )/{k}_{B}T)\theta ({E}_{c}-{\rm{\Delta }}{G}_{k})].$$

Here, the sum over index *k* corresponds to averaging over the input structures listed in Tables 1 or 2. The values of *k* are written in the last columns of these tables. Accordingly, ${\rm{\Delta }}{G}_{k}(Z,\phi )$ denotes ${\rm{\Delta }}G(Z,\phi )$ computed for the input structure with index *k*. The unit step function*, θ*, is defined as follows: $\theta (x)=1$ for *x* > 0 and $\theta (x)=0$ for *x* < 0. Thus the configurations (*Z*, *φ*) corresponding to the binding mode I are those with $-\pi /2 < \phi  < \pi /2$ and free energies Δ*G*_*k*_ smaller than a cut-off value *E*_*c*_. As we show in the Results section, *E*_*I*_ does not depend on the choice of the cut-off as long as *E*_*c*_ is larger than about −25 kcal/mol.

By analogy, the free energy of Coh-Doc binding in mode II is

2 $${F}_{II}=-{k}_{B}T\,\mathrm{log}[\sum _{k}\sum _{Z}\sum _{\pi \mathrm{/2 < }\phi \mathrm{ < 3}\pi \mathrm{/2}}\exp (-{\rm{\Delta }}{G}_{k}(Z,\phi )/{k}_{B}T)\theta ({E}_{c}-{\rm{\Delta }}{G}_{k})].$$

The configurations (*Z*, *φ*) corresponding to the binding mode II are those with $\pi /2 < \phi  < 3\pi /2$ and energies Δ*G*_*k*_ smaller than a cut-off value *E*_*c*_.

If the equilibrium probability of finding the Coh-Doc complex in mode I is denoted by *p*_*I*_ and in mode II by *p*_*II*_ then

3 $${p}_{I}/{p}_{II}=\exp [-({F}_{I}-{F}_{II})/{k}_{B}T].$$

The dual binding occures if the probabilities *p*_*I*_ and *p*_*II*_ are of the same order of magnitude.

### Free energy of binding by using all-atom simulations

The employment of the empirical models described above allows for the elucidation of the free energy landscape through the usage of the *Z* and *φ* variables. It also allows for a considerable probing of the side-chain conformations. However, the drawback involved is the lack of the flexibility of the backbone. It is thus worthwhile to compare the binding energies to those obtained through all-atom molecular dynamics (MD) simulations.

To this end, we used the Amber 14 package^59^ with the AMBER force field 99SB^60^ and the TIP3P model for the molecules of water^61^. The Newton equations of motion were integrated by using the leapfrog algorithm with the time step of 2 fs. All bonds with the hydrogen atoms were constrained through the SHAKE algorithm^62^. The *T* was maintained around 300 K by adding terms corresponding to the Langevin dynamics^63^ with the collision frequency of 2 ps^−1^. A 10 Å cut-off was applied to all non-bonded interactions. The Particle Mesh Ewald method^64^ was used to treat the long-range electrostatics.

The Coh-Doc complex (shown in Fig. S1 in SI) was solvated in a truncated octahedron box of water that is large enough to avoid interactions between the protein complex and its images in the adjacent cells. The system was neutralized by adding Na^+^ ions. The energy of the system was then minimized in three stages: the first minimization was done with all atoms of the protein complex being restrained to let the water molecules move to empty places around the proteins; in the second minimization stage, only the backbone heavy atoms are restrained; and in the last stage, no restraints were applied. Each minimization stage involved 2500 steepest descent steps that removed steric clashes and then 2500 conjugate gradient steps to achieve quick convergence. After the energy minimization, the system was heated up from 0 to 300 K in 2.5 × 10^4^ steps with weak restrains applied to the atoms of the Coh-Doc complex. This was followed by 2.5 × 10^4^ relaxation steps at *T* = 300 K (with the restrains kept in place), which brought the system to the density of 1 g/cm^3^, and then by 2.5 × 10^5^ steps of equilibration without any restrains. Both the energy minimization and the heating-up simulations were performed at constant volume. The subsequent relaxation and equilibration simulations were performed at constant pressure *p* = 1 atm.

In the production runs, pressure was also maintained at *p* = 1 atm. The coordinates of all atoms of the Coh-Doc complex were recorded every 10 ps. Five independent trajectories of 200 ns were generated for each of the systems under study. For the recorded conformations of the complex, the root-mean-square distance (RMSD) from the native structure was computed by considering only the backbone atoms. An example of the dependence of RMSD on time is shown in Fig. S2 in SI. Based on the RMSD plots we selected, for each of the trajectories independently, a time interval in which RMSD attained a steady state and fluctuations in RMSD did not exceed 2 Å. The Coh-Doc conformations recorded in these time intervals were taken as input for implicit-solvent free-energy calculations, which were performed within the framework of the Molecular Mechanics/Poisson Boltzmann Surface Area (MM/PBSA) method.

The MM/PBSA method is based on calculating the free energy difference, Δ*G*_*bind*_, between the bound and unbound states

$${\rm{\Delta }}{G}_{bind}={G}_{Coh-Doc}-{G}_{Coh}-{G}_{Doc}={\rm{\Delta }}{E}_{ele}+{\rm{\Delta }}{E}_{vdW}+{\rm{\Delta }}{G}_{PB}+{\rm{\Delta }}{G}_{SUR}-T{\rm{\Delta }}S.$$

Here, ${\rm{\Delta }}{E}_{ele}$ and ${\rm{\Delta }}{E}_{vdW}$ denote the electrostatic and van der Waals contributions. These energy contributions are the same as in the AMBER force field used in the MD simulations. ${\rm{\Delta }}{G}_{PB}$ and ${\rm{\Delta }}{G}_{SUR}$ are the polar and non-polar solvation energy terms. The entropic contribution, *T*Δ*S*, is estimated by the normal mode approximation method using the *mmpbsa_py_nabnmode* program implemented in the AMBER package. The solvation energy was calculated by *pbsa*, which is also included in the AMBER package. The polar term, ${\rm{\Delta }}{G}_{PB}$, was obtained by solving linearized Poisson-Boltzmann equation numerically. The non-polar term is defined by ${\rm{\Delta }}{G}_{SUR}=\alpha SASA+\beta$, where SASA is the solvent-accessible surface area that was calculated by the LCPO method^65^. The regression coeficients *α* and *β* are set to 0.005 kcal mol^−1^ Å^−2^ and 0, respectively. For a given MD trajectory, ${\rm{\Delta }}{G}_{bind}$ is averaged over the selected time interval. The resulting values of time-averaged ${\rm{\Delta }}{G}_{bind}$ are summarized in Tables S1–S5 in SI.

### Structure-based coarse-grained simulations

We use a structure-based coarse-grained model^44–49^ in which amino acid residues are represented by single beads centered on their *α*-C atoms. The beads are tethered together into chains by strong harmonic potentials with the spring constant ${k}_{{\rm{b}}{\rm{o}}{\rm{n}}{\rm{d}}}=100\,\varepsilon /{\mathop{\text{A}}\limits^{\circ }}^{2}$, where *ε* is the depth of the potential well associated with the native contacts, which serves as the basic energy scale in our model. We assume $\varepsilon =\mathrm{(110}\pm \mathrm{30)}$ pN Å, as determined by benchmarking against experimental results for 38 proteins^45^. The native contacts are identified using an overlap criterion^49^ applied to the coordinates of all heavy atoms in the native structure. Here, the van der Waals radii of the heavy atoms are taken from ref.^66^ The effective spheres associated with the atoms, when checking for the overlaps, have radii which are 1.24 times larger^67^ (this factor corresponds to the point of inflection in the Lennard-Jones potential). In addition, the amino acid pairs that are very close sequentially, (*i*, *i* + 1) and (*i*, *i* + 2), are excluded from the contact map. Examples of contact maps for Doc and Coh-Doc are shown, respectively, in Figs S3 and S4 in SI.

The interactions within the native contacts are described by the Lennard-Jones potential

$${V}^{{\rm{N}}AT}({r}_{ij})=4\varepsilon \,[{(\frac{{\sigma }_{ij}}{{r}_{ij}})}^{12}-{(\frac{{\sigma }_{ij}}{{r}_{ij}})}^{6}]$$

Here, *r*_*ij*_ denotes the distance between residue beads *i* and *j*. The parameters *σ*_*ij*_ are chosen so that each contact in the native structure is stabilized at the minimum of the Lennard-Jones potential. The contacts between the proteins are treated in the same manner as the contacts within the proteins as both sets are dominated by hydrogen bonds. The contacts between the dockerin and the Ca^2+^ ions are determined on the basis of the overlap criterion^49^ with the van der Waals radius of Ca^2+^ being 1.53 Å, as in the AMBER force-field^68^. The interactions between the residues that do not form native contacts are purely repulsive and given by the truncated and shifted Lennard-Jones potential corresponding to ${\sigma }_{ij}={r}_{0}/\sqrt[6]{2}$ with ${r}_{0}=4$ Å. The energy function comprises also harmonic terms that favor the native values of local chiralities in each amino acid chain^69^.

The solvent is implicit and the system evolves in time according to the Langevin dynamics. The overall force acting on a particular bead *i* is a sum of three terms: (i) the direct force ${\vec{F}}_{i}$ that derives from all the potential energy terms, (ii) the damping force that is proportional to the velocity of the bead, and (iii) the random force, ${\vec{{\rm{\Gamma }}}}_{i}$, that represents thermal noise. The corresponding equations of motion, $m\frac{{{\rm{d}}}^{2}{\vec{r}}_{i}}{{\rm{d}}{t}^{2}}={\vec{F}}_{i}-\gamma \frac{{\rm{d}}{\vec{r}}_{i}}{{\rm{d}}t}+{\vec{{\rm{\Gamma }}}}_{i}$, are solved by the fifth-order predictor-corrector algorithm with the time step of 0.005 *τ*. Here, *γ* is the damping coefficient, and all beads are assumed to have the same mass *m*. The dispersion of the thermal noise is given by $\sqrt{2\gamma {k}_{B}T\,}$, where *k*_*B*_ is the Boltzmann constant. The damping coefficient is set to $\gamma =2m/\tau$. This value corresponds to the overdamped case–practically Brownian dynamics–and the characteristic time scale, *τ*, is of the order of 1 ns, as argued in refs^70,71^.

#### Thermal stability of dockerin

We use the structure-based coarse-grained simulations to investigate the thermal stability of the Docs with and without the terminal tails. To this end, we compute the probability of finding the Doc in the native state, *P*_0_, as a function of *T*. The definition of *P*_0_ involves counting the conformations in which all native contacts are present. The native state probability *P*_0_ is thus different from the average fraction of native contacts, *Q*, which is often used to characterize the deviation from the native state.

To compute *P*_0_(*T*) and *Q*(*T*), we perform MD simulations for *T* ranging from $0.02\,\varepsilon /{k}_{B}$ to $1.16\,\varepsilon /{k}_{B}$. Each MD simulation is preceded by a 10^4^*τ* equilibration run and gives $3\times {10}^{5}\,\tau$ of dynamics. At any given *T*, we run 101 independent trajectories. *P*_0_ is then determined as an average over the simulation time and over the trajectories. We perform the simulations for the Docs with the terminal tails (index *k* = 1) and without the terminal tails (index *k* = 9, 11 and 12).

Another way to assess thermal stability of proteins is to simulate their unfolding at elevated temperatures. The unfolding simulations start at the native state and finish when all nonlocal contacts get broken, which defines the unfolding time *t*_unf_. Specifically, the nonlocality refers to the sequential distance $|i-j| > 4$. At any given *T* between $0.9\,\varepsilon /{k}_{B}$ and $2.1\,\varepsilon /{k}_{B}$, we run 301 independent trajectories of 10^5^ *τ* each and, thus, obtain 301 values of *t*_unf_. As the characteristic unfolding time, *t*_*u*_, we take the median of the distribution of unfolding times *t*_unf_. We perform the simulations for the Doc with the tails (*k* = 1) and without the tails (*k* = 9,11 and 12).

#### Stretching simulations of the Coh-Doc complex

We use the structure-based coarse-grained simulations to investigate also the mechanical stability of the Coh-Doc complex. Stretching of the Coh-Doc complex is implemented by attaching two harmonic springs to the N-terminal amino acids of Coh and Doc. (Other ways of pulling are discuss in ref.^40^). The N-terminus of Doc is denoted as N’ and the C-terminus as C’. One of the springs is fixed in space and the other one is moved at a constant speed, *v*_*p*_, so that the distance it travels in time *t* is $d={v}_{p}t$. The force constant of the pulling springs is taken as $K=0.12\,\varepsilon /{\mathop{\text{A}}\limits^{\circ }}^{2}$, which corresponds to about 1 pN/nm and is close to the elasticity of typical AFM cantilevers^45^. All pulling simulations are performed for $T=0.3\,\,\varepsilon /{k}_{B}$, which is near-optimal in folding kinetics and is of the order of room temperature.

In our simulations, the response force *F* acting on the pulling spring is measured and averaged over time periods that correspond to the spring displacements of 0.5 Å^45^. The *F*-*d* curves may come with several peaks, and the height of the largest of them is denoted by *F*m*ax*. As in ref.^40^ we perform simulations for the pulling speed ${v}_{p}=5\times {10}^{-5}\mathop{\text{A}}\limits^{\circ }/\tau \approx 5$ nm/ms which is close to the experimental speeds.

All of the pulling simulations start from the native state. In the course of the simulations, the breaking and re-formation of native contacts is followed in time. The native contact between residues *i* and *j* is considered broken if the inter-residue distance *r*_*ij*_ exceeds a cutoff length of $1.5\,\,{\sigma }_{ij}$. Due to thermal fluctuations, the broken contacts may get re-established. To characterize the unfolding and dissociation patterns, we record the spring displacements at which the native contacts break for the last time.

### Data availability

All data generated or analysed during this study are included in this published article (and its Supplementary Information files).

## Results

### Energy landscapes of Coh-Doc interactions

#### Docs without the tails

**Figure 3:**
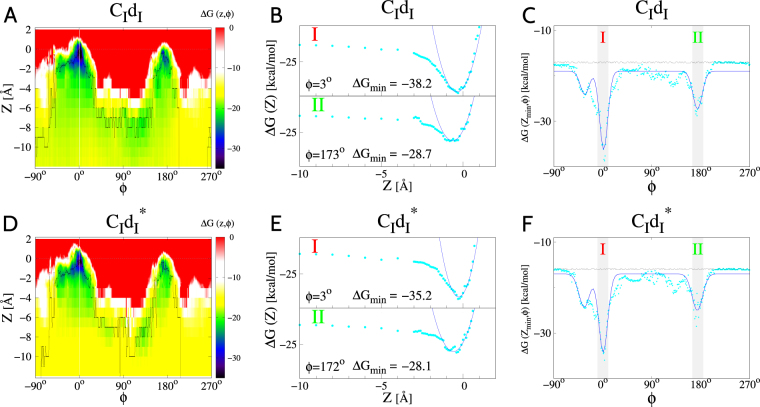
Results of the FoldX-based free-energy calculations for system *C*_*I*_*D*_*I*_ (panels A,B and C) defined in Table 1 for index *k* = 1, and for system ${C}_{I}{d}_{I}^{\ast }$ (panels D–F) corresponding to index *k* = 5 in Table 1. (**A**) Δ*G* as a function of coordinates *Z* and *φ*. The values of Δ*G* are indicated by the color scale shown on the right hand side. (**B**) Δ*G* as a function of *Z* for angles $\phi ={3}^{\circ }$ (top sub-panel, mode I) and $\phi ={173}^{\circ }$ (bottom sub-panel, mode II) at which ${\rm{\Delta }}G(Z,\phi )$ is found to take the minimal values, ${\rm{\Delta }}{G}_{{\rm{m}}in}=-38.2$ kcal/mol and ${\rm{\Delta }}{G}_{{\rm{m}}in}=-28.7$ kcal/mol, respectively. The data points in cyan show the results of the FoldX-based calculations. The solid blue lines correspond to a harmonic approximation at the minimum of Δ*G*(*Z*). (**C**) Δ*G* minimized with respect to *Z* and plotted as a function of *φ*. The data points in cyan show the results of the FoldX-based calculations. The solid blue line represents a fit to the data points. This line is a to guide the eye. The fitting function used here involves three Gaussians. Panels (D–F) are analogous to panels (A–C), respectively.

Figure 3 illustrates the FoldX-derived free-energy landscapes for systems *C*_*I*_*d*_*I*_ (panels A, B and C) and ${C}_{I}{d}_{I}^{\ast }$ (panels D, E and F). The panels A and D are color-maps showing Δ*G*_*k*_ for *k* = 1 and *k* = 5, respectively, as a function of *Z* and *φ*. (Here, Δ*G* is minimized with respect to the side-chain orientations for the input structures with indices *k* = 1 and *k* = 5). The value of Δ*G* is indicated by colors according to the scale bar on the right-hand side. The regions with smallest/largest values of Δ*G* are marked in blue/red. We note that there are two pronounced local minima in Δ*G*. The first one is located around *φ* = 0 (binding mode I), whereas second one is around *φ* = *π* (binding mode II). More precisely, for system *C*_*I*_*d*_*I*_, the two minima are located at *φ* = 3° and *φ* = 173°. They have different depths, −38.2 and −28.7 kcal/mol, and the parameter region corresponding to mode I has a larger area in the (*Z*, *φ*) plane. For system ${C}_{I}{d}_{I}^{\ast }$, the two minima are located at *φ* = 3° and *φ* = 173°. Their depths are −35.2 and −28.1 kcal/mol.

Panels B and E in Fig. 3 show the *Z*-dependence of Δ*G* for two fixed values of *φ*, i.e., for *φ* = 3°, corresponding to mode I (upper subpanels), and for *φ* = 173° (panel B) or *φ* = 172° (panel D), corresponding to mode II (lower subpanels). The data points are fitted to parabolas around the minima to help estimate their depth. Panels C and F in Fig. 3 show the *φ*-dependence at *Z* equal to the value at the deepest minimum, *Z*_*min*_. The solid lines are curves that are fitted to the data points.

The values of Δ*G*_*min*_, corresponding to the two binding modes for the eight complexes under study, are listed in Table 1, columns number five and six. For a given binding mode (I or II) and a given sequence (WT or two-site mutant), the values of Δ*G*_*min*_ depend on *k* because the positions of the backbone atoms are not identical in the structural models that are taken as input for the FoldX calculations. For the systems comprising the WT Doc, for which $k=\mathrm{1,}\ldots \mathrm{,4}$, the values of Δ*G*_*min*_ are observed to be scattered between −38.2 and −35.1 kcal/mol in mode I and between −35.5 and −28.7 kcal/mol in mode II. For the systems comprising the mutated Doc, with $k=\mathrm{5,}\ldots \mathrm{,8}$, they are seen to be between −36.0 and −31.0 kcal/mol in mode I and between −34.0 and −28.5 kcal/mol in mode II.

**Figure 4:**
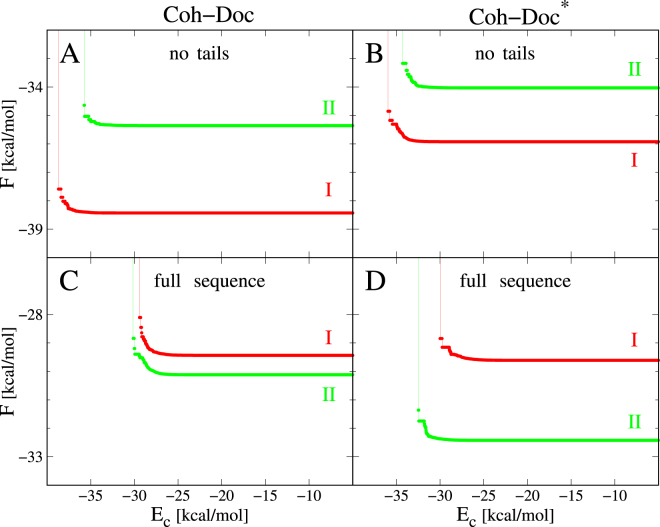
Free energies of binding in mode I (red) and II (green) as functions of the energy cut-off, *E*_*c*_, which is used to compute *F*_*I*_ and *F*_*II*_ according to Eqs (1) and (2). The top panels, A and B, correspond to the Doc without the tails (sequences comprising residues 1 through 56). The bottom panels, (C and D), correspond to the full-length Doc (sequences comprising residues −5 through 56). Four cases are considered: (**A**) Coh-Doc in which Doc is WT and without the tails. Here, *F*_*I*_ and *F*_*II*_ are computed by taking $k=\mathrm{1,}\ldots \mathrm{,4}$. (**B**) Coh-Doc^*^ in which Doc^*^ is the two-point mutant without the tails. The binding energies are computed by taking $k=\mathrm{5,}\ldots \mathrm{,8}$ in this case. (**C**) Coh-Doc in which Doc is WT and full-length, i.e., comprising residues −5 through 56. This case corresponds to averaging over $k=\mathrm{9,}\ldots \mathrm{,14}$ in Eqs (1) and (2). (**D**) Coh-Doc^*^ in which Doc^*^ is the two-point mutant with the tails, i.e., comprising residues −5 through 56. Structural models with $k=\mathrm{15,}\ldots \mathrm{,20}$ have been used for calculating *F*_*I*_ and *F*_*II*_ in this case.

The probability that Doc binds Coh in mode I or II is not determined only by the corresponding values of Δ*G*_*min*_ but also by the widths of the two valleys in the Coh-Doc energy landscape. For this reason we consider the binding free energies *F*_*I*_ and *F*_*II*_ as well as the binding probabilities *p*_*I*_ and *p*_*II*_ as defined by Eqs (1–3) with the condition that *p*_*I*_ + *p*_*II*_ = 1. The binding free energies *F*_*I*_ and *F*_*II*_ for the WT sequence are given by Eqs (1 and 2) with $k=\mathrm{1,}\ldots \mathrm{,4}$. By analogy, the binding free energies *F*_*I*_ and *F*_*II*_ for the two-site mutant are given by Eqs (1 and 2) with $k=\mathrm{5,}\ldots \mathrm{,8}$. Figure 4A shows *F*_*I*_ and *F*_*II*_ as a function of the energy cut-off *E*_*c*_ for the WT case. We observe that *F*_*I*_ and *F*_*II*_ saturate above a threshold of about −25 kcal/mol, and we take the saturation values as estimates of the binding energies (from now on *F*_*I*_ and *F*_*II*_ denote the saturation values).

For the WT system, we obtain *F*_*I*_ = −38.5 kcal/mol and *F*_*II*_ = −35.3 kcal/mol which yields ${p}_{I}/{p}_{II}\approx 200$, indicating a strong preference for binding in mode I. On the other hand, for the mutated system we get *F*_*I*_ = −35.9 kcal/mol and *F*_*II*_ = −34.0 kcal/mol, as can be seen in Fig. 4B. According to Eq. (3), the probability of binding mode I to the probability of binding mode II is ${p}_{I}/{p}_{II}\approx 20$. Thus mode I is still more preferred than mode II, but the equilibrium shifts towards mode II. It should be noted, however, that FoldX does not strictly endorse mode II for the mutated sequence that is evidenced by PDB:2CCL.

The MM/PBSA calculations yield the following results: ${\rm{\Delta }}{G}_{bind}=-36.3\pm 2.9$ kcal/mol for the binding of the WT Doc to the Coh in mode I, i.e., for the Coh-Doc system with index *k* = 1, and ${\rm{\Delta }}{G}_{bind}=-34.5\pm 2.9$ kcal/mol for the binding of the mutated Doc the Coh in mode II, i.e., for the Coh-Doc system with index *k* = 8. These binding energies are comparable within the statistical error. They are also consistent with the binding free energies obtained from the FoldX calculations.

#### Docs with the tails

**Figure 5:**
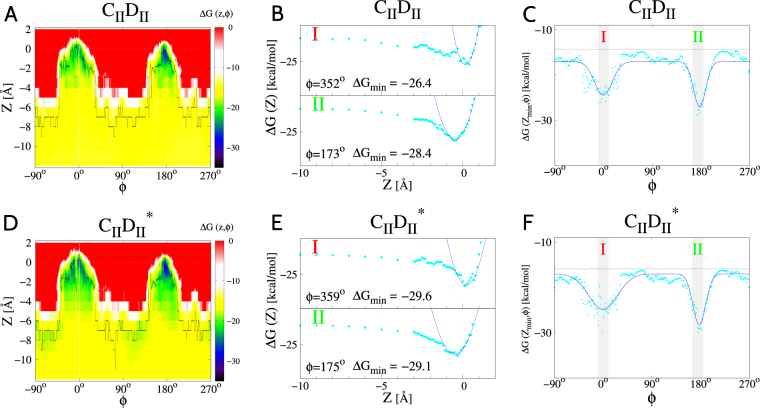
Analogous to Fig. 3 but for system *C*_*II*_*D*_*II*_ (panels A–C) corresponding to index *k* = 10 in Table 2, and for system ${C}_{II}{D}_{II}^{\ast }$ (panels D–F) corresponding to index *k* = 16 in Table 2.

Figure 5 illustrates the FoldX-derived free-energy landscapes for systems *C*_*II*_*D*_*II*_ (*k* = 10; panels A, B and C) and ${C}_{II}{D}_{II}^{\ast }$ (*k* = 16; panels D, E and F). Overall, they resemble the energy landscapes obtained for systems *C*_*I*_*D*_*I*_ and ${C}_{I}{d}_{I}^{\ast }$, in which the Docs lack the terminal tails (compare with Fig. 3). The noticeable differences are slight shifts in the minimum-energy angles *φ*. In the case of system *C*_*II*_*D*_*II*_, Δ*G*_*min*_ favors binding mode II by 2 kcal/mol. In the case of system ${C}_{II}{D}_{II}^{\ast }$, however, Δ*G*_*min*_ for mode I is smaller by 0.5 kcal/mol than Δ*G*_*min*_ for mode II. The values of Δ*G*_*min*_ corresponding to modes I and II for *k* between 9 and 20 are listed in Table 2.

The binding free energies for the WT system ($k=\mathrm{9,}\ldots \mathrm{,14}$) are *F*_*I*_ = −29.2 kcal/mol and *F*_*II*_ = −30.2 kcal/mol, as can be seen in Fig. 4C. Thus mode II has a somewhat lower free energy than mode I: *F*_*I*_–*F*_*II*_, is only of order of 0.5 kcal/mol. This implies that ${p}_{I}/{p}_{II}\approx 0.4$, which suggests that the WT full-length Doc should bind its partner in modes I and II with comparable probabilities. This result rationalizes the fact that no crystal structure of the full-length Coh-Doc WT complex has ever been solved. When the sequence is truncated, mode I is preferred, and that is why it was crystallized as PDB:1OHZ. It should be noted that none of our predicted models indicates any presence of flexible or disordered parts in the tails. Such a flexibility would provide the usual mechanism of not having a well defined structure.

For the system with the double-site mutation $(k=\mathrm{15,}\ldots \mathrm{,20})$, we get *F*_*I*_ = −29.6 kcal/mol and *F*_*II*_ = −32.4 kcal/mol, as can be seen in Fig. 4D, which implies ${p}_{I}/{p}_{II}\approx 0.01$ according to Eq. (3). This result means that the two-site mutation shifts the equilibrium towards mode II, from ${p}_{I}/{p}_{II}\approx 0.4$ to ${p}_{I}/{p}_{II}\approx 0.01$. Thus mode II is clearly dominating in this case and probably could be crystallized, if attempted.

We also use the MM/PBSA method to compute the binding free energy between the Coh and the Doc with the tails. For the system in which the Coh binds in mode I to the WT Doc, we obtain ${\rm{\Delta }}{G}_{bind}=-39.4\pm 7.2$ kcal/mol. For the system in which the Coh binds in mode II to the mutated Doc, the MM/PBSA method yields ${\rm{\Delta }}{G}_{bind}=-43.6\pm 6.0$ kcal/mol. The difference in the binding free energies is only about 4 kcal/mol and, thus, is comparable to the statistical error on Δ*G*_*bind*_. Therefore, one can not state with confidence which of the two systems is bound more tightly. However, the difference between *F*_*II*_ for the full-length Coh-Doc* complex and *F*_*I*_ for the full-length Coh-Doc complex, as obtained from the FoldX calculations, is about 3 kcal/mol, which compares well with the results obtained in the framework of the MM/PBSA method.

For the WT Coh-Doc complex in the binding mode I and II, the MM/PBSA method yields ${\rm{\Delta }}{G}_{bind}=-39.4\pm 7.2$ kcal/mol and ${\rm{\Delta }}{G}_{bind}=-45.5\pm 7.3$ kcal/mol, respectively. The difference in these two energies is smaller than the statistical error of the MM/PBSA calculations, which supports the hypothesis of dual binding. We also note that truncation of the terminal tails from the Doc leads to an increase inΔ*G*_*bind*_ from −39.4  ± 7.2 kcal/mol to −36.3 ± 2.9 kcal/mol for the WT system in mode I, and from −43.6 ± 6.0 kcal/mol to −34.5 ± 2.9 kcal/mol for the mutated system in mode II. These results indicate that the presence of the tails in the Doc enhances the Coh-Doc binding affinity.

#### The effects of heating

We have used FoldX to perform analogous free-energy calculations for *T* = 308 K, which is 10 K higher than the room temperature considered so far. The results of these calculations are shown in Fig. S5 in SI. For the Coh in complex with the Doc without the tails, we obtain the following free-energy values: *F*_*I*_ = −30.8 kcal/mol and *F*_*II*_ = −26.4 kcal/mol for the WT system (Fig. S5A) and *F*_*I*_ = −28.6 kcal/mol and *F*_*II*_ = −26 kcal/mol for the mutated system (Fig. S5B). Therefore, mode I is seen to be dominating in both cases. For the Coh in complex with the Doc containing the tails, we obtain the following results: *F*_*I*_ = −20.8 kcal/mol and *F*_*II*_ = −21.9 kcal/mol for the WT system (Fig. S5C) and *F*_*I*_ = −22.8 kcal/mol and *F*_*II*_ = −23.7 kcal/mol for the mutated one (Fig. S5D). These results show that mode II is somewhat more favorable than mode I but the free-energy difference between the two modes is only of the order of 1 kcal/mol. We thus conclude that the dual binding persists at *T* = 308 K.

The results of coarse-grained simulations shown in Fig. S6 indicate that the WT Doc with the tails is thermally more stable than without the tails. For instance, the characteristic time of thermal unfolding, *t*_*u*_, at a given temperature, is longer for the Doc with the tails than for the Doc without the tails. We observe this dependence both for the WT Doc and for the Doc with the two-site mutation. The corresponding simulation data are shown in the upper and lower panels of Fig. S6. In addition, the characteristic temperature at which the average fraction of native contacts, *Q*, takes the value $Q=\frac{1}{2}$ is higher for the Doc with the tails than for the Doc without the tails (data not shown). However, another characteristic temperature, *T*_0_, at which *P*_0_ crosses $\frac{1}{2}$ does not distinguish between the systems outside of the error bars (data not shown). Nevertheless, these observations, taken together, are consistent with recent fluorescence experiments on the ScaA dockerin from *R. flavefaciens* indicating that interactions between the N- and C-terminal tails in Doc have a significant influence on thermal stability^43^.

The differences in the thermal stability can be explained, to some extent, in terms of the contact map shown in Fig. S3. The Doc in PDB:1OHZ has 137 native contacts, according to the overlap criterion that underlies the coarse-grained simulations. By building the terminal tails into the dockerin structure, the number of native contacts is increased by 20 to 23, depending on the procedure used to generate the full-length structure (20 contacts for iTaser with the 1OHZ template, 23 contacts for iTaser with the 4DH2 template, and 22 for Swiss modeller with the 4DH2 template). Similarly, the mutated Doc in PDB:2CCL has 134 native contacts and including the tails adds between 29 and 30 new contacts (30 contacts for iTaser with the 2CCL template, 29 contacts for iTaser with the 4DH2 template, and 30 for Swiss modeller with the 4DH2 template). In both cases, the increased number of the native contacts results in the enhanced thermal stability. These new contacts are not related to the presence of the Ca^2+^ ions.

### Stretching of the Coh-Doc complex with tails

**Figure 6:**
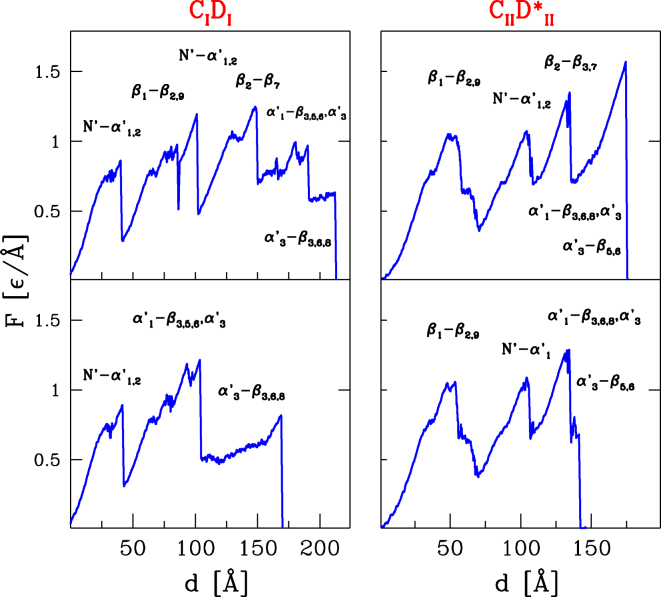
Force-displacement curves for the WT system *C*_*I*_*D*_*I*_ with *k* = 9 (left panels) and for the mutated system ${C}_{II}{D}_{II}^{\ast }$ with *k* = 19 (right panels). The upper panels show typical long trajectories. The lower panels correspond to short trajectories. The symbols indicate the contacts that break at particular displacements. The primed symbols refer to Doc and the unprimed to Coh. N’ is the region near the N-terminus in Doc. For the short trajectory in the lower panel, the contacts ${\alpha }_{1}^{\prime} -{\beta }_{\mathrm{3,6,8}},{\alpha }_{3}^{\prime}$ break both around 130 and 140 Å, i.e., at the two last force peaks.

We follow the procedure and notation as described in ref.^40^ except that now we consider Docs with the tails. We illustrate our findings only for *C*_*I*_*D*_*I*_ (*k* = 9) and ${C}_{II}{D}_{II}^{\ast }$ (*k* = 16). Examples of the force-displacement (*F*–*d*) curves are shown in Fig. 6, the left and right panels respectively.

The figures also show displacements at which particular types of contacts are breaking. For instance, ${\beta }_{1}-{\beta }_{\mathrm{2,9}}$ indicates contacts between ${\beta }_{1}$–${\beta }_{2}$ and ${\beta }_{1}$–${\beta }_{9}$ in Coh, whereas $N^{\prime} -{\alpha }_{1,2}^{\prime}$ means contacts between the N-terminal region on Doc and the first two helices in Doc. The former contacts break first in ${C}_{II}{D}_{II}^{\ast }$ and the latter in *C*_*I*_*D*_*I*_.

The interesting observation is that for both binding modes, there are short and long trajectories. For *C*_*I*_*D*_*I*_, about 66% of the trajectories (100 trajectories were considered) are of the long type and 34% of the short type. The sequence of the unravelling events in the short trajectory is like in the short trajectory for PDB:1OHZ. In the long trajectory, it is similar to the sequence in the dominant trajectory for PDB:1OHZ (the middle panel in Fig. 4 in ref.^40^).

For ${C}_{II}{D}_{II}^{\ast }$ most of the trajectories are short. Only 3% were long, but for the corresponding system without the tails, we have not observed any long trajectories. Both kinds of the trajectories start with unravelling of ${\beta }_{1}-{\beta }_{\mathrm{2,9}}$, whereas for PDB:2CCL, i.e. without the tails, they do with unravelling of $N^{\prime} -{\alpha }_{1}^{\prime}$ (as shown in ref.^40^). Whenever a trajectory for the system with the tails has a corresponding (long or short) trajectory for the system without the tails, the force peaks are found to be identical within the thermal noise of of 0.1 ε/Å. However the locations of the peaks and of the points of dissociation are often shifted. For instance, long trajectories in C_*I*_D_*I*_ result in dissociation around 215 Å but in C_*I*_d_*I*_–around 180 Å.

## Conclusions

Our computational studies show that the full-length WT Coh-Doc complex exhibits dual binding at room temperature, and the ability to bind in the two modes persists at temperatures elevated by 10 K. At the same time, our MD simulations indicate that each mode of binding leads to two kinds of dissociation pathways in AFM-like stretching trajectories. In our opinion, experimental tests of dual binding require other kinds of spectroscopy experiments. In addition, it would be interesting to find other examples of protein complexes with dual binding. Our MD simulations show also that the full-length Doc is thermally more stable than the truncated Doc missing the terminal tails. This result is fully consistent with recent fluorescence experiments^43^. Taken together, our computational studies provide a detailed analysis of the Coh-Doc energy landscape and of the role of the short, terminal segments in the Doc module.

## Electronic supplementary material

Supplementary Information

20_struc_Coh-Doc.doc
