# Supplementary material for: Dual binding in cohesin-dockerin complexes: the energy landscape and the role of short, terminal segments of the dockerin module
Source: Sci Rep. 2018 Mar 22;8:5051. doi: 10.1038/s41598-018-23380-9 (PMC5864761; doi:10.1038/s41598-018-23380-9)
Supplement: Supplementary file 1 — Supplementary Information [file 41598_2018_23380_MOESM1_ESM.pdf]

Dual binding in cohesin-dockerin complexes: the energy landscape and of the role of short, terminal segments of the dockerin module

Michał Wojciechowski,[†] Bartosz Różycki,[†] Pham Dinh Quoc Huy,[†] Mai Suan Li,[†] Edward A. Bayer,[¶] and Marek Cieplak[*,†]

Institute of Physics, Polish Academy of Sciences, Al. Lotników 32/46, PL-02668 Warsaw, Poland, Institute for Computational Sciences and Technology, SBI building, Quang Trung Software city, Tan Chanh Hiep Ward, District 12, Ho Chi Minh City, Vietnam, and Department of Biomolecular Sciences, The Weizmann Institute of Science, 234 Herzl Street, Rehovot 7610001, Israel

E-mail: mc@ifpan.edu.pl

SUPPLEMENTARY INFORMATION

FoldX employs an energy function of the following form:

$$\begin{aligned}\Delta G = a\cdot\Delta G_{\mathrm{vdw}} + b\cdot\Delta G_{\mathrm{solvH}} + c\cdot\Delta G_{\mathrm{solvP}} \\ + d\cdot\Delta G_{\mathrm{wb}} + e\cdot\Delta G_{\mathrm{hbond}} + f\cdot\Delta G_{\mathrm{el}} + g\cdot\Delta G_{\mathrm{kon}} \\ + h\cdot T\Delta S_{\mathrm{mc}} + k\cdot T\Delta S_{\mathrm{sc}} + l\cdot\Delta G_{\mathrm{clash}}\end{aligned}$$

[*]To whom correspondence should be addressed

[†]Institute of Physics, Polish Academy of Sciences, Al. Lotników 32/46, PL-02668 Warsaw, Poland

[‡]Institute for Computational Sciences and Technology, SBI building, Quang Trung Software city, Tan Chanh Hiep Ward, District 12, Ho Chi Minh City, Vietnam

[¶]Department of Biomolecular Sciences, The Weizmann Institute of Science, 234 Herzl Street, Rehovot 7610001, Israel

Here the parameters $a \dots l$ are server-provided relative weights of the different free energy terms. ΔG_{vdw} is a free energy contribution resulting from the van der Waals interactions. ΔG_{solvH} and ΔG_{solvP} are solvation energy terms for apolar and polar groups, respectively. ΔG_{wb} takes into account water bridges. It is an extra stabilizing free-energy term resulting from a water molecule making more than one hydrogen bond with the protein. ΔG_{hbond} is the free energy needed to form a hydrogen bond. ΔG_{el} is the electrostatic contribution of charged groups. ΔG_{kon} is an additional electrostatic contribution for atoms belonging to different polypeptide chains. ΔS_{mc} and ΔS_{sc} are entropy costs related to fixing, respectively, the backbone and the side-chain atoms in a given conformation. Finally, ΔG_{clash} takes into account steric overlaps, either in a soft or hard manner, depending on the task. We use the hard description since our goal is to predict a conformation and determine its free energy.

We use the following options in the FoldX calculations: `command=RepairPDB`, `pdb=template.pdb` and `repair_Interface=ALL`. The option `command=RepairPDB` means that all detected deficiencies in the provided structure are repaired, and `repair_Interface=ALL` means that the geometry of all side-chains in both Coh and Doc – including those at the Coh-Doc interface – are optimized energetically. As a result, for a given Coh-Doc separation Z and rotation angle φ, the backbone atoms are kept at fixed positions, but all of the side-chain atoms are adjusted to minimize ΔG.

Table S1: Free energy of binding between Coh and Doc in mode I calculated by the MM/PBSA method for the WT complex without the tails in Doc, i.e, for the Coh-Doc system with index k=1. All energy values are given in kcal/mol. Here, five MD trajectories are considered.

1OHZ							
	Traj.	ΔE_{ele}	ΔE_{vdW}	ΔG_{PB}	ΔG_{SUR}	-TΔS	ΔG_{bind}
$k=1$	1	-792.65	-75.19	789.77	-8.50	47.06	-39.51
	2	-771.63	-72.93	770.84	-8.36	50.36	-31.72
	3	-790.98	-74.86	786.91	-8.51	50.09	-37.35
	4	-775.38	-73.27	774.08	-8.45	45.55	-37.47
	5	-783.04	-74.30	783.22	-8.35	46.82	-35.66
Average		-782.74	-74.11	780.96	-8.43	47.98	**-36.3 ± 2.9**

Table S2: Binding free energy between Coh and Doc in mode II calculated by the MM/PBSA method for the mutated complex without the tails in Doc, i.e, for the Coh-Doc system with index k=8. All energy values are given in kcal/mol.

2CCL							
	Traj.	ΔE_{ele}	ΔE_{vdW}	ΔG_{PB}	ΔG_{SUR}	-TΔS	ΔG_{bind}
$k=8$	1	-740.58	-68.38	742.22	-7.57	41.51	-32.80
	2	-779.41	-71.07	774.66	-7.75	45.83	-37.74
	3	-821.05	-72.88	813.09	-7.89	51.71	-37.03
	4	-745.92	-68.26	746.22	-7.62	44.63	-30.94
	5	-797.35	-70.47	793.37	-7.78	48.10	-34.12
Average		-776.86	-70.21	773.91	-7.72	46.36	**-34.5 $\pm$ 2.9**

Table S3: Free energy of binding between Coh and Doc in mode I calculated by the MM/PBSA method for the WT complex with the tails in Doc. All energy values are given in kcal/mol. For each of the systems under study, k=9, 11 and 12, five MD trajectories are considered.

$C_I D_I$								
	Traj.	ΔE_{ele}	ΔE_{vdW}	ΔG_{PB}	ΔG_{SUR}	-TΔS	ΔG_{bind}	Average
$k=9$	1	-862.26	-78.53	869.91	-8.72	49.60	-30.01	-40.3 $\pm$ 10.5
	2	-981.65	-95.90	985.50	-10.49	49.11	-53.43	
	3	-1007.09	-86.56	1005.79	-10.38	55.08	-43.16	
	4	-914.78	-74.78	917.94	-8.46	50.92	-29.15	
	5	-1061.30	-85.01	1053.68	-9.81	56.82	-45.62	
$k=12$	1	-918.53	-78.60	916.22	-9.02	52.48	-37.46	-38.8 $\pm$ 5.8
	2	-914.19	-78.67	907.77	-9.07	46.85	-47.33	
	3	-918.03	-76.82	915.85	-8.81	48.47	-39.34	
	4	-893.23	-77.90	898.40	-8.98	50.66	-31.05	
	5	-934.98	-78.11	932.96	-9.04	50.24	-38.93	
$k=11$	1	-895.68	-74.16	892.84	-8.64	49.76	-35.88	-39.1 $\pm$ 6.0
	2	-1020.81	-84.16	1015.17	-9.89	61.99	-37.71	
	3	-904.25	-77.66	910.62	-8.61	46.49	-33.41	
	4	-897.52	-76.25	897.10	-8.60	36.02	-49.24	
	5	-939.51	-77.94	940.13	-8.74	46.75	-39.30	
Average		-937.59	-80.07	937.33	-9.15	50.08	**-39.4 $\pm$ 7.2**	

Table S4: Binding free energy between Coh and Doc in mode II calculated by the MM/PBSA method for the mutated complex with the tails in Doc. All terms are in kcal/mol. For each system, five trajectories are considered.

$C_{II}D^*_{II}$								
	Traj.	ΔE_{ele}	ΔE_{vdW}	ΔG_{PB}	ΔG_{SUR}	-TΔS	ΔG_{bind}	Average
$k = 16$	1	-947.70	-80.58	947.28	-8.96	53.13	-36.83	-41.1 $\pm$ 8.0
	2	-1015.88	-85.53	1012.95	-9.06	54.09	-43.44	
	3	-937.59	-81.34	944.28	-8.84	54.17	-29.33	
	4	-946.05	-80.89	944.32	-8.79	44.54	-46.87	
	5	-997.06	-82.17	986.87	-9.28	52.60	-49.05	
$k = 20$	1	-952.18	-83.62	954.51	-9.04	43.96	-46.37	-46.7 $\pm$ 4.2
	2	-967.47	-81.58	969.40	-9.08	44.78	-43.95	
	3	-992.11	-88.26	987.72	-9.49	48.70	-53.43	
	4	-933.18	-79.40	932.21	-8.93	46.87	-42.43	
	5	-986.68	-83.69	980.79	-9.13	51.59	-47.11	
$k = 19$	1	-853.65	-83.88	853.58	-8.83	47.88	-44.90	-43.1 $\pm$ 4.7
	2	-881.99	-85.39	882.98	-9.00	56.05	-37.34	
	3	-882.76	-83.65	874.78	-8.98	50.84	-49.77	
	4	-870.37	-80.89	871.43	-8.58	47.71	-40.69	
	5	-838.02	-81.67	837.20	-8.64	48.31	-42.83	
Average		-933.51	-82.84	932.02	-8.98	49.68	**-43.6 $\pm$ 6.0**	

Table S5: Free energy of binding between Coh and Doc in mode II calculated by the MM/PBSA method for the WT complex with the tails in Doc. All energy values are given in kcal/mol. For each of the systems under study, with k=10 and k=14, five MD trajectories are considered.

$C_{II}D_{II}$								
	Traj.	ΔE_{ele}	ΔE_{vdW}	ΔG_{PB}	ΔG_{SUR}	-TΔS	ΔG_{bind}	Average
k10	1	-996.9778	-87.0467	993.9438	-9.2281	55.3428	-43.9661	-47.4 $\pm$ 5.3
	2	-951.3534	-80.9987	947.7732	-9.0211	52.8134	-40.7866	
	3	-980.3927	-84.5025	974.8368	-9.1752	53.2084	-46.0252	
	4	-984.5379	-83.2031	977.4924	-9.0981	49.3528	-49.9938	
	5	-1011.6737	-84.1867	1002.7876	-9.0182	46.0849	-56.0061	
k14	1	-997.6491	-87.7408	989.3127	-9.3319	50.2742	-55.1349	-43.6 $\pm$ 8.4
	2	-906.0494	-80.3925	911.8900	-8.8419	48.8036	-34.5901	
	3	-941.7650	-83.2262	944.9474	-8.8905	51.4579	-37.4765	
	4	-947.1659	-79.7071	949.1868	-8.8474	48.2902	-38.2435	
	5	-957.8630	-84.5068	951.8417	-9.1685	47.3842	-52.3125	
Average		-1063.5110	-78.5440	1076.6112	-9.0966	49.1149	**-45.5 $\pm$ 7.3**	

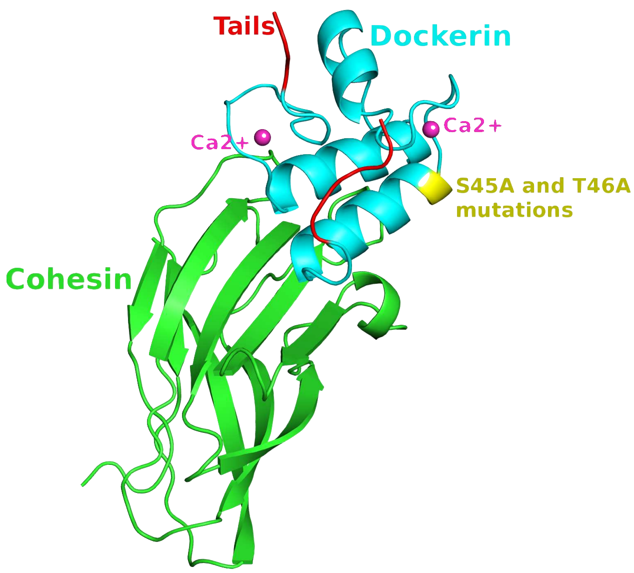

Figure S1: An example of the Coh-Doc structure used for MD simulations. This structure corresponds to the binding mode I. The color code is the following: the Coh is shown in green, the Doc – in cyan, the N- and C-terminal tails of the Doc – in red, the Doc-bound Ca^{2+} ions – in magenta, and the two-site mutation in the Doc is marked in yellow.

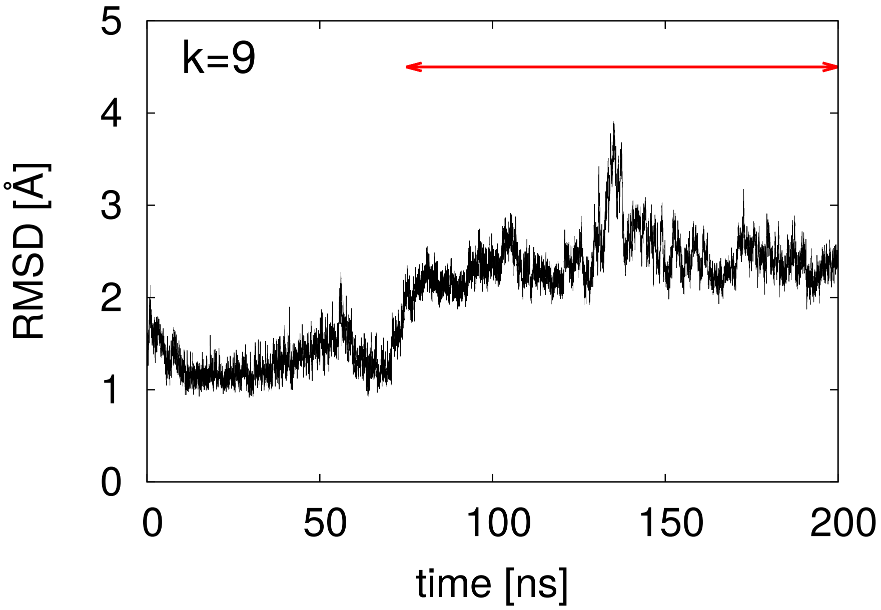

Figure S2: Root-mean-square distance (RMSD) from the native structure as a function of time. The data correspond to one of the MD trajectories of the Coh-Doc system with index k=9, i.e., with the WT Doc with the tails. The red arrow indicates the equilibrated phase of the MD trajectory, which is used for the subsequent MM/PBSA calculations of the binding free energy, ΔG_{bind}. Within the indicated part of the trajectory, the computed values of ΔG_{bind} are scattered around a single minimum which corresponds to the binding mode I. Typical fluctuations in ΔG_{bind} around this minimum are of the order of 10 kcal/mol. They are thus comparable to the statistical error on the trajectory-averaged ΔG_{bind}, as specified in tables S1–S4.

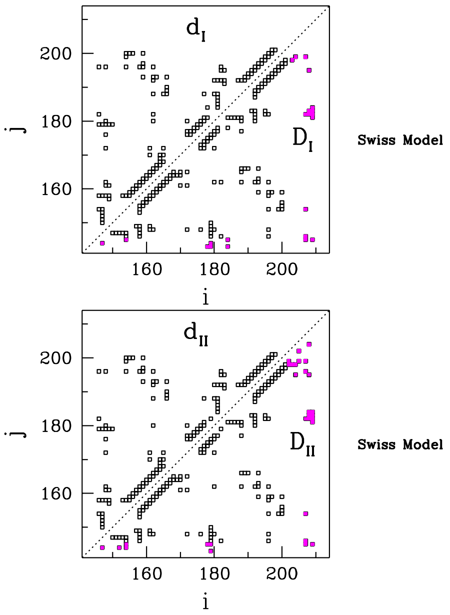

Figure S3: The contact map of the Doc modules. The top panel is for Doc from PDB:1OHZ: above the diagonal is the contact map corresponding to the structure file and below the diagonal – to the structure with the tails, as predicted by the Swiss Model. The symbols in magenta correspond to contacts with and within the tails. The bottom panel is similar but for Doc coming from PDB:2CCL.

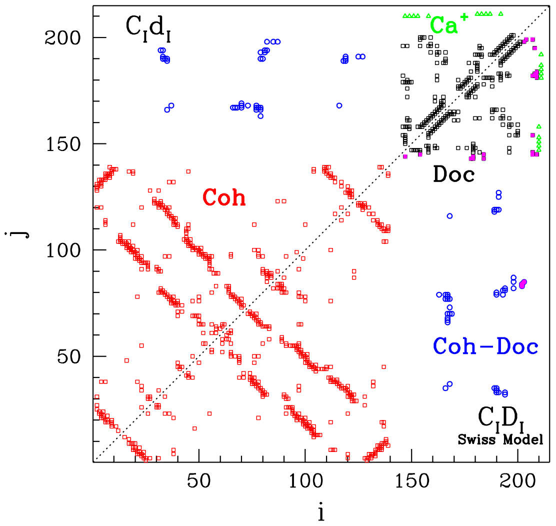

Figure S4: Similar to Fig. S3 but for the whole complex corresponding to PDB:1OHZ (above the diagonal) and PDB:1OHZ with tails (below the diagonal). The red symbols are for contacs in Coh, the blue symbols are for the interfacial contacts, i.e. for those occuring between Coh and Doc. The green symbols represent contacts with the Ca^{+} ions. The symbols in magenta correspond to contacts with and within the tails.

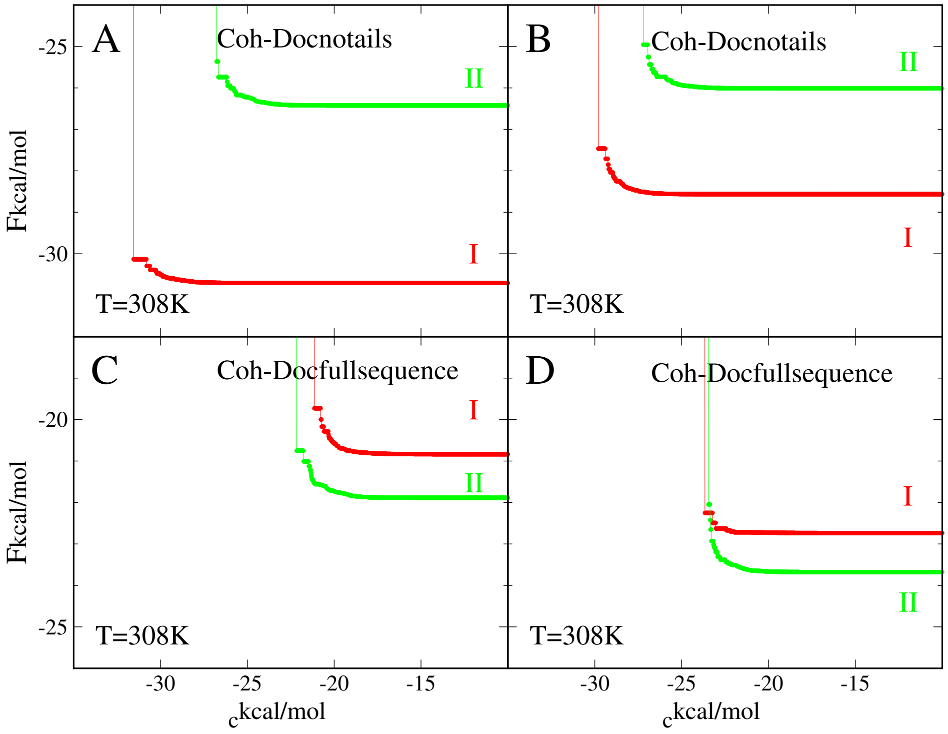

Figure S5: Analogous to Fig. 4 but for T=308 K, i.e., 10 K above the room temperature.

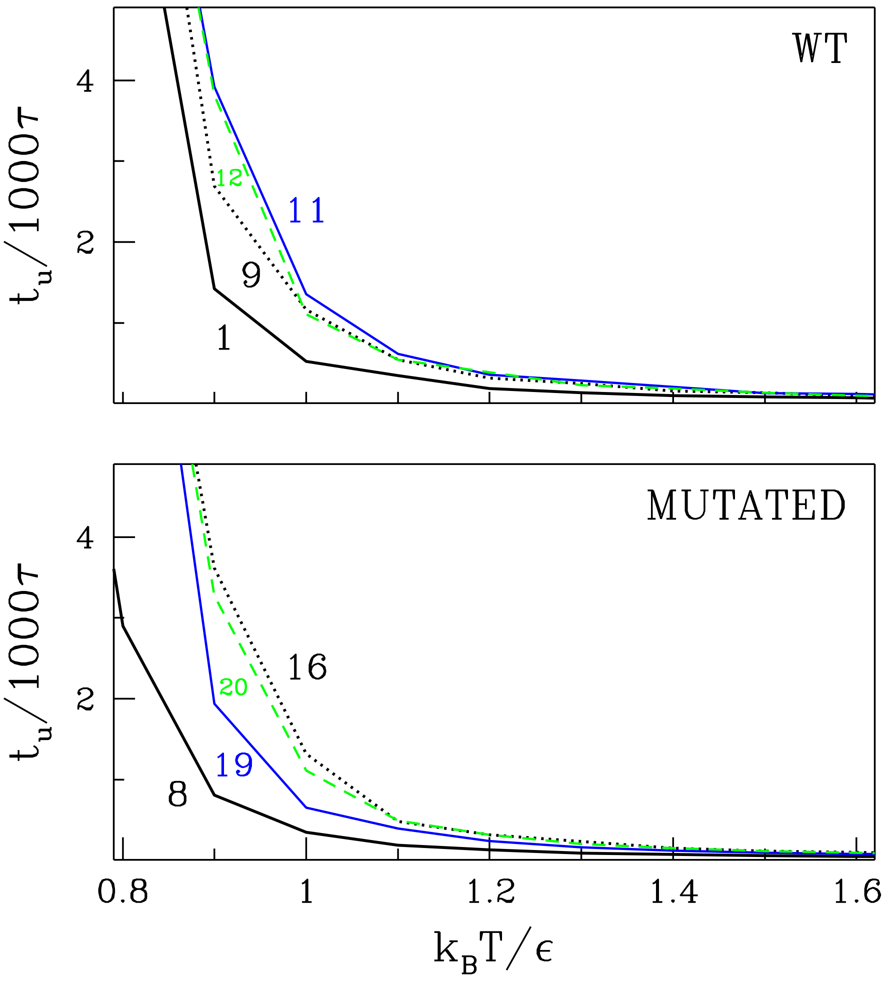

Figure S6: The median time of thermal unfolding, t_u, as a function of temperature T for the wild-type Doc (upper panel) and for the Doc with the two-site mutation (lower panel). The values of index k, which specifies the simulated structres, are given in the figure panels: k=9, 11 and 12 correspond to the WT Docs with the tails, k=1 corresponds to the WT Doc without the tails, as in the PDB:1OHZ, k=16, 19 and 20 correspond to the WT Docs with the tails, and k=8 corresponds to the mutated Doc without the tails, as in the PDB:2CCL.
