# Supplementary material for: Dual binding in cohesin-dockerin complexes: the energy landscape and the role of short, terminal segments of the dockerin module
Source: Sci Rep. 2018 Mar 22;8:5051. doi: 10.1038/s41598-018-23380-9 (PMC5864761; doi:10.1038/s41598-018-23380-9)
Supplement: Supplementary file 2 — 20_struc_Coh-Doc.doc [file 41598_2018_23380_MOESM2_ESM.doc]

MODEL 1

ATOM 0 N GLY A 5 -26.382 0.617 3.011 1.00 1.00

ATOM 1 CA GLY A 5 -24.952 0.189 3.012 1.00 1.00

ATOM 2 C GLY A 5 -24.188 0.716 4.211 1.00 1.00

ATOM 3 O GLY A 5 -24.665 0.631 5.346 1.00 1.00

ATOM 4 N VAL A 6 -23.001 1.261 3.955 1.00 1.00

ATOM 5 CA VAL A 6 -22.150 1.819 5.004 1.00 1.00

ATOM 6 C VAL A 6 -22.351 3.331 5.108 1.00 1.00

ATOM 7 O VAL A 6 -22.383 4.033 4.095 1.00 1.00

ATOM 8 CB VAL A 6 -20.651 1.494 4.765 1.00 1.00

ATOM 9 CG1 VAL A 6 -19.804 1.881 5.975 1.00 1.00

ATOM 10 CG2 VAL A 6 -20.459 0.014 4.442 1.00 1.00

ATOM 11 N VAL A 7 -22.501 3.818 6.339 1.00 1.00

ATOM 12 CA VAL A 7 -22.658 5.247 6.598 1.00 1.00

ATOM 13 C VAL A 7 -21.448 5.781 7.362 1.00 1.00

ATOM 14 O VAL A 7 -21.121 5.296 8.449 1.00 1.00

ATOM 15 CB VAL A 7 -23.965 5.555 7.384 1.00 1.00

ATOM 16 CG1 VAL A 7 -24.147 7.056 7.568 1.00 1.00

ATOM 17 CG2 VAL A 7 -25.181 4.957 6.678 1.00 1.00

ATOM 18 N VAL A 8 -20.779 6.771 6.778 1.00 1.00

ATOM 19 CA VAL A 8 -19.646 7.428 7.427 1.00 1.00

ATOM 20 C VAL A 8 -20.111 8.746 8.048 1.00 1.00

ATOM 21 O VAL A 8 -20.573 9.648 7.346 1.00 1.00

ATOM 22 CB VAL A 8 -18.471 7.672 6.448 1.00 1.00

ATOM 23 CG1 VAL A 8 -17.296 8.328 7.164 1.00 1.00

ATOM 24 CG2 VAL A 8 -18.031 6.365 5.795 1.00 1.00

ATOM 25 N GLU A 9 -19.990 8.840 9.370 1.00 1.00

ATOM 26 CA GLU A 9 -20.486 9.995 10.110 1.00 1.00

ATOM 27 C GLU A 9 -19.352 10.862 10.640 1.00 1.00

ATOM 28 O GLU A 9 -18.575 10.427 11.494 1.00 1.00

ATOM 29 CB GLU A 9 -21.376 9.544 11.273 1.00 1.00

ATOM 30 CG GLU A 9 -22.802 9.193 10.880 1.00 1.00

ATOM 31 CD GLU A 9 -23.518 8.343 11.918 1.00 1.00

ATOM 32 OE1 GLU A 9 -22.960 8.120 13.016 1.00 1.00

ATOM 33 OE2 GLU A 9 -24.648 7.891 11.632 1.00 1.00

ATOM 34 N ILE A 10 -19.257 12.084 10.125 1.00 1.00

ATOM 35 CA ILE A 10 -18.360 13.082 10.695 1.00 1.00

ATOM 36 C ILE A 10 -19.049 13.666 11.923 1.00 1.00

ATOM 37 O ILE A 10 -20.124 14.261 11.818 1.00 1.00

ATOM 38 CB ILE A 10 -18.010 14.191 9.670 1.00 1.00

ATOM 39 CG1 ILE A 10 -17.315 13.592 8.442 1.00 1.00

ATOM 40 CG2 ILE A 10 -17.127 15.263 10.315 1.00 1.00

ATOM 41 CD1 ILE A 10 -17.570 14.348 7.147 1.00 1.00

ATOM 42 N GLY A 11 -18.427 13.472 13.085 1.00 1.00

ATOM 43 CA GLY A 11 -18.997 13.888 14.355 1.00 1.00

ATOM 44 C GLY A 11 -19.236 15.381 14.481 1.00 1.00

ATOM 45 O GLY A 11 -18.627 16.184 13.768 1.00 1.00

ATOM 46 N LYS A 12 -20.137 15.742 15.390 1.00 1.00

ATOM 47 CA LYS A 12 -20.441 17.138 15.681 1.00 1.00

ATOM 48 C LYS A 12 -20.135 17.435 17.146 1.00 1.00

ATOM 49 O LYS A 12 -20.752 16.861 18.045 1.00 1.00

ATOM 50 CB LYS A 12 -21.905 17.452 15.363 1.00 1.00

ATOM 51 CG LYS A 12 -22.193 17.629 13.884 1.00 1.00

ATOM 52 CD LYS A 12 -23.679 17.769 13.617 1.00 1.00

ATOM 53 CE LYS A 12 -24.036 17.288 12.221 1.00 1.00

ATOM 54 NZ LYS A 12 -25.219 18.006 11.666 1.00 1.00

ATOM 55 N VAL A 13 -19.169 18.321 17.369 1.00 1.00

ATOM 56 CA VAL A 13 -18.741 18.690 18.719 1.00 1.00

ATOM 57 C VAL A 13 -18.770 20.206 18.923 1.00 1.00

ATOM 58 O VAL A 13 -18.858 20.970 17.958 1.00 1.00

ATOM 59 CB VAL A 13 -17.322 18.141 19.059 1.00 1.00

ATOM 60 CG1 VAL A 13 -17.336 16.620 19.168 1.00 1.00

ATOM 61 CG2 VAL A 13 -16.283 18.610 18.037 1.00 1.00

ATOM 62 N THR A 14 -18.711 20.632 20.183 1.00 1.00

ATOM 63 CA THR A 14 -18.613 22.052 20.522 1.00 1.00

ATOM 64 C THR A 14 -17.377 22.309 21.379 1.00 1.00

ATOM 65 O THR A 14 -16.962 21.448 22.158 1.00 1.00

ATOM 66 CB THR A 14 -19.884 22.553 21.251 1.00 1.00

ATOM 67 OG1 THR A 14 -20.230 21.649 22.307 1.00 1.00

ATOM 68 CG2 THR A 14 -21.099 22.514 20.329 1.00 1.00

ATOM 69 N GLY A 15 -16.796 23.496 21.232 1.00 1.00

ATOM 70 CA GLY A 15 -15.604 23.865 21.974 1.00 1.00

ATOM 71 C GLY A 15 -15.295 25.348 21.925 1.00 1.00

ATOM 72 O GLY A 15 -15.709 26.051 20.999 1.00 1.00

ATOM 73 N SER A 16 -14.566 25.819 22.934 1.00 1.00

ATOM 74 CA SER A 16 -14.172 27.220 23.026 1.00 1.00

ATOM 75 C SER A 16 -12.972 27.530 22.134 1.00 1.00

ATOM 76 O SER A 16 -12.187 26.637 21.798 1.00 1.00

ATOM 77 CB SER A 16 -13.848 27.589 24.477 1.00 1.00

ATOM 78 OG SER A 16 -14.997 27.485 25.301 1.00 1.00

ATOM 79 N VAL A 17 -12.843 28.798 21.752 1.00 1.00

ATOM 80 CA VAL A 17 -11.699 29.275 20.979 1.00 1.00

ATOM 81 C VAL A 17 -10.420 29.126 21.807 1.00 1.00

ATOM 82 O VAL A 17 -10.384 29.506 22.980 1.00 1.00

ATOM 83 CB VAL A 17 -11.887 30.751 20.529 1.00 1.00

ATOM 84 CG1 VAL A 17 -10.743 31.207 19.623 1.00 1.00

ATOM 85 CG2 VAL A 17 -13.225 30.939 19.822 1.00 1.00

ATOM 86 N GLY A 18 -9.387 28.551 21.195 1.00 1.00

ATOM 87 CA GLY A 18 -8.115 28.337 21.861 1.00 1.00

ATOM 88 C GLY A 18 -7.946 26.944 22.445 1.00 1.00

ATOM 89 O GLY A 18 -6.852 26.589 22.889 1.00 1.00

ATOM 90 N THR A 19 -9.024 26.163 22.457 1.00 1.00

ATOM 91 CA THR A 19 -8.972 24.788 22.955 1.00 1.00

ATOM 92 C THR A 19 -8.813 23.791 21.809 1.00 1.00

ATOM 93 O THR A 19 -9.215 24.064 20.677 1.00 1.00

ATOM 94 CB THR A 19 -10.228 24.435 23.796 1.00 1.00

ATOM 95 OG1 THR A 19 -11.386 24.399 22.951 1.00 1.00

ATOM 96 CG2 THR A 19 -10.549 25.534 24.807 1.00 1.00

ATOM 97 N THR A 20 -8.199 22.643 22.118 1.00 1.00

ATOM 98 CA THR A 20 -8.110 21.561 21.126 1.00 1.00

ATOM 99 C THR A 20 -9.226 20.537 21.391 1.00 1.00

ATOM 100 O THR A 20 -9.376 20.023 22.507 1.00 1.00

ATOM 101 CB THR A 20 -6.735 20.882 21.230 1.00 1.00

ATOM 102 OG1 THR A 20 -5.700 21.858 21.182 1.00 1.00

ATOM 103 CG2 THR A 20 -6.469 19.888 20.102 1.00 1.00

ATOM 104 N VAL A 21 -9.994 20.261 20.347 1.00 1.00

ATOM 105 CA VAL A 21 -11.122 19.309 20.432 1.00 1.00

ATOM 106 C VAL A 21 -10.900 18.113 19.505 1.00 1.00

ATOM 107 O VAL A 21 -10.069 18.151 18.588 1.00 1.00

ATOM 108 CB VAL A 21 -12.434 19.994 20.031 1.00 1.00

ATOM 109 CG1 VAL A 21 -12.853 21.090 21.014 1.00 1.00

ATOM 110 CG2 VAL A 21 -12.370 20.667 18.655 1.00 1.00

ATOM 111 N GLU A 22 -11.660 17.073 19.780 1.00 1.00

ATOM 112 CA GLU A 22 -11.597 15.833 19.008 1.00 1.00

ATOM 113 C GLU A 22 -12.903 15.602 18.262 1.00 1.00

ATOM 114 O GLU A 22 -13.985 15.572 18.861 1.00 1.00

ATOM 115 CB GLU A 22 -11.336 14.654 19.937 1.00 1.00

ATOM 116 CG GLU A 22 -9.968 14.009 19.761 1.00 1.00

ATOM 117 CD GLU A 22 -9.575 13.137 20.938 1.00 1.00

ATOM 118 OE1 GLU A 22 -10.196 12.069 21.121 1.00 1.00

ATOM 119 OE2 GLU A 22 -8.644 13.522 21.678 1.00 1.00

ATOM 120 N ILE A 23 -12.767 15.448 16.961 1.00 1.00

ATOM 121 CA ILE A 23 -13.914 15.193 16.083 1.00 1.00

ATOM 122 C ILE A 23 -13.847 13.758 15.564 1.00 1.00

ATOM 123 O ILE A 23 -12.950 13.401 14.789 1.00 1.00

ATOM 124 CB ILE A 23 -13.918 16.147 14.886 1.00 1.00

ATOM 125 CG1 ILE A 23 -13.774 17.617 15.290 1.00 1.00

ATOM 126 CG2 ILE A 23 -15.215 16.057 14.073 1.00 1.00

ATOM 127 CD1 ILE A 23 -12.458 18.242 14.820 1.00 1.00

ATOM 128 N PRO A 24 -14.713 12.914 16.117 1.00 1.00

ATOM 129 CA PRO A 24 -14.730 11.494 15.747 1.00 1.00

ATOM 130 C PRO A 24 -15.326 11.272 14.360 1.00 1.00

ATOM 131 O PRO A 24 -16.227 12.002 13.943 1.00 1.00

ATOM 132 CB PRO A 24 -15.617 10.858 16.827 1.00 1.00

ATOM 133 CG PRO A 24 -16.506 11.961 17.304 1.00 1.00

ATOM 134 CD PRO A 24 -15.767 13.249 17.095 1.00 1.00

ATOM 135 N VAL A 25 -14.793 10.279 13.652 1.00 1.00

ATOM 136 CA VAL A 25 -15.353 9.833 12.381 1.00 1.00

ATOM 137 C VAL A 25 -15.895 8.422 12.596 1.00 1.00

ATOM 138 O VAL A 25 -15.135 7.497 12.885 1.00 1.00

ATOM 139 CB VAL A 25 -14.297 9.845 11.244 1.00 1.00

ATOM 140 CG1 VAL A 25 -14.908 9.376 9.929 1.00 1.00

ATOM 141 CG2 VAL A 25 -13.693 11.235 11.080 1.00 1.00

ATOM 142 N TYR A 26 -17.211 8.272 12.466 1.00 1.00

ATOM 143 CA TYR A 26 -17.880 7.006 12.752 1.00 1.00

ATOM 144 C TYR A 26 -18.131 6.175 11.497 1.00 1.00

ATOM 145 O TYR A 26 -18.486 6.711 10.447 1.00 1.00

ATOM 146 CB TYR A 26 -19.218 7.250 13.460 1.00 1.00

ATOM 147 CG TYR A 26 -19.117 7.942 14.802 1.00 1.00

ATOM 148 CD1 TYR A 26 -19.445 9.290 14.937 1.00 1.00

ATOM 149 CD2 TYR A 26 -18.709 7.245 15.942 1.00 1.00

ATOM 150 CE1 TYR A 26 -19.363 9.934 16.170 1.00 1.00

ATOM 151 CE2 TYR A 26 -18.623 7.880 17.180 1.00 1.00

ATOM 152 CZ TYR A 26 -18.952 9.222 17.286 1.00 1.00

ATOM 153 OH TYR A 26 -18.869 9.854 18.508 1.00 1.00

ATOM 154 N PHE A 27 -17.942 4.863 11.623 1.00 1.00

ATOM 155 CA PHE A 27 -18.354 3.914 10.594 1.00 1.00

ATOM 156 C PHE A 27 -19.603 3.184 11.078 1.00 1.00

ATOM 157 O PHE A 27 -19.561 2.457 12.072 1.00 1.00

ATOM 158 CB PHE A 27 -17.235 2.909 10.293 1.00 1.00

ATOM 159 CG PHE A 27 -16.260 3.363 9.231 1.00 1.00

ATOM 160 CD1 PHE A 27 -16.177 4.701 8.847 1.00 1.00

ATOM 161 CD2 PHE A 27 -15.412 2.441 8.624 1.00 1.00

ATOM 162 CE1 PHE A 27 -15.266 5.111 7.873 1.00 1.00

ATOM 163 CE2 PHE A 27 -14.500 2.841 7.647 1.00 1.00

ATOM 164 CZ PHE A 27 -14.428 4.180 7.273 1.00 1.00

ATOM 165 N ARG A 28 -20.715 3.396 10.380 1.00 1.00

ATOM 166 CA ARG A 28 -21.978 2.745 10.717 1.00 1.00

ATOM 167 C ARG A 28 -22.413 1.800 9.602 1.00 1.00

ATOM 168 O ARG A 28 -22.226 2.096 8.421 1.00 1.00

ATOM 169 CB ARG A 28 -23.077 3.777 10.997 1.00 1.00

ATOM 170 CG ARG A 28 -22.707 4.841 12.027 1.00 1.00

ATOM 171 CD ARG A 28 -23.058 4.484 13.463 1.00 1.00

ATOM 172 NE ARG A 28 -22.739 5.572 14.389 1.00 1.00

ATOM 173 CZ ARG A 28 -22.233 5.401 15.608 1.00 1.00

ATOM 174 NH1 ARG A 28 -21.980 6.458 16.372 1.00 1.00

ATOM 175 NH2 ARG A 28 -21.976 4.184 16.070 1.00 1.00

ATOM 176 N GLY A 29 -22.984 0.663 9.992 1.00 1.00

ATOM 177 CA GLY A 29 -23.461 -0.331 9.045 1.00 1.00

ATOM 178 C GLY A 29 -22.346 -1.091 8.348 1.00 1.00

ATOM 179 O GLY A 29 -22.407 -1.318 7.137 1.00 1.00

ATOM 180 N VAL A 30 -21.329 -1.479 9.118 1.00 1.00

ATOM 181 CA VAL A 30 -20.206 -2.268 8.612 1.00 1.00

ATOM 182 C VAL A 30 -20.703 -3.666 8.229 1.00 1.00

ATOM 183 O VAL A 30 -21.301 -4.357 9.059 1.00 1.00

ATOM 184 CB VAL A 30 -19.054 -2.354 9.654 1.00 1.00

ATOM 185 CG1 VAL A 30 -17.937 -3.281 9.179 1.00 1.00

ATOM 186 CG2 VAL A 30 -18.493 -0.968 9.959 1.00 1.00

ATOM 187 N PRO A 31 -20.467 -4.075 6.978 1.00 1.00

ATOM 188 CA PRO A 31 -20.999 -5.343 6.456 1.00 1.00

ATOM 189 C PRO A 31 -20.384 -6.584 7.104 1.00 1.00

ATOM 190 O PRO A 31 -19.391 -6.480 7.830 1.00 1.00

ATOM 191 CB PRO A 31 -20.644 -5.286 4.965 1.00 1.00

ATOM 192 CG PRO A 31 -19.470 -4.378 4.890 1.00 1.00

ATOM 193 CD PRO A 31 -19.678 -3.353 5.964 1.00 1.00

ATOM 194 N SER A 32 -20.949 -7.741 6.756 1.00 1.00

ATOM 195 CA SER A 32 -20.514 -8.969 7.421 1.00 1.00

ATOM 196 C SER A 32 -19.112 -9.385 6.951 1.00 1.00

ATOM 197 O SER A 32 -18.348 -9.998 7.708 1.00 1.00

ATOM 198 CB SER A 32 -21.490 -10.091 7.126 1.00 1.00

ATOM 199 OG SER A 32 -21.773 -10.187 5.735 1.00 1.00

ATOM 200 N LYS A 33 -18.793 -9.042 5.707 1.00 1.00

ATOM 201 CA LYS A 33 -17.474 -9.376 5.118 1.00 1.00

ATOM 202 C LYS A 33 -16.394 -8.386 5.651 1.00 1.00

ATOM 203 O LYS A 33 -15.187 -8.639 5.535 1.00 1.00

ATOM 204 CB LYS A 33 -17.536 -9.322 3.564 1.00 1.00

ATOM 205 CG LYS A 33 -18.515 -10.307 2.974 1.00 1.00

ATOM 206 CD LYS A 33 -18.630 -10.105 1.472 1.00 1.00

ATOM 207 CE LYS A 33 -19.982 -9.522 1.094 1.00 1.00

ATOM 208 NZ LYS A 33 -20.074 -9.233 -0.364 1.00 1.00

ATOM 209 N GLY A 34 -16.862 -7.274 6.229 1.00 1.00

ATOM 210 CA GLY A 34 -15.983 -6.225 6.830 1.00 1.00

ATOM 211 C GLY A 34 -15.553 -5.173 5.798 1.00 1.00

ATOM 212 O GLY A 34 -16.113 -5.079 4.704 1.00 1.00

ATOM 213 N ILE A 35 -14.557 -4.394 6.194 1.00 1.00

ATOM 214 CA ILE A 35 -13.980 -3.345 5.335 1.00 1.00

ATOM 215 C ILE A 35 -12.454 -3.470 5.339 1.00 1.00

ATOM 216 O ILE A 35 -11.798 -3.259 6.371 1.00 1.00

ATOM 217 CB ILE A 35 -14.357 -1.939 5.823 1.00 1.00

ATOM 218 CG1 ILE A 35 -15.885 -1.687 5.838 1.00 1.00

ATOM 219 CG2 ILE A 35 -13.739 -0.834 4.950 1.00 1.00

ATOM 220 CD1 ILE A 35 -16.527 -1.663 4.443 1.00 1.00

ATOM 221 N ALA A 36 -11.917 -3.941 4.220 1.00 1.00

ATOM 222 CA ALA A 36 -10.484 -4.206 4.080 1.00 1.00

ATOM 223 C ALA A 36 -9.691 -2.957 3.695 1.00 1.00

ATOM 224 O ALA A 36 -8.515 -2.829 4.043 1.00 1.00

ATOM 225 CB ALA A 36 -10.249 -5.321 3.066 1.00 1.00

ATOM 226 N ASN A 37 -10.339 -2.048 2.969 1.00 1.00

ATOM 227 CA ASN A 37 -9.706 -0.809 2.520 1.00 1.00

ATOM 228 C ASN A 37 -10.710 0.308 2.257 1.00 1.00

ATOM 229 O ASN A 37 -11.846 0.052 1.846 1.00 1.00

ATOM 230 CB ASN A 37 -8.861 -1.053 1.261 1.00 1.00

ATOM 231 CG ASN A 37 -9.706 -1.385 0.043 1.00 1.00

ATOM 232 OD1 ASN A 37 -10.028 -2.549 -0.207 1.00 1.00

ATOM 233 ND2 ASN A 37 -10.066 -0.364 -0.722 1.00 1.00

ATOM 234 N CYS A 38 -10.277 1.544 2.493 1.00 1.00

ATOM 235 CA CYS A 38 -11.066 2.728 2.164 1.00 1.00

ATOM 236 C CYS A 38 -10.207 3.985 2.094 1.00 1.00

ATOM 237 O CYS A 38 -9.181 4.093 2.773 1.00 1.00

ATOM 238 CB CYS A 38 -12.215 2.926 3.160 1.00 1.00

ATOM 239 SG CYS A 38 -11.700 3.240 4.856 1.00 1.00

ATOM 240 N ASP A 39 -10.624 4.923 1.250 1.00 1.00

ATOM 241 CA ASP A 39 -10.000 6.238 1.174 1.00 1.00

ATOM 242 C ASP A 39 -11.036 7.297 0.825 1.00 1.00

ATOM 243 O ASP A 39 -11.946 7.052 0.029 1.00 1.00

ATOM 244 CB ASP A 39 -8.826 6.252 0.182 1.00 1.00

ATOM 245 CG ASP A 39 -9.276 6.230 -1.266 1.00 1.00

ATOM 246 OD1 ASP A 39 -9.512 5.126 -1.799 1.00 1.00

ATOM 247 OD2 ASP A 39 -9.420 7.264 -1.950 1.00 1.00

ATOM 248 N PHE A 40 -10.903 8.462 1.453 1.00 1.00

ATOM 249 CA PHE A 40 -11.802 9.589 1.214 1.00 1.00

ATOM 250 C PHE A 40 -11.191 10.918 1.651 1.00 1.00

ATOM 251 O PHE A 40 -10.274 10.954 2.476 1.00 1.00

ATOM 252 CB PHE A 40 -13.179 9.380 1.880 1.00 1.00

ATOM 253 CG PHE A 40 -13.119 8.748 3.247 1.00 1.00

ATOM 254 CD1 PHE A 40 -12.876 9.524 4.378 1.00 1.00

ATOM 255 CD2 PHE A 40 -13.335 7.383 3.406 1.00 1.00

ATOM 256 CE1 PHE A 40 -12.830 8.944 5.645 1.00 1.00

ATOM 257 CE2 PHE A 40 -13.291 6.794 4.667 1.00 1.00

ATOM 258 CZ PHE A 40 -13.039 7.578 5.789 1.00 1.00

ATOM 259 N VAL A 41 -11.709 12.000 1.077 1.00 1.00

ATOM 260 CA VAL A 41 -11.228 13.352 1.342 1.00 1.00

ATOM 261 C VAL A 41 -12.326 14.172 2.019 1.00 1.00

ATOM 262 O VAL A 41 -13.487 14.127 1.603 1.00 1.00

ATOM 263 CB VAL A 41 -10.783 14.059 0.029 1.00 1.00

ATOM 264 CG1 VAL A 41 -10.251 15.464 0.306 1.00 1.00

ATOM 265 CG2 VAL A 41 -9.737 13.232 -0.713 1.00 1.00

ATOM 266 N PHE A 42 -11.958 14.902 3.071 1.00 1.00

ATOM 267 CA PHE A 42 -12.865 15.842 3.726 1.00 1.00

ATOM 268 C PHE A 42 -12.389 17.281 3.524 1.00 1.00

ATOM 269 O PHE A 42 -11.186 17.543 3.456 1.00 1.00

ATOM 270 CB PHE A 42 -12.987 15.551 5.231 1.00 1.00

ATOM 271 CG PHE A 42 -13.671 14.243 5.565 1.00 1.00

ATOM 272 CD1 PHE A 42 -13.521 13.685 6.831 1.00 1.00

ATOM 273 CD2 PHE A 42 -14.472 13.579 4.636 1.00 1.00

ATOM 274 CE1 PHE A 42 -14.141 12.482 7.164 1.00 1.00

ATOM 275 CE2 PHE A 42 -15.098 12.376 4.960 1.00 1.00

ATOM 276 CZ PHE A 42 -14.932 11.829 6.229 1.00 1.00

ATOM 277 N ARG A 43 -13.341 18.206 3.434 1.00 1.00

ATOM 278 CA ARG A 43 -13.047 19.631 3.299 1.00 1.00

ATOM 279 C ARG A 43 -13.218 20.345 4.642 1.00 1.00

ATOM 280 O ARG A 43 -14.064 19.959 5.452 1.00 1.00

ATOM 281 CB ARG A 43 -13.968 20.257 2.249 1.00 1.00

ATOM 282 CG ARG A 43 -13.427 21.522 1.602 1.00 1.00

ATOM 283 CD ARG A 43 -13.790 21.673 0.133 1.00 1.00

ATOM 284 NE ARG A 43 -12.635 21.456 -0.735 1.00 1.00

ATOM 285 CZ ARG A 43 -12.701 21.194 -2.036 1.00 1.00

ATOM 286 NH1 ARG A 43 -13.878 21.115 -2.651 1.00 1.00

ATOM 287 NH2 ARG A 43 -11.586 21.011 -2.728 1.00 1.00

ATOM 288 N TYR A 44 -12.407 21.377 4.869 1.00 1.00

ATOM 289 CA TYR A 44 -12.500 22.199 6.080 1.00 1.00

ATOM 290 C TYR A 44 -11.967 23.618 5.866 1.00 1.00

ATOM 291 O TYR A 44 -11.327 23.904 4.852 1.00 1.00

ATOM 292 CB TYR A 44 -11.787 21.519 7.261 1.00 1.00

ATOM 293 CG TYR A 44 -10.279 21.686 7.294 1.00 1.00

ATOM 294 CD1 TYR A 44 -9.459 20.984 6.409 1.00 1.00

ATOM 295 CD2 TYR A 44 -9.673 22.535 8.221 1.00 1.00

ATOM 296 CE1 TYR A 44 -8.073 21.129 6.442 1.00 1.00

ATOM 297 CE2 TYR A 44 -8.290 22.687 8.264 1.00 1.00

ATOM 298 CZ TYR A 44 -7.497 21.981 7.371 1.00 1.00

ATOM 299 OH TYR A 44 -6.130 22.126 7.407 1.00 1.00

ATOM 300 N ASP A 45 -12.243 24.498 6.828 1.00 1.00

ATOM 301 CA ASP A 45 -11.743 25.871 6.809 1.00 1.00

ATOM 302 C ASP A 45 -10.557 26.029 7.763 1.00 1.00

ATOM 303 O ASP A 45 -10.722 25.947 8.984 1.00 1.00

ATOM 304 CB ASP A 45 -12.857 26.859 7.174 1.00 1.00

ATOM 305 CG ASP A 45 -12.555 28.285 6.731 1.00 1.00

ATOM 306 OD1 ASP A 45 -11.368 28.671 6.659 1.00 1.00

ATOM 307 OD2 ASP A 45 -13.456 29.101 6.437 1.00 1.00

ATOM 308 N PRO A 46 -9.367 26.260 7.204 1.00 1.00

ATOM 309 CA PRO A 46 -8.139 26.432 7.996 1.00 1.00

ATOM 310 C PRO A 46 -8.123 27.708 8.841 1.00 1.00

ATOM 311 O PRO A 46 -7.372 27.772 9.816 1.00 1.00

ATOM 312 CB PRO A 46 -7.040 26.496 6.928 1.00 1.00

ATOM 313 CG PRO A 46 -7.668 25.946 5.701 1.00 1.00

ATOM 314 CD PRO A 46 -9.100 26.362 5.759 1.00 1.00

ATOM 315 N ASN A 47 -8.927 28.702 8.465 1.00 1.00

ATOM 316 CA ASN A 47 -9.056 29.937 9.241 1.00 1.00

ATOM 317 C ASN A 47 -9.897 29.745 10.502 1.00 1.00

ATOM 318 O ASN A 47 -9.771 30.506 11.464 1.00 1.00

ATOM 319 CB ASN A 47 -9.648 31.064 8.386 1.00 1.00

ATOM 320 CG ASN A 47 -8.770 31.430 7.202 1.00 1.00

ATOM 321 OD1 ASN A 47 -7.541 31.464 7.303 1.00 1.00

ATOM 322 ND2 ASN A 47 -9.400 31.712 6.067 1.00 1.00

ATOM 323 N VAL A 48 -10.757 28.728 10.484 1.00 1.00

ATOM 324 CA VAL A 48 -11.626 28.414 11.614 1.00 1.00

ATOM 325 C VAL A 48 -11.025 27.306 12.481 1.00 1.00

ATOM 326 O VAL A 48 -11.031 27.401 13.710 1.00 1.00

ATOM 327 CB VAL A 48 -13.050 28.008 11.147 1.00 1.00

ATOM 328 CG1 VAL A 48 -13.966 27.782 12.341 1.00 1.00

ATOM 329 CG2 VAL A 48 -13.641 29.062 10.214 1.00 1.00

ATOM 330 N LEU A 49 -10.504 26.263 11.836 1.00 1.00

ATOM 331 CA LEU A 49 -9.929 25.122 12.545 1.00 1.00

ATOM 332 C LEU A 49 -8.503 24.828 12.102 1.00 1.00

ATOM 333 O LEU A 49 -8.210 24.768 10.907 1.00 1.00

ATOM 334 CB LEU A 49 -10.790 23.867 12.357 1.00 1.00

ATOM 335 CG LEU A 49 -12.254 23.862 12.806 1.00 1.00

ATOM 336 CD1 LEU A 49 -12.935 22.585 12.333 1.00 1.00

ATOM 337 CD2 LEU A 49 -12.386 24.016 14.316 1.00 1.00

ATOM 338 N GLU A 50 -7.618 24.650 13.079 1.00 1.00

ATOM 339 CA GLU A 50 -6.256 24.201 12.811 1.00 1.00

ATOM 340 C GLU A 50 -6.145 22.724 13.176 1.00 1.00

ATOM 341 O GLU A 50 -6.047 22.372 14.353 1.00 1.00

ATOM 342 CB GLU A 50 -5.237 25.040 13.591 1.00 1.00

ATOM 343 CG GLU A 50 -3.784 24.689 13.296 1.00 1.00

ATOM 344 CD GLU A 50 -2.800 25.391 14.213 1.00 1.00

ATOM 345 OE1 GLU A 50 -3.032 25.426 15.441 1.00 1.00

ATOM 346 OE2 GLU A 50 -1.782 25.905 13.702 1.00 1.00

ATOM 347 N ILE A 51 -6.181 21.864 12.160 1.00 1.00

ATOM 348 CA ILE A 51 -6.078 20.423 12.374 1.00 1.00

ATOM 349 C ILE A 51 -4.613 20.035 12.560 1.00 1.00

ATOM 350 O ILE A 51 -3.797 20.174 11.645 1.00 1.00

ATOM 351 CB ILE A 51 -6.746 19.630 11.219 1.00 1.00

ATOM 352 CG1 ILE A 51 -8.253 19.920 11.182 1.00 1.00

ATOM 353 CG2 ILE A 51 -6.485 18.127 11.371 1.00 1.00

ATOM 354 CD1 ILE A 51 -8.964 19.434 9.934 1.00 1.00

ATOM 355 N ILE A 52 -4.296 19.556 13.759 1.00 1.00

ATOM 356 CA ILE A 52 -2.916 19.267 14.149 1.00 1.00

ATOM 357 C ILE A 52 -2.565 17.778 14.086 1.00 1.00

ATOM 358 O ILE A 52 -1.395 17.406 14.206 1.00 1.00

ATOM 359 CB ILE A 52 -2.605 19.849 15.556 1.00 1.00

ATOM 360 CG1 ILE A 52 -3.650 19.402 16.585 1.00 1.00

ATOM 361 CG2 ILE A 52 -2.514 21.374 15.495 1.00 1.00

ATOM 362 CD1 ILE A 52 -3.062 19.002 17.928 1.00 1.00

ATOM 363 N GLY A 53 -3.580 16.935 13.897 1.00 1.00

ATOM 364 CA GLY A 53 -3.370 15.502 13.798 1.00 1.00

ATOM 365 C GLY A 53 -4.637 14.686 13.632 1.00 1.00

ATOM 366 O GLY A 53 -5.736 15.142 13.963 1.00 1.00

ATOM 367 N ILE A 54 -4.476 13.476 13.098 1.00 1.00

ATOM 368 CA ILE A 54 -5.569 12.513 12.973 1.00 1.00

ATOM 369 C ILE A 54 -5.101 11.145 13.475 1.00 1.00

ATOM 370 O ILE A 54 -4.070 10.632 13.033 1.00 1.00

ATOM 371 CB ILE A 54 -6.084 12.413 11.509 1.00 1.00

ATOM 372 CG1 ILE A 54 -6.525 13.783 10.978 1.00 1.00

ATOM 373 CG2 ILE A 54 -7.240 11.420 11.413 1.00 1.00

ATOM 374 CD1 ILE A 54 -6.438 13.927 9.466 1.00 1.00

ATOM 375 N ASP A 55 -5.866 10.570 14.399 1.00 1.00

ATOM 376 CA ASP A 55 -5.539 9.276 14.991 1.00 1.00

ATOM 377 C ASP A 55 -6.551 8.209 14.580 1.00 1.00

ATOM 378 O ASP A 55 -7.730 8.517 14.407 1.00 1.00

ATOM 379 CB ASP A 55 -5.497 9.382 16.519 1.00 1.00

ATOM 380 CG ASP A 55 -4.502 10.415 17.010 1.00 1.00

ATOM 381 OD1 ASP A 55 -3.298 10.290 16.699 1.00 1.00

ATOM 382 OD2 ASP A 55 -4.839 11.387 17.718 1.00 1.00

ATOM 383 N PRO A 56 -6.098 6.965 14.416 1.00 1.00

ATOM 384 CA PRO A 56 -7.004 5.843 14.134 1.00 1.00

ATOM 385 C PRO A 56 -7.872 5.490 15.342 1.00 1.00

ATOM 386 O PRO A 56 -7.402 5.562 16.480 1.00 1.00

ATOM 387 CB PRO A 56 -6.048 4.689 13.815 1.00 1.00

ATOM 388 CG PRO A 56 -4.782 5.037 14.515 1.00 1.00

ATOM 389 CD PRO A 56 -4.688 6.533 14.466 1.00 1.00

ATOM 390 N GLY A 57 -9.123 5.120 15.086 1.00 1.00

ATOM 391 CA GLY A 57 -10.039 4.711 16.137 1.00 1.00

ATOM 392 C GLY A 57 -9.835 3.269 16.562 1.00 1.00

ATOM 393 O GLY A 57 -8.948 2.582 16.049 1.00 1.00

ATOM 394 N ASP A 58 -10.667 2.808 17.495 1.00 1.00

ATOM 395 CA ASP A 58 -10.531 1.463 18.059 1.00 1.00

ATOM 396 C ASP A 58 -10.989 0.336 17.127 1.00 1.00

ATOM 397 O ASP A 58 -10.685 -0.834 17.377 1.00 1.00

ATOM 398 CB ASP A 58 -11.233 1.353 19.425 1.00 1.00

ATOM 399 CG ASP A 58 -12.575 2.064 19.464 1.00 1.00

ATOM 400 OD1 ASP A 58 -13.460 1.738 18.643 1.00 1.00

ATOM 401 OD2 ASP A 58 -12.837 2.960 20.296 1.00 1.00

ATOM 402 N ILE A 59 -11.708 0.683 16.062 1.00 1.00

ATOM 403 CA ILE A 59 -12.164 -0.316 15.086 1.00 1.00

ATOM 404 C ILE A 59 -11.050 -0.760 14.128 1.00 1.00

ATOM 405 O ILE A 59 -11.219 -1.716 13.368 1.00 1.00

ATOM 406 CB ILE A 59 -13.439 0.153 14.320 1.00 1.00

ATOM 407 CG1 ILE A 59 -13.137 1.350 13.407 1.00 1.00

ATOM 408 CG2 ILE A 59 -14.582 0.440 15.303 1.00 1.00

ATOM 409 CD1 ILE A 59 -14.219 1.644 12.382 1.00 1.00

ATOM 410 N ILE A 60 -9.917 -0.060 14.176 1.00 1.00

ATOM 411 CA ILE A 60 -8.702 -0.509 13.505 1.00 1.00

ATOM 412 C ILE A 60 -7.897 -1.346 14.499 1.00 1.00

ATOM 413 O ILE A 60 -7.414 -0.831 15.513 1.00 1.00

ATOM 414 CB ILE A 60 -7.894 0.692 12.943 1.00 1.00

ATOM 415 CG1 ILE A 60 -8.543 1.200 11.651 1.00 1.00

ATOM 416 CG2 ILE A 60 -6.437 0.310 12.669 1.00 1.00

ATOM 417 CD1 ILE A 60 -8.824 2.682 11.648 1.00 1.00

ATOM 418 N VAL A 61 -7.776 -2.638 14.206 1.00 1.00

ATOM 419 CA VAL A 61 -7.240 -3.611 15.164 1.00 1.00

ATOM 420 C VAL A 61 -5.775 -3.994 14.933 1.00 1.00

ATOM 421 O VAL A 61 -5.186 -4.716 15.743 1.00 1.00

ATOM 422 CB VAL A 61 -8.123 -4.892 15.247 1.00 1.00

ATOM 423 CG1 VAL A 61 -9.499 -4.565 15.820 1.00 1.00

ATOM 424 CG2 VAL A 61 -8.246 -5.574 13.883 1.00 1.00

ATOM 425 N ASP A 62 -5.196 -3.512 13.832 1.00 1.00

ATOM 426 CA ASP A 62 -3.781 -3.724 13.526 1.00 1.00

ATOM 427 C ASP A 62 -2.908 -3.288 14.711 1.00 1.00

ATOM 428 O ASP A 62 -2.964 -2.126 15.124 1.00 1.00

ATOM 429 CB ASP A 62 -3.388 -2.944 12.260 1.00 1.00

ATOM 430 CG ASP A 62 -2.070 -3.411 11.650 1.00 1.00

ATOM 431 OD1 ASP A 62 -1.118 -3.729 12.396 1.00 1.00

ATOM 432 OD2 ASP A 62 -1.883 -3.479 10.417 1.00 1.00

ATOM 433 N PRO A 63 -2.128 -4.224 15.264 1.00 1.00

ATOM 434 CA PRO A 63 -1.211 -3.931 16.378 1.00 1.00

ATOM 435 C PRO A 63 -0.143 -2.897 16.012 1.00 1.00

ATOM 436 O PRO A 63 0.400 -2.232 16.899 1.00 1.00

ATOM 437 CB PRO A 63 -0.558 -5.289 16.664 1.00 1.00

ATOM 438 CG PRO A 63 -0.753 -6.083 15.418 1.00 1.00

ATOM 439 CD PRO A 63 -2.073 -5.644 14.871 1.00 1.00

ATOM 440 N ASN A 64 0.151 -2.787 14.717 1.00 1.00

ATOM 441 CA ASN A 64 0.983 -1.721 14.174 1.00 1.00

ATOM 442 C ASN A 64 0.116 -0.846 13.259 1.00 1.00

ATOM 443 O ASN A 64 0.188 -0.969 12.034 1.00 1.00

ATOM 444 CB ASN A 64 2.156 -2.315 13.388 1.00 1.00

ATOM 445 CG ASN A 64 3.499 -1.746 13.809 1.00 1.00

ATOM 446 OD1 ASN A 64 4.408 -2.490 14.180 1.00 1.00

ATOM 447 ND2 ASN A 64 3.637 -0.423 13.744 1.00 1.00

ATOM 448 N PRO A 65 -0.703 0.030 13.850 1.00 1.00

ATOM 449 CA PRO A 65 -1.763 0.731 13.108 1.00 1.00

ATOM 450 C PRO A 65 -1.287 1.559 11.913 1.00 1.00

ATOM 451 O PRO A 65 -2.055 1.707 10.960 1.00 1.00

ATOM 452 CB PRO A 65 -2.405 1.634 14.171 1.00 1.00

ATOM 453 CG PRO A 65 -1.394 1.743 15.251 1.00 1.00

ATOM 454 CD PRO A 65 -0.679 0.430 15.269 1.00 1.00

ATOM 455 N THR A 66 -0.057 2.069 11.955 1.00 1.00

ATOM 456 CA THR A 66 0.486 2.892 10.866 1.00 1.00

ATOM 457 C THR A 66 0.716 2.099 9.577 1.00 1.00

ATOM 458 O THR A 66 0.812 2.682 8.494 1.00 1.00

ATOM 459 CB THR A 66 1.794 3.599 11.294 1.00 1.00

ATOM 460 OG1 THR A 66 2.730 2.631 11.784 1.00 1.00

ATOM 461 CG2 THR A 66 1.555 4.519 12.491 1.00 1.00

ATOM 462 N LYS A 67 0.809 0.776 9.701 1.00 1.00

ATOM 463 CA LYS A 67 0.960 -0.108 8.546 1.00 1.00

ATOM 464 C LYS A 67 -0.332 -0.190 7.729 1.00 1.00

ATOM 465 O LYS A 67 -0.290 -0.364 6.510 1.00 1.00

ATOM 466 CB LYS A 67 1.399 -1.510 8.987 1.00 1.00

ATOM 467 CG LYS A 67 2.718 -1.548 9.752 1.00 1.00

ATOM 468 CD LYS A 67 3.770 -2.365 9.017 1.00 1.00

ATOM 469 CE LYS A 67 5.012 -2.564 9.877 1.00 1.00

ATOM 470 NZ LYS A 67 6.200 -1.861 9.313 1.00 1.00

ATOM 471 N SER A 68 -1.468 -0.051 8.412 1.00 1.00

ATOM 472 CA SER A 68 -2.788 -0.138 7.783 1.00 1.00

ATOM 473 C SER A 68 -3.482 1.216 7.613 1.00 1.00

ATOM 474 O SER A 68 -4.354 1.361 6.756 1.00 1.00

ATOM 475 CB SER A 68 -3.696 -1.071 8.590 1.00 1.00

ATOM 476 OG SER A 68 -3.262 -2.417 8.498 1.00 1.00

ATOM 477 N PHE A 69 -3.089 2.198 8.424 1.00 1.00

ATOM 478 CA PHE A 69 -3.785 3.484 8.503 1.00 1.00

ATOM 479 C PHE A 69 -2.818 4.656 8.346 1.00 1.00

ATOM 480 O PHE A 69 -1.797 4.715 9.030 1.00 1.00

ATOM 481 CB PHE A 69 -4.526 3.571 9.848 1.00 1.00

ATOM 482 CG PHE A 69 -5.353 4.820 10.027 1.00 1.00

ATOM 483 CD1 PHE A 69 -4.818 5.944 10.651 1.00 1.00

ATOM 484 CD2 PHE A 69 -6.681 4.857 9.608 1.00 1.00

ATOM 485 CE1 PHE A 69 -5.584 7.092 10.837 1.00 1.00

ATOM 486 CE2 PHE A 69 -7.453 6.002 9.788 1.00 1.00

ATOM 487 CZ PHE A 69 -6.903 7.122 10.406 1.00 1.00

ATOM 488 N ASP A 70 -3.149 5.583 7.444 1.00 1.00

ATOM 489 CA ASP A 70 -2.368 6.806 7.242 1.00 1.00

ATOM 490 C ASP A 70 -3.255 7.972 6.806 1.00 1.00

ATOM 491 O ASP A 70 -4.283 7.768 6.155 1.00 1.00

ATOM 492 CB ASP A 70 -1.247 6.586 6.215 1.00 1.00

ATOM 493 CG ASP A 70 -0.085 7.569 6.380 1.00 1.00

ATOM 494 OD1 ASP A 70 -0.192 8.525 7.181 1.00 1.00

ATOM 495 OD2 ASP A 70 0.985 7.459 5.747 1.00 1.00

ATOM 496 N THR A 71 -2.851 9.187 7.177 1.00 1.00

ATOM 497 CA THR A 71 -3.588 10.408 6.839 1.00 1.00

ATOM 498 C THR A 71 -2.666 11.528 6.350 1.00 1.00

ATOM 499 O THR A 71 -1.437 11.421 6.428 1.00 1.00

ATOM 500 CB THR A 71 -4.417 10.921 8.046 1.00 1.00

ATOM 501 OG1 THR A 71 -3.589 10.970 9.214 1.00 1.00

ATOM 502 CG2 THR A 71 -5.527 9.945 8.418 1.00 1.00

ATOM 503 N ALA A 72 -3.274 12.604 5.854 1.00 1.00

ATOM 504 CA ALA A 72 -2.549 13.800 5.431 1.00 1.00

ATOM 505 C ALA A 72 -3.406 15.051 5.611 1.00 1.00

ATOM 506 O ALA A 72 -4.617 15.022 5.370 1.00 1.00

ATOM 507 CB ALA A 72 -2.097 13.663 3.987 1.00 1.00

ATOM 508 N ILE A 73 -2.772 16.142 6.040 1.00 1.00

ATOM 509 CA ILE A 73 -3.456 17.418 6.268 1.00 1.00

ATOM 510 C ILE A 73 -2.869 18.515 5.372 1.00 1.00

ATOM 511 O ILE A 73 -1.659 18.753 5.390 1.00 1.00

ATOM 512 CB ILE A 73 -3.369 17.834 7.768 1.00 1.00

ATOM 513 CG1 ILE A 73 -3.973 16.756 8.674 1.00 1.00

ATOM 514 CG2 ILE A 73 -4.062 19.176 8.005 1.00 1.00

ATOM 515 CD1 ILE A 73 -3.241 16.574 9.994 1.00 1.00

ATOM 516 N TYR A 74 -3.730 19.175 4.600 1.00 1.00

ATOM 517 CA TYR A 74 -3.309 20.244 3.689 1.00 1.00

ATOM 518 C TYR A 74 -4.097 21.540 3.911 1.00 1.00

ATOM 519 O TYR A 74 -5.121 21.761 3.263 1.00 1.00

ATOM 520 CB TYR A 74 -3.439 19.797 2.226 1.00 1.00

ATOM 521 CG TYR A 74 -2.804 18.461 1.925 1.00 1.00

ATOM 522 CD1 TYR A 74 -3.590 17.332 1.704 1.00 1.00

ATOM 523 CD2 TYR A 74 -1.416 18.324 1.859 1.00 1.00

ATOM 524 CE1 TYR A 74 -3.015 16.103 1.426 1.00 1.00

ATOM 525 CE2 TYR A 74 -0.831 17.097 1.585 1.00 1.00

ATOM 526 CZ TYR A 74 -1.636 15.992 1.368 1.00 1.00

ATOM 527 OH TYR A 74 -1.067 14.772 1.096 1.00 1.00

ATOM 528 N PRO A 75 -3.624 22.388 4.829 1.00 1.00

ATOM 529 CA PRO A 75 -4.261 23.690 5.086 1.00 1.00

ATOM 530 C PRO A 75 -4.358 24.591 3.849 1.00 1.00

ATOM 531 O PRO A 75 -5.389 25.239 3.666 1.00 1.00

ATOM 532 CB PRO A 75 -3.359 24.327 6.156 1.00 1.00

ATOM 533 CG PRO A 75 -2.097 23.540 6.130 1.00 1.00

ATOM 534 CD PRO A 75 -2.470 22.157 5.716 1.00 1.00

ATOM 535 N ASP A 76 -3.307 24.587 2.992 1.00 1.00

ATOM 536 CA ASP A 76 -3.318 25.448 1.799 1.00 1.00

ATOM 537 C ASP A 76 -4.378 24.986 0.784 1.00 1.00

ATOM 538 O ASP A 76 -4.891 25.784 -0.008 1.00 1.00

ATOM 539 CB ASP A 76 -1.963 25.415 1.075 1.00 1.00

ATOM 540 CG ASP A 76 -0.795 25.511 2.033 1.00 1.00

ATOM 541 OD1 ASP A 76 -0.708 26.514 2.772 1.00 1.00

ATOM 542 OD2 ASP A 76 0.037 24.580 2.044 1.00 1.00

ATOM 543 N ARG A 77 -4.694 23.694 0.821 1.00 1.00

ATOM 544 CA ARG A 77 -5.688 23.113 -0.114 1.00 1.00

ATOM 545 C ARG A 77 -7.053 22.902 0.585 1.00 1.00

ATOM 546 O ARG A 77 -8.032 22.493 -0.051 1.00 1.00

ATOM 547 CB ARG A 77 -5.180 21.782 -0.663 1.00 1.00

ATOM 548 CG ARG A 77 -4.321 21.971 -1.922 1.00 1.00

ATOM 549 CD ARG A 77 -4.010 20.666 -2.640 1.00 1.00

ATOM 550 NE ARG A 77 -2.914 19.939 -2.012 1.00 1.00

ATOM 551 CZ ARG A 77 -2.680 18.644 -2.179 1.00 1.00

ATOM 552 NH1 ARG A 77 -3.463 17.898 -2.975 1.00 1.00

ATOM 553 NH2 ARG A 77 -1.670 18.001 -1.584 1.00 1.00

ATOM 554 N LYS A 78 -7.060 23.192 1.880 1.00 1.00

ATOM 555 CA LYS A 78 -8.269 23.113 2.744 1.00 1.00

ATOM 556 C LYS A 78 -8.909 21.706 2.776 1.00 1.00

ATOM 557 O LYS A 78 -10.145 21.564 2.856 1.00 1.00

ATOM 558 CB LYS A 78 -9.328 24.078 2.224 1.00 1.00

ATOM 559 CG LYS A 78 -8.941 25.546 2.326 1.00 1.00

ATOM 560 CD LYS A 78 -8.200 26.015 1.084 1.00 1.00

ATOM 561 CE LYS A 78 -7.708 27.444 1.241 1.00 1.00

ATOM 562 NZ LYS A 78 -6.903 27.886 0.068 1.00 1.00

ATOM 563 N ILE A 79 -8.074 20.679 2.727 1.00 1.00

ATOM 564 CA ILE A 79 -8.564 19.280 2.749 1.00 1.00

ATOM 565 C ILE A 79 -7.718 18.398 3.680 1.00 1.00

ATOM 566 O ILE A 79 -6.561 18.704 3.989 1.00 1.00

ATOM 567 CB ILE A 79 -8.521 18.681 1.342 1.00 1.00

ATOM 568 CG1 ILE A 79 -7.186 18.936 0.618 1.00 1.00

ATOM 569 CG2 ILE A 79 -9.622 19.248 0.436 1.00 1.00

ATOM 570 CD1 ILE A 79 -6.749 17.772 -0.280 1.00 1.00

ATOM 571 N ILE A 80 -8.376 17.313 4.125 1.00 1.00

ATOM 572 CA ILE A 80 -7.708 16.224 4.836 1.00 1.00

ATOM 573 C ILE A 80 -8.018 14.900 4.138 1.00 1.00

ATOM 574 O ILE A 80 -9.115 14.716 3.605 1.00 1.00

ATOM 575 CB ILE A 80 -8.105 16.176 6.343 1.00 1.00

ATOM 576 CG1 ILE A 80 -9.629 16.180 6.523 1.00 1.00

ATOM 577 CG2 ILE A 80 -7.449 17.328 7.109 1.00 1.00

ATOM 578 CD1 ILE A 80 -10.104 15.680 7.884 1.00 1.00

ATOM 579 N VAL A 81 -7.048 13.990 4.125 1.00 1.00

ATOM 580 CA VAL A 81 -7.178 12.733 3.385 1.00 1.00

ATOM 581 C VAL A 81 -6.945 11.520 4.284 1.00 1.00

ATOM 582 O VAL A 81 -6.044 11.524 5.121 1.00 1.00

ATOM 583 CB VAL A 81 -6.216 12.668 2.164 1.00 1.00

ATOM 584 CG1 VAL A 81 -6.644 11.571 1.191 1.00 1.00

ATOM 585 CG2 VAL A 81 -6.138 14.015 1.446 1.00 1.00

ATOM 586 N PHE A 82 -7.763 10.489 4.092 1.00 1.00

ATOM 587 CA PHE A 82 -7.670 9.248 4.856 1.00 1.00

ATOM 588 C PHE A 82 -7.307 8.086 3.937 1.00 1.00

ATOM 589 O PHE A 82 -7.871 7.952 2.851 1.00 1.00

ATOM 590 CB PHE A 82 -9.005 8.942 5.547 1.00 1.00

ATOM 591 CG PHE A 82 -9.433 9.982 6.550 1.00 1.00

ATOM 592 CD1 PHE A 82 -10.060 11.153 6.136 1.00 1.00

ATOM 593 CD2 PHE A 82 -9.226 9.779 7.909 1.00 1.00

ATOM 594 CE1 PHE A 82 -10.462 12.113 7.062 1.00 1.00

ATOM 595 CE2 PHE A 82 -9.626 10.732 8.843 1.00 1.00

ATOM 596 CZ PHE A 82 -10.244 11.901 8.418 1.00 1.00

ATOM 597 N LEU A 83 -6.365 7.252 4.371 1.00 1.00

ATOM 598 CA LEU A 83 -6.029 6.031 3.639 1.00 1.00

ATOM 599 C LEU A 83 -5.953 4.817 4.558 1.00 1.00

ATOM 600 O LEU A 83 -5.056 4.709 5.397 1.00 1.00

ATOM 601 CB LEU A 83 -4.728 6.194 2.837 1.00 1.00

ATOM 602 CG LEU A 83 -4.128 4.965 2.132 1.00 1.00

ATOM 603 CD1 LEU A 83 -5.061 4.393 1.069 1.00 1.00

ATOM 604 CD2 LEU A 83 -2.773 5.292 1.523 1.00 1.00

ATOM 605 N PHE A 84 -6.911 3.910 4.390 1.00 1.00

ATOM 606 CA PHE A 84 -6.908 2.639 5.104 1.00 1.00

ATOM 607 C PHE A 84 -6.803 1.485 4.118 1.00 1.00

ATOM 608 O PHE A 84 -7.534 1.432 3.130 1.00 1.00

ATOM 609 CB PHE A 84 -8.163 2.485 5.972 1.00 1.00

ATOM 610 CG PHE A 84 -8.277 1.143 6.656 1.00 1.00

ATOM 611 CD1 PHE A 84 -7.495 0.843 7.770 1.00 1.00

ATOM 612 CD2 PHE A 84 -9.163 0.178 6.184 1.00 1.00

ATOM 613 CE1 PHE A 84 -7.593 -0.396 8.405 1.00 1.00

ATOM 614 CE2 PHE A 84 -9.272 -1.066 6.812 1.00 1.00

ATOM 615 CZ PHE A 84 -8.483 -1.351 7.925 1.00 1.00

ATOM 616 N ALA A 85 -5.871 0.578 4.395 1.00 1.00

ATOM 617 CA ALA A 85 -5.725 -0.669 3.652 1.00 1.00

ATOM 618 C ALA A 85 -5.080 -1.691 4.577 1.00 1.00

ATOM 619 O ALA A 85 -3.870 -1.642 4.819 1.00 1.00

ATOM 620 CB ALA A 85 -4.886 -0.462 2.394 1.00 1.00

ATOM 621 N GLU A 86 -5.897 -2.603 5.104 1.00 1.00

ATOM 622 CA GLU A 86 -5.441 -3.590 6.085 1.00 1.00

ATOM 623 C GLU A 86 -4.192 -4.332 5.600 1.00 1.00

ATOM 624 O GLU A 86 -4.129 -4.780 4.455 1.00 1.00

ATOM 625 CB GLU A 86 -6.580 -4.544 6.480 1.00 1.00

ATOM 626 CG GLU A 86 -6.933 -5.626 5.468 1.00 1.00

ATOM 627 CD GLU A 86 -6.156 -6.909 5.695 1.00 1.00

ATOM 628 OE1 GLU A 86 -5.828 -7.206 6.861 1.00 1.00

ATOM 629 OE2 GLU A 86 -5.865 -7.618 4.709 1.00 1.00

ATOM 630 N ASP A 87 -3.200 -4.442 6.480 1.00 1.00

ATOM 631 CA ASP A 87 -1.862 -4.884 6.088 1.00 1.00

ATOM 632 C ASP A 87 -1.496 -6.315 6.505 1.00 1.00

ATOM 633 O ASP A 87 -0.317 -6.682 6.516 1.00 1.00

ATOM 634 CB ASP A 87 -0.816 -3.887 6.608 1.00 1.00

ATOM 635 CG ASP A 87 0.461 -3.893 5.790 1.00 1.00

ATOM 636 OD1 ASP A 87 0.380 -3.906 4.543 1.00 1.00

ATOM 637 OD2 ASP A 87 1.595 -3.889 6.314 1.00 1.00

ATOM 638 N SER A 88 -2.501 -7.124 6.835 1.00 1.00

ATOM 639 CA SER A 88 -2.261 -8.515 7.233 1.00 1.00

ATOM 640 C SER A 88 -1.913 -9.402 6.041 1.00 1.00

ATOM 641 O SER A 88 -1.093 -10.314 6.158 1.00 1.00

ATOM 642 CB SER A 88 -3.467 -9.087 7.983 1.00 1.00

ATOM 643 OG SER A 88 -4.495 -9.475 7.088 1.00 1.00

ATOM 644 N GLY A 89 -2.548 -9.129 4.901 1.00 1.00

ATOM 645 CA GLY A 89 -2.381 -9.932 3.702 1.00 1.00

ATOM 646 C GLY A 89 -3.448 -11.002 3.560 1.00 1.00

ATOM 647 O GLY A 89 -3.565 -11.633 2.506 1.00 1.00

ATOM 648 N THR A 90 -4.227 -11.201 4.623 1.00 1.00

ATOM 649 CA THR A 90 -5.229 -12.270 4.684 1.00 1.00

ATOM 650 C THR A 90 -6.643 -11.740 4.925 1.00 1.00

ATOM 651 O THR A 90 -7.622 -12.474 4.766 1.00 1.00

ATOM 652 CB THR A 90 -4.868 -13.289 5.795 1.00 1.00

ATOM 653 OG1 THR A 90 -4.710 -12.603 7.044 1.00 1.00

ATOM 654 CG2 THR A 90 -3.494 -13.912 5.551 1.00 1.00

ATOM 655 N GLY A 91 -6.745 -10.471 5.312 1.00 1.00

ATOM 656 CA GLY A 91 -8.020 -9.869 5.664 1.00 1.00

ATOM 657 C GLY A 91 -8.254 -9.830 7.165 1.00 1.00

ATOM 658 O GLY A 91 -9.351 -9.497 7.618 1.00 1.00

ATOM 659 N ALA A 92 -7.216 -10.163 7.929 1.00 1.00

ATOM 660 CA ALA A 92 -7.306 -10.260 9.387 1.00 1.00

ATOM 661 C ALA A 92 -7.448 -8.906 10.084 1.00 1.00

ATOM 662 O ALA A 92 -8.075 -8.815 11.143 1.00 1.00

ATOM 663 CB ALA A 92 -6.108 -11.024 9.943 1.00 1.00

ATOM 664 N TYR A 93 -6.872 -7.861 9.492 1.00 1.00

ATOM 665 CA TYR A 93 -6.898 -6.525 10.093 1.00 1.00

ATOM 666 C TYR A 93 -7.986 -5.624 9.501 1.00 1.00

ATOM 667 O TYR A 93 -7.946 -4.400 9.652 1.00 1.00

ATOM 668 CB TYR A 93 -5.519 -5.855 10.003 1.00 1.00

ATOM 669 CG TYR A 93 -4.407 -6.589 10.732 1.00 1.00

ATOM 670 CD1 TYR A 93 -3.086 -6.492 10.297 1.00 1.00

ATOM 671 CD2 TYR A 93 -4.674 -7.372 11.860 1.00 1.00

ATOM 672 CE1 TYR A 93 -2.058 -7.161 10.960 1.00 1.00

ATOM 673 CE2 TYR A 93 -3.653 -8.044 12.528 1.00 1.00

ATOM 674 CZ TYR A 93 -2.349 -7.934 12.072 1.00 1.00

ATOM 675 OH TYR A 93 -1.337 -8.594 12.729 1.00 1.00

ATOM 676 N ALA A 94 -8.957 -6.239 8.830 1.00 1.00

ATOM 677 CA ALA A 94 -10.111 -5.522 8.292 1.00 1.00

ATOM 678 C ALA A 94 -11.007 -5.008 9.418 1.00 1.00

ATOM 679 O ALA A 94 -11.034 -5.582 10.509 1.00 1.00

ATOM 680 CB ALA A 94 -10.901 -6.423 7.353 1.00 1.00

ATOM 681 N ILE A 95 -11.706 -3.892 9.172 1.00 1.00

ATOM 682 CA ILE A 95 -12.706 -3.378 10.121 1.00 1.00

ATOM 683 C ILE A 95 -13.944 -4.275 10.054 1.00 1.00

ATOM 684 O ILE A 95 -14.535 -4.477 8.985 1.00 1.00

ATOM 685 CB ILE A 95 -13.048 -1.940 9.749 1.00 1.00

ATOM 686 CG1 ILE A 95 -11.900 -0.972 10.085 1.00 1.00

ATOM 687 CG2 ILE A 95 -14.286 -1.418 10.479 1.00 1.00

ATOM 688 CD1 ILE A 95 -11.808 0.223 9.135 1.00 1.00

ATOM 689 N THR A 96 -14.319 -4.802 11.209 1.00 1.00

ATOM 690 CA THR A 96 -15.449 -5.734 11.288 1.00 1.00

ATOM 691 C THR A 96 -16.627 -5.215 12.140 1.00 1.00

ATOM 692 O THR A 96 -17.740 -5.761 12.086 1.00 1.00

ATOM 693 CB THR A 96 -14.965 -7.061 11.893 1.00 1.00

ATOM 694 OG1 THR A 96 -14.261 -6.833 13.105 1.00 1.00

ATOM 695 CG2 THR A 96 -14.013 -7.821 10.963 1.00 1.00

ATOM 696 N LYS A 97 -16.408 -4.169 12.925 1.00 1.00

ATOM 697 CA LYS A 97 -17.497 -3.631 13.782 1.00 1.00

ATOM 698 C LYS A 97 -17.640 -2.103 13.658 1.00 1.00

ATOM 699 O LYS A 97 -16.715 -1.396 13.233 1.00 1.00

ATOM 700 CB LYS A 97 -17.232 -3.974 15.257 1.00 1.00

ATOM 701 CG LYS A 97 -16.122 -3.156 15.887 1.00 1.00

ATOM 702 CD LYS A 97 -16.019 -3.431 17.378 1.00 1.00

ATOM 703 CE LYS A 97 -17.316 -3.091 18.093 1.00 1.00

ATOM 704 NZ LYS A 97 -17.116 -2.933 19.561 1.00 1.00

ATOM 705 N ASP A 98 -18.827 -1.648 14.046 1.00 1.00

ATOM 706 CA ASP A 98 -19.196 -0.223 14.022 1.00 1.00

ATOM 707 C ASP A 98 -18.455 0.521 15.131 1.00 1.00

ATOM 708 O ASP A 98 -18.084 -0.069 16.156 1.00 1.00

ATOM 709 CB ASP A 98 -20.704 -0.062 14.238 1.00 1.00

ATOM 710 CG ASP A 98 -21.518 -0.365 12.982 1.00 1.00

ATOM 711 OD1 ASP A 98 -21.065 -1.194 12.111 1.00 1.00

ATOM 712 OD2 ASP A 98 -22.656 0.207 12.793 1.00 1.00

ATOM 713 N GLY A 99 -18.235 1.812 14.914 1.00 1.00

ATOM 714 CA GLY A 99 -17.588 2.654 15.905 1.00 1.00

ATOM 715 C GLY A 99 -16.713 3.737 15.310 1.00 1.00

ATOM 716 O GLY A 99 -16.900 4.144 14.162 1.00 1.00

ATOM 717 N VAL A 100 -15.750 4.199 16.102 1.00 1.00

ATOM 718 CA VAL A 100 -14.863 5.289 15.703 1.00 1.00

ATOM 719 C VAL A 100 -13.776 4.797 14.747 1.00 1.00

ATOM 720 O VAL A 100 -12.943 3.966 15.112 1.00 1.00

ATOM 721 CB VAL A 100 -14.220 5.985 16.933 1.00 1.00

ATOM 722 CG1 VAL A 100 -13.409 7.206 16.504 1.00 1.00

ATOM 723 CG2 VAL A 100 -15.284 6.383 17.953 1.00 1.00

ATOM 724 N PHE A 101 -13.805 5.315 13.521 1.00 1.00

ATOM 725 CA PHE A 101 -12.786 5.018 12.517 1.00 1.00

ATOM 726 C PHE A 101 -11.543 5.869 12.745 1.00 1.00

ATOM 727 O PHE A 101 -10.421 5.361 12.719 1.00 1.00

ATOM 728 CB PHE A 101 -13.345 5.239 11.104 1.00 1.00

ATOM 729 CG PHE A 101 -12.304 5.184 10.014 1.00 1.00

ATOM 730 CD1 PHE A 101 -11.718 3.974 9.649 1.00 1.00

ATOM 731 CD2 PHE A 101 -11.918 6.344 9.345 1.00 1.00

ATOM 732 CE1 PHE A 101 -10.759 3.922 8.639 1.00 1.00

ATOM 733 CE2 PHE A 101 -10.960 6.300 8.334 1.00 1.00

ATOM 734 CZ PHE A 101 -10.380 5.087 7.979 1.00 1.00

ATOM 735 N ALA A 102 -11.755 7.165 12.969 1.00 1.00

ATOM 736 CA ALA A 102 -10.666 8.112 13.182 1.00 1.00

ATOM 737 C ALA A 102 -11.079 9.253 14.108 1.00 1.00

ATOM 738 O ALA A 102 -12.267 9.545 14.259 1.00 1.00

ATOM 739 CB ALA A 102 -10.169 8.660 11.848 1.00 1.00

ATOM 740 N LYS A 103 -10.087 9.889 14.726 1.00 1.00

ATOM 741 CA LYS A 103 -10.311 11.058 15.570 1.00 1.00

ATOM 742 C LYS A 103 -9.498 12.237 15.042 1.00 1.00

ATOM 743 O LYS A 103 -8.267 12.181 14.999 1.00 1.00

ATOM 744 CB LYS A 103 -9.927 10.764 17.023 1.00 1.00

ATOM 745 CG LYS A 103 -10.616 9.545 17.611 1.00 1.00

ATOM 746 CD LYS A 103 -11.740 9.946 18.551 1.00 1.00

ATOM 747 CE LYS A 103 -12.190 8.773 19.407 1.00 1.00

ATOM 748 NZ LYS A 103 -12.729 9.219 20.721 1.00 1.00

ATOM 749 N ILE A 104 -10.197 13.293 14.634 1.00 1.00

ATOM 750 CA ILE A 104 -9.550 14.505 14.141 1.00 1.00

ATOM 751 C ILE A 104 -9.290 15.462 15.301 1.00 1.00

ATOM 752 O ILE A 104 -10.224 15.918 15.963 1.00 1.00

ATOM 753 CB ILE A 104 -10.406 15.196 13.043 1.00 1.00

ATOM 754 CG1 ILE A 104 -10.749 14.219 11.914 1.00 1.00

ATOM 755 CG2 ILE A 104 -9.679 16.427 12.488 1.00 1.00

ATOM 756 CD1 ILE A 104 -12.005 14.584 11.138 1.00 1.00

ATOM 757 N ARG A 105 -8.016 15.752 15.546 1.00 1.00

ATOM 758 CA ARG A 105 -7.632 16.715 16.572 1.00 1.00

ATOM 759 C ARG A 105 -7.489 18.097 15.945 1.00 1.00

ATOM 760 O ARG A 105 -6.650 18.304 15.063 1.00 1.00

ATOM 761 CB ARG A 105 -6.332 16.289 17.258 1.00 1.00

ATOM 762 CG ARG A 105 -6.524 15.267 18.369 1.00 1.00

ATOM 763 CD ARG A 105 -5.234 14.848 19.059 1.00 1.00

ATOM 764 NE ARG A 105 -4.429 13.962 18.219 1.00 1.00

ATOM 765 CZ ARG A 105 -3.262 14.286 17.673 1.00 1.00

ATOM 766 NH1 ARG A 105 -2.735 15.489 17.871 1.00 1.00

ATOM 767 NH2 ARG A 105 -2.616 13.402 16.925 1.00 1.00

ATOM 768 N ALA A 106 -8.321 19.031 16.396 1.00 1.00

ATOM 769 CA ALA A 106 -8.329 20.386 15.852 1.00 1.00

ATOM 770 C ALA A 106 -8.358 21.449 16.943 1.00 1.00

ATOM 771 O ALA A 106 -9.085 21.323 17.931 1.00 1.00

ATOM 772 CB ALA A 106 -9.505 20.570 14.901 1.00 1.00

ATOM 773 N THR A 107 -7.533 22.468 16.765 1.00 1.00

ATOM 774 CA THR A 107 -7.584 23.629 17.663 1.00 1.00

ATOM 775 C THR A 107 -8.496 24.704 17.049 1.00 1.00

ATOM 776 O THR A 107 -8.288 25.151 15.914 1.00 1.00

ATOM 777 CB THR A 107 -6.164 24.186 17.854 1.00 1.00

ATOM 778 OG1 THR A 107 -5.296 23.159 18.314 1.00 1.00

ATOM 779 CG2 THR A 107 -6.093 25.324 18.874 1.00 1.00

ATOM 780 N VAL A 108 -9.496 25.092 17.826 1.00 1.00

ATOM 781 CA VAL A 108 -10.478 26.112 17.408 1.00 1.00

ATOM 782 C VAL A 108 -9.811 27.493 17.414 1.00 1.00

ATOM 783 O VAL A 108 -9.330 27.968 18.453 1.00 1.00

ATOM 784 CB VAL A 108 -11.668 26.104 18.366 1.00 1.00

ATOM 785 CG1 VAL A 108 -12.824 26.983 17.885 1.00 1.00

ATOM 786 CG2 VAL A 108 -12.261 24.704 18.564 1.00 1.00

ATOM 787 N LYS A 109 -9.803 28.105 16.237 1.00 1.00

ATOM 788 CA LYS A 109 -9.169 29.425 16.028 1.00 1.00

ATOM 789 C LYS A 109 -10.176 30.593 16.048 1.00 1.00

ATOM 790 O LYS A 109 -9.808 31.744 16.314 1.00 1.00

ATOM 791 CB LYS A 109 -8.461 29.482 14.669 1.00 1.00

ATOM 792 CG LYS A 109 -7.489 28.341 14.432 1.00 1.00

ATOM 793 CD LYS A 109 -6.058 28.771 14.708 1.00 1.00

ATOM 794 CE LYS A 109 -5.359 29.221 13.435 1.00 1.00

ATOM 795 NZ LYS A 109 -4.039 29.849 13.718 1.00 1.00

ATOM 796 N SER A 110 -11.436 30.302 15.759 1.00 1.00

ATOM 797 CA SER A 110 -12.482 31.354 15.732 1.00 1.00

ATOM 798 C SER A 110 -13.859 30.775 16.071 1.00 1.00

ATOM 799 O SER A 110 -14.041 29.554 16.157 1.00 1.00

ATOM 800 CB SER A 110 -12.542 31.992 14.336 1.00 1.00

ATOM 801 OG SER A 110 -13.792 31.716 13.720 1.00 1.00

ATOM 802 N SER A 111 -14.793 31.707 16.423 1.00 1.00

ATOM 803 CA SER A 111 -16.133 31.351 16.887 1.00 1.00

ATOM 804 C SER A 111 -17.076 30.911 15.763 1.00 1.00

ATOM 805 O SER A 111 -18.142 30.351 16.027 1.00 1.00

ATOM 806 CB SER A 111 -16.751 32.518 17.660 1.00 1.00

ATOM 807 OG SER A 111 -16.996 33.620 16.805 1.00 1.00

ATOM 808 N ALA A 112 -16.678 31.177 14.521 1.00 1.00

ATOM 809 CA ALA A 112 -17.452 30.804 13.339 1.00 1.00

ATOM 810 C ALA A 112 -17.609 29.283 13.227 1.00 1.00

ATOM 811 O ALA A 112 -16.764 28.540 13.737 1.00 1.00

ATOM 812 CB ALA A 112 -16.796 31.370 12.080 1.00 1.00

ATOM 813 N PRO A 113 -18.684 28.818 12.584 1.00 1.00

ATOM 814 CA PRO A 113 -18.892 27.379 12.372 1.00 1.00

ATOM 815 C PRO A 113 -17.728 26.727 11.626 1.00 1.00

ATOM 816 O PRO A 113 -17.291 27.233 10.588 1.00 1.00

ATOM 817 CB PRO A 113 -20.168 27.331 11.525 1.00 1.00

ATOM 818 CG PRO A 113 -20.876 28.604 11.832 1.00 1.00

ATOM 819 CD PRO A 113 -19.794 29.620 12.033 1.00 1.00

ATOM 820 N GLY A 114 -17.226 25.623 12.175 1.00 1.00

ATOM 821 CA GLY A 114 -16.120 24.891 11.585 1.00 1.00

ATOM 822 C GLY A 114 -16.594 23.625 10.905 1.00 1.00

ATOM 823 O GLY A 114 -16.533 22.541 11.485 1.00 1.00

ATOM 824 N TYR A 115 -17.068 23.772 9.669 1.00 1.00

ATOM 825 CA TYR A 115 -17.593 22.656 8.894 1.00 1.00

ATOM 826 C TYR A 115 -16.494 21.693 8.452 1.00 1.00

ATOM 827 O TYR A 115 -15.465 22.112 7.915 1.00 1.00

ATOM 828 CB TYR A 115 -18.350 23.162 7.659 1.00 1.00

ATOM 829 CG TYR A 115 -19.454 24.156 7.948 1.00 1.00

ATOM 830 CD1 TYR A 115 -19.287 25.512 7.667 1.00 1.00

ATOM 831 CD2 TYR A 115 -20.672 23.741 8.487 1.00 1.00

ATOM 832 CE1 TYR A 115 -20.300 26.433 7.926 1.00 1.00

ATOM 833 CE2 TYR A 115 -21.692 24.651 8.752 1.00 1.00

ATOM 834 CZ TYR A 115 -21.500 25.993 8.465 1.00 1.00

ATOM 835 OH TYR A 115 -22.504 26.897 8.723 1.00 1.00

ATOM 836 N ILE A 116 -16.718 20.405 8.701 1.00 1.00

ATOM 837 CA ILE A 116 -15.913 19.343 8.104 1.00 1.00

ATOM 838 C ILE A 116 -16.845 18.493 7.241 1.00 1.00

ATOM 839 O ILE A 116 -17.669 17.728 7.752 1.00 1.00

ATOM 840 CB ILE A 116 -15.175 18.501 9.178 1.00 1.00

ATOM 841 CG1 ILE A 116 -14.211 19.386 9.981 1.00 1.00

ATOM 842 CG2 ILE A 116 -14.408 17.349 8.528 1.00 1.00

ATOM 843 CD1 ILE A 116 -13.829 18.833 11.341 1.00 1.00

ATOM 844 N THR A 117 -16.721 18.660 5.927 1.00 1.00

ATOM 845 CA THR A 117 -17.668 18.089 4.973 1.00 1.00

ATOM 846 C THR A 117 -17.020 17.068 4.046 1.00 1.00

ATOM 847 O THR A 117 -15.827 17.150 3.749 1.00 1.00

ATOM 848 CB THR A 117 -18.327 19.202 4.130 1.00 1.00

ATOM 849 OG1 THR A 117 -17.313 20.025 3.543 1.00 1.00

ATOM 850 CG2 THR A 117 -19.108 20.171 5.014 1.00 1.00

ATOM 851 N PHE A 118 -17.826 16.113 3.589 1.00 1.00

ATOM 852 CA PHE A 118 -17.391 15.104 2.630 1.00 1.00

ATOM 853 C PHE A 118 -17.104 15.744 1.274 1.00 1.00

ATOM 854 O PHE A 118 -17.915 16.518 0.756 1.00 1.00

ATOM 855 CB PHE A 118 -18.456 14.006 2.510 1.00 1.00

ATOM 856 CG PHE A 118 -18.348 13.172 1.258 1.00 1.00

ATOM 857 CD1 PHE A 118 -17.282 12.295 1.074 1.00 1.00

ATOM 858 CD2 PHE A 118 -19.327 13.253 0.270 1.00 1.00

ATOM 859 CE1 PHE A 118 -17.186 11.522 -0.081 1.00 1.00

ATOM 860 CE2 PHE A 118 -19.240 12.483 -0.886 1.00 1.00

ATOM 861 CZ PHE A 118 -18.166 11.615 -1.061 1.00 1.00

ATOM 862 N ASP A 119 -15.944 15.418 0.713 1.00 1.00

ATOM 863 CA ASP A 119 -15.514 15.978 -0.566 1.00 1.00

ATOM 864 C ASP A 119 -15.462 14.911 -1.662 1.00 1.00

ATOM 865 O ASP A 119 -16.107 15.053 -2.702 1.00 1.00

ATOM 866 CB ASP A 119 -14.158 16.683 -0.409 1.00 1.00

ATOM 867 CG ASP A 119 -13.710 17.398 -1.676 1.00 1.00

ATOM 868 OD1 ASP A 119 -14.570 17.895 -2.438 1.00 1.00

ATOM 869 OD2 ASP A 119 -12.507 17.518 -1.988 1.00 1.00

ATOM 870 N GLU A 120 -14.698 13.847 -1.419 1.00 1.00

ATOM 871 CA GLU A 120 -14.505 12.785 -2.402 1.00 1.00

ATOM 872 C GLU A 120 -14.289 11.436 -1.726 1.00 1.00

ATOM 873 O GLU A 120 -13.698 11.365 -0.651 1.00 1.00

ATOM 874 CB GLU A 120 -13.307 13.111 -3.306 1.00 1.00

ATOM 875 CG GLU A 120 -13.209 12.262 -4.568 1.00 1.00

ATOM 876 CD GLU A 120 -12.208 11.122 -4.448 1.00 1.00

ATOM 877 OE1 GLU A 120 -12.066 10.357 -5.427 1.00 1.00

ATOM 878 OE2 GLU A 120 -11.562 10.982 -3.386 1.00 1.00

ATOM 879 N VAL A 121 -14.787 10.376 -2.359 1.00 1.00

ATOM 880 CA VAL A 121 -14.524 9.006 -1.922 1.00 1.00

ATOM 881 C VAL A 121 -13.907 8.196 -3.072 1.00 1.00

ATOM 882 O VAL A 121 -14.329 8.315 -4.224 1.00 1.00

ATOM 883 CB VAL A 121 -15.801 8.317 -1.341 1.00 1.00

ATOM 884 CG1 VAL A 121 -16.883 8.108 -2.411 1.00 1.00

ATOM 885 CG2 VAL A 121 -15.451 7.003 -0.643 1.00 1.00

ATOM 886 N GLY A 122 -12.891 7.399 -2.752 1.00 1.00

ATOM 887 CA GLY A 122 -12.237 6.552 -3.733 1.00 1.00

ATOM 888 C GLY A 122 -12.717 5.119 -3.629 1.00 1.00

ATOM 889 O GLY A 122 -13.844 4.806 -4.012 1.00 1.00

ATOM 890 N GLY A 123 -11.854 4.253 -3.106 1.00 1.00

ATOM 891 CA GLY A 123 -12.196 2.863 -2.862 1.00 1.00

ATOM 892 C GLY A 123 -12.851 2.669 -1.507 1.00 1.00

ATOM 893 O GLY A 123 -12.618 3.447 -0.577 1.00 1.00

ATOM 894 N PHE A 124 -13.677 1.630 -1.406 1.00 1.00

ATOM 895 CA PHE A 124 -14.381 1.276 -0.174 1.00 1.00

ATOM 896 C PHE A 124 -14.875 -0.163 -0.301 1.00 1.00

ATOM 897 O PHE A 124 -16.054 -0.404 -0.563 1.00 1.00

ATOM 898 CB PHE A 124 -15.558 2.231 0.071 1.00 1.00

ATOM 899 CG PHE A 124 -15.754 2.604 1.516 1.00 1.00

ATOM 900 CD1 PHE A 124 -15.490 3.899 1.956 1.00 1.00

ATOM 901 CD2 PHE A 124 -16.218 1.668 2.438 1.00 1.00

ATOM 902 CE1 PHE A 124 -15.670 4.253 3.290 1.00 1.00

ATOM 903 CE2 PHE A 124 -16.403 2.013 3.777 1.00 1.00

ATOM 904 CZ PHE A 124 -16.128 3.309 4.202 1.00 1.00

ATOM 905 N ALA A 125 -13.962 -1.115 -0.116 1.00 1.00

ATOM 906 CA ALA A 125 -14.236 -2.518 -0.423 1.00 1.00

ATOM 907 C ALA A 125 -14.163 -3.452 0.784 1.00 1.00

ATOM 908 O ALA A 125 -13.469 -3.168 1.764 1.00 1.00

ATOM 909 CB ALA A 125 -13.302 -3.006 -1.531 1.00 1.00

ATOM 910 N ASP A 126 -14.885 -4.570 0.693 1.00 1.00

ATOM 911 CA ASP A 126 -14.852 -5.608 1.721 1.00 1.00

ATOM 912 C ASP A 126 -13.692 -6.586 1.508 1.00 1.00

ATOM 913 O ASP A 126 -12.838 -6.363 0.646 1.00 1.00

ATOM 914 CB ASP A 126 -16.202 -6.346 1.812 1.00 1.00

ATOM 915 CG ASP A 126 -16.616 -7.016 0.502 1.00 1.00

ATOM 916 OD1 ASP A 126 -15.743 -7.434 -0.288 1.00 1.00

ATOM 917 OD2 ASP A 126 -17.813 -7.182 0.189 1.00 1.00

ATOM 918 N ASN A 127 -13.729 -7.721 2.215 1.00 1.00

ATOM 919 CA ASN A 127 -12.597 -8.656 2.197 1.00 1.00

ATOM 920 C ASN A 127 -12.572 -9.449 0.879 1.00 1.00

ATOM 921 O ASN A 127 -11.578 -10.107 0.546 1.00 1.00

ATOM 922 CB ASN A 127 -12.710 -9.640 3.362 1.00 1.00

ATOM 923 CG ASN A 127 -11.971 -9.163 4.614 1.00 1.00

ATOM 924 OD1 ASN A 127 -10.893 -8.580 4.509 1.00 1.00

ATOM 925 ND2 ASN A 127 -12.490 -9.380 5.809 1.00 1.00

ATOM 926 N ASP A 128 -13.677 -9.371 0.147 1.00 1.00

ATOM 927 CA ASP A 128 -13.812 -10.073 -1.147 1.00 1.00

ATOM 928 C ASP A 128 -13.547 -9.089 -2.295 1.00 1.00

ATOM 929 O ASP A 128 -13.827 -9.387 -3.471 1.00 1.00

ATOM 930 CB ASP A 128 -15.210 -10.668 -1.277 1.00 1.00

ATOM 931 CG ASP A 128 -15.409 -11.876 -0.354 1.00 1.00

ATOM 932 OD1 ASP A 128 -14.398 -12.609 -0.024 1.00 1.00

ATOM 933 OD2 ASP A 128 -16.582 -12.160 0.094 1.00 1.00

ATOM 934 N LEU A 129 -13.013 -7.953 -1.885 1.00 1.00

ATOM 935 CA LEU A 129 -12.613 -6.860 -2.785 1.00 1.00

ATOM 936 C LEU A 129 -13.829 -6.298 -3.592 1.00 1.00

ATOM 937 O LEU A 129 -13.677 -5.768 -4.707 1.00 1.00

ATOM 938 CB LEU A 129 -11.535 -7.411 -3.734 1.00 1.00

ATOM 939 CG LEU A 129 -10.087 -7.149 -3.327 1.00 1.00

ATOM 940 CD1 LEU A 129 -9.789 -5.659 -3.358 1.00 1.00

ATOM 941 CD2 LEU A 129 -9.823 -7.658 -1.919 1.00 1.00

ATOM 942 N VAL A 130 -15.018 -6.427 -3.003 1.00 1.00

ATOM 943 CA VAL A 130 -16.273 -5.901 -3.605 1.00 1.00

ATOM 944 C VAL A 130 -16.510 -4.491 -3.051 1.00 1.00

ATOM 945 O VAL A 130 -16.544 -4.283 -1.831 1.00 1.00

ATOM 946 CB VAL A 130 -17.476 -6.791 -3.243 1.00 1.00

ATOM 947 CG1 VAL A 130 -18.807 -6.244 -3.779 1.00 1.00

ATOM 948 CG2 VAL A 130 -17.368 -8.213 -3.793 1.00 1.00

ATOM 949 N GLU A 131 -16.667 -3.540 -3.956 1.00 1.00

ATOM 950 CA GLU A 131 -16.888 -2.128 -3.580 1.00 1.00

ATOM 951 C GLU A 131 -18.280 -1.950 -2.964 1.00 1.00

ATOM 952 O GLU A 131 -19.293 -2.367 -3.540 1.00 1.00

ATOM 953 CB GLU A 131 -16.771 -1.222 -4.810 1.00 1.00

ATOM 954 CG GLU A 131 -15.411 -1.330 -5.511 1.00 1.00

ATOM 955 CD GLU A 131 -14.277 -0.632 -4.753 1.00 1.00

ATOM 956 OE1 GLU A 131 -14.538 0.349 -3.956 1.00 1.00

ATOM 957 OE2 GLU A 131 -13.057 -1.024 -4.912 1.00 1.00

ATOM 958 N GLN A 132 -18.301 -1.340 -1.786 1.00 1.00

ATOM 959 CA GLN A 132 -19.536 -1.222 -0.996 1.00 1.00

ATOM 960 C GLN A 132 -20.203 0.129 -1.227 1.00 1.00

ATOM 961 O GLN A 132 -19.540 1.126 -1.558 1.00 1.00

ATOM 962 CB GLN A 132 -19.227 -1.317 0.511 1.00 1.00

ATOM 963 CG GLN A 132 -18.485 -2.588 0.913 1.00 1.00

ATOM 964 CD GLN A 132 -19.322 -3.847 0.712 1.00 1.00

ATOM 965 OE1 GLN A 132 -20.295 -4.060 1.434 1.00 1.00

ATOM 966 NE2 GLN A 132 -18.992 -4.700 -0.237 1.00 1.00

ATOM 967 N LYS A 133 -21.514 0.143 -1.053 1.00 1.00

ATOM 968 CA LYS A 133 -22.273 1.384 -1.171 1.00 1.00

ATOM 969 C LYS A 133 -21.985 2.183 0.086 1.00 1.00

ATOM 970 O LYS A 133 -22.031 1.651 1.205 1.00 1.00

ATOM 971 CB LYS A 133 -23.760 1.126 -1.287 1.00 1.00

ATOM 972 CG LYS A 133 -24.208 0.578 -2.639 1.00 1.00

ATOM 973 CD LYS A 133 -23.889 -0.901 -2.784 1.00 1.00

ATOM 974 CE LYS A 133 -23.728 -1.291 -4.245 1.00 1.00

ATOM 975 NZ LYS A 133 -22.477 -2.066 -4.477 1.00 1.00

ATOM 976 N VAL A 134 -21.686 3.451 -0.083 1.00 1.00

ATOM 977 CA VAL A 134 -21.339 4.287 1.069 1.00 1.00

ATOM 978 C VAL A 134 -21.912 5.712 0.946 1.00 1.00

ATOM 979 O VAL A 134 -21.946 6.311 -0.138 1.00 1.00

ATOM 980 CB VAL A 134 -19.801 4.342 1.174 1.00 1.00

ATOM 981 CG1 VAL A 134 -19.138 5.184 0.072 1.00 1.00

ATOM 982 CG2 VAL A 134 -19.309 4.931 2.496 1.00 1.00

ATOM 983 N SER A 135 -22.351 6.210 2.097 1.00 1.00

ATOM 984 CA SER A 135 -22.927 7.559 2.228 1.00 1.00

ATOM 985 C SER A 135 -22.251 8.295 3.389 1.00 1.00

ATOM 986 O SER A 135 -21.669 7.670 4.290 1.00 1.00

ATOM 987 CB SER A 135 -24.439 7.481 2.496 1.00 1.00

ATOM 988 OG SER A 135 -24.694 6.787 3.708 1.00 1.00

ATOM 989 N PHE A 136 -22.236 9.619 3.300 1.00 1.00

ATOM 990 CA PHE A 136 -21.495 10.458 4.233 1.00 1.00

ATOM 991 C PHE A 136 -22.388 11.512 4.879 1.00 1.00

ATOM 992 O PHE A 136 -23.202 12.146 4.206 1.00 1.00

ATOM 993 CB PHE A 136 -20.327 11.143 3.515 1.00 1.00

ATOM 994 CG PHE A 136 -19.212 10.209 3.118 1.00 1.00

ATOM 995 CD1 PHE A 136 -19.315 9.419 1.971 1.00 1.00

ATOM 996 CD2 PHE A 136 -18.050 10.133 3.875 1.00 1.00

ATOM 997 CE1 PHE A 136 -18.286 8.557 1.600 1.00 1.00

ATOM 998 CE2 PHE A 136 -17.012 9.276 3.511 1.00 1.00

ATOM 999 CZ PHE A 136 -17.131 8.486 2.370 1.00 1.00

ATOM 1000 N ILE A 137 -22.228 11.691 6.189 1.00 1.00

ATOM 1001 CA ILE A 137 -22.909 12.761 6.918 1.00 1.00

ATOM 1002 C ILE A 137 -21.886 13.792 7.394 1.00 1.00

ATOM 1003 O ILE A 137 -20.936 13.457 8.103 1.00 1.00

ATOM 1004 CB ILE A 137 -23.743 12.195 8.101 1.00 1.00

ATOM 1005 CG1 ILE A 137 -24.949 11.406 7.577 1.00 1.00

ATOM 1006 CG2 ILE A 137 -24.208 13.318 9.037 1.00 1.00

ATOM 1007 CD1 ILE A 137 -25.541 10.425 8.577 1.00 1.00

ATOM 1008 N ASP A 138 -22.089 15.042 6.984 1.00 1.00

ATOM 1009 CA ASP A 138 -21.186 16.141 7.329 1.00 1.00

ATOM 1010 C ASP A 138 -21.325 16.557 8.791 1.00 1.00

ATOM 1011 O ASP A 138 -22.393 16.404 9.388 1.00 1.00

ATOM 1012 CB ASP A 138 -21.434 17.346 6.413 1.00 1.00

ATOM 1013 CG ASP A 138 -21.171 17.039 4.946 1.00 1.00

ATOM 1014 OD1 ASP A 138 -20.499 16.027 4.644 1.00 1.00

ATOM 1015 OD2 ASP A 138 -21.595 17.761 4.020 1.00 1.00

ATOM 1016 N GLY A 139 -20.236 17.079 9.353 1.00 1.00

ATOM 1017 CA GLY A 139 -20.214 17.534 10.733 1.00 1.00

ATOM 1018 C GLY A 139 -19.223 18.659 10.963 1.00 1.00

ATOM 1019 O GLY A 139 -19.099 19.566 10.135 1.00 1.00

ATOM 1020 N GLY A 140 -18.523 18.600 12.093 1.00 1.00

ATOM 1021 CA GLY A 140 -17.502 19.578 12.418 1.00 1.00

ATOM 1022 C GLY A 140 -17.576 20.128 13.831 1.00 1.00

ATOM 1023 O GLY A 140 -18.039 19.450 14.752 1.00 1.00

ATOM 1024 N VAL A 141 -17.111 21.366 13.993 1.00 1.00

ATOM 1025 CA VAL A 141 -17.039 22.028 15.294 1.00 1.00

ATOM 1026 C VAL A 141 -17.916 23.279 15.305 1.00 1.00

ATOM 1027 O VAL A 141 -17.822 24.117 14.404 1.00 1.00

ATOM 1028 CB VAL A 141 -15.578 22.410 15.664 1.00 1.00

ATOM 1029 CG1 VAL A 141 -15.505 23.043 17.049 1.00 1.00

ATOM 1030 CG2 VAL A 141 -14.665 21.195 15.594 1.00 1.00

ATOM 1031 N ASN A 142 -18.760 23.390 16.332 1.00 1.00

ATOM 1032 CA ASN A 142 -19.681 24.519 16.504 1.00 1.00

ATOM 1033 C ASN A 142 -20.561 24.775 15.277 1.00 1.00

ATOM 1034 O ASN A 142 -20.731 25.919 14.845 1.00 1.00

ATOM 1035 CB ASN A 142 -18.922 25.793 16.910 1.00 1.00

ATOM 1036 CG ASN A 142 -18.133 25.620 18.195 1.00 1.00

ATOM 1037 OD1 ASN A 142 -18.637 25.081 19.181 1.00 1.00

ATOM 1038 ND2 ASN A 142 -16.890 26.085 18.192 1.00 1.00

ATOM 1039 N VAL A 143 -21.113 23.697 14.727 1.00 1.00

ATOM 1040 CA VAL A 143 -21.924 23.769 13.508 1.00 1.00

ATOM 1041 C VAL A 143 -23.428 23.658 13.785 1.00 1.00

ATOM 1042 O VAL A 143 -24.244 23.748 12.863 1.00 1.00

ATOM 1043 CB VAL A 143 -21.485 22.708 12.454 1.00 1.00

ATOM 1044 CG1 VAL A 143 -20.057 22.973 11.987 1.00 1.00

ATOM 1045 CG2 VAL A 143 -21.624 21.288 13.001 1.00 1.00

ATOM 1046 N GLY A 144 -23.785 23.466 15.054 1.00 1.00

ATOM 1047 CA GLY A 144 -25.175 23.346 15.457 1.00 1.00

ATOM 1048 C GLY A 144 -25.799 24.685 15.796 1.00 1.00

ATOM 1049 O GLY A 144 -26.886 24.743 16.374 1.00 1.00

ATOM 1050 OXT GLY A 144 -25.186 25.720 15.442 1.00 1.00

TER

ATOM 1051 N GLY B 1 1.469 -2.553 -11.497 1.00 1.00

ATOM 1052 CA GLY B 1 2.214 -2.505 -10.205 1.00 1.00

ATOM 1053 C GLY B 1 3.454 -1.629 -10.250 1.00 1.00

ATOM 1054 O GLY B 1 3.822 -1.010 -9.249 1.00 1.00

ATOM 1055 N ASP B 2 4.093 -1.581 -11.418 1.00 1.00

ATOM 1056 CA ASP B 2 5.325 -0.820 -11.620 1.00 1.00

ATOM 1057 C ASP B 2 5.025 0.669 -11.819 1.00 1.00

ATOM 1058 O ASP B 2 5.047 1.174 -12.944 1.00 1.00

ATOM 1059 CB ASP B 2 6.101 -1.393 -12.814 1.00 1.00

ATOM 1060 CG ASP B 2 7.520 -0.853 -12.918 1.00 1.00

ATOM 1061 OD1 ASP B 2 8.028 -0.263 -11.937 1.00 1.00

ATOM 1062 OD2 ASP B 2 8.209 -0.977 -13.950 1.00 1.00

ATOM 1063 N VAL B 3 4.758 1.358 -10.711 1.00 1.00

ATOM 1064 CA VAL B 3 4.333 2.761 -10.722 1.00 1.00

ATOM 1065 C VAL B 3 5.306 3.690 -11.460 1.00 1.00

ATOM 1066 O VAL B 3 4.906 4.377 -12.401 1.00 1.00

ATOM 1067 CB VAL B 3 4.063 3.290 -9.280 1.00 1.00

ATOM 1068 CG1 VAL B 3 3.623 4.747 -9.304 1.00 1.00

ATOM 1069 CG2 VAL B 3 3.011 2.435 -8.584 1.00 1.00

ATOM 1070 N ASN B 4 6.572 3.702 -11.044 1.00 1.00

ATOM 1071 CA ASN B 4 7.570 4.584 -11.654 1.00 1.00

ATOM 1072 C ASN B 4 8.099 4.099 -13.008 1.00 1.00

ATOM 1073 O ASN B 4 8.706 4.869 -13.755 1.00 1.00

ATOM 1074 CB ASN B 4 8.730 4.881 -10.691 1.00 1.00

ATOM 1075 CG ASN B 4 9.326 3.627 -10.070 1.00 1.00

ATOM 1076 OD1 ASN B 4 9.430 2.581 -10.714 1.00 1.00

ATOM 1077 ND2 ASN B 4 9.725 3.733 -8.809 1.00 1.00

ATOM 1078 N GLY B 5 7.866 2.824 -13.310 1.00 1.00

ATOM 1079 CA GLY B 5 8.232 2.244 -14.591 1.00 1.00

ATOM 1080 C GLY B 5 9.713 1.960 -14.779 1.00 1.00

ATOM 1081 O GLY B 5 10.217 2.041 -15.901 1.00 1.00

ATOM 1082 N ASP B 6 10.408 1.618 -13.694 1.00 1.00

ATOM 1083 CA ASP B 6 11.841 1.327 -13.762 1.00 1.00

ATOM 1084 C ASP B 6 12.163 -0.139 -14.070 1.00 1.00

ATOM 1085 O ASP B 6 13.331 -0.505 -14.223 1.00 1.00

ATOM 1086 CB ASP B 6 12.576 1.813 -12.496 1.00 1.00

ATOM 1087 CG ASP B 6 12.130 1.094 -11.226 1.00 1.00

ATOM 1088 OD1 ASP B 6 11.326 0.139 -11.298 1.00 1.00

ATOM 1089 OD2 ASP B 6 12.536 1.425 -10.092 1.00 1.00

ATOM 1090 N GLY B 7 11.126 -0.970 -14.158 1.00 1.00

ATOM 1091 CA GLY B 7 11.276 -2.370 -14.524 1.00 1.00

ATOM 1092 C GLY B 7 11.162 -3.350 -13.369 1.00 1.00

ATOM 1093 O GLY B 7 11.145 -4.567 -13.583 1.00 1.00

ATOM 1094 N THR B 8 11.083 -2.823 -12.149 1.00 1.00

ATOM 1095 CA THR B 8 10.992 -3.647 -10.948 1.00 1.00

ATOM 1096 C THR B 8 9.838 -3.185 -10.062 1.00 1.00

ATOM 1097 O THR B 8 9.636 -1.983 -9.872 1.00 1.00

ATOM 1098 CB THR B 8 12.320 -3.599 -10.152 1.00 1.00

ATOM 1099 OG1 THR B 8 13.430 -3.721 -11.051 1.00 1.00

ATOM 1100 CG2 THR B 8 12.460 -4.826 -9.255 1.00 1.00

ATOM 1101 N ILE B 9 9.085 -4.142 -9.530 1.00 1.00

ATOM 1102 CA ILE B 9 8.012 -3.843 -8.584 1.00 1.00

ATOM 1103 C ILE B 9 8.520 -4.052 -7.158 1.00 1.00

ATOM 1104 O ILE B 9 8.761 -5.184 -6.732 1.00 1.00

ATOM 1105 CB ILE B 9 6.750 -4.701 -8.867 1.00 1.00

ATOM 1106 CG1 ILE B 9 6.267 -4.498 -10.308 1.00 1.00

ATOM 1107 CG2 ILE B 9 5.631 -4.353 -7.888 1.00 1.00

ATOM 1108 CD1 ILE B 9 5.642 -5.732 -10.936 1.00 1.00

ATOM 1109 N ASN B 10 8.693 -2.946 -6.435 1.00 1.00

ATOM 1110 CA ASN B 10 9.181 -2.980 -5.057 1.00 1.00

ATOM 1111 C ASN B 10 8.486 -1.962 -4.147 1.00 1.00

ATOM 1112 O ASN B 10 7.456 -1.392 -4.516 1.00 1.00

ATOM 1113 CB ASN B 10 10.711 -2.814 -5.009 1.00 1.00

ATOM 1114 CG ASN B 10 11.210 -1.619 -5.813 1.00 1.00

ATOM 1115 OD1 ASN B 10 10.558 -0.576 -5.882 1.00 1.00

ATOM 1116 ND2 ASN B 10 12.380 -1.770 -6.421 1.00 1.00

ATOM 1117 N SER B 11 9.058 -1.743 -2.963 1.00 1.00

ATOM 1118 CA SER B 11 8.487 -0.854 -1.948 1.00 1.00

ATOM 1119 C SER B 11 8.354 0.603 -2.405 1.00 1.00

ATOM 1120 O SER B 11 7.458 1.319 -1.950 1.00 1.00

ATOM 1121 CB SER B 11 9.305 -0.927 -0.657 1.00 1.00

ATOM 1122 OG SER B 11 10.648 -0.538 -0.885 1.00 1.00

ATOM 1123 N THR B 12 9.245 1.026 -3.302 1.00 1.00

ATOM 1124 CA THR B 12 9.226 2.381 -3.860 1.00 1.00

ATOM 1125 C THR B 12 7.933 2.666 -4.635 1.00 1.00

ATOM 1126 O THR B 12 7.433 3.793 -4.622 1.00 1.00

ATOM 1127 CB THR B 12 10.464 2.615 -4.761 1.00 1.00

ATOM 1128 OG1 THR B 12 11.654 2.244 -4.051 1.00 1.00

ATOM 1129 CG2 THR B 12 10.676 4.103 -5.033 1.00 1.00

ATOM 1130 N ASP B 13 7.406 1.641 -5.303 1.00 1.00

ATOM 1131 CA ASP B 13 6.145 1.751 -6.040 1.00 1.00

ATOM 1132 C ASP B 13 4.955 1.931 -5.106 1.00 1.00

ATOM 1133 O ASP B 13 4.091 2.774 -5.353 1.00 1.00

ATOM 1134 CB ASP B 13 5.925 0.524 -6.927 1.00 1.00

ATOM 1135 CG ASP B 13 6.930 0.434 -8.052 1.00 1.00

ATOM 1136 OD1 ASP B 13 6.933 1.321 -8.935 1.00 1.00

ATOM 1137 OD2 ASP B 13 7.768 -0.490 -8.140 1.00 1.00

ATOM 1138 N LEU B 14 4.917 1.133 -4.040 1.00 1.00

ATOM 1139 CA LEU B 14 3.846 1.206 -3.050 1.00 1.00

ATOM 1140 C LEU B 14 3.832 2.561 -2.342 1.00 1.00

ATOM 1141 O LEU B 14 2.766 3.141 -2.135 1.00 1.00

ATOM 1142 CB LEU B 14 3.954 0.059 -2.035 1.00 1.00

ATOM 1143 CG LEU B 14 2.844 -0.085 -0.985 1.00 1.00

ATOM 1144 CD1 LEU B 14 1.503 -0.459 -1.616 1.00 1.00

ATOM 1145 CD2 LEU B 14 3.239 -1.102 0.080 1.00 1.00

ATOM 1146 N THR B 15 5.019 3.054 -1.989 1.00 1.00

ATOM 1147 CA THR B 15 5.175 4.364 -1.357 1.00 1.00

ATOM 1148 C THR B 15 4.638 5.486 -2.249 1.00 1.00

ATOM 1149 O THR B 15 3.935 6.375 -1.771 1.00 1.00

ATOM 1150 CB THR B 15 6.657 4.615 -0.990 1.00 1.00

ATOM 1151 OG1 THR B 15 7.117 3.574 -0.122 1.00 1.00

ATOM 1152 CG2 THR B 15 6.803 5.869 -0.127 1.00 1.00

ATOM 1153 N MET B 16 4.969 5.428 -3.538 1.00 1.00

ATOM 1154 CA MET B 16 4.485 6.402 -4.517 1.00 1.00

ATOM 1155 C MET B 16 2.968 6.328 -4.685 1.00 1.00

ATOM 1156 O MET B 16 2.300 7.357 -4.792 1.00 1.00

ATOM 1157 CB MET B 16 5.163 6.186 -5.871 1.00 1.00

ATOM 1158 CG MET B 16 6.501 6.887 -6.018 1.00 1.00

ATOM 1159 SD MET B 16 7.057 6.916 -7.733 1.00 1.00

ATOM 1160 CE MET B 16 8.830 6.803 -7.498 1.00 1.00

ATOM 1161 N LEU B 17 2.439 5.105 -4.705 1.00 1.00

ATOM 1162 CA LEU B 17 1.005 4.870 -4.848 1.00 1.00

ATOM 1163 C LEU B 17 0.220 5.376 -3.635 1.00 1.00

ATOM 1164 O LEU B 17 -0.810 6.034 -3.792 1.00 1.00

ATOM 1165 CB LEU B 17 0.722 3.379 -5.095 1.00 1.00

ATOM 1166 CG LEU B 17 -0.734 2.895 -5.153 1.00 1.00

ATOM 1167 CD1 LEU B 17 -1.489 3.517 -6.319 1.00 1.00

ATOM 1168 CD2 LEU B 17 -0.796 1.379 -5.234 1.00 1.00

ATOM 1169 N LYS B 18 0.719 5.067 -2.440 1.00 1.00

ATOM 1170 CA LYS B 18 0.078 5.482 -1.192 1.00 1.00

ATOM 1171 C LYS B 18 0.088 7.003 -1.026 1.00 1.00

ATOM 1172 O LYS B 18 -0.906 7.589 -0.593 1.00 1.00

ATOM 1173 CB LYS B 18 0.752 4.812 0.010 1.00 1.00

ATOM 1174 CG LYS B 18 0.330 3.361 0.227 1.00 1.00

ATOM 1175 CD LYS B 18 1.168 2.684 1.301 1.00 1.00

ATOM 1176 CE LYS B 18 0.413 2.593 2.617 1.00 1.00

ATOM 1177 NZ LYS B 18 1.189 1.861 3.662 1.00 1.00

ATOM 1178 N ARG B 19 1.209 7.630 -1.380 1.00 1.00

ATOM 1179 CA ARG B 19 1.345 9.085 -1.324 1.00 1.00

ATOM 1180 C ARG B 19 0.444 9.776 -2.348 1.00 1.00

ATOM 1181 O ARG B 19 -0.057 10.873 -2.099 1.00 1.00

ATOM 1182 CB ARG B 19 2.806 9.502 -1.524 1.00 1.00

ATOM 1183 CG ARG B 19 3.659 9.368 -0.266 1.00 1.00

ATOM 1184 CD ARG B 19 5.163 9.563 -0.485 1.00 1.00

ATOM 1185 NE ARG B 19 5.502 10.896 -0.990 1.00 1.00

ATOM 1186 CZ ARG B 19 5.483 12.017 -0.269 1.00 1.00

ATOM 1187 NH1 ARG B 19 5.137 11.995 1.014 1.00 1.00

ATOM 1188 NH2 ARG B 19 5.812 13.169 -0.835 1.00 1.00

ATOM 1189 N SER B 20 0.239 9.117 -3.489 1.00 1.00

ATOM 1190 CA SER B 20 -0.667 9.603 -4.531 1.00 1.00

ATOM 1191 C SER B 20 -2.125 9.614 -4.065 1.00 1.00

ATOM 1192 O SER B 20 -2.843 10.591 -4.291 1.00 1.00

ATOM 1193 CB SER B 20 -0.519 8.760 -5.805 1.00 1.00

ATOM 1194 OG SER B 20 -1.615 8.954 -6.683 1.00 1.00

ATOM 1195 N VAL B 21 -2.549 8.524 -3.427 1.00 1.00

ATOM 1196 CA VAL B 21 -3.907 8.407 -2.885 1.00 1.00

ATOM 1197 C VAL B 21 -4.119 9.413 -1.747 1.00 1.00

ATOM 1198 O VAL B 21 -5.192 10.007 -1.624 1.00 1.00

ATOM 1199 CB VAL B 21 -4.218 6.961 -2.410 1.00 1.00

ATOM 1200 CG1 VAL B 21 -5.623 6.860 -1.819 1.00 1.00

ATOM 1201 CG2 VAL B 21 -4.066 5.971 -3.559 1.00 1.00

ATOM 1202 N LEU B 22 -3.081 9.615 -0.937 1.00 1.00

ATOM 1203 CA LEU B 22 -3.114 10.609 0.134 1.00 1.00

ATOM 1204 C LEU B 22 -2.924 12.037 -0.388 1.00 1.00

ATOM 1205 O LEU B 22 -2.911 12.994 0.391 1.00 1.00

ATOM 1206 CB LEU B 22 -2.074 10.277 1.212 1.00 1.00

ATOM 1207 CG LEU B 22 -2.445 9.139 2.170 1.00 1.00

ATOM 1208 CD1 LEU B 22 -1.199 8.516 2.778 1.00 1.00

ATOM 1209 CD2 LEU B 22 -3.390 9.622 3.263 1.00 1.00

ATOM 1210 N ARG B 23 -2.781 12.163 -1.709 1.00 1.00

ATOM 1211 CA ARG B 23 -2.681 13.452 -2.410 1.00 1.00

ATOM 1212 C ARG B 23 -1.427 14.264 -2.056 1.00 1.00

ATOM 1213 O ARG B 23 -1.411 15.489 -2.205 1.00 1.00

ATOM 1214 CB ARG B 23 -3.957 14.296 -2.221 1.00 1.00

ATOM 1215 CG ARG B 23 -5.262 13.555 -2.502 1.00 1.00

ATOM 1216 CD ARG B 23 -5.913 13.918 -3.823 1.00 1.00

ATOM 1217 NE ARG B 23 -7.170 13.201 -4.035 1.00 1.00

ATOM 1218 CZ ARG B 23 -8.339 13.786 -4.280 1.00 1.00

ATOM 1219 NH1 ARG B 23 -8.430 15.109 -4.342 1.00 1.00

ATOM 1220 NH2 ARG B 23 -9.425 13.045 -4.460 1.00 1.00

ATOM 1221 N ALA B 24 -0.385 13.579 -1.592 1.00 1.00

ATOM 1222 CA ALA B 24 0.895 14.220 -1.298 1.00 1.00

ATOM 1223 C ALA B 24 1.655 14.521 -2.584 1.00 1.00

ATOM 1224 O ALA B 24 2.342 15.537 -2.691 1.00 1.00

ATOM 1225 CB ALA B 24 1.731 13.347 -0.371 1.00 1.00

ATOM 1226 N ILE B 25 1.518 13.625 -3.559 1.00 1.00

ATOM 1227 CA ILE B 25 2.177 13.759 -4.854 1.00 1.00

ATOM 1228 C ILE B 25 1.179 13.585 -5.994 1.00 1.00

ATOM 1229 O ILE B 25 0.068 13.085 -5.794 1.00 1.00

ATOM 1230 CB ILE B 25 3.333 12.724 -5.001 1.00 1.00

ATOM 1231 CG1 ILE B 25 2.809 11.287 -4.903 1.00 1.00

ATOM 1232 CG2 ILE B 25 4.438 12.980 -3.976 1.00 1.00

ATOM 1233 CD1 ILE B 25 3.423 10.340 -5.916 1.00 1.00

ATOM 1234 N THR B 26 1.581 14.011 -7.187 1.00 1.00

ATOM 1235 CA THR B 26 0.848 13.682 -8.404 1.00 1.00

ATOM 1236 C THR B 26 1.665 12.685 -9.213 1.00 1.00

ATOM 1237 O THR B 26 2.900 12.711 -9.190 1.00 1.00

ATOM 1238 CB THR B 26 0.553 14.936 -9.254 1.00 1.00

ATOM 1239 OG1 THR B 26 1.772 15.637 -9.524 1.00 1.00

ATOM 1240 CG2 THR B 26 -0.284 15.946 -8.469 1.00 1.00

ATOM 1241 N LEU B 27 0.969 11.799 -9.913 1.00 1.00

ATOM 1242 CA LEU B 27 1.621 10.874 -10.827 1.00 1.00

ATOM 1243 C LEU B 27 1.461 11.373 -12.256 1.00 1.00

ATOM 1244 O LEU B 27 0.460 12.014 -12.589 1.00 1.00

ATOM 1245 CB LEU B 27 1.039 9.466 -10.686 1.00 1.00

ATOM 1246 CG LEU B 27 1.189 8.761 -9.333 1.00 1.00

ATOM 1247 CD1 LEU B 27 0.281 7.545 -9.277 1.00 1.00

ATOM 1248 CD2 LEU B 27 2.636 8.365 -9.059 1.00 1.00

ATOM 1249 N THR B 28 2.456 11.086 -13.090 1.00 1.00

ATOM 1250 CA THR B 28 2.384 11.406 -14.513 1.00 1.00

ATOM 1251 C THR B 28 1.353 10.499 -15.181 1.00 1.00

ATOM 1252 O THR B 28 0.953 9.481 -14.610 1.00 1.00

ATOM 1253 CB THR B 28 3.763 11.257 -15.188 1.00 1.00

ATOM 1254 OG1 THR B 28 4.217 9.902 -15.073 1.00 1.00

ATOM 1255 CG2 THR B 28 4.826 12.057 -14.437 1.00 1.00

ATOM 1256 N ASP B 29 0.926 10.874 -16.383 1.00 1.00

ATOM 1257 CA ASP B 29 -0.131 10.148 -17.090 1.00 1.00

ATOM 1258 C ASP B 29 0.221 8.689 -17.377 1.00 1.00

ATOM 1259 O ASP B 29 -0.645 7.819 -17.296 1.00 1.00

ATOM 1260 CB ASP B 29 -0.535 10.890 -18.368 1.00 1.00

ATOM 1261 CG ASP B 29 -1.193 12.233 -18.080 1.00 1.00

ATOM 1262 OD1 ASP B 29 -1.850 12.367 -17.026 1.00 1.00

ATOM 1263 OD2 ASP B 29 -1.107 13.216 -18.847 1.00 1.00

ATOM 1264 N ASP B 30 1.491 8.427 -17.691 1.00 1.00

ATOM 1265 CA ASP B 30 1.983 7.059 -17.873 1.00 1.00

ATOM 1266 C ASP B 30 1.934 6.272 -16.567 1.00 1.00

ATOM 1267 O ASP B 30 1.493 5.119 -16.546 1.00 1.00

ATOM 1268 CB ASP B 30 3.423 7.062 -18.391 1.00 1.00

ATOM 1269 CG ASP B 30 3.533 7.487 -19.842 1.00 1.00

ATOM 1270 OD1 ASP B 30 2.500 7.735 -20.500 1.00 1.00

ATOM 1271 OD2 ASP B 30 4.638 7.608 -20.404 1.00 1.00

ATOM 1272 N ALA B 31 2.403 6.903 -15.492 1.00 1.00

ATOM 1273 CA ALA B 31 2.444 6.287 -14.165 1.00 1.00

ATOM 1274 C ALA B 31 1.047 5.999 -13.624 1.00 1.00

ATOM 1275 O ALA B 31 0.845 5.010 -12.918 1.00 1.00

ATOM 1276 CB ALA B 31 3.220 7.168 -13.195 1.00 1.00

ATOM 1277 N LYS B 32 0.092 6.864 -13.966 1.00 1.00

ATOM 1278 CA LYS B 32 -1.305 6.711 -13.560 1.00 1.00

ATOM 1279 C LYS B 32 -1.909 5.387 -14.032 1.00 1.00

ATOM 1280 O LYS B 32 -2.629 4.725 -13.279 1.00 1.00

ATOM 1281 CB LYS B 32 -2.142 7.883 -14.083 1.00 1.00

ATOM 1282 CG LYS B 32 -2.111 9.121 -13.201 1.00 1.00

ATOM 1283 CD LYS B 32 -3.170 10.124 -13.640 1.00 1.00

ATOM 1284 CE LYS B 32 -3.023 11.440 -12.896 1.00 1.00

ATOM 1285 NZ LYS B 32 -3.042 12.602 -13.830 1.00 1.00

ATOM 1286 N ALA B 33 -1.611 5.012 -15.274 1.00 1.00

ATOM 1287 CA ALA B 33 -2.099 3.758 -15.849 1.00 1.00

ATOM 1288 C ALA B 33 -1.502 2.540 -15.145 1.00 1.00

ATOM 1289 O ALA B 33 -2.198 1.551 -14.906 1.00 1.00

ATOM 1290 CB ALA B 33 -1.810 3.710 -17.347 1.00 1.00

ATOM 1291 N ARG B 34 -0.215 2.625 -14.807 1.00 1.00

ATOM 1292 CA ARG B 34 0.493 1.541 -14.127 1.00 1.00

ATOM 1293 C ARG B 34 0.120 1.442 -12.644 1.00 1.00

ATOM 1294 O ARG B 34 0.188 0.360 -12.054 1.00 1.00

ATOM 1295 CB ARG B 34 2.010 1.701 -14.286 1.00 1.00

ATOM 1296 CG ARG B 34 2.501 1.704 -15.735 1.00 1.00

ATOM 1297 CD ARG B 34 4.015 1.806 -15.896 1.00 1.00

ATOM 1298 NE ARG B 34 4.568 2.985 -15.227 1.00 1.00

ATOM 1299 CZ ARG B 34 5.129 4.016 -15.852 1.00 1.00

ATOM 1300 NH1 ARG B 34 5.224 4.032 -17.177 1.00 1.00

ATOM 1301 NH2 ARG B 34 5.600 5.037 -15.150 1.00 1.00

ATOM 1302 N ALA B 35 -0.265 2.570 -12.052 1.00 1.00

ATOM 1303 CA ALA B 35 -0.716 2.614 -10.660 1.00 1.00

ATOM 1304 C ALA B 35 -2.133 2.059 -10.501 1.00 1.00

ATOM 1305 O ALA B 35 -2.518 1.619 -9.415 1.00 1.00

ATOM 1306 CB ALA B 35 -0.642 4.034 -10.121 1.00 1.00

ATOM 1307 N ASP B 36 -2.902 2.094 -11.587 1.00 1.00

ATOM 1308 CA ASP B 36 -4.253 1.542 -11.614 1.00 1.00

ATOM 1309 C ASP B 36 -4.184 0.013 -11.679 1.00 1.00

ATOM 1310 O ASP B 36 -4.362 -0.584 -12.743 1.00 1.00

ATOM 1311 CB ASP B 36 -5.028 2.118 -12.806 1.00 1.00

ATOM 1312 CG ASP B 36 -6.533 1.891 -12.710 1.00 1.00

ATOM 1313 OD1 ASP B 36 -7.005 1.247 -11.747 1.00 1.00

ATOM 1314 OD2 ASP B 36 -7.326 2.326 -13.567 1.00 1.00

ATOM 1315 N VAL B 37 -3.921 -0.604 -10.527 1.00 1.00

ATOM 1316 CA VAL B 37 -3.674 -2.047 -10.427 1.00 1.00

ATOM 1317 C VAL B 37 -4.850 -2.895 -10.931 1.00 1.00

ATOM 1318 O VAL B 37 -4.651 -3.831 -11.708 1.00 1.00

ATOM 1319 CB VAL B 37 -3.281 -2.466 -8.978 1.00 1.00

ATOM 1320 CG1 VAL B 37 -2.862 -3.936 -8.914 1.00 1.00

ATOM 1321 CG2 VAL B 37 -2.163 -1.578 -8.440 1.00 1.00

ATOM 1322 N ASP B 38 -6.063 -2.558 -10.496 1.00 1.00

ATOM 1323 CA ASP B 38 -7.255 -3.324 -10.879 1.00 1.00

ATOM 1324 C ASP B 38 -7.926 -2.816 -12.159 1.00 1.00

ATOM 1325 O ASP B 38 -8.966 -3.333 -12.572 1.00 1.00

ATOM 1326 CB ASP B 38 -8.257 -3.408 -9.715 1.00 1.00

ATOM 1327 CG ASP B 38 -8.930 -4.773 -9.600 1.00 1.00

ATOM 1328 OD1 ASP B 38 -8.383 -5.778 -10.106 1.00 1.00

ATOM 1329 OD2 ASP B 38 -10.020 -4.937 -9.011 1.00 1.00

ATOM 1330 N LYS B 39 -7.374 -1.799 -12.759 1.00 1.00

ATOM 1331 CA LYS B 39 -7.717 -1.190 -14.051 1.00 1.00

ATOM 1332 C LYS B 39 -9.213 -0.828 -14.102 1.00 1.00

ATOM 1333 O LYS B 39 -9.950 -1.259 -15.004 1.00 1.00

ATOM 1334 CB LYS B 39 -7.421 -2.177 -15.191 1.00 1.00

ATOM 1335 CG LYS B 39 -5.930 -2.456 -15.374 1.00 1.00

ATOM 1336 CD LYS B 39 -5.205 -1.333 -16.112 1.00 1.00

ATOM 1337 CE LYS B 39 -3.692 -1.360 -15.900 1.00 1.00

ATOM 1338 NZ LYS B 39 -2.982 -0.392 -16.746 1.00 1.00

ATOM 1339 N ASN B 40 -9.661 -0.031 -13.133 1.00 1.00

ATOM 1340 CA ASN B 40 -11.081 0.372 -13.093 1.00 1.00

ATOM 1341 C ASN B 40 -11.248 1.889 -13.378 1.00 1.00

ATOM 1342 O ASN B 40 -12.346 2.447 -13.248 1.00 1.00

ATOM 1343 CB ASN B 40 -11.726 0.019 -11.741 1.00 1.00

ATOM 1344 CG ASN B 40 -11.184 0.780 -10.536 1.00 1.00

ATOM 1345 OD1 ASN B 40 -10.125 1.396 -10.620 1.00 1.00

ATOM 1346 ND2 ASN B 40 -11.867 0.765 -9.406 1.00 1.00

ATOM 1347 N GLY B 41 -10.149 2.525 -13.770 1.00 1.00

ATOM 1348 CA GLY B 41 -10.149 3.962 -14.163 1.00 1.00

ATOM 1349 C GLY B 41 -9.879 4.943 -13.010 1.00 1.00

ATOM 1350 O GLY B 41 -9.905 6.168 -13.206 1.00 1.00

ATOM 1351 N SER B 42 -9.624 4.408 -11.836 1.00 1.00

ATOM 1352 CA SER B 42 -9.347 5.238 -10.640 1.00 1.00

ATOM 1353 C SER B 42 -8.186 4.691 -9.844 1.00 1.00

ATOM 1354 O SER B 42 -7.961 3.481 -9.800 1.00 1.00

ATOM 1355 CB SER B 42 -10.543 5.251 -9.706 1.00 1.00

ATOM 1356 OG SER B 42 -11.601 6.048 -10.224 1.00 1.00

ATOM 1357 N ILE B 43 -7.465 5.600 -9.219 1.00 1.00

ATOM 1358 CA ILE B 43 -6.325 5.229 -8.378 1.00 1.00

ATOM 1359 C ILE B 43 -6.664 5.506 -6.909 1.00 1.00

ATOM 1360 O ILE B 43 -6.910 6.653 -6.509 1.00 1.00

ATOM 1361 CB ILE B 43 -5.076 6.018 -8.766 1.00 1.00

ATOM 1362 CG1 ILE B 43 -4.526 5.610 -10.137 1.00 1.00

ATOM 1363 CG2 ILE B 43 -3.926 5.823 -7.771 1.00 1.00

ATOM 1364 CD1 ILE B 43 -4.215 6.800 -11.046 1.00 1.00

ATOM 1365 N ASN B 44 -6.864 4.408 -6.167 1.00 1.00

ATOM 1366 CA ASN B 44 -7.379 4.469 -4.799 1.00 1.00

ATOM 1367 C ASN B 44 -6.844 3.377 -3.861 1.00 1.00

ATOM 1368 O ASN B 44 -5.885 2.675 -4.196 1.00 1.00

ATOM 1369 CB ASN B 44 -8.921 4.491 -4.806 1.00 1.00

ATOM 1370 CG ASN B 44 -9.537 3.254 -5.456 1.00 1.00

ATOM 1371 OD1 ASN B 44 -9.050 2.137 -5.288 1.00 1.00

ATOM 1372 ND2 ASN B 44 -10.624 3.453 -6.186 1.00 1.00

ATOM 1373 N SER B 45 -7.478 3.245 -2.696 1.00 1.00

ATOM 1374 CA SER B 45 -7.060 2.305 -1.649 1.00 1.00

ATOM 1375 C SER B 45 -7.169 0.828 -2.041 1.00 1.00

ATOM 1376 O SER B 45 -6.505 -0.022 -1.439 1.00 1.00

ATOM 1377 CB SER B 45 -7.846 2.558 -0.357 1.00 1.00

ATOM 1378 OG SER B 45 -9.240 2.432 -0.570 1.00 1.00

ATOM 1379 N THR B 46 -8.009 0.521 -3.028 1.00 1.00

ATOM 1380 CA THR B 46 -8.127 -0.846 -3.540 1.00 1.00

ATOM 1381 C THR B 46 -6.849 -1.256 -4.275 1.00 1.00

ATOM 1382 O THR B 46 -6.384 -2.389 -4.125 1.00 1.00

ATOM 1383 CB THR B 46 -9.374 -1.001 -4.443 1.00 1.00

ATOM 1384 OG1 THR B 46 -10.556 -0.744 -3.676 1.00 1.00

ATOM 1385 CG2 THR B 46 -9.558 -2.456 -4.874 1.00 1.00

ATOM 1386 N ASP B 47 -6.287 -0.330 -5.054 1.00 1.00

ATOM 1387 CA ASP B 47 -4.992 -0.531 -5.707 1.00 1.00

ATOM 1388 C ASP B 47 -3.876 -0.692 -4.680 1.00 1.00

ATOM 1389 O ASP B 47 -2.986 -1.529 -4.846 1.00 1.00

ATOM 1390 CB ASP B 47 -4.652 0.642 -6.631 1.00 1.00

ATOM 1391 CG ASP B 47 -5.722 0.900 -7.667 1.00 1.00

ATOM 1392 OD1 ASP B 47 -6.011 -0.007 -8.478 1.00 1.00

ATOM 1393 OD2 ASP B 47 -6.334 1.987 -7.752 1.00 1.00

ATOM 1394 N VAL B 48 -3.937 0.123 -3.625 1.00 1.00

ATOM 1395 CA VAL B 48 -2.979 0.074 -2.521 1.00 1.00

ATOM 1396 C VAL B 48 -2.974 -1.312 -1.870 1.00 1.00

ATOM 1397 O VAL B 48 -1.912 -1.912 -1.692 1.00 1.00

ATOM 1398 CB VAL B 48 -3.275 1.174 -1.460 1.00 1.00

ATOM 1399 CG1 VAL B 48 -2.434 0.970 -0.203 1.00 1.00

ATOM 1400 CG2 VAL B 48 -3.034 2.563 -2.040 1.00 1.00

ATOM 1401 N LEU B 49 -4.162 -1.812 -1.539 1.00 1.00

ATOM 1402 CA LEU B 49 -4.316 -3.128 -0.922 1.00 1.00

ATOM 1403 C LEU B 49 -3.737 -4.241 -1.800 1.00 1.00

ATOM 1404 O LEU B 49 -3.001 -5.101 -1.311 1.00 1.00

ATOM 1405 CB LEU B 49 -5.789 -3.407 -0.602 1.00 1.00

ATOM 1406 CG LEU B 49 -6.116 -4.628 0.266 1.00 1.00

ATOM 1407 CD1 LEU B 49 -5.980 -4.306 1.747 1.00 1.00

ATOM 1408 CD2 LEU B 49 -7.517 -5.141 -0.041 1.00 1.00

ATOM 1409 N LEU B 50 -4.062 -4.202 -3.089 1.00 1.00

ATOM 1410 CA LEU B 50 -3.622 -5.224 -4.040 1.00 1.00

ATOM 1411 C LEU B 50 -2.106 -5.241 -4.243 1.00 1.00

ATOM 1412 O LEU B 50 -1.497 -6.314 -4.276 1.00 1.00

ATOM 1413 CB LEU B 50 -4.340 -5.064 -5.384 1.00 1.00

ATOM 1414 CG LEU B 50 -5.825 -5.441 -5.427 1.00 1.00

ATOM 1415 CD1 LEU B 50 -6.459 -4.953 -6.720 1.00 1.00

ATOM 1416 CD2 LEU B 50 -6.035 -6.946 -5.257 1.00 1.00

ATOM 1417 N LEU B 51 -1.504 -4.060 -4.371 1.00 1.00

ATOM 1418 CA LEU B 51 -0.055 -3.948 -4.544 1.00 1.00

ATOM 1419 C LEU B 51 0.712 -4.379 -3.289 1.00 1.00

ATOM 1420 O LEU B 51 1.734 -5.061 -3.391 1.00 1.00

ATOM 1421 CB LEU B 51 0.350 -2.528 -4.973 1.00 1.00

ATOM 1422 CG LEU B 51 1.833 -2.242 -5.257 1.00 1.00

ATOM 1423 CD1 LEU B 51 2.429 -3.224 -6.265 1.00 1.00

ATOM 1424 CD2 LEU B 51 2.032 -0.814 -5.733 1.00 1.00

ATOM 1425 N SER B 52 0.216 -3.978 -2.118 1.00 1.00

ATOM 1426 CA SER B 52 0.840 -4.342 -0.846 1.00 1.00

ATOM 1427 C SER B 52 0.806 -5.852 -0.598 1.00 1.00

ATOM 1428 O SER B 52 1.757 -6.418 -0.058 1.00 1.00

ATOM 1429 CB SER B 52 0.191 -3.590 0.322 1.00 1.00

ATOM 1430 OG SER B 52 -1.146 -4.010 0.534 1.00 1.00

ATOM 1431 N ARG B 53 -0.293 -6.488 -1.004 1.00 1.00

ATOM 1432 CA ARG B 53 -0.442 -7.940 -0.910 1.00 1.00

ATOM 1433 C ARG B 53 0.484 -8.668 -1.885 1.00 1.00

ATOM 1434 O ARG B 53 1.006 -9.739 -1.569 1.00 1.00

ATOM 1435 CB ARG B 53 -1.897 -8.351 -1.153 1.00 1.00

ATOM 1436 CG ARG B 53 -2.858 -7.847 -0.089 1.00 1.00

ATOM 1437 CD ARG B 53 -4.269 -8.396 -0.205 1.00 1.00

ATOM 1438 NE ARG B 53 -4.371 -9.745 0.353 1.00 1.00

ATOM 1439 CZ ARG B 53 -5.184 -10.692 -0.102 1.00 1.00

ATOM 1440 NH1 ARG B 53 -5.987 -10.457 -1.134 1.00 1.00

ATOM 1441 NH2 ARG B 53 -5.197 -11.883 0.479 1.00 1.00

ATOM 1442 N TYR B 54 0.682 -8.078 -3.064 1.00 1.00

ATOM 1443 CA TYR B 54 1.584 -8.624 -4.077 1.00 1.00

ATOM 1444 C TYR B 54 3.031 -8.651 -3.579 1.00 1.00

ATOM 1445 O TYR B 54 3.756 -9.620 -3.813 1.00 1.00

ATOM 1446 CB TYR B 54 1.480 -7.825 -5.384 1.00 1.00

ATOM 1447 CG TYR B 54 2.460 -8.254 -6.458 1.00 1.00

ATOM 1448 CD1 TYR B 54 2.187 -9.341 -7.287 1.00 1.00

ATOM 1449 CD2 TYR B 54 3.665 -7.569 -6.644 1.00 1.00

ATOM 1450 CE1 TYR B 54 3.087 -9.739 -8.273 1.00 1.00

ATOM 1451 CE2 TYR B 54 4.571 -7.959 -7.626 1.00 1.00

ATOM 1452 CZ TYR B 54 4.274 -9.043 -8.436 1.00 1.00

ATOM 1453 OH TYR B 54 5.167 -9.433 -9.410 1.00 1.00

ATOM 1454 N LEU B 55 3.438 -7.585 -2.893 1.00 1.00

ATOM 1455 CA LEU B 55 4.793 -7.472 -2.357 1.00 1.00

ATOM 1456 C LEU B 55 5.006 -8.358 -1.129 1.00 1.00

ATOM 1457 O LEU B 55 6.132 -8.769 -0.842 1.00 1.00

ATOM 1458 CB LEU B 55 5.127 -6.013 -2.026 1.00 1.00

ATOM 1459 CG LEU B 55 5.140 -5.002 -3.178 1.00 1.00

ATOM 1460 CD1 LEU B 55 5.093 -3.579 -2.643 1.00 1.00

ATOM 1461 CD2 LEU B 55 6.351 -5.196 -4.083 1.00 1.00

ATOM 1462 N LEU B 56 3.921 -8.650 -0.414 1.00 1.00

ATOM 1463 CA LEU B 56 3.974 -9.493 0.779 1.00 1.00

ATOM 1464 C LEU B 56 3.684 -10.955 0.442 1.00 1.00

ATOM 1465 O LEU B 56 4.268 -11.524 -0.482 1.00 1.00

ATOM 1466 CB LEU B 56 2.989 -8.990 1.836 1.00 1.00

ATOM 1467 CG LEU B 56 3.526 -7.952 2.823 1.00 1.00

ATOM 1468 CD1 LEU B 56 4.704 -8.512 3.605 1.00 1.00

ATOM 1469 CD2 LEU B 56 3.993 -6.705 2.089 1.00 1.00

ATOM 1470 OXT LEU B 56 2.810 -11.547 1.119 1.00 1.00

ATOM 1471 CA CA C 160 9.197 0.279 -10.001 1.00 1.00

ATOM 1472 CA CA D 161 -7.996 1.263 -9.567 1.00 1.00

TER

ENDMDL

MODEL 2

ATOM 0 N GLY A 5 -25.852 0.602 2.639 1.00 1.00

ATOM 1 CA GLY A 5 -24.451 0.107 2.790 1.00 1.00

ATOM 2 C GLY A 5 -23.739 0.723 3.982 1.00 1.00

ATOM 3 O GLY A 5 -24.291 0.780 5.087 1.00 1.00

ATOM 4 N VAL A 6 -22.509 1.183 3.762 1.00 1.00

ATOM 5 CA VAL A 6 -21.710 1.797 4.825 1.00 1.00

ATOM 6 C VAL A 6 -22.110 3.262 5.034 1.00 1.00

ATOM 7 O VAL A 6 -22.338 3.994 4.072 1.00 1.00

ATOM 8 CB VAL A 6 -20.180 1.692 4.547 1.00 1.00

ATOM 9 CG1 VAL A 6 -19.371 2.339 5.668 1.00 1.00

ATOM 10 CG2 VAL A 6 -19.759 0.237 4.365 1.00 1.00

ATOM 11 N VAL A 7 -22.207 3.674 6.297 1.00 1.00

ATOM 12 CA VAL A 7 -22.502 5.062 6.636 1.00 1.00

ATOM 13 C VAL A 7 -21.323 5.650 7.406 1.00 1.00

ATOM 14 O VAL A 7 -20.906 5.114 8.430 1.00 1.00

ATOM 15 CB VAL A 7 -23.820 5.205 7.453 1.00 1.00

ATOM 16 CG1 VAL A 7 -24.054 6.664 7.860 1.00 1.00

ATOM 17 CG2 VAL A 7 -25.017 4.688 6.653 1.00 1.00

ATOM 18 N VAL A 8 -20.778 6.747 6.892 1.00 1.00

ATOM 19 CA VAL A 8 -19.680 7.436 7.559 1.00 1.00

ATOM 20 C VAL A 8 -20.211 8.748 8.139 1.00 1.00

ATOM 21 O VAL A 8 -20.645 9.633 7.395 1.00 1.00

ATOM 22 CB VAL A 8 -18.502 7.705 6.593 1.00 1.00

ATOM 23 CG1 VAL A 8 -17.332 8.347 7.328 1.00 1.00

ATOM 24 CG2 VAL A 8 -18.062 6.416 5.891 1.00 1.00

ATOM 25 N GLU A 9 -20.183 8.862 9.465 1.00 1.00

ATOM 26 CA GLU A 9 -20.681 10.061 10.127 1.00 1.00

ATOM 27 C GLU A 9 -19.566 10.845 10.807 1.00 1.00

ATOM 28 O GLU A 9 -18.832 10.305 11.637 1.00 1.00

ATOM 29 CB GLU A 9 -21.795 9.731 11.128 1.00 1.00

ATOM 30 CG GLU A 9 -22.374 10.973 11.812 1.00 1.00

ATOM 31 CD GLU A 9 -23.679 10.722 12.555 1.00 1.00

ATOM 32 OE1 GLU A 9 -24.263 9.619 12.432 1.00 1.00

ATOM 33 OE2 GLU A 9 -24.125 11.648 13.266 1.00 1.00

ATOM 34 N ILE A 10 -19.448 12.118 10.430 1.00 1.00

ATOM 35 CA ILE A 10 -18.557 13.059 11.101 1.00 1.00

ATOM 36 C ILE A 10 -19.280 13.611 12.331 1.00 1.00

ATOM 37 O ILE A 10 -20.375 14.163 12.220 1.00 1.00

ATOM 38 CB ILE A 10 -18.131 14.217 10.151 1.00 1.00

ATOM 39 CG1 ILE A 10 -17.372 13.661 8.938 1.00 1.00

ATOM 40 CG2 ILE A 10 -17.282 15.264 10.896 1.00 1.00

ATOM 41 CD1 ILE A 10 -17.525 14.487 7.668 1.00 1.00

ATOM 42 N GLY A 11 -18.665 13.438 13.498 1.00 1.00

ATOM 43 CA GLY A 11 -19.239 13.905 14.765 1.00 1.00

ATOM 44 C GLY A 11 -19.423 15.414 14.823 1.00 1.00

ATOM 45 O GLY A 11 -18.806 16.158 14.053 1.00 1.00

ATOM 46 N LYS A 12 -20.287 15.859 15.729 1.00 1.00

ATOM 47 CA LYS A 12 -20.515 17.283 15.951 1.00 1.00

ATOM 48 C LYS A 12 -20.124 17.627 17.379 1.00 1.00

ATOM 49 O LYS A 12 -20.629 17.028 18.328 1.00 1.00

ATOM 50 CB LYS A 12 -21.972 17.665 15.679 1.00 1.00

ATOM 51 CG LYS A 12 -22.398 17.517 14.222 1.00 1.00

ATOM 52 CD LYS A 12 -23.900 17.714 14.077 1.00 1.00

ATOM 53 CE LYS A 12 -24.371 17.459 12.652 1.00 1.00

ATOM 54 NZ LYS A 12 -24.175 16.040 12.233 1.00 1.00

ATOM 55 N VAL A 13 -19.197 18.573 17.521 1.00 1.00

ATOM 56 CA VAL A 13 -18.660 18.944 18.830 1.00 1.00

ATOM 57 C VAL A 13 -18.688 20.459 19.028 1.00 1.00

ATOM 58 O VAL A 13 -18.928 21.216 18.085 1.00 1.00

ATOM 59 CB VAL A 13 -17.206 18.413 19.052 1.00 1.00

ATOM 60 CG1 VAL A 13 -17.164 16.886 19.002 1.00 1.00

ATOM 61 CG2 VAL A 13 -16.240 19.016 18.037 1.00 1.00

ATOM 62 N THR A 14 -18.454 20.886 20.263 1.00 1.00

ATOM 63 CA THR A 14 -18.413 22.299 20.604 1.00 1.00

ATOM 64 C THR A 14 -17.088 22.604 21.300 1.00 1.00

ATOM 65 O THR A 14 -16.442 21.700 21.838 1.00 1.00

ATOM 66 CB THR A 14 -19.595 22.709 21.522 1.00 1.00

ATOM 67 OG1 THR A 14 -19.572 21.918 22.715 1.00 1.00

ATOM 68 CG2 THR A 14 -20.933 22.510 20.814 1.00 1.00

ATOM 69 N GLY A 15 -16.687 23.869 21.282 1.00 1.00

ATOM 70 CA GLY A 15 -15.458 24.294 21.947 1.00 1.00

ATOM 71 C GLY A 15 -15.190 25.774 21.783 1.00 1.00

ATOM 72 O GLY A 15 -15.469 26.354 20.730 1.00 1.00

ATOM 73 N SER A 16 -14.649 26.382 22.837 1.00 1.00

ATOM 74 CA SER A 16 -14.279 27.794 22.822 1.00 1.00

ATOM 75 C SER A 16 -13.024 28.039 21.986 1.00 1.00

ATOM 76 O SER A 16 -12.210 27.134 21.788 1.00 1.00

ATOM 77 CB SER A 16 -14.056 28.297 24.253 1.00 1.00

ATOM 78 OG SER A 16 -15.227 28.129 25.033 1.00 1.00

ATOM 79 N VAL A 17 -12.886 29.271 21.492 1.00 1.00

ATOM 80 CA VAL A 17 -11.693 29.701 20.764 1.00 1.00

ATOM 81 C VAL A 17 -10.432 29.445 21.591 1.00 1.00

ATOM 82 O VAL A 17 -10.373 29.786 22.777 1.00 1.00

ATOM 83 CB VAL A 17 -11.780 31.205 20.371 1.00 1.00

ATOM 84 CG1 VAL A 17 -10.460 31.712 19.793 1.00 1.00

ATOM 85 CG2 VAL A 17 -12.918 31.435 19.384 1.00 1.00

ATOM 86 N GLY A 18 -9.435 28.829 20.961 1.00 1.00

ATOM 87 CA GLY A 18 -8.157 28.551 21.612 1.00 1.00

ATOM 88 C GLY A 18 -8.031 27.170 22.223 1.00 1.00

ATOM 89 O GLY A 18 -6.936 26.771 22.621 1.00 1.00

ATOM 90 N THR A 19 -9.143 26.441 22.308 1.00 1.00

ATOM 91 CA THR A 19 -9.137 25.084 22.864 1.00 1.00

ATOM 92 C THR A 19 -8.964 24.024 21.773 1.00 1.00

ATOM 93 O THR A 19 -9.195 24.288 20.590 1.00 1.00

ATOM 94 CB THR A 19 -10.419 24.762 23.682 1.00 1.00

ATOM 95 OG1 THR A 19 -11.545 24.653 22.803 1.00 1.00

ATOM 96 CG2 THR A 19 -10.688 25.826 24.737 1.00 1.00

ATOM 97 N THR A 20 -8.552 22.829 22.188 1.00 1.00

ATOM 98 CA THR A 20 -8.439 21.692 21.284 1.00 1.00

ATOM 99 C THR A 20 -9.671 20.806 21.440 1.00 1.00

ATOM 100 O THR A 20 -10.119 20.538 22.558 1.00 1.00

ATOM 101 CB THR A 20 -7.155 20.868 21.552 1.00 1.00

ATOM 102 OG1 THR A 20 -6.028 21.747 21.657 1.00 1.00

ATOM 103 CG2 THR A 20 -6.900 19.888 20.421 1.00 1.00

ATOM 104 N VAL A 21 -10.220 20.377 20.307 1.00 1.00

ATOM 105 CA VAL A 21 -11.368 19.470 20.281 1.00 1.00

ATOM 106 C VAL A 21 -11.039 18.205 19.490 1.00 1.00

ATOM 107 O VAL A 21 -10.125 18.207 18.662 1.00 1.00

ATOM 108 CB VAL A 21 -12.647 20.149 19.688 1.00 1.00

ATOM 109 CG1 VAL A 21 -13.181 21.228 20.625 1.00 1.00

ATOM 110 CG2 VAL A 21 -12.377 20.723 18.297 1.00 1.00

ATOM 111 N GLU A 22 -11.775 17.129 19.764 1.00 1.00

ATOM 112 CA GLU A 22 -11.669 15.892 18.997 1.00 1.00

ATOM 113 C GLU A 22 -12.958 15.610 18.233 1.00 1.00

ATOM 114 O GLU A 22 -14.026 15.480 18.834 1.00 1.00

ATOM 115 CB GLU A 22 -11.328 14.697 19.895 1.00 1.00

ATOM 116 CG GLU A 22 -9.859 14.566 20.219 1.00 1.00

ATOM 117 CD GLU A 22 -9.403 13.118 20.381 1.00 1.00

ATOM 118 OE1 GLU A 22 -10.206 12.263 20.816 1.00 1.00

ATOM 119 OE2 GLU A 22 -8.224 12.842 20.075 1.00 1.00

ATOM 120 N ILE A 23 -12.842 15.518 16.912 1.00 1.00

ATOM 121 CA ILE A 23 -13.987 15.227 16.051 1.00 1.00

ATOM 122 C ILE A 23 -13.899 13.777 15.570 1.00 1.00

ATOM 123 O ILE A 23 -13.076 13.457 14.712 1.00 1.00

ATOM 124 CB ILE A 23 -14.085 16.186 14.828 1.00 1.00

ATOM 125 CG1 ILE A 23 -13.954 17.658 15.247 1.00 1.00

ATOM 126 CG2 ILE A 23 -15.390 15.958 14.068 1.00 1.00

ATOM 127 CD1 ILE A 23 -12.546 18.217 15.119 1.00 1.00

ATOM 128 N PRO A 24 -14.742 12.894 16.134 1.00 1.00

ATOM 129 CA PRO A 24 -14.700 11.486 15.737 1.00 1.00

ATOM 130 C PRO A 24 -15.362 11.231 14.386 1.00 1.00

ATOM 131 O PRO A 24 -16.301 11.941 14.003 1.00 1.00

ATOM 132 CB PRO A 24 -15.472 10.777 16.853 1.00 1.00

ATOM 133 CG PRO A 24 -16.382 11.800 17.393 1.00 1.00

ATOM 134 CD PRO A 24 -15.767 13.150 17.163 1.00 1.00

ATOM 135 N VAL A 25 -14.857 10.231 13.671 1.00 1.00

ATOM 136 CA VAL A 25 -15.447 9.799 12.413 1.00 1.00

ATOM 137 C VAL A 25 -15.990 8.392 12.635 1.00 1.00

ATOM 138 O VAL A 25 -15.219 7.452 12.855 1.00 1.00

ATOM 139 CB VAL A 25 -14.418 9.826 11.249 1.00 1.00

ATOM 140 CG1 VAL A 25 -15.070 9.399 9.942 1.00 1.00

ATOM 141 CG2 VAL A 25 -13.794 11.217 11.101 1.00 1.00

ATOM 142 N TYR A 26 -17.319 8.265 12.603 1.00 1.00

ATOM 143 CA TYR A 26 -17.997 7.005 12.904 1.00 1.00

ATOM 144 C TYR A 26 -18.279 6.204 11.649 1.00 1.00

ATOM 145 O TYR A 26 -18.656 6.763 10.618 1.00 1.00

ATOM 146 CB TYR A 26 -19.331 7.247 13.623 1.00 1.00

ATOM 147 CG TYR A 26 -19.241 7.980 14.942 1.00 1.00

ATOM 148 CD1 TYR A 26 -19.472 9.352 15.007 1.00 1.00

ATOM 149 CD2 TYR A 26 -18.947 7.303 16.123 1.00 1.00

ATOM 150 CE1 TYR A 26 -19.403 10.037 16.214 1.00 1.00

ATOM 151 CE2 TYR A 26 -18.874 7.977 17.338 1.00 1.00

ATOM 152 CZ TYR A 26 -19.105 9.345 17.374 1.00 1.00

ATOM 153 OH TYR A 26 -19.042 10.027 18.566 1.00 1.00

ATOM 154 N PHE A 27 -18.104 4.891 11.753 1.00 1.00

ATOM 155 CA PHE A 27 -18.497 3.962 10.701 1.00 1.00

ATOM 156 C PHE A 27 -19.699 3.165 11.187 1.00 1.00

ATOM 157 O PHE A 27 -19.677 2.616 12.289 1.00 1.00

ATOM 158 CB PHE A 27 -17.349 2.992 10.385 1.00 1.00

ATOM 159 CG PHE A 27 -16.347 3.512 9.381 1.00 1.00

ATOM 160 CD1 PHE A 27 -16.293 4.860 9.033 1.00 1.00

ATOM 161 CD2 PHE A 27 -15.426 2.639 8.810 1.00 1.00

ATOM 162 CE1 PHE A 27 -15.352 5.324 8.114 1.00 1.00

ATOM 163 CE2 PHE A 27 -14.481 3.093 7.892 1.00 1.00

ATOM 164 CZ PHE A 27 -14.445 4.440 7.545 1.00 1.00

ATOM 165 N ARG A 28 -20.745 3.117 10.368 1.00 1.00

ATOM 166 CA ARG A 28 -21.869 2.205 10.597 1.00 1.00

ATOM 167 C ARG A 28 -22.107 1.369 9.345 1.00 1.00

ATOM 168 O ARG A 28 -21.578 1.679 8.277 1.00 1.00

ATOM 169 CB ARG A 28 -23.141 2.965 10.995 1.00 1.00

ATOM 170 CG ARG A 28 -23.101 3.584 12.395 1.00 1.00

ATOM 171 CD ARG A 28 -24.470 4.115 12.829 1.00 1.00

ATOM 172 NE ARG A 28 -24.997 5.157 11.939 1.00 1.00

ATOM 173 CZ ARG A 28 -24.716 6.456 12.029 1.00 1.00

ATOM 174 NH1 ARG A 28 -23.892 6.911 12.963 1.00 1.00

ATOM 175 NH2 ARG A 28 -25.259 7.309 11.170 1.00 1.00

ATOM 176 N GLY A 29 -22.896 0.306 9.493 1.00 1.00

ATOM 177 CA GLY A 29 -23.212 -0.596 8.397 1.00 1.00

ATOM 178 C GLY A 29 -22.004 -1.327 7.850 1.00 1.00

ATOM 179 O GLY A 29 -21.932 -1.610 6.656 1.00 1.00

ATOM 180 N VAL A 30 -21.049 -1.627 8.729 1.00 1.00

ATOM 181 CA VAL A 30 -19.859 -2.385 8.352 1.00 1.00

ATOM 182 C VAL A 30 -20.292 -3.712 7.698 1.00 1.00

ATOM 183 O VAL A 30 -21.150 -4.414 8.233 1.00 1.00

ATOM 184 CB VAL A 30 -18.918 -2.609 9.572 1.00 1.00

ATOM 185 CG1 VAL A 30 -17.822 -3.621 9.257 1.00 1.00

ATOM 186 CG2 VAL A 30 -18.297 -1.281 10.012 1.00 1.00

ATOM 187 N PRO A 31 -19.716 -4.033 6.522 1.00 1.00

ATOM 188 CA PRO A 31 -20.104 -5.205 5.737 1.00 1.00

ATOM 189 C PRO A 31 -19.880 -6.525 6.473 1.00 1.00

ATOM 190 O PRO A 31 -19.088 -6.581 7.421 1.00 1.00

ATOM 191 CB PRO A 31 -19.178 -5.124 4.517 1.00 1.00

ATOM 192 CG PRO A 31 -18.779 -3.700 4.438 1.00 1.00

ATOM 193 CD PRO A 31 -18.650 -3.271 5.853 1.00 1.00

ATOM 194 N SER A 32 -20.568 -7.574 6.023 1.00 1.00

ATOM 195 CA SER A 32 -20.443 -8.905 6.620 1.00 1.00

ATOM 196 C SER A 32 -19.067 -9.522 6.367 1.00 1.00

ATOM 197 O SER A 32 -18.652 -10.449 7.074 1.00 1.00

ATOM 198 CB SER A 32 -21.545 -9.832 6.103 1.00 1.00

ATOM 199 OG SER A 32 -21.382 -10.078 4.719 1.00 1.00

ATOM 200 N LYS A 33 -18.364 -9.010 5.358 1.00 1.00

ATOM 201 CA LYS A 33 -17.000 -9.453 5.064 1.00 1.00

ATOM 202 C LYS A 33 -15.987 -8.342 5.355 1.00 1.00

ATOM 203 O LYS A 33 -14.885 -8.330 4.813 1.00 1.00

ATOM 204 CB LYS A 33 -16.886 -9.975 3.626 1.00 1.00

ATOM 205 CG LYS A 33 -17.821 -11.145 3.326 1.00 1.00

ATOM 206 CD LYS A 33 -17.475 -11.812 2.008 1.00 1.00

ATOM 207 CE LYS A 33 -18.575 -12.753 1.551 1.00 1.00

ATOM 208 NZ LYS A 33 -19.801 -11.998 1.151 1.00 1.00

ATOM 209 N GLY A 34 -16.385 -7.417 6.224 1.00 1.00

ATOM 210 CA GLY A 34 -15.506 -6.371 6.734 1.00 1.00

ATOM 211 C GLY A 34 -15.196 -5.240 5.776 1.00 1.00

ATOM 212 O GLY A 34 -15.730 -5.177 4.667 1.00 1.00

ATOM 213 N ILE A 35 -14.341 -4.329 6.225 1.00 1.00

ATOM 214 CA ILE A 35 -13.742 -3.330 5.350 1.00 1.00

ATOM 215 C ILE A 35 -12.246 -3.610 5.254 1.00 1.00

ATOM 216 O ILE A 35 -11.521 -3.474 6.237 1.00 1.00

ATOM 217 CB ILE A 35 -13.993 -1.883 5.849 1.00 1.00

ATOM 218 CG1 ILE A 35 -15.501 -1.597 5.906 1.00 1.00

ATOM 219 CG2 ILE A 35 -13.276 -0.869 4.947 1.00 1.00

ATOM 220 CD1 ILE A 35 -15.879 -0.342 6.677 1.00 1.00

ATOM 221 N ALA A 36 -11.807 -4.020 4.065 1.00 1.00

ATOM 222 CA ALA A 36 -10.390 -4.254 3.782 1.00 1.00

ATOM 223 C ALA A 36 -9.657 -2.957 3.457 1.00 1.00

ATOM 224 O ALA A 36 -8.468 -2.813 3.754 1.00 1.00

ATOM 225 CB ALA A 36 -10.231 -5.250 2.641 1.00 1.00

ATOM 226 N ASN A 37 -10.370 -2.017 2.837 1.00 1.00

ATOM 227 CA ASN A 37 -9.788 -0.731 2.462 1.00 1.00

ATOM 228 C ASN A 37 -10.820 0.366 2.296 1.00 1.00

ATOM 229 O ASN A 37 -11.979 0.099 1.959 1.00 1.00

ATOM 230 CB ASN A 37 -8.953 -0.854 1.175 1.00 1.00

ATOM 231 CG ASN A 37 -9.789 -1.253 -0.026 1.00 1.00

ATOM 232 OD1 ASN A 37 -10.403 -0.405 -0.681 1.00 1.00

ATOM 233 ND2 ASN A 37 -9.812 -2.546 -0.328 1.00 1.00

ATOM 234 N CYS A 38 -10.378 1.598 2.533 1.00 1.00

ATOM 235 CA CYS A 38 -11.169 2.783 2.239 1.00 1.00

ATOM 236 C CYS A 38 -10.275 4.002 2.065 1.00 1.00

ATOM 237 O CYS A 38 -9.263 4.153 2.753 1.00 1.00

ATOM 238 CB CYS A 38 -12.245 3.027 3.314 1.00 1.00

ATOM 239 SG CYS A 38 -11.654 3.302 4.988 1.00 1.00

ATOM 240 N ASP A 39 -10.644 4.857 1.118 1.00 1.00

ATOM 241 CA ASP A 39 -9.971 6.134 0.929 1.00 1.00

ATOM 242 C ASP A 39 -11.003 7.217 0.637 1.00 1.00

ATOM 243 O ASP A 39 -11.888 7.043 -0.203 1.00 1.00

ATOM 244 CB ASP A 39 -8.889 6.059 -0.166 1.00 1.00

ATOM 245 CG ASP A 39 -9.457 5.767 -1.554 1.00 1.00

ATOM 246 OD1 ASP A 39 -10.029 4.674 -1.771 1.00 1.00

ATOM 247 OD2 ASP A 39 -9.321 6.633 -2.438 1.00 1.00

ATOM 248 N PHE A 40 -10.907 8.319 1.373 1.00 1.00

ATOM 249 CA PHE A 40 -11.807 9.452 1.177 1.00 1.00

ATOM 250 C PHE A 40 -11.163 10.764 1.616 1.00 1.00

ATOM 251 O PHE A 40 -10.140 10.768 2.307 1.00 1.00

ATOM 252 CB PHE A 40 -13.160 9.227 1.878 1.00 1.00

ATOM 253 CG PHE A 40 -13.050 8.792 3.314 1.00 1.00

ATOM 254 CD1 PHE A 40 -13.014 9.731 4.340 1.00 1.00

ATOM 255 CD2 PHE A 40 -13.008 7.438 3.644 1.00 1.00

ATOM 256 CE1 PHE A 40 -12.924 9.327 5.669 1.00 1.00

ATOM 257 CE2 PHE A 40 -12.915 7.028 4.975 1.00 1.00

ATOM 258 CZ PHE A 40 -12.875 7.973 5.985 1.00 1.00

ATOM 259 N VAL A 41 -11.769 11.869 1.190 1.00 1.00

ATOM 260 CA VAL A 41 -11.240 13.206 1.440 1.00 1.00

ATOM 261 C VAL A 41 -12.330 14.062 2.092 1.00 1.00

ATOM 262 O VAL A 41 -13.472 14.076 1.624 1.00 1.00

ATOM 263 CB VAL A 41 -10.760 13.872 0.108 1.00 1.00

ATOM 264 CG1 VAL A 41 -10.305 15.307 0.344 1.00 1.00

ATOM 265 CG2 VAL A 41 -9.640 13.055 -0.540 1.00 1.00

ATOM 266 N PHE A 42 -11.982 14.737 3.189 1.00 1.00

ATOM 267 CA PHE A 42 -12.872 15.723 3.816 1.00 1.00

ATOM 268 C PHE A 42 -12.344 17.138 3.588 1.00 1.00

ATOM 269 O PHE A 42 -11.141 17.342 3.448 1.00 1.00

ATOM 270 CB PHE A 42 -13.017 15.507 5.331 1.00 1.00

ATOM 271 CG PHE A 42 -13.733 14.233 5.725 1.00 1.00

ATOM 272 CD1 PHE A 42 -14.516 13.519 4.820 1.00 1.00

ATOM 273 CD2 PHE A 42 -13.641 13.774 7.038 1.00 1.00

ATOM 274 CE1 PHE A 42 -15.170 12.352 5.205 1.00 1.00

ATOM 275 CE2 PHE A 42 -14.292 12.612 7.442 1.00 1.00

ATOM 276 CZ PHE A 42 -15.065 11.897 6.522 1.00 1.00

ATOM 277 N ARG A 43 -13.255 18.107 3.571 1.00 1.00

ATOM 278 CA ARG A 43 -12.890 19.516 3.487 1.00 1.00

ATOM 279 C ARG A 43 -13.072 20.202 4.829 1.00 1.00

ATOM 280 O ARG A 43 -13.918 19.803 5.631 1.00 1.00

ATOM 281 CB ARG A 43 -13.723 20.239 2.431 1.00 1.00

ATOM 282 CG ARG A 43 -13.233 20.015 1.025 1.00 1.00

ATOM 283 CD ARG A 43 -13.784 21.051 0.068 1.00 1.00

ATOM 284 NE ARG A 43 -13.323 20.777 -1.287 1.00 1.00

ATOM 285 CZ ARG A 43 -12.280 21.361 -1.863 1.00 1.00

ATOM 286 NH1 ARG A 43 -11.585 22.293 -1.219 1.00 1.00

ATOM 287 NH2 ARG A 43 -11.941 21.023 -3.100 1.00 1.00

ATOM 288 N TYR A 44 -12.269 21.237 5.057 1.00 1.00

ATOM 289 CA TYR A 44 -12.376 22.054 6.260 1.00 1.00

ATOM 290 C TYR A 44 -11.860 23.462 5.993 1.00 1.00

ATOM 291 O TYR A 44 -11.144 23.696 5.014 1.00 1.00

ATOM 292 CB TYR A 44 -11.609 21.413 7.423 1.00 1.00

ATOM 293 CG TYR A 44 -10.106 21.598 7.378 1.00 1.00

ATOM 294 CD1 TYR A 44 -9.475 22.518 8.217 1.00 1.00

ATOM 295 CD2 TYR A 44 -9.310 20.844 6.507 1.00 1.00

ATOM 296 CE1 TYR A 44 -8.101 22.688 8.192 1.00 1.00

ATOM 297 CE2 TYR A 44 -7.928 21.005 6.475 1.00 1.00

ATOM 298 CZ TYR A 44 -7.330 21.930 7.320 1.00 1.00

ATOM 299 OH TYR A 44 -5.964 22.097 7.297 1.00 1.00

ATOM 300 N ASP A 45 -12.224 24.395 6.864 1.00 1.00

ATOM 301 CA ASP A 45 -11.783 25.772 6.723 1.00 1.00

ATOM 302 C ASP A 45 -10.691 26.064 7.751 1.00 1.00

ATOM 303 O ASP A 45 -10.970 26.126 8.952 1.00 1.00

ATOM 304 CB ASP A 45 -12.974 26.732 6.868 1.00 1.00

ATOM 305 CG ASP A 45 -12.636 28.156 6.475 1.00 1.00

ATOM 306 OD1 ASP A 45 -11.469 28.571 6.640 1.00 1.00

ATOM 307 OD2 ASP A 45 -13.547 28.872 5.999 1.00 1.00

ATOM 308 N PRO A 46 -9.440 26.240 7.277 1.00 1.00

ATOM 309 CA PRO A 46 -8.282 26.456 8.148 1.00 1.00

ATOM 310 C PRO A 46 -8.304 27.793 8.901 1.00 1.00

ATOM 311 O PRO A 46 -7.526 27.975 9.834 1.00 1.00

ATOM 312 CB PRO A 46 -7.093 26.390 7.182 1.00 1.00

ATOM 313 CG PRO A 46 -7.654 26.761 5.861 1.00 1.00

ATOM 314 CD PRO A 46 -9.060 26.238 5.850 1.00 1.00

ATOM 315 N ASN A 47 -9.184 28.710 8.494 1.00 1.00

ATOM 316 CA ASN A 47 -9.424 29.949 9.236 1.00 1.00

ATOM 317 C ASN A 47 -10.141 29.685 10.554 1.00 1.00

ATOM 318 O ASN A 47 -9.995 30.444 11.515 1.00 1.00

ATOM 319 CB ASN A 47 -10.263 30.932 8.412 1.00 1.00

ATOM 320 CG ASN A 47 -9.490 31.561 7.274 1.00 1.00

ATOM 321 OD1 ASN A 47 -8.267 31.675 7.324 1.00 1.00

ATOM 322 ND2 ASN A 47 -10.209 31.989 6.239 1.00 1.00

ATOM 323 N VAL A 48 -10.923 28.608 10.578 1.00 1.00

ATOM 324 CA VAL A 48 -11.731 28.253 11.740 1.00 1.00

ATOM 325 C VAL A 48 -11.065 27.147 12.558 1.00 1.00

ATOM 326 O VAL A 48 -11.102 27.169 13.794 1.00 1.00

ATOM 327 CB VAL A 48 -13.158 27.804 11.315 1.00 1.00

ATOM 328 CG1 VAL A 48 -14.020 27.501 12.530 1.00 1.00

ATOM 329 CG2 VAL A 48 -13.823 28.868 10.439 1.00 1.00

ATOM 330 N LEU A 49 -10.459 26.185 11.865 1.00 1.00

ATOM 331 CA LEU A 49 -9.901 25.000 12.509 1.00 1.00

ATOM 332 C LEU A 49 -8.456 24.760 12.092 1.00 1.00

ATOM 333 O LEU A 49 -8.156 24.657 10.903 1.00 1.00

ATOM 334 CB LEU A 49 -10.750 23.755 12.191 1.00 1.00

ATOM 335 CG LEU A 49 -12.220 23.687 12.636 1.00 1.00

ATOM 336 CD1 LEU A 49 -12.843 22.346 12.251 1.00 1.00

ATOM 337 CD2 LEU A 49 -12.380 23.937 14.133 1.00 1.00

ATOM 338 N GLU A 50 -7.566 24.689 13.079 1.00 1.00

ATOM 339 CA GLU A 50 -6.204 24.235 12.842 1.00 1.00

ATOM 340 C GLU A 50 -6.115 22.755 13.195 1.00 1.00

ATOM 341 O GLU A 50 -6.076 22.386 14.372 1.00 1.00

ATOM 342 CB GLU A 50 -5.192 25.036 13.664 1.00 1.00

ATOM 343 CG GLU A 50 -3.741 24.630 13.405 1.00 1.00

ATOM 344 CD GLU A 50 -2.751 25.280 14.353 1.00 1.00

ATOM 345 OE1 GLU A 50 -3.175 25.852 15.384 1.00 1.00

ATOM 346 OE2 GLU A 50 -1.538 25.207 14.068 1.00 1.00

ATOM 347 N ILE A 51 -6.087 21.912 12.169 1.00 1.00

ATOM 348 CA ILE A 51 -6.007 20.471 12.371 1.00 1.00

ATOM 349 C ILE A 51 -4.571 20.076 12.711 1.00 1.00

ATOM 350 O ILE A 51 -3.655 20.237 11.896 1.00 1.00

ATOM 351 CB ILE A 51 -6.561 19.686 11.158 1.00 1.00

ATOM 352 CG1 ILE A 51 -8.013 20.111 10.893 1.00 1.00

ATOM 353 CG2 ILE A 51 -6.438 18.178 11.402 1.00 1.00

ATOM 354 CD1 ILE A 51 -8.740 19.316 9.832 1.00 1.00

ATOM 355 N ILE A 52 -4.388 19.572 13.926 1.00 1.00

ATOM 356 CA ILE A 52 -3.055 19.254 14.438 1.00 1.00

ATOM 357 C ILE A 52 -2.692 17.773 14.299 1.00 1.00

ATOM 358 O ILE A 52 -1.512 17.415 14.308 1.00 1.00

ATOM 359 CB ILE A 52 -2.847 19.768 15.891 1.00 1.00

ATOM 360 CG1 ILE A 52 -3.823 19.103 16.865 1.00 1.00

ATOM 361 CG2 ILE A 52 -2.968 21.304 15.936 1.00 1.00

ATOM 362 CD1 ILE A 52 -3.498 19.357 18.333 1.00 1.00

ATOM 363 N GLY A 53 -3.699 16.922 14.145 1.00 1.00

ATOM 364 CA GLY A 53 -3.460 15.498 13.937 1.00 1.00

ATOM 365 C GLY A 53 -4.724 14.681 13.776 1.00 1.00

ATOM 366 O GLY A 53 -5.817 15.135 14.119 1.00 1.00

ATOM 367 N ILE A 54 -4.563 13.477 13.233 1.00 1.00

ATOM 368 CA ILE A 54 -5.647 12.511 13.108 1.00 1.00

ATOM 369 C ILE A 54 -5.165 11.162 13.652 1.00 1.00

ATOM 370 O ILE A 54 -4.171 10.608 13.176 1.00 1.00

ATOM 371 CB ILE A 54 -6.137 12.372 11.641 1.00 1.00

ATOM 372 CG1 ILE A 54 -6.542 13.742 11.071 1.00 1.00

ATOM 373 CG2 ILE A 54 -7.305 11.394 11.553 1.00 1.00

ATOM 374 CD1 ILE A 54 -6.518 13.825 9.547 1.00 1.00

ATOM 375 N ASP A 55 -5.863 10.651 14.662 1.00 1.00

ATOM 376 CA ASP A 55 -5.512 9.369 15.265 1.00 1.00

ATOM 377 C ASP A 55 -6.453 8.279 14.775 1.00 1.00

ATOM 378 O ASP A 55 -7.631 8.551 14.535 1.00 1.00

ATOM 379 CB ASP A 55 -5.585 9.455 16.791 1.00 1.00

ATOM 380 CG ASP A 55 -4.680 10.532 17.358 1.00 1.00

ATOM 381 OD1 ASP A 55 -3.547 10.696 16.860 1.00 1.00

ATOM 382 OD2 ASP A 55 -5.108 11.217 18.309 1.00 1.00

ATOM 383 N PRO A 56 -5.939 7.042 14.622 1.00 1.00

ATOM 384 CA PRO A 56 -6.809 5.920 14.262 1.00 1.00

ATOM 385 C PRO A 56 -7.776 5.572 15.396 1.00 1.00

ATOM 386 O PRO A 56 -7.406 5.646 16.571 1.00 1.00

ATOM 387 CB PRO A 56 -5.824 4.769 14.015 1.00 1.00

ATOM 388 CG PRO A 56 -4.608 5.127 14.805 1.00 1.00

ATOM 389 CD PRO A 56 -4.528 6.627 14.764 1.00 1.00

ATOM 390 N GLY A 57 -9.007 5.220 15.041 1.00 1.00

ATOM 391 CA GLY A 57 -10.006 4.802 16.020 1.00 1.00

ATOM 392 C GLY A 57 -9.790 3.362 16.454 1.00 1.00

ATOM 393 O GLY A 57 -8.947 2.655 15.893 1.00 1.00

ATOM 394 N ASP A 58 -10.563 2.917 17.440 1.00 1.00

ATOM 395 CA ASP A 58 -10.371 1.577 17.995 1.00 1.00

ATOM 396 C ASP A 58 -10.884 0.424 17.119 1.00 1.00

ATOM 397 O ASP A 58 -10.655 -0.744 17.442 1.00 1.00

ATOM 398 CB ASP A 58 -10.904 1.482 19.437 1.00 1.00

ATOM 399 CG ASP A 58 -12.396 1.749 19.546 1.00 1.00

ATOM 400 OD1 ASP A 58 -13.017 2.216 18.566 1.00 1.00

ATOM 401 OD2 ASP A 58 -12.950 1.500 20.637 1.00 1.00

ATOM 402 N ILE A 59 -11.555 0.743 16.010 1.00 1.00

ATOM 403 CA ILE A 59 -11.946 -0.287 15.031 1.00 1.00

ATOM 404 C ILE A 59 -10.793 -0.644 14.082 1.00 1.00

ATOM 405 O ILE A 59 -10.927 -1.528 13.230 1.00 1.00

ATOM 406 CB ILE A 59 -13.239 0.077 14.230 1.00 1.00

ATOM 407 CG1 ILE A 59 -12.989 1.236 13.248 1.00 1.00

ATOM 408 CG2 ILE A 59 -14.405 0.337 15.190 1.00 1.00

ATOM 409 CD1 ILE A 59 -14.217 1.656 12.437 1.00 1.00

ATOM 410 N ILE A 60 -9.671 0.060 14.238 1.00 1.00

ATOM 411 CA ILE A 60 -8.428 -0.266 13.551 1.00 1.00

ATOM 412 C ILE A 60 -7.673 -1.236 14.455 1.00 1.00

ATOM 413 O ILE A 60 -7.156 -0.840 15.504 1.00 1.00

ATOM 414 CB ILE A 60 -7.564 1.003 13.275 1.00 1.00

ATOM 415 CG1 ILE A 60 -8.377 2.102 12.561 1.00 1.00

ATOM 416 CG2 ILE A 60 -6.279 0.652 12.510 1.00 1.00

ATOM 417 CD1 ILE A 60 -9.014 1.695 11.227 1.00 1.00

ATOM 418 N VAL A 61 -7.622 -2.504 14.051 1.00 1.00

ATOM 419 CA VAL A 61 -7.140 -3.583 14.926 1.00 1.00

ATOM 420 C VAL A 61 -5.694 -4.023 14.689 1.00 1.00

ATOM 421 O VAL A 61 -5.146 -4.786 15.490 1.00 1.00

ATOM 422 CB VAL A 61 -8.084 -4.822 14.917 1.00 1.00

ATOM 423 CG1 VAL A 61 -9.456 -4.455 15.489 1.00 1.00

ATOM 424 CG2 VAL A 61 -8.207 -5.422 13.510 1.00 1.00

ATOM 425 N ASP A 62 -5.084 -3.553 13.601 1.00 1.00

ATOM 426 CA ASP A 62 -3.665 -3.810 13.324 1.00 1.00

ATOM 427 C ASP A 62 -2.839 -3.368 14.543 1.00 1.00

ATOM 428 O ASP A 62 -2.880 -2.195 14.913 1.00 1.00

ATOM 429 CB ASP A 62 -3.233 -3.058 12.054 1.00 1.00

ATOM 430 CG ASP A 62 -1.855 -3.485 11.531 1.00 1.00

ATOM 431 OD1 ASP A 62 -1.028 -4.009 12.309 1.00 1.00

ATOM 432 OD2 ASP A 62 -1.591 -3.279 10.325 1.00 1.00

ATOM 433 N PRO A 63 -2.114 -4.315 15.186 1.00 1.00

ATOM 434 CA PRO A 63 -1.308 -3.997 16.378 1.00 1.00

ATOM 435 C PRO A 63 -0.228 -2.938 16.130 1.00 1.00

ATOM 436 O PRO A 63 0.291 -2.346 17.085 1.00 1.00

ATOM 437 CB PRO A 63 -0.673 -5.344 16.750 1.00 1.00

ATOM 438 CG PRO A 63 -0.741 -6.162 15.511 1.00 1.00

ATOM 439 CD PRO A 63 -2.016 -5.742 14.839 1.00 1.00

ATOM 440 N ASN A 64 0.112 -2.730 14.858 1.00 1.00

ATOM 441 CA ASN A 64 0.863 -1.556 14.421 1.00 1.00

ATOM 442 C ASN A 64 -0.026 -0.763 13.466 1.00 1.00

ATOM 443 O ASN A 64 0.013 -0.989 12.255 1.00 1.00

ATOM 444 CB ASN A 64 2.166 -1.958 13.728 1.00 1.00

ATOM 445 CG ASN A 64 3.038 -2.853 14.587 1.00 1.00

ATOM 446 OD1 ASN A 64 3.329 -3.986 14.214 1.00 1.00

ATOM 447 ND2 ASN A 64 3.450 -2.349 15.746 1.00 1.00

ATOM 448 N PRO A 65 -0.847 0.154 14.010 1.00 1.00

ATOM 449 CA PRO A 65 -1.883 0.851 13.235 1.00 1.00

ATOM 450 C PRO A 65 -1.380 1.569 11.979 1.00 1.00

ATOM 451 O PRO A 65 -2.113 1.633 10.995 1.00 1.00

ATOM 452 CB PRO A 65 -2.454 1.855 14.239 1.00 1.00

ATOM 453 CG PRO A 65 -2.173 1.249 15.570 1.00 1.00

ATOM 454 CD PRO A 65 -0.846 0.583 15.421 1.00 1.00

ATOM 455 N THR A 66 -0.148 2.082 12.015 1.00 1.00

ATOM 456 CA THR A 66 0.414 2.869 10.904 1.00 1.00

ATOM 457 C THR A 66 0.711 2.039 9.652 1.00 1.00

ATOM 458 O THR A 66 0.895 2.591 8.564 1.00 1.00

ATOM 459 CB THR A 66 1.682 3.668 11.322 1.00 1.00

ATOM 460 OG1 THR A 66 2.739 2.766 11.675 1.00 1.00

ATOM 461 CG2 THR A 66 1.377 4.591 12.500 1.00 1.00

ATOM 462 N LYS A 67 0.757 0.717 9.816 1.00 1.00

ATOM 463 CA LYS A 67 0.872 -0.213 8.692 1.00 1.00

ATOM 464 C LYS A 67 -0.420 -0.272 7.870 1.00 1.00

ATOM 465 O LYS A 67 -0.380 -0.495 6.655 1.00 1.00

ATOM 466 CB LYS A 67 1.235 -1.616 9.189 1.00 1.00

ATOM 467 CG LYS A 67 2.642 -1.753 9.772 1.00 1.00

ATOM 468 CD LYS A 67 3.694 -1.939 8.689 1.00 1.00

ATOM 469 CE LYS A 67 5.101 -1.965 9.284 1.00 1.00

ATOM 470 NZ LYS A 67 6.143 -2.112 8.226 1.00 1.00

ATOM 471 N SER A 68 -1.558 -0.077 8.533 1.00 1.00

ATOM 472 CA SER A 68 -2.866 -0.144 7.874 1.00 1.00

ATOM 473 C SER A 68 -3.509 1.224 7.651 1.00 1.00

ATOM 474 O SER A 68 -4.315 1.389 6.740 1.00 1.00

ATOM 475 CB SER A 68 -3.831 -1.028 8.675 1.00 1.00

ATOM 476 OG SER A 68 -3.453 -2.391 8.626 1.00 1.00

ATOM 477 N PHE A 69 -3.144 2.195 8.483 1.00 1.00

ATOM 478 CA PHE A 69 -3.851 3.471 8.563 1.00 1.00

ATOM 479 C PHE A 69 -2.884 4.640 8.403 1.00 1.00

ATOM 480 O PHE A 69 -1.877 4.716 9.102 1.00 1.00

ATOM 481 CB PHE A 69 -4.571 3.549 9.917 1.00 1.00

ATOM 482 CG PHE A 69 -5.376 4.809 10.133 1.00 1.00

ATOM 483 CD1 PHE A 69 -6.734 4.836 9.848 1.00 1.00

ATOM 484 CD2 PHE A 69 -4.784 5.952 10.668 1.00 1.00

ATOM 485 CE1 PHE A 69 -7.490 5.990 10.065 1.00 1.00

ATOM 486 CE2 PHE A 69 -5.525 7.113 10.886 1.00 1.00

ATOM 487 CZ PHE A 69 -6.880 7.131 10.584 1.00 1.00

ATOM 488 N ASP A 70 -3.196 5.546 7.478 1.00 1.00

ATOM 489 CA ASP A 70 -2.423 6.777 7.305 1.00 1.00

ATOM 490 C ASP A 70 -3.351 7.918 6.903 1.00 1.00

ATOM 491 O ASP A 70 -4.422 7.690 6.324 1.00 1.00

ATOM 492 CB ASP A 70 -1.315 6.590 6.250 1.00 1.00

ATOM 493 CG ASP A 70 -0.162 7.592 6.408 1.00 1.00

ATOM 494 OD1 ASP A 70 -0.284 8.571 7.176 1.00 1.00

ATOM 495 OD2 ASP A 70 0.889 7.385 5.770 1.00 1.00

ATOM 496 N THR A 71 -2.943 9.140 7.229 1.00 1.00

ATOM 497 CA THR A 71 -3.674 10.340 6.833 1.00 1.00

ATOM 498 C THR A 71 -2.712 11.412 6.315 1.00 1.00

ATOM 499 O THR A 71 -1.494 11.303 6.491 1.00 1.00

ATOM 500 CB THR A 71 -4.499 10.931 8.007 1.00 1.00

ATOM 501 OG1 THR A 71 -3.630 11.235 9.100 1.00 1.00

ATOM 502 CG2 THR A 71 -5.590 9.966 8.472 1.00 1.00

ATOM 503 N ALA A 72 -3.270 12.444 5.684 1.00 1.00

ATOM 504 CA ALA A 72 -2.500 13.610 5.240 1.00 1.00

ATOM 505 C ALA A 72 -3.314 14.893 5.414 1.00 1.00

ATOM 506 O ALA A 72 -4.512 14.924 5.121 1.00 1.00

ATOM 507 CB ALA A 72 -2.051 13.441 3.790 1.00 1.00

ATOM 508 N ILE A 73 -2.658 15.943 5.906 1.00 1.00

ATOM 509 CA ILE A 73 -3.309 17.235 6.151 1.00 1.00

ATOM 510 C ILE A 73 -2.742 18.301 5.202 1.00 1.00

ATOM 511 O ILE A 73 -1.524 18.502 5.140 1.00 1.00

ATOM 512 CB ILE A 73 -3.158 17.674 7.644 1.00 1.00

ATOM 513 CG1 ILE A 73 -3.815 16.648 8.576 1.00 1.00

ATOM 514 CG2 ILE A 73 -3.743 19.074 7.874 1.00 1.00

ATOM 515 CD1 ILE A 73 -3.249 16.608 9.996 1.00 1.00

ATOM 516 N TYR A 74 -3.634 18.967 4.465 1.00 1.00

ATOM 517 CA TYR A 74 -3.256 20.028 3.519 1.00 1.00

ATOM 518 C TYR A 74 -3.990 21.344 3.795 1.00 1.00

ATOM 519 O TYR A 74 -5.051 21.594 3.220 1.00 1.00

ATOM 520 CB TYR A 74 -3.529 19.597 2.074 1.00 1.00

ATOM 521 CG TYR A 74 -2.895 18.294 1.665 1.00 1.00

ATOM 522 CD1 TYR A 74 -3.638 17.116 1.647 1.00 1.00

ATOM 523 CD2 TYR A 74 -1.553 18.236 1.291 1.00 1.00

ATOM 524 CE1 TYR A 74 -3.059 15.914 1.271 1.00 1.00

ATOM 525 CE2 TYR A 74 -0.967 17.033 0.912 1.00 1.00

ATOM 526 CZ TYR A 74 -1.728 15.882 0.903 1.00 1.00

ATOM 527 OH TYR A 74 -1.154 14.698 0.531 1.00 1.00

ATOM 528 N PRO A 75 -3.426 22.197 4.670 1.00 1.00

ATOM 529 CA PRO A 75 -4.018 23.507 4.973 1.00 1.00

ATOM 530 C PRO A 75 -4.176 24.403 3.737 1.00 1.00

ATOM 531 O PRO A 75 -5.226 25.029 3.573 1.00 1.00

ATOM 532 CB PRO A 75 -3.025 24.121 5.969 1.00 1.00

ATOM 533 CG PRO A 75 -2.319 22.958 6.562 1.00 1.00

ATOM 534 CD PRO A 75 -2.197 21.967 5.448 1.00 1.00

ATOM 535 N ASP A 76 -3.156 24.444 2.877 1.00 1.00

ATOM 536 CA ASP A 76 -3.205 25.231 1.635 1.00 1.00

ATOM 537 C ASP A 76 -4.327 24.754 0.705 1.00 1.00

ATOM 538 O ASP A 76 -4.958 25.558 0.018 1.00 1.00

ATOM 539 CB ASP A 76 -1.859 25.175 0.893 1.00 1.00

ATOM 540 CG ASP A 76 -0.707 25.793 1.686 1.00 1.00

ATOM 541 OD1 ASP A 76 -0.662 25.653 2.931 1.00 1.00

ATOM 542 OD2 ASP A 76 0.176 26.408 1.051 1.00 1.00

ATOM 543 N ARG A 77 -4.563 23.445 0.695 1.00 1.00

ATOM 544 CA ARG A 77 -5.591 22.833 -0.143 1.00 1.00

ATOM 545 C ARG A 77 -6.939 22.708 0.571 1.00 1.00

ATOM 546 O ARG A 77 -7.957 22.414 -0.063 1.00 1.00

ATOM 547 CB ARG A 77 -5.134 21.452 -0.613 1.00 1.00

ATOM 548 CG ARG A 77 -4.158 21.479 -1.768 1.00 1.00

ATOM 549 CD ARG A 77 -3.869 20.072 -2.252 1.00 1.00

ATOM 550 NE ARG A 77 -2.506 19.644 -1.946 1.00 1.00

ATOM 551 CZ ARG A 77 -1.988 18.476 -2.318 1.00 1.00

ATOM 552 NH1 ARG A 77 -2.724 17.607 -3.000 1.00 1.00

ATOM 553 NH2 ARG A 77 -0.733 18.177 -2.008 1.00 1.00

ATOM 554 N LYS A 78 -6.930 22.920 1.885 1.00 1.00

ATOM 555 CA LYS A 78 -8.135 22.888 2.726 1.00 1.00

ATOM 556 C LYS A 78 -8.792 21.505 2.781 1.00 1.00

ATOM 557 O LYS A 78 -10.015 21.390 2.880 1.00 1.00

ATOM 558 CB LYS A 78 -9.151 23.946 2.274 1.00 1.00

ATOM 559 CG LYS A 78 -8.625 25.360 2.285 1.00 1.00

ATOM 560 CD LYS A 78 -9.634 26.321 1.688 1.00 1.00

ATOM 561 CE LYS A 78 -9.181 27.763 1.868 1.00 1.00

ATOM 562 NZ LYS A 78 -7.787 27.984 1.368 1.00 1.00

ATOM 563 N ILE A 79 -7.970 20.461 2.720 1.00 1.00

ATOM 564 CA ILE A 79 -8.463 19.086 2.764 1.00 1.00

ATOM 565 C ILE A 79 -7.667 18.209 3.729 1.00 1.00

ATOM 566 O ILE A 79 -6.512 18.503 4.059 1.00 1.00

ATOM 567 CB ILE A 79 -8.482 18.409 1.351 1.00 1.00

ATOM 568 CG1 ILE A 79 -7.081 18.402 0.714 1.00 1.00

ATOM 569 CG2 ILE A 79 -9.541 19.057 0.447 1.00 1.00

ATOM 570 CD1 ILE A 79 -6.961 17.600 -0.579 1.00 1.00

ATOM 571 N ILE A 80 -8.315 17.138 4.178 1.00 1.00

ATOM 572 CA ILE A 80 -7.661 16.054 4.909 1.00 1.00

ATOM 573 C ILE A 80 -7.984 14.735 4.212 1.00 1.00

ATOM 574 O ILE A 80 -9.098 14.538 3.721 1.00 1.00

ATOM 575 CB ILE A 80 -8.052 16.015 6.417 1.00 1.00

ATOM 576 CG1 ILE A 80 -9.573 16.031 6.606 1.00 1.00

ATOM 577 CG2 ILE A 80 -7.394 17.178 7.165 1.00 1.00

ATOM 578 CD1 ILE A 80 -10.036 15.752 8.044 1.00 1.00

ATOM 579 N VAL A 81 -7.002 13.844 4.145 1.00 1.00

ATOM 580 CA VAL A 81 -7.142 12.609 3.375 1.00 1.00

ATOM 581 C VAL A 81 -6.954 11.393 4.284 1.00 1.00

ATOM 582 O VAL A 81 -6.085 11.394 5.155 1.00 1.00

ATOM 583 CB VAL A 81 -6.133 12.565 2.186 1.00 1.00

ATOM 584 CG1 VAL A 81 -6.481 11.432 1.205 1.00 1.00

ATOM 585 CG2 VAL A 81 -6.092 13.909 1.456 1.00 1.00

ATOM 586 N PHE A 82 -7.787 10.375 4.085 1.00 1.00

ATOM 587 CA PHE A 82 -7.749 9.151 4.880 1.00 1.00

ATOM 588 C PHE A 82 -7.448 7.960 3.982 1.00 1.00

ATOM 589 O PHE A 82 -8.071 7.800 2.927 1.00 1.00

ATOM 590 CB PHE A 82 -9.093 8.913 5.587 1.00 1.00

ATOM 591 CG PHE A 82 -9.513 10.022 6.525 1.00 1.00

ATOM 592 CD1 PHE A 82 -10.103 11.189 6.034 1.00 1.00

ATOM 593 CD2 PHE A 82 -9.348 9.885 7.903 1.00 1.00

ATOM 594 CE1 PHE A 82 -10.502 12.204 6.899 1.00 1.00

ATOM 595 CE2 PHE A 82 -9.750 10.897 8.776 1.00 1.00

ATOM 596 CZ PHE A 82 -10.326 12.058 8.276 1.00 1.00

ATOM 597 N LEU A 83 -6.500 7.127 4.400 1.00 1.00

ATOM 598 CA LEU A 83 -6.200 5.882 3.695 1.00 1.00

ATOM 599 C LEU A 83 -6.121 4.697 4.664 1.00 1.00

ATOM 600 O LEU A 83 -5.244 4.641 5.526 1.00 1.00

ATOM 601 CB LEU A 83 -4.905 6.001 2.875 1.00 1.00

ATOM 602 CG LEU A 83 -4.393 4.762 2.118 1.00 1.00

ATOM 603 CD1 LEU A 83 -5.351 4.314 0.996 1.00 1.00

ATOM 604 CD2 LEU A 83 -3.008 5.007 1.557 1.00 1.00

ATOM 605 N PHE A 84 -7.066 3.772 4.518 1.00 1.00

ATOM 606 CA PHE A 84 -7.031 2.506 5.235 1.00 1.00

ATOM 607 C PHE A 84 -6.895 1.361 4.237 1.00 1.00

ATOM 608 O PHE A 84 -7.620 1.299 3.248 1.00 1.00

ATOM 609 CB PHE A 84 -8.284 2.313 6.099 1.00 1.00

ATOM 610 CG PHE A 84 -8.361 0.959 6.762 1.00 1.00

ATOM 611 CD1 PHE A 84 -7.604 0.681 7.897 1.00 1.00

ATOM 612 CD2 PHE A 84 -9.182 -0.038 6.244 1.00 1.00

ATOM 613 CE1 PHE A 84 -7.666 -0.576 8.514 1.00 1.00

ATOM 614 CE2 PHE A 84 -9.253 -1.296 6.850 1.00 1.00

ATOM 615 CZ PHE A 84 -8.493 -1.564 7.988 1.00 1.00

ATOM 616 N ALA A 85 -5.942 0.471 4.500 1.00 1.00

ATOM 617 CA ALA A 85 -5.773 -0.748 3.723 1.00 1.00

ATOM 618 C ALA A 85 -5.183 -1.789 4.658 1.00 1.00

ATOM 619 O ALA A 85 -3.995 -1.720 5.003 1.00 1.00

ATOM 620 CB ALA A 85 -4.854 -0.516 2.518 1.00 1.00

ATOM 621 N GLU A 86 -6.024 -2.724 5.092 1.00 1.00

ATOM 622 CA GLU A 86 -5.623 -3.719 6.083 1.00 1.00

ATOM 623 C GLU A 86 -4.335 -4.419 5.631 1.00 1.00

ATOM 624 O GLU A 86 -4.236 -4.867 4.486 1.00 1.00

ATOM 625 CB GLU A 86 -6.780 -4.690 6.378 1.00 1.00

ATOM 626 CG GLU A 86 -7.037 -5.778 5.339 1.00 1.00

ATOM 627 CD GLU A 86 -6.175 -7.011 5.572 1.00 1.00

ATOM 628 OE1 GLU A 86 -5.709 -7.195 6.715 1.00 1.00

ATOM 629 OE2 GLU A 86 -5.959 -7.788 4.617 1.00 1.00

ATOM 630 N ASP A 87 -3.346 -4.474 6.522 1.00 1.00

ATOM 631 CA ASP A 87 -1.975 -4.830 6.125 1.00 1.00

ATOM 632 C ASP A 87 -1.632 -6.324 6.181 1.00 1.00

ATOM 633 O ASP A 87 -0.612 -6.738 5.624 1.00 1.00

ATOM 634 CB ASP A 87 -0.941 -4.011 6.925 1.00 1.00

ATOM 635 CG ASP A 87 0.443 -4.001 6.266 1.00 1.00

ATOM 636 OD1 ASP A 87 0.548 -3.685 5.066 1.00 1.00

ATOM 637 OD2 ASP A 87 1.436 -4.305 6.960 1.00 1.00

ATOM 638 N SER A 88 -2.478 -7.127 6.823 1.00 1.00

ATOM 639 CA SER A 88 -2.232 -8.574 6.936 1.00 1.00

ATOM 640 C SER A 88 -2.112 -9.263 5.571 1.00 1.00

ATOM 641 O SER A 88 -1.280 -10.152 5.387 1.00 1.00

ATOM 642 CB SER A 88 -3.323 -9.254 7.771 1.00 1.00

ATOM 643 OG SER A 88 -4.476 -9.523 6.991 1.00 1.00

ATOM 644 N GLY A 89 -2.946 -8.840 4.621 1.00 1.00

ATOM 645 CA GLY A 89 -3.019 -9.473 3.307 1.00 1.00

ATOM 646 C GLY A 89 -3.995 -10.636 3.261 1.00 1.00

ATOM 647 O GLY A 89 -4.243 -11.203 2.193 1.00 1.00

ATOM 648 N THR A 90 -4.548 -10.984 4.421 1.00 1.00

ATOM 649 CA THR A 90 -5.435 -12.142 4.559 1.00 1.00

ATOM 650 C THR A 90 -6.845 -11.740 4.997 1.00 1.00

ATOM 651 O THR A 90 -7.757 -12.572 5.021 1.00 1.00

ATOM 652 CB THR A 90 -4.871 -13.185 5.561 1.00 1.00

ATOM 653 OG1 THR A 90 -4.949 -12.668 6.896 1.00 1.00

ATOM 654 CG2 THR A 90 -3.424 -13.540 5.230 1.00 1.00

ATOM 655 N GLY A 91 -7.012 -10.465 5.331 1.00 1.00

ATOM 656 CA GLY A 91 -8.272 -9.948 5.855 1.00 1.00

ATOM 657 C GLY A 91 -8.318 -9.870 7.371 1.00 1.00

ATOM 658 O GLY A 91 -9.315 -9.429 7.941 1.00 1.00

ATOM 659 N ALA A 92 -7.234 -10.294 8.020 1.00 1.00

ATOM 660 CA ALA A 92 -7.168 -10.347 9.486 1.00 1.00

ATOM 661 C ALA A 92 -7.287 -8.978 10.163 1.00 1.00

ATOM 662 O ALA A 92 -7.823 -8.876 11.273 1.00 1.00

ATOM 663 CB ALA A 92 -5.898 -11.055 9.942 1.00 1.00

ATOM 664 N TYR A 93 -6.799 -7.933 9.496 1.00 1.00

ATOM 665 CA TYR A 93 -6.823 -6.585 10.071 1.00 1.00

ATOM 666 C TYR A 93 -7.925 -5.704 9.490 1.00 1.00

ATOM 667 O TYR A 93 -7.917 -4.483 9.677 1.00 1.00

ATOM 668 CB TYR A 93 -5.454 -5.899 9.943 1.00 1.00

ATOM 669 CG TYR A 93 -4.315 -6.594 10.667 1.00 1.00

ATOM 670 CD1 TYR A 93 -4.547 -7.401 11.782 1.00 1.00

ATOM 671 CD2 TYR A 93 -2.996 -6.419 10.248 1.00 1.00

ATOM 672 CE1 TYR A 93 -3.496 -8.034 12.442 1.00 1.00

ATOM 673 CE2 TYR A 93 -1.944 -7.038 10.906 1.00 1.00

ATOM 674 CZ TYR A 93 -2.198 -7.841 11.997 1.00 1.00

ATOM 675 OH TYR A 93 -1.149 -8.451 12.645 1.00 1.00

ATOM 676 N ALA A 94 -8.872 -6.326 8.789 1.00 1.00

ATOM 677 CA ALA A 94 -10.048 -5.623 8.281 1.00 1.00

ATOM 678 C ALA A 94 -10.872 -5.046 9.428 1.00 1.00

ATOM 679 O ALA A 94 -10.768 -5.506 10.569 1.00 1.00

ATOM 680 CB ALA A 94 -10.909 -6.555 7.428 1.00 1.00

ATOM 681 N ILE A 95 -11.675 -4.032 9.116 1.00 1.00

ATOM 682 CA ILE A 95 -12.630 -3.463 10.059 1.00 1.00

ATOM 683 C ILE A 95 -13.841 -4.394 10.109 1.00 1.00

ATOM 684 O ILE A 95 -14.432 -4.697 9.071 1.00 1.00

ATOM 685 CB ILE A 95 -13.034 -2.017 9.642 1.00 1.00

ATOM 686 CG1 ILE A 95 -11.823 -1.080 9.746 1.00 1.00

ATOM 687 CG2 ILE A 95 -14.201 -1.505 10.484 1.00 1.00

ATOM 688 CD1 ILE A 95 -11.958 0.230 8.982 1.00 1.00

ATOM 689 N THR A 96 -14.195 -4.857 11.306 1.00 1.00

ATOM 690 CA THR A 96 -15.245 -5.879 11.459 1.00 1.00

ATOM 691 C THR A 96 -16.461 -5.416 12.270 1.00 1.00

ATOM 692 O THR A 96 -17.512 -6.063 12.242 1.00 1.00

ATOM 693 CB THR A 96 -14.685 -7.189 12.084 1.00 1.00

ATOM 694 OG1 THR A 96 -13.990 -6.884 13.298 1.00 1.00

ATOM 695 CG2 THR A 96 -13.738 -7.884 11.127 1.00 1.00

ATOM 696 N LYS A 97 -16.320 -4.300 12.980 1.00 1.00

ATOM 697 CA LYS A 97 -17.417 -3.756 13.774 1.00 1.00

ATOM 698 C LYS A 97 -17.630 -2.267 13.506 1.00 1.00

ATOM 699 O LYS A 97 -16.707 -1.576 13.062 1.00 1.00

ATOM 700 CB LYS A 97 -17.171 -4.004 15.271 1.00 1.00

ATOM 701 CG LYS A 97 -15.984 -3.236 15.865 1.00 1.00

ATOM 702 CD LYS A 97 -15.708 -3.586 17.338 1.00 1.00

ATOM 703 CE LYS A 97 -16.844 -3.183 18.285 1.00 1.00

ATOM 704 NZ LYS A 97 -17.207 -1.741 18.195 1.00 1.00

ATOM 705 N ASP A 98 -18.845 -1.789 13.777 1.00 1.00

ATOM 706 CA ASP A 98 -19.152 -0.358 13.794 1.00 1.00

ATOM 707 C ASP A 98 -18.367 0.313 14.914 1.00 1.00

ATOM 708 O ASP A 98 -18.039 -0.326 15.914 1.00 1.00

ATOM 709 CB ASP A 98 -20.635 -0.118 14.081 1.00 1.00

ATOM 710 CG ASP A 98 -21.562 -0.618 12.982 1.00 1.00

ATOM 711 OD1 ASP A 98 -21.103 -1.090 11.916 1.00 1.00

ATOM 712 OD2 ASP A 98 -22.784 -0.518 13.203 1.00 1.00

ATOM 713 N GLY A 99 -18.092 1.606 14.761 1.00 1.00

ATOM 714 CA GLY A 99 -17.406 2.379 15.799 1.00 1.00

ATOM 715 C GLY A 99 -16.602 3.536 15.236 1.00 1.00

ATOM 716 O GLY A 99 -16.848 3.989 14.118 1.00 1.00

ATOM 717 N VAL A 100 -15.638 4.014 16.017 1.00 1.00

ATOM 718 CA VAL A 100 -14.830 5.166 15.621 1.00 1.00

ATOM 719 C VAL A 100 -13.690 4.717 14.712 1.00 1.00

ATOM 720 O VAL A 100 -12.858 3.891 15.101 1.00 1.00

ATOM 721 CB VAL A 100 -14.284 5.943 16.855 1.00 1.00

ATOM 722 CG1 VAL A 100 -13.367 7.079 16.423 1.00 1.00

ATOM 723 CG2 VAL A 100 -15.432 6.483 17.696 1.00 1.00

ATOM 724 N PHE A 101 -13.682 5.258 13.495 1.00 1.00

ATOM 725 CA PHE A 101 -12.631 5.001 12.510 1.00 1.00

ATOM 726 C PHE A 101 -11.405 5.892 12.738 1.00 1.00

ATOM 727 O PHE A 101 -10.267 5.439 12.611 1.00 1.00

ATOM 728 CB PHE A 101 -13.189 5.214 11.096 1.00 1.00

ATOM 729 CG PHE A 101 -12.143 5.214 10.003 1.00 1.00

ATOM 730 CD1 PHE A 101 -11.512 4.033 9.620 1.00 1.00

ATOM 731 CD2 PHE A 101 -11.826 6.390 9.330 1.00 1.00

ATOM 732 CE1 PHE A 101 -10.562 4.027 8.593 1.00 1.00

ATOM 733 CE2 PHE A 101 -10.876 6.395 8.304 1.00 1.00

ATOM 734 CZ PHE A 101 -10.243 5.211 7.937 1.00 1.00

ATOM 735 N ALA A 102 -11.655 7.158 13.057 1.00 1.00

ATOM 736 CA ALA A 102 -10.586 8.135 13.274 1.00 1.00

ATOM 737 C ALA A 102 -11.052 9.241 14.205 1.00 1.00

ATOM 738 O ALA A 102 -12.255 9.473 14.349 1.00 1.00

ATOM 739 CB ALA A 102 -10.110 8.725 11.940 1.00 1.00

ATOM 740 N LYS A 103 -10.091 9.907 14.841 1.00 1.00

ATOM 741 CA LYS A 103 -10.359 11.081 15.665 1.00 1.00

ATOM 742 C LYS A 103 -9.519 12.249 15.160 1.00 1.00

ATOM 743 O LYS A 103 -8.285 12.196 15.179 1.00 1.00

ATOM 744 CB LYS A 103 -10.069 10.795 17.141 1.00 1.00

ATOM 745 CG LYS A 103 -10.962 9.709 17.754 1.00 1.00

ATOM 746 CD LYS A 103 -10.514 9.288 19.162 1.00 1.00

ATOM 747 CE LYS A 103 -9.187 8.521 19.159 1.00 1.00

ATOM 748 NZ LYS A 103 -9.169 7.361 18.225 1.00 1.00

ATOM 749 N ILE A 104 -10.195 13.289 14.686 1.00 1.00

ATOM 750 CA ILE A 104 -9.529 14.478 14.160 1.00 1.00

ATOM 751 C ILE A 104 -9.290 15.454 15.307 1.00 1.00

ATOM 752 O ILE A 104 -10.241 15.879 15.970 1.00 1.00

ATOM 753 CB ILE A 104 -10.373 15.171 13.058 1.00 1.00

ATOM 754 CG1 ILE A 104 -10.782 14.169 11.970 1.00 1.00

ATOM 755 CG2 ILE A 104 -9.607 16.357 12.452 1.00 1.00

ATOM 756 CD1 ILE A 104 -12.005 14.589 11.162 1.00 1.00

ATOM 757 N ARG A 105 -8.023 15.786 15.548 1.00 1.00

ATOM 758 CA ARG A 105 -7.664 16.752 16.583 1.00 1.00

ATOM 759 C ARG A 105 -7.484 18.133 15.972 1.00 1.00

ATOM 760 O ARG A 105 -6.692 18.318 15.043 1.00 1.00

ATOM 761 CB ARG A 105 -6.400 16.325 17.334 1.00 1.00

ATOM 762 CG ARG A 105 -6.659 15.427 18.535 1.00 1.00

ATOM 763 CD ARG A 105 -5.368 14.962 19.208 1.00 1.00

ATOM 764 NE ARG A 105 -4.574 14.072 18.358 1.00 1.00

ATOM 765 CZ ARG A 105 -3.483 14.436 17.685 1.00 1.00

ATOM 766 NH1 ARG A 105 -3.028 15.682 17.753 1.00 1.00

ATOM 767 NH2 ARG A 105 -2.840 13.547 16.939 1.00 1.00

ATOM 768 N ALA A 106 -8.231 19.102 16.495 1.00 1.00

ATOM 769 CA ALA A 106 -8.243 20.448 15.935 1.00 1.00

ATOM 770 C ALA A 106 -8.298 21.529 17.010 1.00 1.00

ATOM 771 O ALA A 106 -9.077 21.433 17.964 1.00 1.00

ATOM 772 CB ALA A 106 -9.416 20.602 14.975 1.00 1.00

ATOM 773 N THR A 107 -7.471 22.556 16.839 1.00 1.00

ATOM 774 CA THR A 107 -7.539 23.757 17.667 1.00 1.00

ATOM 775 C THR A 107 -8.529 24.750 17.046 1.00 1.00

ATOM 776 O THR A 107 -8.474 25.031 15.846 1.00 1.00

ATOM 777 CB THR A 107 -6.148 24.403 17.835 1.00 1.00

ATOM 778 OG1 THR A 107 -5.219 23.420 18.299 1.00 1.00

ATOM 779 CG2 THR A 107 -6.194 25.560 18.834 1.00 1.00

ATOM 780 N VAL A 108 -9.436 25.259 17.874 1.00 1.00

ATOM 781 CA VAL A 108 -10.471 26.198 17.436 1.00 1.00

ATOM 782 C VAL A 108 -9.882 27.606 17.283 1.00 1.00

ATOM 783 O VAL A 108 -9.389 28.195 18.250 1.00 1.00

ATOM 784 CB VAL A 108 -11.667 26.209 18.424 1.00 1.00

ATOM 785 CG1 VAL A 108 -12.794 27.124 17.923 1.00 1.00

ATOM 786 CG2 VAL A 108 -12.188 24.793 18.650 1.00 1.00

ATOM 787 N LYS A 109 -9.926 28.131 16.062 1.00 1.00

ATOM 788 CA LYS A 109 -9.315 29.428 15.754 1.00 1.00

ATOM 789 C LYS A 109 -10.315 30.582 15.751 1.00 1.00

ATOM 790 O LYS A 109 -9.933 31.740 15.952 1.00 1.00

ATOM 791 CB LYS A 109 -8.566 29.372 14.420 1.00 1.00

ATOM 792 CG LYS A 109 -7.273 28.570 14.462 1.00 1.00

ATOM 793 CD LYS A 109 -6.663 28.407 13.065 1.00 1.00

ATOM 794 CE LYS A 109 -6.249 29.739 12.459 1.00 1.00

ATOM 795 NZ LYS A 109 -5.496 29.572 11.190 1.00 1.00

ATOM 796 N SER A 110 -11.584 30.272 15.504 1.00 1.00

ATOM 797 CA SER A 110 -12.647 31.273 15.587 1.00 1.00

ATOM 798 C SER A 110 -13.962 30.637 16.032 1.00 1.00

ATOM 799 O SER A 110 -14.119 29.416 15.972 1.00 1.00

ATOM 800 CB SER A 110 -12.817 32.014 14.249 1.00 1.00

ATOM 801 OG SER A 110 -13.853 31.460 13.462 1.00 1.00

ATOM 802 N SER A 111 -14.893 31.477 16.475 1.00 1.00

ATOM 803 CA SER A 111 -16.203 31.031 16.957 1.00 1.00

ATOM 804 C SER A 111 -17.192 30.711 15.832 1.00 1.00

ATOM 805 O SER A 111 -18.275 30.181 16.095 1.00 1.00

ATOM 806 CB SER A 111 -16.810 32.074 17.909 1.00 1.00

ATOM 807 OG SER A 111 -17.113 33.281 17.223 1.00 1.00

ATOM 808 N ALA A 112 -16.825 31.042 14.593 1.00 1.00

ATOM 809 CA ALA A 112 -17.637 30.685 13.425 1.00 1.00

ATOM 810 C ALA A 112 -17.697 29.159 13.297 1.00 1.00

ATOM 811 O ALA A 112 -16.781 28.471 13.748 1.00 1.00

ATOM 812 CB ALA A 112 -17.058 31.313 12.165 1.00 1.00

ATOM 813 N PRO A 113 -18.788 28.619 12.717 1.00 1.00

ATOM 814 CA PRO A 113 -18.859 27.163 12.563 1.00 1.00

ATOM 815 C PRO A 113 -17.661 26.597 11.804 1.00 1.00

ATOM 816 O PRO A 113 -17.212 27.187 10.814 1.00 1.00

ATOM 817 CB PRO A 113 -20.142 26.948 11.756 1.00 1.00

ATOM 818 CG PRO A 113 -20.961 28.146 12.006 1.00 1.00

ATOM 819 CD PRO A 113 -19.999 29.284 12.200 1.00 1.00

ATOM 820 N GLY A 114 -17.149 25.472 12.289 1.00 1.00

ATOM 821 CA GLY A 114 -16.038 24.778 11.659 1.00 1.00

ATOM 822 C GLY A 114 -16.499 23.461 11.076 1.00 1.00

ATOM 823 O GLY A 114 -16.257 22.401 11.650 1.00 1.00

ATOM 824 N TYR A 115 -17.175 23.535 9.934 1.00 1.00

ATOM 825 CA TYR A 115 -17.709 22.338 9.291 1.00 1.00

ATOM 826 C TYR A 115 -16.616 21.480 8.665 1.00 1.00

ATOM 827 O TYR A 115 -15.709 21.988 7.998 1.00 1.00

ATOM 828 CB TYR A 115 -18.755 22.707 8.238 1.00 1.00

ATOM 829 CG TYR A 115 -19.941 23.477 8.786 1.00 1.00

ATOM 830 CD1 TYR A 115 -20.360 24.667 8.189 1.00 1.00

ATOM 831 CD2 TYR A 115 -20.639 23.018 9.903 1.00 1.00

ATOM 832 CE1 TYR A 115 -21.455 25.372 8.684 1.00 1.00

ATOM 833 CE2 TYR A 115 -21.731 23.720 10.405 1.00 1.00

ATOM 834 CZ TYR A 115 -22.135 24.890 9.793 1.00 1.00

ATOM 835 OH TYR A 115 -23.215 25.572 10.294 1.00 1.00

ATOM 836 N ILE A 116 -16.697 20.180 8.919 1.00 1.00

ATOM 837 CA ILE A 116 -15.856 19.208 8.249 1.00 1.00

ATOM 838 C ILE A 116 -16.785 18.428 7.320 1.00 1.00

ATOM 839 O ILE A 116 -17.769 17.841 7.766 1.00 1.00

ATOM 840 CB ILE A 116 -15.125 18.285 9.247 1.00 1.00

ATOM 841 CG1 ILE A 116 -14.244 19.126 10.186 1.00 1.00

ATOM 842 CG2 ILE A 116 -14.308 17.223 8.498 1.00 1.00

ATOM 843 CD1 ILE A 116 -13.553 18.340 11.301 1.00 1.00

ATOM 844 N THR A 117 -16.478 18.453 6.027 1.00 1.00

ATOM 845 CA THR A 117 -17.430 17.993 5.021 1.00 1.00

ATOM 846 C THR A 117 -16.856 16.944 4.080 1.00 1.00

ATOM 847 O THR A 117 -15.689 17.015 3.690 1.00 1.00

ATOM 848 CB THR A 117 -17.948 19.174 4.165 1.00 1.00

ATOM 849 OG1 THR A 117 -16.836 19.932 3.689 1.00 1.00

ATOM 850 CG2 THR A 117 -18.861 20.092 4.975 1.00 1.00

ATOM 851 N PHE A 118 -17.700 15.988 3.699 1.00 1.00

ATOM 852 CA PHE A 118 -17.349 15.009 2.675 1.00 1.00

ATOM 853 C PHE A 118 -17.029 15.698 1.347 1.00 1.00

ATOM 854 O PHE A 118 -17.840 16.465 0.821 1.00 1.00

ATOM 855 CB PHE A 118 -18.483 13.993 2.493 1.00 1.00

ATOM 856 CG PHE A 118 -18.403 13.221 1.204 1.00 1.00

ATOM 857 CD1 PHE A 118 -17.368 12.313 0.983 1.00 1.00

ATOM 858 CD2 PHE A 118 -19.358 13.406 0.210 1.00 1.00

ATOM 859 CE1 PHE A 118 -17.287 11.602 -0.206 1.00 1.00

ATOM 860 CE2 PHE A 118 -19.285 12.693 -0.989 1.00 1.00

ATOM 861 CZ PHE A 118 -18.247 11.792 -1.196 1.00 1.00

ATOM 862 N ASP A 119 -15.841 15.418 0.815 1.00 1.00

ATOM 863 CA ASP A 119 -15.395 16.004 -0.450 1.00 1.00

ATOM 864 C ASP A 119 -15.404 14.950 -1.561 1.00 1.00

ATOM 865 O ASP A 119 -16.088 15.107 -2.566 1.00 1.00

ATOM 866 CB ASP A 119 -13.996 16.620 -0.284 1.00 1.00

ATOM 867 CG ASP A 119 -13.480 17.304 -1.551 1.00 1.00

ATOM 868 OD1 ASP A 119 -14.240 17.499 -2.525 1.00 1.00

ATOM 869 OD2 ASP A 119 -12.287 17.661 -1.569 1.00 1.00

ATOM 870 N GLU A 120 -14.648 13.875 -1.360 1.00 1.00

ATOM 871 CA GLU A 120 -14.507 12.826 -2.365 1.00 1.00

ATOM 872 C GLU A 120 -14.316 11.469 -1.696 1.00 1.00

ATOM 873 O GLU A 120 -13.770 11.387 -0.598 1.00 1.00

ATOM 874 CB GLU A 120 -13.312 13.136 -3.273 1.00 1.00

ATOM 875 CG GLU A 120 -13.398 12.531 -4.668 1.00 1.00

ATOM 876 CD GLU A 120 -12.481 13.222 -5.674 1.00 1.00

ATOM 877 OE1 GLU A 120 -11.446 13.791 -5.255 1.00 1.00

ATOM 878 OE2 GLU A 120 -12.801 13.192 -6.888 1.00 1.00

ATOM 879 N VAL A 121 -14.786 10.414 -2.356 1.00 1.00

ATOM 880 CA VAL A 121 -14.528 9.044 -1.920 1.00 1.00

ATOM 881 C VAL A 121 -13.950 8.235 -3.080 1.00 1.00

ATOM 882 O VAL A 121 -14.392 8.368 -4.222 1.00 1.00

ATOM 883 CB VAL A 121 -15.800 8.355 -1.313 1.00 1.00

ATOM 884 CG1 VAL A 121 -16.993 8.387 -2.286 1.00 1.00

ATOM 885 CG2 VAL A 121 -15.500 6.923 -0.863 1.00 1.00

ATOM 886 N GLY A 122 -12.937 7.426 -2.783 1.00 1.00

ATOM 887 CA GLY A 122 -12.387 6.494 -3.756 1.00 1.00

ATOM 888 C GLY A 122 -13.150 5.186 -3.705 1.00 1.00

ATOM 889 O GLY A 122 -14.222 5.058 -4.300 1.00 1.00

ATOM 890 N GLY A 123 -12.600 4.217 -2.979 1.00 1.00

ATOM 891 CA GLY A 123 -13.261 2.926 -2.810 1.00 1.00

ATOM 892 C GLY A 123 -13.375 2.480 -1.366 1.00 1.00

ATOM 893 O GLY A 123 -12.483 2.735 -0.567 1.00 1.00

ATOM 894 N PHE A 124 -14.497 1.836 -1.049 1.00 1.00

ATOM 895 CA PHE A 124 -14.733 1.161 0.227 1.00 1.00

ATOM 896 C PHE A 124 -14.984 -0.294 -0.138 1.00 1.00

ATOM 897 O PHE A 124 -16.048 -0.611 -0.666 1.00 1.00

ATOM 898 CB PHE A 124 -16.011 1.700 0.892 1.00 1.00

ATOM 899 CG PHE A 124 -15.783 2.676 2.014 1.00 1.00

ATOM 900 CD1 PHE A 124 -15.476 4.009 1.753 1.00 1.00

ATOM 901 CD2 PHE A 124 -15.939 2.275 3.340 1.00 1.00

ATOM 902 CE1 PHE A 124 -15.292 4.920 2.790 1.00 1.00

ATOM 903 CE2 PHE A 124 -15.765 3.177 4.390 1.00 1.00

ATOM 904 CZ PHE A 124 -15.441 4.505 4.112 1.00 1.00

ATOM 905 N ALA A 125 -14.027 -1.180 0.121 1.00 1.00

ATOM 906 CA ALA A 125 -14.181 -2.583 -0.273 1.00 1.00

ATOM 907 C ALA A 125 -14.137 -3.552 0.904 1.00 1.00

ATOM 908 O ALA A 125 -13.491 -3.282 1.916 1.00 1.00

ATOM 909 CB ALA A 125 -13.135 -2.964 -1.323 1.00 1.00

ATOM 910 N ASP A 126 -14.832 -4.678 0.765 1.00 1.00

ATOM 911 CA ASP A 126 -14.754 -5.751 1.759 1.00 1.00

ATOM 912 C ASP A 126 -13.566 -6.668 1.476 1.00 1.00

ATOM 913 O ASP A 126 -12.788 -6.411 0.548 1.00 1.00

ATOM 914 CB ASP A 126 -16.082 -6.531 1.867 1.00 1.00

ATOM 915 CG ASP A 126 -16.420 -7.340 0.609 1.00 1.00

ATOM 916 OD1 ASP A 126 -15.562 -7.536 -0.277 1.00 1.00

ATOM 917 OD2 ASP A 126 -17.575 -7.798 0.518 1.00 1.00

ATOM 918 N ASN A 127 -13.436 -7.736 2.260 1.00 1.00

ATOM 919 CA ASN A 127 -12.351 -8.701 2.079 1.00 1.00

ATOM 920 C ASN A 127 -12.382 -9.498 0.770 1.00 1.00

ATOM 921 O ASN A 127 -11.392 -10.132 0.416 1.00 1.00

ATOM 922 CB ASN A 127 -12.252 -9.646 3.280 1.00 1.00

ATOM 923 CG ASN A 127 -11.527 -9.019 4.449 1.00 1.00

ATOM 924 OD1 ASN A 127 -10.492 -8.370 4.276 1.00 1.00

ATOM 925 ND2 ASN A 127 -12.060 -9.212 5.653 1.00 1.00

ATOM 926 N ASP A 128 -13.512 -9.458 0.062 1.00 1.00

ATOM 927 CA ASP A 128 -13.612 -10.085 -1.265 1.00 1.00

ATOM 928 C ASP A 128 -13.403 -9.069 -2.389 1.00 1.00

ATOM 929 O ASP A 128 -13.613 -9.378 -3.564 1.00 1.00

ATOM 930 CB ASP A 128 -14.957 -10.797 -1.432 1.00 1.00

ATOM 931 CG ASP A 128 -15.024 -12.116 -0.674 1.00 1.00

ATOM 932 OD1 ASP A 128 -14.061 -12.457 0.041 1.00 1.00

ATOM 933 OD2 ASP A 128 -16.054 -12.813 -0.798 1.00 1.00

ATOM 934 N LEU A 129 -12.986 -7.862 -2.005 1.00 1.00

ATOM 935 CA LEU A 129 -12.761 -6.736 -2.923 1.00 1.00

ATOM 936 C LEU A 129 -14.049 -6.224 -3.571 1.00 1.00

ATOM 937 O LEU A 129 -14.009 -5.524 -4.587 1.00 1.00

ATOM 938 CB LEU A 129 -11.709 -7.073 -3.998 1.00 1.00

ATOM 939 CG LEU A 129 -10.308 -7.519 -3.571 1.00 1.00

ATOM 940 CD1 LEU A 129 -9.348 -7.364 -4.743 1.00 1.00

ATOM 941 CD2 LEU A 129 -9.803 -6.741 -2.379 1.00 1.00

ATOM 942 N VAL A 130 -15.186 -6.571 -2.977 1.00 1.00

ATOM 943 CA VAL A 130 -16.475 -6.066 -3.432 1.00 1.00

ATOM 944 C VAL A 130 -16.655 -4.655 -2.888 1.00 1.00

ATOM 945 O VAL A 130 -16.685 -4.448 -1.667 1.00 1.00

ATOM 946 CB VAL A 130 -17.651 -6.977 -2.992 1.00 1.00

ATOM 947 CG1 VAL A 130 -18.992 -6.409 -3.464 1.00 1.00

ATOM 948 CG2 VAL A 130 -17.459 -8.395 -3.520 1.00 1.00

ATOM 949 N GLU A 131 -16.755 -3.690 -3.801 1.00 1.00

ATOM 950 CA GLU A 131 -16.960 -2.284 -3.454 1.00 1.00

ATOM 951 C GLU A 131 -18.334 -2.067 -2.836 1.00 1.00

ATOM 952 O GLU A 131 -19.321 -2.636 -3.302 1.00 1.00

ATOM 953 CB GLU A 131 -16.811 -1.399 -4.697 1.00 1.00

ATOM 954 CG GLU A 131 -15.383 -1.288 -5.225 1.00 1.00

ATOM 955 CD GLU A 131 -14.572 -0.214 -4.528 1.00 1.00

ATOM 956 OE1 GLU A 131 -15.038 0.340 -3.512 1.00 1.00

ATOM 957 OE2 GLU A 131 -13.459 0.085 -5.008 1.00 1.00

ATOM 958 N GLN A 132 -18.389 -1.235 -1.804 1.00 1.00

ATOM 959 CA GLN A 132 -19.613 -1.016 -1.054 1.00 1.00

ATOM 960 C GLN A 132 -20.286 0.298 -1.429 1.00 1.00

ATOM 961 O GLN A 132 -19.629 1.255 -1.850 1.00 1.00

ATOM 962 CB GLN A 132 -19.306 -1.033 0.449 1.00 1.00

ATOM 963 CG GLN A 132 -18.658 -2.322 0.947 1.00 1.00

ATOM 964 CD GLN A 132 -19.629 -3.486 0.990 1.00 1.00

ATOM 965 OE1 GLN A 132 -20.667 -3.415 1.646 1.00 1.00

ATOM 966 NE2 GLN A 132 -19.293 -4.564 0.297 1.00 1.00

ATOM 967 N LYS A 133 -21.610 0.321 -1.289 1.00 1.00

ATOM 968 CA LYS A 133 -22.364 1.561 -1.326 1.00 1.00

ATOM 969 C LYS A 133 -21.980 2.319 -0.058 1.00 1.00

ATOM 970 O LYS A 133 -21.901 1.728 1.021 1.00 1.00

ATOM 971 CB LYS A 133 -23.862 1.264 -1.338 1.00 1.00

ATOM 972 CG LYS A 133 -24.697 2.225 -2.171 1.00 1.00

ATOM 973 CD LYS A 133 -26.099 1.669 -2.435 1.00 1.00

ATOM 974 CE LYS A 133 -26.077 0.505 -3.435 1.00 1.00

ATOM 975 NZ LYS A 133 -27.450 0.050 -3.807 1.00 1.00

ATOM 976 N VAL A 134 -21.706 3.611 -0.193 1.00 1.00

ATOM 977 CA VAL A 134 -21.279 4.418 0.951 1.00 1.00

ATOM 978 C VAL A 134 -21.918 5.808 0.936 1.00 1.00

ATOM 979 O VAL A 134 -22.017 6.446 -0.115 1.00 1.00

ATOM 980 CB VAL A 134 -19.716 4.472 1.064 1.00 1.00

ATOM 981 CG1 VAL A 134 -19.077 4.979 -0.228 1.00 1.00

ATOM 982 CG2 VAL A 134 -19.262 5.287 2.281 1.00 1.00

ATOM 983 N SER A 135 -22.389 6.253 2.099 1.00 1.00

ATOM 984 CA SER A 135 -22.933 7.599 2.244 1.00 1.00

ATOM 985 C SER A 135 -22.335 8.307 3.460 1.00 1.00

ATOM 986 O SER A 135 -21.944 7.667 4.440 1.00 1.00

ATOM 987 CB SER A 135 -24.465 7.582 2.306 1.00 1.00

ATOM 988 OG SER A 135 -24.934 6.811 3.396 1.00 1.00

ATOM 989 N PHE A 136 -22.262 9.633 3.379 1.00 1.00

ATOM 990 CA PHE A 136 -21.585 10.431 4.393 1.00 1.00

ATOM 991 C PHE A 136 -22.536 11.395 5.081 1.00 1.00

ATOM 992 O PHE A 136 -23.462 11.912 4.459 1.00 1.00

ATOM 993 CB PHE A 136 -20.425 11.210 3.766 1.00 1.00

ATOM 994 CG PHE A 136 -19.297 10.338 3.280 1.00 1.00

ATOM 995 CD1 PHE A 136 -19.380 9.681 2.051 1.00 1.00

ATOM 996 CD2 PHE A 136 -18.151 10.179 4.047 1.00 1.00

ATOM 997 CE1 PHE A 136 -18.339 8.872 1.605 1.00 1.00

ATOM 998 CE2 PHE A 136 -17.105 9.377 3.611 1.00 1.00

ATOM 999 CZ PHE A 136 -17.197 8.720 2.388 1.00 1.00

ATOM 1000 N ILE A 137 -22.303 11.611 6.372 1.00 1.00

ATOM 1001 CA ILE A 137 -23.021 12.611 7.152 1.00 1.00

ATOM 1002 C ILE A 137 -21.992 13.619 7.661 1.00 1.00

ATOM 1003 O ILE A 137 -21.074 13.257 8.396 1.00 1.00

ATOM 1004 CB ILE A 137 -23.799 11.984 8.339 1.00 1.00

ATOM 1005 CG1 ILE A 137 -24.829 10.962 7.835 1.00 1.00

ATOM 1006 CG2 ILE A 137 -24.477 13.070 9.177 1.00 1.00

ATOM 1007 CD1 ILE A 137 -25.422 10.084 8.928 1.00 1.00

ATOM 1008 N ASP A 138 -22.141 14.875 7.243 1.00 1.00

ATOM 1009 CA ASP A 138 -21.204 15.931 7.607 1.00 1.00

ATOM 1010 C ASP A 138 -21.381 16.394 9.055 1.00 1.00

ATOM 1011 O ASP A 138 -22.447 16.219 9.645 1.00 1.00

ATOM 1012 CB ASP A 138 -21.329 17.115 6.637 1.00 1.00

ATOM 1013 CG ASP A 138 -20.879 16.768 5.220 1.00 1.00

ATOM 1014 OD1 ASP A 138 -20.219 15.727 5.031 1.00 1.00

ATOM 1015 OD2 ASP A 138 -21.174 17.551 4.294 1.00 1.00

ATOM 1016 N GLY A 139 -20.324 16.983 9.614 1.00 1.00

ATOM 1017 CA GLY A 139 -20.358 17.509 10.974 1.00 1.00

ATOM 1018 C GLY A 139 -19.297 18.567 11.197 1.00 1.00

ATOM 1019 O GLY A 139 -19.075 19.430 10.338 1.00 1.00

ATOM 1020 N GLY A 140 -18.635 18.498 12.347 1.00 1.00

ATOM 1021 CA GLY A 140 -17.575 19.448 12.680 1.00 1.00

ATOM 1022 C GLY A 140 -17.788 20.154 14.008 1.00 1.00

ATOM 1023 O GLY A 140 -18.326 19.576 14.950 1.00 1.00

ATOM 1024 N VAL A 141 -17.375 21.417 14.069 1.00 1.00

ATOM 1025 CA VAL A 141 -17.278 22.137 15.338 1.00 1.00

ATOM 1026 C VAL A 141 -18.228 23.338 15.397 1.00 1.00

ATOM 1027 O VAL A 141 -18.346 24.095 14.427 1.00 1.00

ATOM 1028 CB VAL A 141 -15.809 22.584 15.607 1.00 1.00

ATOM 1029 CG1 VAL A 141 -15.664 23.217 16.989 1.00 1.00

ATOM 1030 CG2 VAL A 141 -14.852 21.394 15.469 1.00 1.00

ATOM 1031 N ASN A 142 -18.903 23.495 16.539 1.00 1.00

ATOM 1032 CA ASN A 142 -19.809 24.625 16.777 1.00 1.00

ATOM 1033 C ASN A 142 -20.743 24.868 15.592 1.00 1.00

ATOM 1034 O ASN A 142 -20.788 25.974 15.042 1.00 1.00

ATOM 1035 CB ASN A 142 -19.010 25.901 17.092 1.00 1.00

ATOM 1036 CG ASN A 142 -18.152 25.774 18.341 1.00 1.00

ATOM 1037 OD1 ASN A 142 -18.587 25.231 19.354 1.00 1.00

ATOM 1038 ND2 ASN A 142 -16.933 26.298 18.276 1.00 1.00

ATOM 1039 N VAL A 143 -21.488 23.834 15.210 1.00 1.00

ATOM 1040 CA VAL A 143 -22.252 23.845 13.956 1.00 1.00

ATOM 1041 C VAL A 143 -23.422 24.837 13.932 1.00 1.00

ATOM 1042 O VAL A 143 -23.884 25.222 12.858 1.00 1.00

ATOM 1043 CB VAL A 143 -22.746 22.417 13.540 1.00 1.00

ATOM 1044 CG1 VAL A 143 -21.553 21.473 13.311 1.00 1.00

ATOM 1045 CG2 VAL A 143 -23.732 21.837 14.556 1.00 1.00

ATOM 1046 N GLY A 144 -23.885 25.243 15.111 1.00 1.00

ATOM 1047 CA GLY A 144 -25.041 26.131 15.223 1.00 1.00

ATOM 1048 C GLY A 144 -24.716 27.585 15.511 1.00 1.00

ATOM 1049 O GLY A 144 -25.623 28.397 15.683 1.00 1.00

ATOM 1050 OXT GLY A 144 -23.507 27.919 15.535 1.00 1.00

TER

ATOM 1051 N GLY B 1 -1.205 2.688 -11.497 1.00 1.00

ATOM 1052 CA GLY B 1 -1.951 2.715 -10.205 1.00 1.00

ATOM 1053 C GLY B 1 -3.273 1.968 -10.250 1.00 1.00

ATOM 1054 O GLY B 1 -3.701 1.389 -9.249 1.00 1.00

ATOM 1055 N ASP B 2 -3.913 1.985 -11.418 1.00 1.00

ATOM 1056 CA ASP B 2 -5.215 1.352 -11.620 1.00 1.00

ATOM 1057 C ASP B 2 -5.067 -0.160 -11.819 1.00 1.00

ATOM 1058 O ASP B 2 -5.140 -0.660 -12.944 1.00 1.00

ATOM 1059 CB ASP B 2 -5.930 2.000 -12.814 1.00 1.00

ATOM 1060 CG ASP B 2 -7.396 1.605 -12.918 1.00 1.00

ATOM 1061 OD1 ASP B 2 -7.961 1.069 -11.937 1.00 1.00

ATOM 1062 OD2 ASP B 2 -8.069 1.798 -13.950 1.00 1.00

ATOM 1063 N VAL B 3 -4.870 -0.872 -10.711 1.00 1.00

ATOM 1064 CA VAL B 3 -4.589 -2.311 -10.722 1.00 1.00

ATOM 1065 C VAL B 3 -5.650 -3.137 -11.460 1.00 1.00

ATOM 1066 O VAL B 3 -5.322 -3.861 -12.401 1.00 1.00

ATOM 1067 CB VAL B 3 -4.373 -2.864 -9.280 1.00 1.00

ATOM 1068 CG1 VAL B 3 -4.082 -4.358 -9.304 1.00 1.00

ATOM 1069 CG2 VAL B 3 -3.241 -2.120 -8.584 1.00 1.00

ATOM 1070 N ASN B 4 -6.911 -3.022 -11.044 1.00 1.00

ATOM 1071 CA ASN B 4 -7.993 -3.799 -11.654 1.00 1.00

ATOM 1072 C ASN B 4 -8.470 -3.263 -13.008 1.00 1.00

ATOM 1073 O ASN B 4 -9.152 -3.968 -13.755 1.00 1.00

ATOM 1074 CB ASN B 4 -9.177 -3.978 -10.691 1.00 1.00

ATOM 1075 CG ASN B 4 -9.644 -2.670 -10.070 1.00 1.00

ATOM 1076 OD1 ASN B 4 -9.642 -1.619 -10.714 1.00 1.00

ATOM 1077 ND2 ASN B 4 -10.051 -2.736 -8.809 1.00 1.00

ATOM 1078 N GLY B 5 -8.110 -2.018 -13.310 1.00 1.00

ATOM 1079 CA GLY B 5 -8.416 -1.404 -14.591 1.00 1.00

ATOM 1080 C GLY B 5 -9.861 -0.973 -14.779 1.00 1.00

ATOM 1081 O GLY B 5 -10.371 -1.003 -15.901 1.00 1.00

ATOM 1082 N ASP B 6 -10.518 -0.563 -13.694 1.00 1.00

ATOM 1083 CA ASP B 6 -11.914 -0.129 -13.762 1.00 1.00

ATOM 1084 C ASP B 6 -12.087 1.362 -14.070 1.00 1.00

ATOM 1085 O ASP B 6 -13.213 1.844 -14.223 1.00 1.00

ATOM 1086 CB ASP B 6 -12.695 -0.538 -12.496 1.00 1.00

ATOM 1087 CG ASP B 6 -12.179 0.132 -11.226 1.00 1.00

ATOM 1088 OD1 ASP B 6 -11.283 1.001 -11.298 1.00 1.00

ATOM 1089 OD2 ASP B 6 -12.616 -0.156 -10.092 1.00 1.00

ATOM 1090 N GLY B 7 -10.972 2.085 -14.158 1.00 1.00

ATOM 1091 CA GLY B 7 -10.980 3.493 -14.524 1.00 1.00

ATOM 1092 C GLY B 7 -10.768 4.456 -13.369 1.00 1.00

ATOM 1093 O GLY B 7 -10.629 5.665 -13.583 1.00 1.00

ATOM 1094 N THR B 8 -10.743 3.924 -12.149 1.00 1.00

ATOM 1095 CA THR B 8 -10.569 4.735 -10.948 1.00 1.00

ATOM 1096 C THR B 8 -9.468 4.159 -10.062 1.00 1.00

ATOM 1097 O THR B 8 -9.388 2.943 -9.872 1.00 1.00

ATOM 1098 CB THR B 8 -11.895 4.820 -10.152 1.00 1.00

ATOM 1099 OG1 THR B 8 -12.987 5.053 -11.051 1.00 1.00

ATOM 1100 CG2 THR B 8 -11.911 6.055 -9.255 1.00 1.00

ATOM 1101 N ILE B 9 -8.622 5.035 -9.530 1.00 1.00

ATOM 1102 CA ILE B 9 -7.585 4.630 -8.584 1.00 1.00

ATOM 1103 C ILE B 9 -8.069 4.889 -7.158 1.00 1.00

ATOM 1104 O ILE B 9 -8.195 6.039 -6.732 1.00 1.00

ATOM 1105 CB ILE B 9 -6.243 5.356 -8.867 1.00 1.00

ATOM 1106 CG1 ILE B 9 -5.783 5.106 -10.308 1.00 1.00

ATOM 1107 CG2 ILE B 9 -5.164 4.898 -7.888 1.00 1.00

ATOM 1108 CD1 ILE B 9 -5.037 6.271 -10.936 1.00 1.00

ATOM 1109 N ASN B 10 -8.352 3.806 -6.435 1.00 1.00

ATOM 1110 CA ASN B 10 -8.835 3.889 -5.057 1.00 1.00

ATOM 1111 C ASN B 10 -8.246 2.806 -4.147 1.00 1.00

ATOM 1112 O ASN B 10 -7.278 2.135 -4.516 1.00 1.00

ATOM 1113 CB ASN B 10 -10.373 3.877 -5.009 1.00 1.00

ATOM 1114 CG ASN B 10 -10.990 2.739 -5.813 1.00 1.00

ATOM 1115 OD1 ASN B 10 -10.446 1.635 -5.882 1.00 1.00

ATOM 1116 ND2 ASN B 10 -12.139 3.007 -6.421 1.00 1.00

ATOM 1117 N SER B 11 -8.837 2.646 -2.963 1.00 1.00

ATOM 1118 CA SER B 11 -8.358 1.704 -1.948 1.00 1.00

ATOM 1119 C SER B 11 -8.372 0.241 -2.405 1.00 1.00

ATOM 1120 O SER B 11 -7.553 -0.562 -1.950 1.00 1.00

ATOM 1121 CB SER B 11 -9.164 1.859 -0.657 1.00 1.00

ATOM 1122 OG SER B 11 -10.540 1.607 -0.885 1.00 1.00

ATOM 1123 N THR B 12 -9.301 -0.091 -3.302 1.00 1.00

ATOM 1124 CA THR B 12 -9.419 -1.441 -3.860 1.00 1.00

ATOM 1125 C THR B 12 -8.161 -1.854 -4.635 1.00 1.00

ATOM 1126 O THR B 12 -7.777 -3.026 -4.622 1.00 1.00

ATOM 1127 CB THR B 12 -10.674 -1.549 -4.761 1.00 1.00

ATOM 1128 OG1 THR B 12 -11.821 -1.060 -4.051 1.00 1.00

ATOM 1129 CG2 THR B 12 -11.035 -3.008 -5.033 1.00 1.00

ATOM 1130 N ASP B 13 -7.534 -0.887 -5.303 1.00 1.00

ATOM 1131 CA ASP B 13 -6.290 -1.124 -6.040 1.00 1.00

ATOM 1132 C ASP B 13 -5.124 -1.423 -5.106 1.00 1.00

ATOM 1133 O ASP B 13 -4.349 -2.348 -5.353 1.00 1.00

ATOM 1134 CB ASP B 13 -5.948 0.075 -6.927 1.00 1.00

ATOM 1135 CG ASP B 13 -6.938 0.266 -8.052 1.00 1.00

ATOM 1136 OD1 ASP B 13 -7.031 -0.617 -8.935 1.00 1.00

ATOM 1137 OD2 ASP B 13 -7.679 1.269 -8.140 1.00 1.00

ATOM 1138 N LEU B 14 -5.006 -0.632 -4.040 1.00 1.00

ATOM 1139 CA LEU B 14 -3.948 -0.813 -3.050 1.00 1.00

ATOM 1140 C LEU B 14 -4.070 -2.162 -2.342 1.00 1.00

ATOM 1141 O LEU B 14 -3.068 -2.847 -2.135 1.00 1.00

ATOM 1142 CB LEU B 14 -3.940 0.339 -2.035 1.00 1.00

ATOM 1143 CG LEU B 14 -2.821 0.371 -0.985 1.00 1.00

ATOM 1144 CD1 LEU B 14 -1.449 0.608 -1.616 1.00 1.00

ATOM 1145 CD2 LEU B 14 -3.112 1.422 0.080 1.00 1.00

ATOM 1146 N THR B 15 -5.301 -2.533 -1.989 1.00 1.00

ATOM 1147 CA THR B 15 -5.588 -3.821 -1.357 1.00 1.00

ATOM 1148 C THR B 15 -5.166 -4.991 -2.249 1.00 1.00

ATOM 1149 O THR B 15 -4.556 -5.947 -1.771 1.00 1.00

ATOM 1150 CB THR B 15 -7.088 -3.922 -0.990 1.00 1.00

ATOM 1151 OG1 THR B 15 -7.441 -2.840 -0.122 1.00 1.00

ATOM 1152 CG2 THR B 15 -7.359 -5.155 -0.127 1.00 1.00

ATOM 1153 N MET B 16 -5.490 -4.900 -3.538 1.00 1.00

ATOM 1154 CA MET B 16 -5.106 -5.918 -4.517 1.00 1.00

ATOM 1155 C MET B 16 -3.590 -5.997 -4.685 1.00 1.00

ATOM 1156 O MET B 16 -3.029 -7.088 -4.792 1.00 1.00

ATOM 1157 CB MET B 16 -5.759 -5.635 -5.871 1.00 1.00

ATOM 1158 CG MET B 16 -7.161 -6.198 -6.018 1.00 1.00

ATOM 1159 SD MET B 16 -7.717 -6.171 -7.733 1.00 1.00

ATOM 1160 CE MET B 16 -9.470 -5.880 -7.498 1.00 1.00

ATOM 1161 N LEU B 17 -2.940 -4.834 -4.705 1.00 1.00

ATOM 1162 CA LEU B 17 -1.490 -4.744 -4.848 1.00 1.00

ATOM 1163 C LEU B 17 -0.760 -5.327 -3.635 1.00 1.00

ATOM 1164 O LEU B 17 0.199 -6.085 -3.792 1.00 1.00

ATOM 1165 CB LEU B 17 -1.058 -3.289 -5.095 1.00 1.00

ATOM 1166 CG LEU B 17 0.439 -2.954 -5.153 1.00 1.00

ATOM 1167 CD1 LEU B 17 1.128 -3.649 -6.319 1.00 1.00

ATOM 1168 CD2 LEU B 17 0.653 -1.452 -5.234 1.00 1.00

ATOM 1169 N LYS B 18 -1.225 -4.969 -2.440 1.00 1.00

ATOM 1170 CA LYS B 18 -0.629 -5.446 -1.192 1.00 1.00

ATOM 1171 C LYS B 18 -0.792 -6.959 -1.026 1.00 1.00

ATOM 1172 O LYS B 18 0.138 -7.642 -0.593 1.00 1.00

ATOM 1173 CB LYS B 18 -1.232 -4.712 0.010 1.00 1.00

ATOM 1174 CG LYS B 18 -0.667 -3.311 0.227 1.00 1.00

ATOM 1175 CD LYS B 18 -1.432 -2.553 1.301 1.00 1.00

ATOM 1176 CE LYS B 18 -0.672 -2.538 2.617 1.00 1.00

ATOM 1177 NZ LYS B 18 -1.370 -1.732 3.662 1.00 1.00

ATOM 1178 N ARG B 19 -1.971 -7.470 -1.380 1.00 1.00

ATOM 1179 CA ARG B 19 -2.252 -8.904 -1.324 1.00 1.00

ATOM 1180 C ARG B 19 -1.425 -9.682 -2.348 1.00 1.00

ATOM 1181 O ARG B 19 -1.037 -10.824 -2.099 1.00 1.00

ATOM 1182 CB ARG B 19 -3.748 -9.171 -1.524 1.00 1.00

ATOM 1183 CG ARG B 19 -4.583 -8.952 -0.266 1.00 1.00

ATOM 1184 CD ARG B 19 -6.099 -8.995 -0.485 1.00 1.00

ATOM 1185 NE ARG B 19 -6.570 -10.287 -0.990 1.00 1.00

ATOM 1186 CZ ARG B 19 -6.664 -11.404 -0.269 1.00 1.00

ATOM 1187 NH1 ARG B 19 -6.318 -11.417 1.014 1.00 1.00

ATOM 1188 NH2 ARG B 19 -7.108 -12.517 -0.835 1.00 1.00

ATOM 1189 N SER B 20 -1.155 -9.047 -3.489 1.00 1.00

ATOM 1190 CA SER B 20 -0.303 -9.621 -4.531 1.00 1.00

ATOM 1191 C SER B 20 1.147 -9.779 -4.065 1.00 1.00

ATOM 1192 O SER B 20 1.763 -10.823 -4.291 1.00 1.00

ATOM 1193 CB SER B 20 -0.365 -8.768 -5.805 1.00 1.00

ATOM 1194 OG SER B 20 0.706 -9.071 -6.683 1.00 1.00

ATOM 1195 N VAL B 21 1.678 -8.737 -3.427 1.00 1.00

ATOM 1196 CA VAL B 21 3.041 -8.757 -2.885 1.00 1.00

ATOM 1197 C VAL B 21 3.151 -9.780 -1.747 1.00 1.00

ATOM 1198 O VAL B 21 4.159 -10.479 -1.624 1.00 1.00

ATOM 1199 CB VAL B 21 3.496 -7.350 -2.410 1.00 1.00

ATOM 1200 CG1 VAL B 21 4.964 -7.353 -1.988 1.00 1.00

ATOM 1201 CG2 VAL B 21 3.274 -6.313 -3.505 1.00 1.00

ATOM 1202 N LEU B 22 2.098 -9.876 -0.937 1.00 1.00

ATOM 1203 CA LEU B 22 2.031 -10.868 0.134 1.00 1.00

ATOM 1204 C LEU B 22 1.698 -12.270 -0.388 1.00 1.00

ATOM 1205 O LEU B 22 1.589 -13.221 0.391 1.00 1.00

ATOM 1206 CB LEU B 22 1.029 -10.434 1.212 1.00 1.00

ATOM 1207 CG LEU B 22 1.513 -9.339 2.170 1.00 1.00

ATOM 1208 CD1 LEU B 22 0.336 -8.593 2.778 1.00 1.00

ATOM 1209 CD2 LEU B 22 2.405 -9.914 3.263 1.00 1.00

ATOM 1210 N ARG B 23 1.543 -12.381 -1.709 1.00 1.00

ATOM 1211 CA ARG B 23 1.314 -13.653 -2.410 1.00 1.00

ATOM 1212 C ARG B 23 -0.016 -14.335 -2.056 1.00 1.00

ATOM 1213 O ARG B 23 -0.155 -15.552 -2.205 1.00 1.00

ATOM 1214 CB ARG B 23 2.498 -14.622 -2.221 1.00 1.00

ATOM 1215 CG ARG B 23 3.871 -14.016 -2.502 1.00 1.00

ATOM 1216 CD ARG B 23 4.483 -14.442 -3.823 1.00 1.00

ATOM 1217 NE ARG B 23 5.805 -13.855 -4.035 1.00 1.00

ATOM 1218 CZ ARG B 23 6.910 -14.555 -4.280 1.00 1.00

ATOM 1219 NH1 ARG B 23 6.867 -15.881 -4.342 1.00 1.00

ATOM 1220 NH2 ARG B 23 8.065 -13.927 -4.460 1.00 1.00

ATOM 1221 N ALA B 24 -0.983 -13.549 -1.592 1.00 1.00

ATOM 1222 CA ALA B 24 -2.321 -14.058 -1.298 1.00 1.00

ATOM 1223 C ALA B 24 -3.108 -14.281 -2.584 1.00 1.00

ATOM 1224 O ALA B 24 -3.893 -15.222 -2.691 1.00 1.00

ATOM 1225 CB ALA B 24 -3.065 -13.105 -0.371 1.00 1.00

ATOM 1226 N ILE B 25 -2.881 -13.403 -3.559 1.00 1.00

ATOM 1227 CA ILE B 25 -3.550 -13.470 -4.854 1.00 1.00

ATOM 1228 C ILE B 25 -2.540 -13.397 -5.994 1.00 1.00

ATOM 1229 O ILE B 25 -1.384 -13.012 -5.794 1.00 1.00

ATOM 1230 CB ILE B 25 -4.596 -12.324 -5.001 1.00 1.00

ATOM 1231 CG1 ILE B 25 -3.930 -10.947 -4.903 1.00 1.00

ATOM 1232 CG2 ILE B 25 -5.722 -12.468 -3.976 1.00 1.00

ATOM 1233 CD1 ILE B 25 -4.446 -9.943 -5.916 1.00 1.00

ATOM 1234 N THR B 26 -2.983 -13.781 -7.187 1.00 1.00

ATOM 1235 CA THR B 26 -2.220 -13.527 -8.404 1.00 1.00

ATOM 1236 C THR B 26 -2.933 -12.453 -9.213 1.00 1.00

ATOM 1237 O THR B 26 -4.164 -12.355 -9.190 1.00 1.00

ATOM 1238 CB THR B 26 -2.053 -14.805 -9.254 1.00 1.00

ATOM 1239 OG1 THR B 26 -3.336 -15.379 -9.524 1.00 1.00

ATOM 1240 CG2 THR B 26 -1.322 -15.894 -8.469 1.00 1.00

ATOM 1241 N LEU B 27 -2.151 -11.642 -9.913 1.00 1.00

ATOM 1242 CA LEU B 27 -2.707 -10.656 -10.827 1.00 1.00

ATOM 1243 C LEU B 27 -2.598 -11.168 -12.256 1.00 1.00

ATOM 1244 O LEU B 27 -1.667 -11.907 -12.589 1.00 1.00

ATOM 1245 CB LEU B 27 -1.986 -9.313 -10.686 1.00 1.00

ATOM 1246 CG LEU B 27 -2.065 -8.597 -9.333 1.00 1.00

ATOM 1247 CD1 LEU B 27 -1.039 -7.478 -9.277 1.00 1.00

ATOM 1248 CD2 LEU B 27 -3.464 -8.057 -9.059 1.00 1.00

ATOM 1249 N THR B 28 -3.559 -10.783 -13.090 1.00 1.00

ATOM 1250 CA THR B 28 -3.520 -11.108 -14.513 1.00 1.00

ATOM 1251 C THR B 28 -2.403 -10.310 -15.181 1.00 1.00

ATOM 1252 O THR B 28 -1.902 -9.337 -14.610 1.00 1.00

ATOM 1253 CB THR B 28 -4.877 -10.821 -15.188 1.00 1.00

ATOM 1254 OG1 THR B 28 -5.192 -9.427 -15.073 1.00 1.00

ATOM 1255 CG2 THR B 28 -6.015 -11.510 -14.437 1.00 1.00

ATOM 1256 N ASP B 29 -2.015 -10.726 -16.383 1.00 1.00

ATOM 1257 CA ASP B 29 -0.891 -10.110 -17.090 1.00 1.00

ATOM 1258 C ASP B 29 -1.094 -8.623 -17.377 1.00 1.00

ATOM 1259 O ASP B 29 -0.145 -7.844 -17.296 1.00 1.00

ATOM 1260 CB ASP B 29 -0.563 -10.889 -18.368 1.00 1.00

ATOM 1261 CG ASP B 29 -0.044 -12.291 -18.080 1.00 1.00

ATOM 1262 OD1 ASP B 29 0.596 -12.490 -17.026 1.00 1.00

ATOM 1263 OD2 ASP B 29 -0.228 -13.260 -18.847 1.00 1.00

ATOM 1264 N ASP B 30 -2.331 -8.234 -17.691 1.00 1.00

ATOM 1265 CA ASP B 30 -2.683 -6.824 -17.873 1.00 1.00

ATOM 1266 C ASP B 30 -2.555 -6.046 -16.567 1.00 1.00

ATOM 1267 O ASP B 30 -2.001 -4.943 -16.546 1.00 1.00

ATOM 1268 CB ASP B 30 -4.116 -6.682 -18.391 1.00 1.00

ATOM 1269 CG ASP B 30 -4.268 -7.094 -19.842 1.00 1.00

ATOM 1270 OD1 ASP B 30 -3.266 -7.444 -20.500 1.00 1.00

ATOM 1271 OD2 ASP B 30 -5.380 -7.103 -20.404 1.00 1.00

ATOM 1272 N ALA B 31 -3.085 -6.626 -15.492 1.00 1.00

ATOM 1273 CA ALA B 31 -3.064 -6.009 -14.165 1.00 1.00

ATOM 1274 C ALA B 31 -1.645 -5.863 -13.624 1.00 1.00

ATOM 1275 O ALA B 31 -1.345 -4.900 -12.918 1.00 1.00

ATOM 1276 CB ALA B 31 -3.925 -6.808 -13.195 1.00 1.00

ATOM 1277 N LYS B 32 -0.782 -6.820 -13.966 1.00 1.00

ATOM 1278 CA LYS B 32 0.623 -6.808 -13.560 1.00 1.00

ATOM 1279 C LYS B 32 1.357 -5.552 -14.032 1.00 1.00

ATOM 1280 O LYS B 32 2.140 -4.966 -13.279 1.00 1.00

ATOM 1281 CB LYS B 32 1.338 -8.059 -14.083 1.00 1.00

ATOM 1282 CG LYS B 32 1.183 -9.287 -13.201 1.00 1.00

ATOM 1283 CD LYS B 32 2.135 -10.392 -13.640 1.00 1.00

ATOM 1284 CE LYS B 32 1.857 -11.686 -12.896 1.00 1.00

ATOM 1285 NZ LYS B 32 1.759 -12.844 -13.830 1.00 1.00

ATOM 1286 N ALA B 33 1.099 -5.149 -15.274 1.00 1.00

ATOM 1287 CA ALA B 33 1.710 -3.950 -15.849 1.00 1.00

ATOM 1288 C ALA B 33 1.239 -2.678 -15.145 1.00 1.00

ATOM 1289 O ALA B 33 2.031 -1.764 -14.906 1.00 1.00

ATOM 1290 CB ALA B 33 1.428 -3.873 -17.347 1.00 1.00

ATOM 1291 N ARG B 34 -0.050 -2.633 -14.807 1.00 1.00

ATOM 1292 CA ARG B 34 -0.646 -1.484 -14.127 1.00 1.00

ATOM 1293 C ARG B 34 -0.264 -1.423 -12.644 1.00 1.00

ATOM 1294 O ARG B 34 -0.223 -0.339 -12.054 1.00 1.00

ATOM 1295 CB ARG B 34 -2.171 -1.490 -14.286 1.00 1.00

ATOM 1296 CG ARG B 34 -2.660 -1.444 -15.735 1.00 1.00

ATOM 1297 CD ARG B 34 -4.176 -1.393 -15.896 1.00 1.00

ATOM 1298 NE ARG B 34 -4.845 -2.510 -15.227 1.00 1.00

ATOM 1299 CZ ARG B 34 -5.507 -3.480 -15.852 1.00 1.00

ATOM 1300 NH1 ARG B 34 -5.603 -3.486 -17.177 1.00 1.00

ATOM 1301 NH2 ARG B 34 -6.078 -4.448 -15.150 1.00 1.00

ATOM 1302 N ALA B 35 0.005 -2.584 -12.052 1.00 1.00

ATOM 1303 CA ALA B 35 0.449 -2.673 -10.660 1.00 1.00

ATOM 1304 C ALA B 35 1.915 -2.263 -10.501 1.00 1.00

ATOM 1305 O ALA B 35 2.342 -1.864 -9.415 1.00 1.00

ATOM 1306 CB ALA B 35 0.233 -4.078 -10.121 1.00 1.00

ATOM 1307 N ASP B 36 2.677 -2.375 -11.587 1.00 1.00

ATOM 1308 CA ASP B 36 4.076 -1.962 -11.614 1.00 1.00

ATOM 1309 C ASP B 36 4.161 -0.434 -11.679 1.00 1.00

ATOM 1310 O ASP B 36 4.399 0.142 -12.743 1.00 1.00

ATOM 1311 CB ASP B 36 4.789 -2.613 -12.806 1.00 1.00

ATOM 1312 CG ASP B 36 6.310 -2.539 -12.710 1.00 1.00

ATOM 1313 OD1 ASP B 36 6.844 -1.946 -11.747 1.00 1.00

ATOM 1314 OD2 ASP B 36 7.055 -3.051 -13.567 1.00 1.00

ATOM 1315 N VAL B 37 3.962 0.206 -10.527 1.00 1.00

ATOM 1316 CA VAL B 37 3.861 1.667 -10.427 1.00 1.00

ATOM 1317 C VAL B 37 5.117 2.392 -10.931 1.00 1.00

ATOM 1318 O VAL B 37 5.013 3.344 -11.708 1.00 1.00

ATOM 1319 CB VAL B 37 3.512 2.123 -8.978 1.00 1.00

ATOM 1320 CG1 VAL B 37 3.410 3.646 -8.880 1.00 1.00

ATOM 1321 CG2 VAL B 37 2.214 1.479 -8.503 1.00 1.00

ATOM 1322 N ASP B 38 6.290 1.935 -10.496 1.00 1.00

ATOM 1323 CA ASP B 38 7.553 2.577 -10.879 1.00 1.00

ATOM 1324 C ASP B 38 8.169 2.004 -12.159 1.00 1.00

ATOM 1325 O ASP B 38 9.256 2.414 -12.572 1.00 1.00

ATOM 1326 CB ASP B 38 8.558 2.560 -9.715 1.00 1.00

ATOM 1327 CG ASP B 38 9.106 3.944 -9.375 1.00 1.00

ATOM 1328 OD1 ASP B 38 8.633 4.953 -9.943 1.00 1.00

ATOM 1329 OD2 ASP B 38 10.018 4.118 -8.539 1.00 1.00

ATOM 1330 N LYS B 39 7.518 1.048 -12.759 1.00 1.00

ATOM 1331 CA LYS B 39 7.798 0.407 -14.051 1.00 1.00

ATOM 1332 C LYS B 39 9.250 -0.103 -14.102 1.00 1.00

ATOM 1333 O LYS B 39 10.026 0.251 -15.004 1.00 1.00

ATOM 1334 CB LYS B 39 7.602 1.419 -15.191 1.00 1.00

ATOM 1335 CG LYS B 39 6.147 1.847 -15.374 1.00 1.00

ATOM 1336 CD LYS B 39 5.313 0.802 -16.112 1.00 1.00

ATOM 1337 CE LYS B 39 3.810 0.982 -15.900 1.00 1.00

ATOM 1338 NZ LYS B 39 3.006 0.090 -16.746 1.00 1.00

ATOM 1339 N ASN B 40 9.615 -0.941 -13.133 1.00 1.00

ATOM 1340 CA ASN B 40 10.987 -1.485 -13.093 1.00 1.00

ATOM 1341 C ASN B 40 11.001 -3.011 -13.378 1.00 1.00

ATOM 1342 O ASN B 40 12.037 -3.677 -13.248 1.00 1.00

ATOM 1343 CB ASN B 40 11.665 -1.199 -11.741 1.00 1.00

ATOM 1344 CG ASN B 40 11.049 -1.901 -10.536 1.00 1.00

ATOM 1345 OD1 ASN B 40 9.933 -2.408 -10.620 1.00 1.00

ATOM 1346 ND2 ASN B 40 11.730 -1.955 -9.406 1.00 1.00

ATOM 1347 N GLY B 41 9.843 -3.533 -13.770 1.00 1.00

ATOM 1348 CA GLY B 41 9.699 -4.963 -14.163 1.00 1.00

ATOM 1349 C GLY B 41 9.331 -5.912 -13.010 1.00 1.00

ATOM 1350 O GLY B 41 9.234 -7.133 -13.206 1.00 1.00

ATOM 1351 N SER B 42 9.132 -5.354 -11.836 1.00 1.00

ATOM 1352 CA SER B 42 8.773 -6.152 -10.640 1.00 1.00

ATOM 1353 C SER B 42 7.672 -5.491 -9.844 1.00 1.00

ATOM 1354 O SER B 42 7.570 -4.264 -9.800 1.00 1.00

ATOM 1355 CB SER B 42 9.961 -6.285 -9.706 1.00 1.00

ATOM 1356 OG SER B 42 10.930 -7.190 -10.219 1.00 1.00

ATOM 1357 N ILE B 43 6.864 -6.323 -9.219 1.00 1.00

ATOM 1358 CA ILE B 43 5.767 -5.839 -8.378 1.00 1.00

ATOM 1359 C ILE B 43 6.076 -6.149 -6.909 1.00 1.00

ATOM 1360 O ILE B 43 6.205 -7.315 -6.509 1.00 1.00

ATOM 1361 CB ILE B 43 4.445 -6.498 -8.766 1.00 1.00

ATOM 1362 CG1 ILE B 43 3.939 -6.037 -10.137 1.00 1.00

ATOM 1363 CG2 ILE B 43 3.320 -6.188 -7.771 1.00 1.00

ATOM 1364 CD1 ILE B 43 3.509 -7.190 -11.046 1.00 1.00

ATOM 1365 N ASN B 44 6.386 -5.076 -6.167 1.00 1.00

ATOM 1366 CA ASN B 44 6.892 -5.189 -4.799 1.00 1.00

ATOM 1367 C ASN B 44 6.469 -4.049 -3.861 1.00 1.00

ATOM 1368 O ASN B 44 5.586 -3.254 -4.196 1.00 1.00

ATOM 1369 CB ASN B 44 8.424 -5.366 -4.806 1.00 1.00

ATOM 1370 CG ASN B 44 9.175 -4.089 -5.177 1.00 1.00

ATOM 1371 OD1 ASN B 44 9.389 -3.211 -4.342 1.00 1.00

ATOM 1372 ND2 ASN B 44 9.593 -3.995 -6.430 1.00 1.00

ATOM 1373 N SER B 45 7.114 -3.981 -2.696 1.00 1.00

ATOM 1374 CA SER B 45 6.792 -3.004 -1.649 1.00 1.00

ATOM 1375 C SER B 45 7.049 -1.545 -2.041 1.00 1.00

ATOM 1376 O SER B 45 6.474 -0.633 -1.439 1.00 1.00

ATOM 1377 CB SER B 45 7.549 -3.334 -0.357 1.00 1.00

ATOM 1378 OG SER B 45 6.825 -4.248 0.445 1.00 1.00

ATOM 1379 N THR B 46 7.916 -1.324 -3.028 1.00 1.00

ATOM 1380 CA THR B 46 8.171 0.024 -3.540 1.00 1.00

ATOM 1381 C THR B 46 6.941 0.560 -4.275 1.00 1.00

ATOM 1382 O THR B 46 6.592 1.735 -4.125 1.00 1.00

ATOM 1383 CB THR B 46 9.427 0.053 -4.443 1.00 1.00

ATOM 1384 OG1 THR B 46 10.497 -0.648 -3.798 1.00 1.00

ATOM 1385 CG2 THR B 46 9.977 1.474 -4.567 1.00 1.00

ATOM 1386 N ASP B 47 6.288 -0.304 -5.054 1.00 1.00

ATOM 1387 CA ASP B 47 5.020 0.026 -5.707 1.00 1.00

ATOM 1388 C ASP B 47 3.926 0.298 -4.680 1.00 1.00

ATOM 1389 O ASP B 47 3.125 1.221 -4.846 1.00 1.00

ATOM 1390 CB ASP B 47 4.564 -1.107 -6.631 1.00 1.00

ATOM 1391 CG ASP B 47 5.602 -1.471 -7.667 1.00 1.00

ATOM 1392 OD1 ASP B 47 5.981 -0.598 -8.478 1.00 1.00

ATOM 1393 OD2 ASP B 47 6.102 -2.614 -7.752 1.00 1.00

ATOM 1394 N VAL B 48 3.905 -0.519 -3.625 1.00 1.00

ATOM 1395 CA VAL B 48 2.956 -0.373 -2.521 1.00 1.00

ATOM 1396 C VAL B 48 3.091 1.006 -1.870 1.00 1.00

ATOM 1397 O VAL B 48 2.095 1.710 -1.692 1.00 1.00

ATOM 1398 CB VAL B 48 3.140 -1.498 -1.460 1.00 1.00

ATOM 1399 CG1 VAL B 48 2.324 -1.210 -0.203 1.00 1.00

ATOM 1400 CG2 VAL B 48 2.761 -2.855 -2.040 1.00 1.00

ATOM 1401 N LEU B 49 4.323 1.384 -1.539 1.00 1.00

ATOM 1402 CA LEU B 49 4.609 2.678 -0.922 1.00 1.00

ATOM 1403 C LEU B 49 4.145 3.843 -1.800 1.00 1.00

ATOM 1404 O LEU B 49 3.499 4.773 -1.311 1.00 1.00

ATOM 1405 CB LEU B 49 6.102 2.807 -0.602 1.00 1.00

ATOM 1406 CG LEU B 49 6.637 4.192 -0.216 1.00 1.00

ATOM 1407 CD1 LEU B 49 6.397 4.486 1.258 1.00 1.00

ATOM 1408 CD2 LEU B 49 8.119 4.303 -0.547 1.00 1.00

ATOM 1409 N LEU B 50 4.464 3.772 -3.089 1.00 1.00

ATOM 1410 CA LEU B 50 4.129 4.833 -4.040 1.00 1.00

ATOM 1411 C LEU B 50 2.623 5.002 -4.243 1.00 1.00

ATOM 1412 O LEU B 50 2.125 6.131 -4.276 1.00 1.00

ATOM 1413 CB LEU B 50 4.828 4.602 -5.384 1.00 1.00

ATOM 1414 CG LEU B 50 6.218 5.223 -5.561 1.00 1.00

ATOM 1415 CD1 LEU B 50 7.133 4.806 -4.420 1.00 1.00

ATOM 1416 CD2 LEU B 50 6.836 4.859 -6.911 1.00 1.00

ATOM 1417 N LEU B 51 1.905 3.888 -4.371 1.00 1.00

ATOM 1418 CA LEU B 51 0.452 3.922 -4.544 1.00 1.00

ATOM 1419 C LEU B 51 -0.268 4.428 -3.289 1.00 1.00

ATOM 1420 O LEU B 51 -1.216 5.210 -3.391 1.00 1.00

ATOM 1421 CB LEU B 51 -0.094 2.550 -4.973 1.00 1.00

ATOM 1422 CG LEU B 51 -1.598 2.415 -5.257 1.00 1.00

ATOM 1423 CD1 LEU B 51 -2.092 3.452 -6.265 1.00 1.00

ATOM 1424 CD2 LEU B 51 -1.940 1.014 -5.733 1.00 1.00

ATOM 1425 N SER B 52 0.185 3.980 -2.118 1.00 1.00

ATOM 1426 CA SER B 52 -0.399 4.404 -0.846 1.00 1.00

ATOM 1427 C SER B 52 -0.213 5.903 -0.598 1.00 1.00

ATOM 1428 O SER B 52 -1.102 6.562 -0.058 1.00 1.00

ATOM 1429 CB SER B 52 0.171 3.591 0.322 1.00 1.00

ATOM 1430 OG SER B 52 1.544 3.874 0.534 1.00 1.00

ATOM 1431 N ARG B 53 0.944 6.426 -1.004 1.00 1.00

ATOM 1432 CA ARG B 53 1.239 7.855 -0.910 1.00 1.00

ATOM 1433 C ARG B 53 0.391 8.673 -1.885 1.00 1.00

ATOM 1434 O ARG B 53 -0.021 9.791 -1.569 1.00 1.00

ATOM 1435 CB ARG B 53 2.728 8.118 -1.153 1.00 1.00

ATOM 1436 CG ARG B 53 3.594 7.892 0.075 1.00 1.00

ATOM 1437 CD ARG B 53 5.081 8.099 -0.158 1.00 1.00

ATOM 1438 NE ARG B 53 5.860 7.819 1.048 1.00 1.00

ATOM 1439 CZ ARG B 53 7.183 7.709 1.085 1.00 1.00

ATOM 1440 NH1 ARG B 53 7.905 7.852 -0.021 1.00 1.00

ATOM 1441 NH2 ARG B 53 7.790 7.453 2.234 1.00 1.00

ATOM 1442 N TYR B 54 0.134 8.106 -3.064 1.00 1.00

ATOM 1443 CA TYR B 54 -0.708 8.740 -4.077 1.00 1.00

ATOM 1444 C TYR B 54 -2.145 8.912 -3.579 1.00 1.00

ATOM 1445 O TYR B 54 -2.769 9.949 -3.813 1.00 1.00

ATOM 1446 CB TYR B 54 -0.685 7.934 -5.384 1.00 1.00

ATOM 1447 CG TYR B 54 -1.617 8.460 -6.458 1.00 1.00

ATOM 1448 CD1 TYR B 54 -1.236 9.514 -7.287 1.00 1.00

ATOM 1449 CD2 TYR B 54 -2.885 7.899 -6.644 1.00 1.00

ATOM 1450 CE1 TYR B 54 -2.091 10.000 -8.273 1.00 1.00

ATOM 1451 CE2 TYR B 54 -3.747 8.379 -7.626 1.00 1.00

ATOM 1452 CZ TYR B 54 -3.342 9.427 -8.436 1.00 1.00

ATOM 1453 OH TYR B 54 -4.192 9.905 -9.410 1.00 1.00

ATOM 1454 N LEU B 55 -2.657 7.892 -2.893 1.00 1.00

ATOM 1455 CA LEU B 55 -4.017 7.916 -2.357 1.00 1.00

ATOM 1456 C LEU B 55 -4.140 8.819 -1.129 1.00 1.00

ATOM 1457 O LEU B 55 -5.219 9.342 -0.842 1.00 1.00

ATOM 1458 CB LEU B 55 -4.496 6.498 -2.026 1.00 1.00

ATOM 1459 CG LEU B 55 -4.611 5.494 -3.178 1.00 1.00

ATOM 1460 CD1 LEU B 55 -4.707 4.073 -2.643 1.00 1.00

ATOM 1461 CD2 LEU B 55 -5.796 5.809 -4.083 1.00 1.00

ATOM 1462 N LEU B 56 -3.031 9.001 -0.414 1.00 1.00

ATOM 1463 CA LEU B 56 -2.999 9.845 0.779 1.00 1.00

ATOM 1464 C LEU B 56 -2.563 11.270 0.442 1.00 1.00

ATOM 1465 O LEU B 56 -3.087 11.895 -0.482 1.00 1.00

ATOM 1466 CB LEU B 56 -2.069 9.245 1.836 1.00 1.00

ATOM 1467 CG LEU B 56 -2.338 9.654 3.285 1.00 1.00

ATOM 1468 CD1 LEU B 56 -3.647 9.058 3.778 1.00 1.00

ATOM 1469 CD2 LEU B 56 -1.222 9.167 4.196 1.00 1.00

ATOM 1470 OXT LEU B 56 -1.633 11.771 1.119 1.00 1.00

ATOM 1471 CA CA C 160 -9.178 0.648 -10.001 1.00 1.00

ATOM 1472 CA CA D 161 7.828 -2.061 -9.567 1.00 1.00

TER

ENDMDL

MODEL 3

ATOM 0 N GLY A 5 -26.382 0.617 3.011 1.00 1.00

ATOM 1 CA GLY A 5 -24.952 0.189 3.012 1.00 1.00

ATOM 2 C GLY A 5 -24.188 0.716 4.211 1.00 1.00

ATOM 3 O GLY A 5 -24.665 0.631 5.346 1.00 1.00

ATOM 4 N VAL A 6 -23.001 1.261 3.955 1.00 1.00

ATOM 5 CA VAL A 6 -22.150 1.819 5.004 1.00 1.00

ATOM 6 C VAL A 6 -22.351 3.331 5.108 1.00 1.00

ATOM 7 O VAL A 6 -22.383 4.033 4.095 1.00 1.00

ATOM 8 CB VAL A 6 -20.651 1.494 4.765 1.00 1.00

ATOM 9 CG1 VAL A 6 -19.804 1.881 5.975 1.00 1.00

ATOM 10 CG2 VAL A 6 -20.459 0.014 4.442 1.00 1.00

ATOM 11 N VAL A 7 -22.501 3.818 6.339 1.00 1.00

ATOM 12 CA VAL A 7 -22.658 5.247 6.598 1.00 1.00

ATOM 13 C VAL A 7 -21.448 5.781 7.362 1.00 1.00

ATOM 14 O VAL A 7 -21.121 5.296 8.449 1.00 1.00

ATOM 15 CB VAL A 7 -23.965 5.555 7.384 1.00 1.00

ATOM 16 CG1 VAL A 7 -24.147 7.056 7.568 1.00 1.00

ATOM 17 CG2 VAL A 7 -25.181 4.957 6.678 1.00 1.00

ATOM 18 N VAL A 8 -20.779 6.771 6.778 1.00 1.00

ATOM 19 CA VAL A 8 -19.646 7.428 7.427 1.00 1.00

ATOM 20 C VAL A 8 -20.111 8.746 8.048 1.00 1.00

ATOM 21 O VAL A 8 -20.573 9.648 7.346 1.00 1.00

ATOM 22 CB VAL A 8 -18.471 7.672 6.448 1.00 1.00

ATOM 23 CG1 VAL A 8 -17.296 8.328 7.164 1.00 1.00

ATOM 24 CG2 VAL A 8 -18.031 6.365 5.795 1.00 1.00

ATOM 25 N GLU A 9 -19.990 8.840 9.370 1.00 1.00

ATOM 26 CA GLU A 9 -20.486 9.995 10.110 1.00 1.00

ATOM 27 C GLU A 9 -19.352 10.862 10.640 1.00 1.00

ATOM 28 O GLU A 9 -18.575 10.427 11.494 1.00 1.00

ATOM 29 CB GLU A 9 -21.376 9.544 11.273 1.00 1.00

ATOM 30 CG GLU A 9 -22.802 9.193 10.880 1.00 1.00

ATOM 31 CD GLU A 9 -23.518 8.343 11.918 1.00 1.00

ATOM 32 OE1 GLU A 9 -22.960 8.120 13.016 1.00 1.00

ATOM 33 OE2 GLU A 9 -24.648 7.891 11.632 1.00 1.00

ATOM 34 N ILE A 10 -19.257 12.084 10.125 1.00 1.00

ATOM 35 CA ILE A 10 -18.360 13.082 10.695 1.00 1.00

ATOM 36 C ILE A 10 -19.049 13.666 11.923 1.00 1.00

ATOM 37 O ILE A 10 -20.124 14.261 11.818 1.00 1.00

ATOM 38 CB ILE A 10 -18.010 14.191 9.670 1.00 1.00

ATOM 39 CG1 ILE A 10 -17.315 13.592 8.442 1.00 1.00

ATOM 40 CG2 ILE A 10 -17.127 15.263 10.315 1.00 1.00

ATOM 41 CD1 ILE A 10 -17.570 14.348 7.147 1.00 1.00

ATOM 42 N GLY A 11 -18.427 13.472 13.085 1.00 1.00

ATOM 43 CA GLY A 11 -18.997 13.888 14.355 1.00 1.00

ATOM 44 C GLY A 11 -19.236 15.381 14.481 1.00 1.00

ATOM 45 O GLY A 11 -18.627 16.184 13.768 1.00 1.00

ATOM 46 N LYS A 12 -20.137 15.742 15.390 1.00 1.00

ATOM 47 CA LYS A 12 -20.441 17.138 15.681 1.00 1.00

ATOM 48 C LYS A 12 -20.135 17.435 17.146 1.00 1.00

ATOM 49 O LYS A 12 -20.752 16.861 18.045 1.00 1.00

ATOM 50 CB LYS A 12 -21.905 17.452 15.363 1.00 1.00

ATOM 51 CG LYS A 12 -22.193 17.629 13.884 1.00 1.00

ATOM 52 CD LYS A 12 -23.679 17.769 13.617 1.00 1.00

ATOM 53 CE LYS A 12 -24.036 17.288 12.221 1.00 1.00

ATOM 54 NZ LYS A 12 -25.219 18.006 11.666 1.00 1.00

ATOM 55 N VAL A 13 -19.169 18.321 17.369 1.00 1.00

ATOM 56 CA VAL A 13 -18.741 18.690 18.719 1.00 1.00

ATOM 57 C VAL A 13 -18.770 20.206 18.923 1.00 1.00

ATOM 58 O VAL A 13 -18.858 20.970 17.958 1.00 1.00

ATOM 59 CB VAL A 13 -17.322 18.141 19.059 1.00 1.00

ATOM 60 CG1 VAL A 13 -17.336 16.620 19.168 1.00 1.00

ATOM 61 CG2 VAL A 13 -16.283 18.610 18.037 1.00 1.00

ATOM 62 N THR A 14 -18.711 20.632 20.183 1.00 1.00

ATOM 63 CA THR A 14 -18.613 22.052 20.522 1.00 1.00

ATOM 64 C THR A 14 -17.377 22.309 21.379 1.00 1.00

ATOM 65 O THR A 14 -16.962 21.448 22.158 1.00 1.00

ATOM 66 CB THR A 14 -19.884 22.553 21.251 1.00 1.00

ATOM 67 OG1 THR A 14 -20.230 21.649 22.307 1.00 1.00

ATOM 68 CG2 THR A 14 -21.099 22.514 20.329 1.00 1.00

ATOM 69 N GLY A 15 -16.796 23.496 21.232 1.00 1.00

ATOM 70 CA GLY A 15 -15.604 23.865 21.974 1.00 1.00

ATOM 71 C GLY A 15 -15.295 25.348 21.925 1.00 1.00

ATOM 72 O GLY A 15 -15.709 26.051 20.999 1.00 1.00

ATOM 73 N SER A 16 -14.566 25.819 22.934 1.00 1.00

ATOM 74 CA SER A 16 -14.172 27.220 23.026 1.00 1.00

ATOM 75 C SER A 16 -12.972 27.530 22.134 1.00 1.00

ATOM 76 O SER A 16 -12.187 26.637 21.798 1.00 1.00

ATOM 77 CB SER A 16 -13.848 27.589 24.477 1.00 1.00

ATOM 78 OG SER A 16 -14.997 27.485 25.301 1.00 1.00

ATOM 79 N VAL A 17 -12.843 28.798 21.752 1.00 1.00

ATOM 80 CA VAL A 17 -11.699 29.275 20.979 1.00 1.00

ATOM 81 C VAL A 17 -10.420 29.126 21.807 1.00 1.00

ATOM 82 O VAL A 17 -10.384 29.506 22.980 1.00 1.00

ATOM 83 CB VAL A 17 -11.887 30.751 20.529 1.00 1.00

ATOM 84 CG1 VAL A 17 -10.743 31.207 19.623 1.00 1.00

ATOM 85 CG2 VAL A 17 -13.225 30.939 19.822 1.00 1.00

ATOM 86 N GLY A 18 -9.387 28.551 21.195 1.00 1.00

ATOM 87 CA GLY A 18 -8.115 28.337 21.861 1.00 1.00

ATOM 88 C GLY A 18 -7.946 26.944 22.445 1.00 1.00

ATOM 89 O GLY A 18 -6.852 26.589 22.889 1.00 1.00

ATOM 90 N THR A 19 -9.024 26.163 22.457 1.00 1.00

ATOM 91 CA THR A 19 -8.972 24.788 22.955 1.00 1.00

ATOM 92 C THR A 19 -8.813 23.791 21.809 1.00 1.00

ATOM 93 O THR A 19 -9.215 24.064 20.677 1.00 1.00

ATOM 94 CB THR A 19 -10.228 24.435 23.796 1.00 1.00

ATOM 95 OG1 THR A 19 -11.386 24.399 22.951 1.00 1.00

ATOM 96 CG2 THR A 19 -10.549 25.534 24.807 1.00 1.00

ATOM 97 N THR A 20 -8.199 22.643 22.118 1.00 1.00

ATOM 98 CA THR A 20 -8.110 21.561 21.126 1.00 1.00

ATOM 99 C THR A 20 -9.226 20.537 21.391 1.00 1.00

ATOM 100 O THR A 20 -9.376 20.023 22.507 1.00 1.00

ATOM 101 CB THR A 20 -6.735 20.882 21.230 1.00 1.00

ATOM 102 OG1 THR A 20 -5.700 21.858 21.182 1.00 1.00

ATOM 103 CG2 THR A 20 -6.469 19.888 20.102 1.00 1.00

ATOM 104 N VAL A 21 -9.994 20.261 20.347 1.00 1.00

ATOM 105 CA VAL A 21 -11.122 19.309 20.432 1.00 1.00

ATOM 106 C VAL A 21 -10.900 18.113 19.505 1.00 1.00

ATOM 107 O VAL A 21 -10.069 18.151 18.588 1.00 1.00

ATOM 108 CB VAL A 21 -12.434 19.994 20.031 1.00 1.00

ATOM 109 CG1 VAL A 21 -12.853 21.090 21.014 1.00 1.00

ATOM 110 CG2 VAL A 21 -12.370 20.667 18.655 1.00 1.00

ATOM 111 N GLU A 22 -11.660 17.073 19.780 1.00 1.00

ATOM 112 CA GLU A 22 -11.597 15.833 19.008 1.00 1.00

ATOM 113 C GLU A 22 -12.903 15.602 18.262 1.00 1.00

ATOM 114 O GLU A 22 -13.985 15.572 18.861 1.00 1.00

ATOM 115 CB GLU A 22 -11.336 14.654 19.937 1.00 1.00

ATOM 116 CG GLU A 22 -9.968 14.009 19.761 1.00 1.00

ATOM 117 CD GLU A 22 -9.575 13.137 20.938 1.00 1.00

ATOM 118 OE1 GLU A 22 -10.196 12.069 21.121 1.00 1.00

ATOM 119 OE2 GLU A 22 -8.644 13.522 21.678 1.00 1.00

ATOM 120 N ILE A 23 -12.767 15.448 16.961 1.00 1.00

ATOM 121 CA ILE A 23 -13.914 15.193 16.083 1.00 1.00

ATOM 122 C ILE A 23 -13.847 13.758 15.564 1.00 1.00

ATOM 123 O ILE A 23 -12.950 13.401 14.789 1.00 1.00

ATOM 124 CB ILE A 23 -13.918 16.147 14.886 1.00 1.00

ATOM 125 CG1 ILE A 23 -13.774 17.617 15.290 1.00 1.00

ATOM 126 CG2 ILE A 23 -15.215 16.057 14.073 1.00 1.00

ATOM 127 CD1 ILE A 23 -12.458 18.242 14.820 1.00 1.00

ATOM 128 N PRO A 24 -14.713 12.914 16.117 1.00 1.00

ATOM 129 CA PRO A 24 -14.730 11.494 15.747 1.00 1.00

ATOM 130 C PRO A 24 -15.326 11.272 14.360 1.00 1.00

ATOM 131 O PRO A 24 -16.227 12.002 13.943 1.00 1.00

ATOM 132 CB PRO A 24 -15.617 10.858 16.827 1.00 1.00

ATOM 133 CG PRO A 24 -16.506 11.961 17.304 1.00 1.00

ATOM 134 CD PRO A 24 -15.767 13.249 17.095 1.00 1.00

ATOM 135 N VAL A 25 -14.793 10.279 13.652 1.00 1.00

ATOM 136 CA VAL A 25 -15.353 9.833 12.381 1.00 1.00

ATOM 137 C VAL A 25 -15.895 8.422 12.596 1.00 1.00

ATOM 138 O VAL A 25 -15.135 7.497 12.885 1.00 1.00

ATOM 139 CB VAL A 25 -14.297 9.845 11.244 1.00 1.00

ATOM 140 CG1 VAL A 25 -14.908 9.376 9.929 1.00 1.00

ATOM 141 CG2 VAL A 25 -13.693 11.235 11.080 1.00 1.00

ATOM 142 N TYR A 26 -17.211 8.272 12.466 1.00 1.00

ATOM 143 CA TYR A 26 -17.880 7.006 12.752 1.00 1.00

ATOM 144 C TYR A 26 -18.131 6.175 11.497 1.00 1.00

ATOM 145 O TYR A 26 -18.486 6.711 10.447 1.00 1.00

ATOM 146 CB TYR A 26 -19.218 7.250 13.460 1.00 1.00

ATOM 147 CG TYR A 26 -19.117 7.942 14.802 1.00 1.00

ATOM 148 CD1 TYR A 26 -19.445 9.290 14.937 1.00 1.00

ATOM 149 CD2 TYR A 26 -18.709 7.245 15.942 1.00 1.00

ATOM 150 CE1 TYR A 26 -19.363 9.934 16.170 1.00 1.00

ATOM 151 CE2 TYR A 26 -18.623 7.880 17.180 1.00 1.00

ATOM 152 CZ TYR A 26 -18.952 9.222 17.286 1.00 1.00

ATOM 153 OH TYR A 26 -18.869 9.854 18.508 1.00 1.00

ATOM 154 N PHE A 27 -17.942 4.863 11.623 1.00 1.00

ATOM 155 CA PHE A 27 -18.354 3.914 10.594 1.00 1.00

ATOM 156 C PHE A 27 -19.603 3.184 11.078 1.00 1.00

ATOM 157 O PHE A 27 -19.561 2.457 12.072 1.00 1.00

ATOM 158 CB PHE A 27 -17.235 2.909 10.293 1.00 1.00

ATOM 159 CG PHE A 27 -16.260 3.363 9.231 1.00 1.00

ATOM 160 CD1 PHE A 27 -16.177 4.701 8.847 1.00 1.00

ATOM 161 CD2 PHE A 27 -15.412 2.441 8.624 1.00 1.00

ATOM 162 CE1 PHE A 27 -15.266 5.111 7.873 1.00 1.00

ATOM 163 CE2 PHE A 27 -14.500 2.841 7.647 1.00 1.00

ATOM 164 CZ PHE A 27 -14.428 4.180 7.273 1.00 1.00

ATOM 165 N ARG A 28 -20.715 3.396 10.380 1.00 1.00

ATOM 166 CA ARG A 28 -21.978 2.745 10.717 1.00 1.00

ATOM 167 C ARG A 28 -22.413 1.800 9.602 1.00 1.00

ATOM 168 O ARG A 28 -22.226 2.096 8.421 1.00 1.00

ATOM 169 CB ARG A 28 -23.077 3.777 10.997 1.00 1.00

ATOM 170 CG ARG A 28 -22.707 4.841 12.027 1.00 1.00

ATOM 171 CD ARG A 28 -23.058 4.484 13.463 1.00 1.00

ATOM 172 NE ARG A 28 -22.739 5.572 14.389 1.00 1.00

ATOM 173 CZ ARG A 28 -22.233 5.401 15.608 1.00 1.00

ATOM 174 NH1 ARG A 28 -21.980 6.458 16.372 1.00 1.00

ATOM 175 NH2 ARG A 28 -21.976 4.184 16.070 1.00 1.00

ATOM 176 N GLY A 29 -22.984 0.663 9.992 1.00 1.00

ATOM 177 CA GLY A 29 -23.461 -0.331 9.045 1.00 1.00

ATOM 178 C GLY A 29 -22.346 -1.091 8.348 1.00 1.00

ATOM 179 O GLY A 29 -22.407 -1.318 7.137 1.00 1.00

ATOM 180 N VAL A 30 -21.329 -1.479 9.118 1.00 1.00

ATOM 181 CA VAL A 30 -20.206 -2.268 8.612 1.00 1.00

ATOM 182 C VAL A 30 -20.703 -3.666 8.229 1.00 1.00

ATOM 183 O VAL A 30 -21.301 -4.357 9.059 1.00 1.00

ATOM 184 CB VAL A 30 -19.054 -2.354 9.654 1.00 1.00

ATOM 185 CG1 VAL A 30 -17.937 -3.281 9.179 1.00 1.00

ATOM 186 CG2 VAL A 30 -18.493 -0.968 9.959 1.00 1.00

ATOM 187 N PRO A 31 -20.467 -4.075 6.978 1.00 1.00

ATOM 188 CA PRO A 31 -20.999 -5.343 6.456 1.00 1.00

ATOM 189 C PRO A 31 -20.384 -6.584 7.104 1.00 1.00

ATOM 190 O PRO A 31 -19.391 -6.480 7.830 1.00 1.00

ATOM 191 CB PRO A 31 -20.644 -5.286 4.965 1.00 1.00

ATOM 192 CG PRO A 31 -19.470 -4.378 4.890 1.00 1.00

ATOM 193 CD PRO A 31 -19.678 -3.353 5.964 1.00 1.00

ATOM 194 N SER A 32 -20.949 -7.741 6.756 1.00 1.00

ATOM 195 CA SER A 32 -20.514 -8.969 7.421 1.00 1.00

ATOM 196 C SER A 32 -19.112 -9.385 6.951 1.00 1.00

ATOM 197 O SER A 32 -18.348 -9.998 7.708 1.00 1.00

ATOM 198 CB SER A 32 -21.490 -10.091 7.126 1.00 1.00

ATOM 199 OG SER A 32 -21.773 -10.187 5.735 1.00 1.00

ATOM 200 N LYS A 33 -18.793 -9.042 5.707 1.00 1.00

ATOM 201 CA LYS A 33 -17.474 -9.376 5.118 1.00 1.00

ATOM 202 C LYS A 33 -16.394 -8.386 5.651 1.00 1.00

ATOM 203 O LYS A 33 -15.187 -8.639 5.535 1.00 1.00

ATOM 204 CB LYS A 33 -17.536 -9.322 3.564 1.00 1.00

ATOM 205 CG LYS A 33 -18.515 -10.307 2.974 1.00 1.00

ATOM 206 CD LYS A 33 -18.648 -10.089 1.476 1.00 1.00

ATOM 207 CE LYS A 33 -19.999 -9.488 1.122 1.00 1.00

ATOM 208 NZ LYS A 33 -20.099 -9.162 -0.327 1.00 1.00

ATOM 209 N GLY A 34 -16.862 -7.274 6.229 1.00 1.00

ATOM 210 CA GLY A 34 -15.983 -6.225 6.830 1.00 1.00

ATOM 211 C GLY A 34 -15.553 -5.173 5.798 1.00 1.00

ATOM 212 O GLY A 34 -16.113 -5.079 4.704 1.00 1.00

ATOM 213 N ILE A 35 -14.557 -4.394 6.194 1.00 1.00

ATOM 214 CA ILE A 35 -13.980 -3.345 5.335 1.00 1.00

ATOM 215 C ILE A 35 -12.454 -3.470 5.339 1.00 1.00

ATOM 216 O ILE A 35 -11.798 -3.259 6.371 1.00 1.00

ATOM 217 CB ILE A 35 -14.357 -1.939 5.823 1.00 1.00

ATOM 218 CG1 ILE A 35 -15.885 -1.687 5.838 1.00 1.00

ATOM 219 CG2 ILE A 35 -13.739 -0.834 4.950 1.00 1.00

ATOM 220 CD1 ILE A 35 -16.527 -1.663 4.443 1.00 1.00

ATOM 221 N ALA A 36 -11.917 -3.941 4.220 1.00 1.00

ATOM 222 CA ALA A 36 -10.484 -4.206 4.080 1.00 1.00

ATOM 223 C ALA A 36 -9.691 -2.957 3.695 1.00 1.00

ATOM 224 O ALA A 36 -8.515 -2.829 4.043 1.00 1.00

ATOM 225 CB ALA A 36 -10.249 -5.321 3.066 1.00 1.00

ATOM 226 N ASN A 37 -10.339 -2.048 2.969 1.00 1.00

ATOM 227 CA ASN A 37 -9.706 -0.809 2.520 1.00 1.00

ATOM 228 C ASN A 37 -10.710 0.308 2.257 1.00 1.00

ATOM 229 O ASN A 37 -11.846 0.052 1.846 1.00 1.00

ATOM 230 CB ASN A 37 -8.861 -1.053 1.261 1.00 1.00

ATOM 231 CG ASN A 37 -9.705 -1.403 0.047 1.00 1.00

ATOM 232 OD1 ASN A 37 -9.994 -2.574 -0.207 1.00 1.00

ATOM 233 ND2 ASN A 37 -10.100 -0.389 -0.709 1.00 1.00

ATOM 234 N CYS A 38 -10.277 1.544 2.493 1.00 1.00

ATOM 235 CA CYS A 38 -11.066 2.728 2.164 1.00 1.00

ATOM 236 C CYS A 38 -10.207 3.985 2.094 1.00 1.00

ATOM 237 O CYS A 38 -9.181 4.093 2.773 1.00 1.00

ATOM 238 CB CYS A 38 -12.215 2.926 3.160 1.00 1.00

ATOM 239 SG CYS A 38 -11.700 3.240 4.856 1.00 1.00

ATOM 240 N ASP A 39 -10.624 4.923 1.250 1.00 1.00

ATOM 241 CA ASP A 39 -10.000 6.238 1.174 1.00 1.00

ATOM 242 C ASP A 39 -11.036 7.297 0.825 1.00 1.00

ATOM 243 O ASP A 39 -11.946 7.052 0.029 1.00 1.00

ATOM 244 CB ASP A 39 -8.826 6.252 0.182 1.00 1.00

ATOM 245 CG ASP A 39 -9.275 6.340 -1.264 1.00 1.00

ATOM 246 OD1 ASP A 39 -9.508 5.280 -1.880 1.00 1.00

ATOM 247 OD2 ASP A 39 -9.422 7.424 -1.866 1.00 1.00

ATOM 248 N PHE A 40 -10.903 8.462 1.453 1.00 1.00

ATOM 249 CA PHE A 40 -11.802 9.589 1.214 1.00 1.00

ATOM 250 C PHE A 40 -11.191 10.918 1.651 1.00 1.00

ATOM 251 O PHE A 40 -10.274 10.954 2.476 1.00 1.00

ATOM 252 CB PHE A 40 -13.179 9.380 1.880 1.00 1.00

ATOM 253 CG PHE A 40 -13.119 8.748 3.247 1.00 1.00

ATOM 254 CD1 PHE A 40 -12.876 9.524 4.378 1.00 1.00

ATOM 255 CD2 PHE A 40 -13.335 7.383 3.406 1.00 1.00

ATOM 256 CE1 PHE A 40 -12.830 8.944 5.645 1.00 1.00

ATOM 257 CE2 PHE A 40 -13.291 6.794 4.667 1.00 1.00

ATOM 258 CZ PHE A 40 -13.039 7.578 5.789 1.00 1.00

ATOM 259 N VAL A 41 -11.709 12.000 1.077 1.00 1.00

ATOM 260 CA VAL A 41 -11.228 13.352 1.342 1.00 1.00

ATOM 261 C VAL A 41 -12.326 14.172 2.019 1.00 1.00

ATOM 262 O VAL A 41 -13.487 14.127 1.603 1.00 1.00

ATOM 263 CB VAL A 41 -10.783 14.059 0.029 1.00 1.00

ATOM 264 CG1 VAL A 41 -10.251 15.464 0.306 1.00 1.00

ATOM 265 CG2 VAL A 41 -9.737 13.232 -0.713 1.00 1.00

ATOM 266 N PHE A 42 -11.958 14.902 3.071 1.00 1.00

ATOM 267 CA PHE A 42 -12.865 15.842 3.726 1.00 1.00

ATOM 268 C PHE A 42 -12.389 17.281 3.524 1.00 1.00

ATOM 269 O PHE A 42 -11.186 17.543 3.456 1.00 1.00

ATOM 270 CB PHE A 42 -12.987 15.551 5.231 1.00 1.00

ATOM 271 CG PHE A 42 -13.671 14.243 5.565 1.00 1.00

ATOM 272 CD1 PHE A 42 -13.521 13.685 6.831 1.00 1.00

ATOM 273 CD2 PHE A 42 -14.472 13.579 4.636 1.00 1.00

ATOM 274 CE1 PHE A 42 -14.141 12.482 7.164 1.00 1.00

ATOM 275 CE2 PHE A 42 -15.098 12.376 4.960 1.00 1.00

ATOM 276 CZ PHE A 42 -14.932 11.829 6.229 1.00 1.00

ATOM 277 N ARG A 43 -13.341 18.206 3.434 1.00 1.00

ATOM 278 CA ARG A 43 -13.047 19.631 3.299 1.00 1.00

ATOM 279 C ARG A 43 -13.218 20.345 4.642 1.00 1.00

ATOM 280 O ARG A 43 -14.064 19.959 5.452 1.00 1.00

ATOM 281 CB ARG A 43 -13.968 20.257 2.249 1.00 1.00

ATOM 282 CG ARG A 43 -13.427 21.522 1.602 1.00 1.00

ATOM 283 CD ARG A 43 -13.790 21.673 0.133 1.00 1.00

ATOM 284 NE ARG A 43 -12.635 21.456 -0.735 1.00 1.00

ATOM 285 CZ ARG A 43 -12.701 21.194 -2.036 1.00 1.00

ATOM 286 NH1 ARG A 43 -13.878 21.115 -2.651 1.00 1.00

ATOM 287 NH2 ARG A 43 -11.586 21.011 -2.728 1.00 1.00

ATOM 288 N TYR A 44 -12.407 21.377 4.869 1.00 1.00

ATOM 289 CA TYR A 44 -12.500 22.199 6.080 1.00 1.00

ATOM 290 C TYR A 44 -11.967 23.618 5.866 1.00 1.00

ATOM 291 O TYR A 44 -11.327 23.904 4.852 1.00 1.00

ATOM 292 CB TYR A 44 -11.787 21.519 7.261 1.00 1.00

ATOM 293 CG TYR A 44 -10.279 21.686 7.294 1.00 1.00

ATOM 294 CD1 TYR A 44 -9.459 20.984 6.409 1.00 1.00

ATOM 295 CD2 TYR A 44 -9.673 22.535 8.221 1.00 1.00

ATOM 296 CE1 TYR A 44 -8.073 21.129 6.442 1.00 1.00

ATOM 297 CE2 TYR A 44 -8.290 22.687 8.264 1.00 1.00

ATOM 298 CZ TYR A 44 -7.497 21.981 7.371 1.00 1.00

ATOM 299 OH TYR A 44 -6.130 22.126 7.407 1.00 1.00

ATOM 300 N ASP A 45 -12.243 24.498 6.828 1.00 1.00

ATOM 301 CA ASP A 45 -11.743 25.871 6.809 1.00 1.00

ATOM 302 C ASP A 45 -10.557 26.029 7.763 1.00 1.00

ATOM 303 O ASP A 45 -10.722 25.947 8.984 1.00 1.00

ATOM 304 CB ASP A 45 -12.857 26.859 7.174 1.00 1.00

ATOM 305 CG ASP A 45 -12.555 28.285 6.731 1.00 1.00

ATOM 306 OD1 ASP A 45 -11.368 28.671 6.659 1.00 1.00

ATOM 307 OD2 ASP A 45 -13.456 29.101 6.437 1.00 1.00

ATOM 308 N PRO A 46 -9.367 26.260 7.204 1.00 1.00

ATOM 309 CA PRO A 46 -8.139 26.432 7.996 1.00 1.00

ATOM 310 C PRO A 46 -8.123 27.708 8.841 1.00 1.00

ATOM 311 O PRO A 46 -7.372 27.772 9.816 1.00 1.00

ATOM 312 CB PRO A 46 -7.040 26.496 6.928 1.00 1.00

ATOM 313 CG PRO A 46 -7.668 25.946 5.701 1.00 1.00

ATOM 314 CD PRO A 46 -9.100 26.362 5.759 1.00 1.00

ATOM 315 N ASN A 47 -8.927 28.702 8.465 1.00 1.00

ATOM 316 CA ASN A 47 -9.056 29.937 9.241 1.00 1.00

ATOM 317 C ASN A 47 -9.897 29.745 10.502 1.00 1.00

ATOM 318 O ASN A 47 -9.771 30.506 11.464 1.00 1.00

ATOM 319 CB ASN A 47 -9.648 31.064 8.386 1.00 1.00

ATOM 320 CG ASN A 47 -8.770 31.430 7.202 1.00 1.00

ATOM 321 OD1 ASN A 47 -7.541 31.464 7.303 1.00 1.00

ATOM 322 ND2 ASN A 47 -9.400 31.712 6.067 1.00 1.00

ATOM 323 N VAL A 48 -10.757 28.728 10.484 1.00 1.00

ATOM 324 CA VAL A 48 -11.626 28.414 11.614 1.00 1.00

ATOM 325 C VAL A 48 -11.025 27.306 12.481 1.00 1.00

ATOM 326 O VAL A 48 -11.031 27.401 13.710 1.00 1.00

ATOM 327 CB VAL A 48 -13.050 28.008 11.147 1.00 1.00

ATOM 328 CG1 VAL A 48 -13.966 27.782 12.341 1.00 1.00

ATOM 329 CG2 VAL A 48 -13.641 29.062 10.214 1.00 1.00

ATOM 330 N LEU A 49 -10.504 26.263 11.836 1.00 1.00

ATOM 331 CA LEU A 49 -9.929 25.122 12.545 1.00 1.00

ATOM 332 C LEU A 49 -8.503 24.828 12.102 1.00 1.00

ATOM 333 O LEU A 49 -8.210 24.768 10.907 1.00 1.00

ATOM 334 CB LEU A 49 -10.790 23.867 12.357 1.00 1.00

ATOM 335 CG LEU A 49 -12.254 23.862 12.806 1.00 1.00

ATOM 336 CD1 LEU A 49 -12.935 22.585 12.333 1.00 1.00

ATOM 337 CD2 LEU A 49 -12.386 24.016 14.316 1.00 1.00

ATOM 338 N GLU A 50 -7.618 24.650 13.079 1.00 1.00

ATOM 339 CA GLU A 50 -6.256 24.201 12.811 1.00 1.00

ATOM 340 C GLU A 50 -6.145 22.724 13.176 1.00 1.00

ATOM 341 O GLU A 50 -6.047 22.372 14.353 1.00 1.00

ATOM 342 CB GLU A 50 -5.237 25.040 13.591 1.00 1.00

ATOM 343 CG GLU A 50 -3.784 24.689 13.296 1.00 1.00

ATOM 344 CD GLU A 50 -2.800 25.391 14.213 1.00 1.00

ATOM 345 OE1 GLU A 50 -3.032 25.426 15.441 1.00 1.00

ATOM 346 OE2 GLU A 50 -1.782 25.905 13.702 1.00 1.00

ATOM 347 N ILE A 51 -6.181 21.864 12.160 1.00 1.00

ATOM 348 CA ILE A 51 -6.078 20.423 12.374 1.00 1.00

ATOM 349 C ILE A 51 -4.613 20.035 12.560 1.00 1.00

ATOM 350 O ILE A 51 -3.797 20.174 11.645 1.00 1.00

ATOM 351 CB ILE A 51 -6.746 19.630 11.219 1.00 1.00

ATOM 352 CG1 ILE A 51 -8.253 19.920 11.182 1.00 1.00

ATOM 353 CG2 ILE A 51 -6.485 18.127 11.371 1.00 1.00

ATOM 354 CD1 ILE A 51 -8.964 19.434 9.934 1.00 1.00

ATOM 355 N ILE A 52 -4.296 19.556 13.759 1.00 1.00

ATOM 356 CA ILE A 52 -2.916 19.267 14.149 1.00 1.00

ATOM 357 C ILE A 52 -2.565 17.778 14.086 1.00 1.00

ATOM 358 O ILE A 52 -1.395 17.406 14.206 1.00 1.00

ATOM 359 CB ILE A 52 -2.605 19.849 15.556 1.00 1.00

ATOM 360 CG1 ILE A 52 -3.650 19.402 16.585 1.00 1.00

ATOM 361 CG2 ILE A 52 -2.514 21.374 15.495 1.00 1.00

ATOM 362 CD1 ILE A 52 -3.062 19.002 17.928 1.00 1.00

ATOM 363 N GLY A 53 -3.580 16.935 13.897 1.00 1.00

ATOM 364 CA GLY A 53 -3.370 15.502 13.798 1.00 1.00

ATOM 365 C GLY A 53 -4.637 14.686 13.632 1.00 1.00

ATOM 366 O GLY A 53 -5.736 15.142 13.963 1.00 1.00

ATOM 367 N ILE A 54 -4.476 13.476 13.098 1.00 1.00

ATOM 368 CA ILE A 54 -5.569 12.513 12.973 1.00 1.00

ATOM 369 C ILE A 54 -5.101 11.145 13.475 1.00 1.00

ATOM 370 O ILE A 54 -4.070 10.632 13.033 1.00 1.00

ATOM 371 CB ILE A 54 -6.084 12.413 11.509 1.00 1.00

ATOM 372 CG1 ILE A 54 -6.525 13.783 10.978 1.00 1.00

ATOM 373 CG2 ILE A 54 -7.240 11.420 11.413 1.00 1.00

ATOM 374 CD1 ILE A 54 -6.438 13.927 9.466 1.00 1.00

ATOM 375 N ASP A 55 -5.866 10.570 14.399 1.00 1.00

ATOM 376 CA ASP A 55 -5.539 9.276 14.991 1.00 1.00

ATOM 377 C ASP A 55 -6.551 8.209 14.580 1.00 1.00

ATOM 378 O ASP A 55 -7.730 8.517 14.407 1.00 1.00

ATOM 379 CB ASP A 55 -5.497 9.382 16.519 1.00 1.00

ATOM 380 CG ASP A 55 -4.502 10.415 17.010 1.00 1.00

ATOM 381 OD1 ASP A 55 -3.298 10.290 16.699 1.00 1.00

ATOM 382 OD2 ASP A 55 -4.839 11.387 17.718 1.00 1.00

ATOM 383 N PRO A 56 -6.098 6.965 14.416 1.00 1.00

ATOM 384 CA PRO A 56 -7.004 5.843 14.134 1.00 1.00

ATOM 385 C PRO A 56 -7.872 5.490 15.342 1.00 1.00

ATOM 386 O PRO A 56 -7.402 5.562 16.480 1.00 1.00

ATOM 387 CB PRO A 56 -6.048 4.689 13.815 1.00 1.00

ATOM 388 CG PRO A 56 -4.782 5.037 14.515 1.00 1.00

ATOM 389 CD PRO A 56 -4.688 6.533 14.466 1.00 1.00

ATOM 390 N GLY A 57 -9.123 5.120 15.086 1.00 1.00

ATOM 391 CA GLY A 57 -10.039 4.711 16.137 1.00 1.00

ATOM 392 C GLY A 57 -9.835 3.269 16.562 1.00 1.00

ATOM 393 O GLY A 57 -8.948 2.582 16.049 1.00 1.00

ATOM 394 N ASP A 58 -10.667 2.808 17.495 1.00 1.00

ATOM 395 CA ASP A 58 -10.531 1.463 18.059 1.00 1.00

ATOM 396 C ASP A 58 -10.989 0.336 17.127 1.00 1.00

ATOM 397 O ASP A 58 -10.685 -0.834 17.377 1.00 1.00

ATOM 398 CB ASP A 58 -11.233 1.353 19.425 1.00 1.00

ATOM 399 CG ASP A 58 -12.575 2.064 19.464 1.00 1.00

ATOM 400 OD1 ASP A 58 -13.460 1.738 18.643 1.00 1.00

ATOM 401 OD2 ASP A 58 -12.837 2.960 20.296 1.00 1.00

ATOM 402 N ILE A 59 -11.708 0.683 16.062 1.00 1.00

ATOM 403 CA ILE A 59 -12.164 -0.316 15.086 1.00 1.00

ATOM 404 C ILE A 59 -11.050 -0.760 14.128 1.00 1.00

ATOM 405 O ILE A 59 -11.219 -1.716 13.368 1.00 1.00

ATOM 406 CB ILE A 59 -13.439 0.153 14.320 1.00 1.00

ATOM 407 CG1 ILE A 59 -13.137 1.350 13.407 1.00 1.00

ATOM 408 CG2 ILE A 59 -14.582 0.440 15.303 1.00 1.00

ATOM 409 CD1 ILE A 59 -14.219 1.644 12.382 1.00 1.00

ATOM 410 N ILE A 60 -9.917 -0.060 14.176 1.00 1.00

ATOM 411 CA ILE A 60 -8.702 -0.509 13.505 1.00 1.00

ATOM 412 C ILE A 60 -7.897 -1.346 14.499 1.00 1.00

ATOM 413 O ILE A 60 -7.414 -0.831 15.513 1.00 1.00

ATOM 414 CB ILE A 60 -7.894 0.692 12.943 1.00 1.00

ATOM 415 CG1 ILE A 60 -8.543 1.200 11.651 1.00 1.00

ATOM 416 CG2 ILE A 60 -6.437 0.310 12.669 1.00 1.00

ATOM 417 CD1 ILE A 60 -8.824 2.682 11.648 1.00 1.00

ATOM 418 N VAL A 61 -7.776 -2.638 14.206 1.00 1.00

ATOM 419 CA VAL A 61 -7.240 -3.611 15.164 1.00 1.00

ATOM 420 C VAL A 61 -5.775 -3.994 14.933 1.00 1.00

ATOM 421 O VAL A 61 -5.186 -4.716 15.743 1.00 1.00

ATOM 422 CB VAL A 61 -8.123 -4.892 15.247 1.00 1.00

ATOM 423 CG1 VAL A 61 -9.499 -4.565 15.820 1.00 1.00

ATOM 424 CG2 VAL A 61 -8.246 -5.574 13.883 1.00 1.00

ATOM 425 N ASP A 62 -5.196 -3.512 13.832 1.00 1.00

ATOM 426 CA ASP A 62 -3.781 -3.724 13.526 1.00 1.00

ATOM 427 C ASP A 62 -2.908 -3.288 14.711 1.00 1.00

ATOM 428 O ASP A 62 -2.964 -2.126 15.124 1.00 1.00

ATOM 429 CB ASP A 62 -3.388 -2.944 12.260 1.00 1.00

ATOM 430 CG ASP A 62 -2.070 -3.411 11.650 1.00 1.00

ATOM 431 OD1 ASP A 62 -1.118 -3.729 12.396 1.00 1.00

ATOM 432 OD2 ASP A 62 -1.883 -3.479 10.417 1.00 1.00

ATOM 433 N PRO A 63 -2.128 -4.224 15.264 1.00 1.00

ATOM 434 CA PRO A 63 -1.211 -3.931 16.378 1.00 1.00

ATOM 435 C PRO A 63 -0.143 -2.897 16.012 1.00 1.00

ATOM 436 O PRO A 63 0.400 -2.232 16.899 1.00 1.00

ATOM 437 CB PRO A 63 -0.558 -5.289 16.664 1.00 1.00

ATOM 438 CG PRO A 63 -0.753 -6.083 15.418 1.00 1.00

ATOM 439 CD PRO A 63 -2.073 -5.644 14.871 1.00 1.00

ATOM 440 N ASN A 64 0.151 -2.787 14.717 1.00 1.00

ATOM 441 CA ASN A 64 0.983 -1.721 14.174 1.00 1.00

ATOM 442 C ASN A 64 0.116 -0.846 13.259 1.00 1.00

ATOM 443 O ASN A 64 0.188 -0.969 12.034 1.00 1.00

ATOM 444 CB ASN A 64 2.156 -2.315 13.388 1.00 1.00

ATOM 445 CG ASN A 64 3.499 -1.746 13.809 1.00 1.00

ATOM 446 OD1 ASN A 64 4.408 -2.490 14.180 1.00 1.00

ATOM 447 ND2 ASN A 64 3.637 -0.423 13.744 1.00 1.00

ATOM 448 N PRO A 65 -0.703 0.030 13.850 1.00 1.00

ATOM 449 CA PRO A 65 -1.763 0.731 13.108 1.00 1.00

ATOM 450 C PRO A 65 -1.287 1.559 11.913 1.00 1.00

ATOM 451 O PRO A 65 -2.055 1.707 10.960 1.00 1.00

ATOM 452 CB PRO A 65 -2.405 1.634 14.171 1.00 1.00

ATOM 453 CG PRO A 65 -1.394 1.743 15.251 1.00 1.00

ATOM 454 CD PRO A 65 -0.679 0.430 15.269 1.00 1.00

ATOM 455 N THR A 66 -0.057 2.069 11.955 1.00 1.00

ATOM 456 CA THR A 66 0.486 2.892 10.866 1.00 1.00

ATOM 457 C THR A 66 0.716 2.099 9.577 1.00 1.00

ATOM 458 O THR A 66 0.812 2.682 8.494 1.00 1.00

ATOM 459 CB THR A 66 1.794 3.599 11.294 1.00 1.00

ATOM 460 OG1 THR A 66 2.730 2.631 11.784 1.00 1.00

ATOM 461 CG2 THR A 66 1.555 4.519 12.491 1.00 1.00

ATOM 462 N LYS A 67 0.809 0.776 9.701 1.00 1.00

ATOM 463 CA LYS A 67 0.960 -0.108 8.546 1.00 1.00

ATOM 464 C LYS A 67 -0.332 -0.190 7.729 1.00 1.00

ATOM 465 O LYS A 67 -0.290 -0.364 6.510 1.00 1.00

ATOM 466 CB LYS A 67 1.399 -1.510 8.987 1.00 1.00

ATOM 467 CG LYS A 67 2.718 -1.548 9.752 1.00 1.00

ATOM 468 CD LYS A 67 3.770 -2.365 9.017 1.00 1.00

ATOM 469 CE LYS A 67 5.012 -2.564 9.877 1.00 1.00

ATOM 470 NZ LYS A 67 6.200 -1.861 9.313 1.00 1.00

ATOM 471 N SER A 68 -1.468 -0.051 8.412 1.00 1.00

ATOM 472 CA SER A 68 -2.788 -0.138 7.783 1.00 1.00

ATOM 473 C SER A 68 -3.482 1.216 7.613 1.00 1.00

ATOM 474 O SER A 68 -4.354 1.361 6.756 1.00 1.00

ATOM 475 CB SER A 68 -3.696 -1.071 8.590 1.00 1.00

ATOM 476 OG SER A 68 -3.262 -2.417 8.498 1.00 1.00

ATOM 477 N PHE A 69 -3.089 2.198 8.424 1.00 1.00

ATOM 478 CA PHE A 69 -3.785 3.484 8.503 1.00 1.00

ATOM 479 C PHE A 69 -2.818 4.656 8.346 1.00 1.00

ATOM 480 O PHE A 69 -1.797 4.715 9.030 1.00 1.00

ATOM 481 CB PHE A 69 -4.526 3.571 9.848 1.00 1.00

ATOM 482 CG PHE A 69 -5.353 4.820 10.027 1.00 1.00

ATOM 483 CD1 PHE A 69 -4.818 5.944 10.651 1.00 1.00

ATOM 484 CD2 PHE A 69 -6.681 4.857 9.608 1.00 1.00

ATOM 485 CE1 PHE A 69 -5.584 7.092 10.837 1.00 1.00

ATOM 486 CE2 PHE A 69 -7.453 6.002 9.788 1.00 1.00

ATOM 487 CZ PHE A 69 -6.903 7.122 10.406 1.00 1.00

ATOM 488 N ASP A 70 -3.149 5.583 7.444 1.00 1.00

ATOM 489 CA ASP A 70 -2.368 6.806 7.242 1.00 1.00

ATOM 490 C ASP A 70 -3.255 7.972 6.806 1.00 1.00

ATOM 491 O ASP A 70 -4.283 7.768 6.155 1.00 1.00

ATOM 492 CB ASP A 70 -1.247 6.586 6.215 1.00 1.00

ATOM 493 CG ASP A 70 -0.085 7.569 6.380 1.00 1.00

ATOM 494 OD1 ASP A 70 -0.192 8.525 7.181 1.00 1.00

ATOM 495 OD2 ASP A 70 0.985 7.459 5.747 1.00 1.00

ATOM 496 N THR A 71 -2.851 9.187 7.177 1.00 1.00

ATOM 497 CA THR A 71 -3.588 10.408 6.839 1.00 1.00

ATOM 498 C THR A 71 -2.666 11.528 6.350 1.00 1.00

ATOM 499 O THR A 71 -1.437 11.421 6.428 1.00 1.00

ATOM 500 CB THR A 71 -4.417 10.921 8.046 1.00 1.00

ATOM 501 OG1 THR A 71 -3.589 10.970 9.214 1.00 1.00

ATOM 502 CG2 THR A 71 -5.527 9.945 8.418 1.00 1.00

ATOM 503 N ALA A 72 -3.274 12.604 5.854 1.00 1.00

ATOM 504 CA ALA A 72 -2.549 13.800 5.431 1.00 1.00

ATOM 505 C ALA A 72 -3.406 15.051 5.611 1.00 1.00

ATOM 506 O ALA A 72 -4.617 15.022 5.370 1.00 1.00

ATOM 507 CB ALA A 72 -2.097 13.663 3.987 1.00 1.00

ATOM 508 N ILE A 73 -2.772 16.142 6.040 1.00 1.00

ATOM 509 CA ILE A 73 -3.456 17.418 6.268 1.00 1.00

ATOM 510 C ILE A 73 -2.869 18.515 5.372 1.00 1.00

ATOM 511 O ILE A 73 -1.659 18.753 5.390 1.00 1.00

ATOM 512 CB ILE A 73 -3.369 17.834 7.768 1.00 1.00

ATOM 513 CG1 ILE A 73 -3.973 16.756 8.674 1.00 1.00

ATOM 514 CG2 ILE A 73 -4.062 19.176 8.005 1.00 1.00

ATOM 515 CD1 ILE A 73 -3.241 16.574 9.994 1.00 1.00

ATOM 516 N TYR A 74 -3.730 19.175 4.600 1.00 1.00

ATOM 517 CA TYR A 74 -3.309 20.244 3.689 1.00 1.00

ATOM 518 C TYR A 74 -4.097 21.540 3.911 1.00 1.00

ATOM 519 O TYR A 74 -5.121 21.761 3.263 1.00 1.00

ATOM 520 CB TYR A 74 -3.439 19.797 2.226 1.00 1.00

ATOM 521 CG TYR A 74 -2.804 18.461 1.925 1.00 1.00

ATOM 522 CD1 TYR A 74 -3.590 17.332 1.704 1.00 1.00

ATOM 523 CD2 TYR A 74 -1.416 18.324 1.859 1.00 1.00

ATOM 524 CE1 TYR A 74 -3.015 16.103 1.426 1.00 1.00

ATOM 525 CE2 TYR A 74 -0.831 17.097 1.585 1.00 1.00

ATOM 526 CZ TYR A 74 -1.636 15.992 1.368 1.00 1.00

ATOM 527 OH TYR A 74 -1.067 14.772 1.096 1.00 1.00

ATOM 528 N PRO A 75 -3.624 22.388 4.829 1.00 1.00

ATOM 529 CA PRO A 75 -4.261 23.690 5.086 1.00 1.00

ATOM 530 C PRO A 75 -4.358 24.591 3.849 1.00 1.00

ATOM 531 O PRO A 75 -5.389 25.239 3.666 1.00 1.00

ATOM 532 CB PRO A 75 -3.359 24.327 6.156 1.00 1.00

ATOM 533 CG PRO A 75 -2.097 23.540 6.130 1.00 1.00

ATOM 534 CD PRO A 75 -2.470 22.157 5.716 1.00 1.00

ATOM 535 N ASP A 76 -3.307 24.587 2.992 1.00 1.00

ATOM 536 CA ASP A 76 -3.318 25.448 1.799 1.00 1.00

ATOM 537 C ASP A 76 -4.378 24.986 0.784 1.00 1.00

ATOM 538 O ASP A 76 -4.891 25.784 -0.008 1.00 1.00

ATOM 539 CB ASP A 76 -1.963 25.415 1.075 1.00 1.00

ATOM 540 CG ASP A 76 -0.796 25.400 2.038 1.00 1.00

ATOM 541 OD1 ASP A 76 -0.621 26.388 2.782 1.00 1.00

ATOM 542 OD2 ASP A 76 -0.051 24.398 2.047 1.00 1.00

ATOM 543 N ARG A 77 -4.694 23.694 0.821 1.00 1.00

ATOM 544 CA ARG A 77 -5.688 23.113 -0.114 1.00 1.00

ATOM 545 C ARG A 77 -7.053 22.902 0.585 1.00 1.00

ATOM 546 O ARG A 77 -8.032 22.493 -0.051 1.00 1.00

ATOM 547 CB ARG A 77 -5.180 21.782 -0.663 1.00 1.00

ATOM 548 CG ARG A 77 -4.321 21.971 -1.922 1.00 1.00

ATOM 549 CD ARG A 77 -4.010 20.666 -2.640 1.00 1.00

ATOM 550 NE ARG A 77 -2.914 19.939 -2.012 1.00 1.00

ATOM 551 CZ ARG A 77 -2.680 18.644 -2.179 1.00 1.00

ATOM 552 NH1 ARG A 77 -3.463 17.898 -2.975 1.00 1.00

ATOM 553 NH2 ARG A 77 -1.670 18.001 -1.584 1.00 1.00

ATOM 554 N LYS A 78 -7.060 23.192 1.880 1.00 1.00

ATOM 555 CA LYS A 78 -8.269 23.113 2.744 1.00 1.00

ATOM 556 C LYS A 78 -8.909 21.706 2.776 1.00 1.00

ATOM 557 O LYS A 78 -10.145 21.564 2.856 1.00 1.00

ATOM 558 CB LYS A 78 -9.328 24.078 2.224 1.00 1.00

ATOM 559 CG LYS A 78 -8.941 25.546 2.326 1.00 1.00

ATOM 560 CD LYS A 78 -8.200 26.015 1.084 1.00 1.00

ATOM 561 CE LYS A 78 -7.708 27.444 1.241 1.00 1.00

ATOM 562 NZ LYS A 78 -6.903 27.886 0.068 1.00 1.00

ATOM 563 N ILE A 79 -8.074 20.679 2.727 1.00 1.00

ATOM 564 CA ILE A 79 -8.564 19.280 2.749 1.00 1.00

ATOM 565 C ILE A 79 -7.718 18.398 3.680 1.00 1.00

ATOM 566 O ILE A 79 -6.561 18.704 3.989 1.00 1.00

ATOM 567 CB ILE A 79 -8.521 18.681 1.342 1.00 1.00

ATOM 568 CG1 ILE A 79 -7.186 18.936 0.618 1.00 1.00

ATOM 569 CG2 ILE A 79 -9.622 19.248 0.436 1.00 1.00

ATOM 570 CD1 ILE A 79 -6.749 17.772 -0.280 1.00 1.00

ATOM 571 N ILE A 80 -8.376 17.313 4.125 1.00 1.00

ATOM 572 CA ILE A 80 -7.708 16.224 4.836 1.00 1.00

ATOM 573 C ILE A 80 -8.018 14.900 4.138 1.00 1.00

ATOM 574 O ILE A 80 -9.115 14.716 3.605 1.00 1.00

ATOM 575 CB ILE A 80 -8.105 16.176 6.343 1.00 1.00

ATOM 576 CG1 ILE A 80 -9.629 16.180 6.523 1.00 1.00

ATOM 577 CG2 ILE A 80 -7.449 17.328 7.109 1.00 1.00

ATOM 578 CD1 ILE A 80 -10.104 15.680 7.884 1.00 1.00

ATOM 579 N VAL A 81 -7.048 13.990 4.125 1.00 1.00

ATOM 580 CA VAL A 81 -7.178 12.733 3.385 1.00 1.00

ATOM 581 C VAL A 81 -6.945 11.520 4.284 1.00 1.00

ATOM 582 O VAL A 81 -6.044 11.524 5.121 1.00 1.00

ATOM 583 CB VAL A 81 -6.216 12.668 2.164 1.00 1.00

ATOM 584 CG1 VAL A 81 -6.644 11.571 1.191 1.00 1.00

ATOM 585 CG2 VAL A 81 -6.138 14.015 1.446 1.00 1.00

ATOM 586 N PHE A 82 -7.763 10.489 4.092 1.00 1.00

ATOM 587 CA PHE A 82 -7.670 9.248 4.856 1.00 1.00

ATOM 588 C PHE A 82 -7.307 8.086 3.937 1.00 1.00

ATOM 589 O PHE A 82 -7.871 7.952 2.851 1.00 1.00

ATOM 590 CB PHE A 82 -9.005 8.942 5.547 1.00 1.00

ATOM 591 CG PHE A 82 -9.433 9.982 6.550 1.00 1.00

ATOM 592 CD1 PHE A 82 -10.060 11.153 6.136 1.00 1.00

ATOM 593 CD2 PHE A 82 -9.226 9.779 7.909 1.00 1.00

ATOM 594 CE1 PHE A 82 -10.462 12.113 7.062 1.00 1.00

ATOM 595 CE2 PHE A 82 -9.626 10.732 8.843 1.00 1.00

ATOM 596 CZ PHE A 82 -10.244 11.901 8.418 1.00 1.00

ATOM 597 N LEU A 83 -6.365 7.252 4.371 1.00 1.00

ATOM 598 CA LEU A 83 -6.029 6.031 3.639 1.00 1.00

ATOM 599 C LEU A 83 -5.953 4.817 4.558 1.00 1.00

ATOM 600 O LEU A 83 -5.056 4.709 5.397 1.00 1.00

ATOM 601 CB LEU A 83 -4.728 6.194 2.837 1.00 1.00

ATOM 602 CG LEU A 83 -4.128 4.965 2.132 1.00 1.00

ATOM 603 CD1 LEU A 83 -5.061 4.393 1.069 1.00 1.00

ATOM 604 CD2 LEU A 83 -2.773 5.292 1.523 1.00 1.00

ATOM 605 N PHE A 84 -6.911 3.910 4.390 1.00 1.00

ATOM 606 CA PHE A 84 -6.908 2.639 5.104 1.00 1.00

ATOM 607 C PHE A 84 -6.803 1.485 4.118 1.00 1.00

ATOM 608 O PHE A 84 -7.534 1.432 3.130 1.00 1.00

ATOM 609 CB PHE A 84 -8.163 2.485 5.972 1.00 1.00

ATOM 610 CG PHE A 84 -8.277 1.143 6.656 1.00 1.00

ATOM 611 CD1 PHE A 84 -7.495 0.843 7.770 1.00 1.00

ATOM 612 CD2 PHE A 84 -9.163 0.178 6.184 1.00 1.00

ATOM 613 CE1 PHE A 84 -7.593 -0.396 8.405 1.00 1.00

ATOM 614 CE2 PHE A 84 -9.272 -1.066 6.812 1.00 1.00

ATOM 615 CZ PHE A 84 -8.483 -1.351 7.925 1.00 1.00

ATOM 616 N ALA A 85 -5.871 0.578 4.395 1.00 1.00

ATOM 617 CA ALA A 85 -5.725 -0.669 3.652 1.00 1.00

ATOM 618 C ALA A 85 -5.080 -1.691 4.577 1.00 1.00

ATOM 619 O ALA A 85 -3.870 -1.642 4.819 1.00 1.00

ATOM 620 CB ALA A 85 -4.886 -0.462 2.394 1.00 1.00

ATOM 621 N GLU A 86 -5.897 -2.603 5.104 1.00 1.00

ATOM 622 CA GLU A 86 -5.441 -3.590 6.085 1.00 1.00

ATOM 623 C GLU A 86 -4.192 -4.332 5.600 1.00 1.00

ATOM 624 O GLU A 86 -4.129 -4.780 4.455 1.00 1.00

ATOM 625 CB GLU A 86 -6.580 -4.544 6.480 1.00 1.00

ATOM 626 CG GLU A 86 -6.933 -5.626 5.468 1.00 1.00

ATOM 627 CD GLU A 86 -6.156 -6.909 5.695 1.00 1.00

ATOM 628 OE1 GLU A 86 -5.828 -7.206 6.861 1.00 1.00

ATOM 629 OE2 GLU A 86 -5.865 -7.618 4.709 1.00 1.00

ATOM 630 N ASP A 87 -3.200 -4.442 6.480 1.00 1.00

ATOM 631 CA ASP A 87 -1.862 -4.884 6.088 1.00 1.00

ATOM 632 C ASP A 87 -1.496 -6.315 6.505 1.00 1.00

ATOM 633 O ASP A 87 -0.317 -6.682 6.516 1.00 1.00

ATOM 634 CB ASP A 87 -0.816 -3.887 6.608 1.00 1.00

ATOM 635 CG ASP A 87 0.461 -3.893 5.790 1.00 1.00

ATOM 636 OD1 ASP A 87 0.380 -3.906 4.543 1.00 1.00

ATOM 637 OD2 ASP A 87 1.595 -3.889 6.314 1.00 1.00

ATOM 638 N SER A 88 -2.501 -7.124 6.835 1.00 1.00

ATOM 639 CA SER A 88 -2.261 -8.515 7.233 1.00 1.00

ATOM 640 C SER A 88 -1.913 -9.402 6.041 1.00 1.00

ATOM 641 O SER A 88 -1.093 -10.314 6.158 1.00 1.00

ATOM 642 CB SER A 88 -3.467 -9.087 7.983 1.00 1.00

ATOM 643 OG SER A 88 -4.495 -9.475 7.088 1.00 1.00

ATOM 644 N GLY A 89 -2.548 -9.129 4.901 1.00 1.00

ATOM 645 CA GLY A 89 -2.381 -9.932 3.702 1.00 1.00

ATOM 646 C GLY A 89 -3.448 -11.002 3.560 1.00 1.00

ATOM 647 O GLY A 89 -3.565 -11.633 2.506 1.00 1.00

ATOM 648 N THR A 90 -4.227 -11.201 4.623 1.00 1.00

ATOM 649 CA THR A 90 -5.229 -12.270 4.684 1.00 1.00

ATOM 650 C THR A 90 -6.643 -11.740 4.925 1.00 1.00

ATOM 651 O THR A 90 -7.622 -12.474 4.766 1.00 1.00

ATOM 652 CB THR A 90 -4.868 -13.289 5.795 1.00 1.00

ATOM 653 OG1 THR A 90 -4.710 -12.603 7.044 1.00 1.00

ATOM 654 CG2 THR A 90 -3.494 -13.912 5.551 1.00 1.00

ATOM 655 N GLY A 91 -6.745 -10.471 5.312 1.00 1.00

ATOM 656 CA GLY A 91 -8.020 -9.869 5.664 1.00 1.00

ATOM 657 C GLY A 91 -8.254 -9.830 7.165 1.00 1.00

ATOM 658 O GLY A 91 -9.351 -9.497 7.618 1.00 1.00

ATOM 659 N ALA A 92 -7.216 -10.163 7.929 1.00 1.00

ATOM 660 CA ALA A 92 -7.306 -10.260 9.387 1.00 1.00

ATOM 661 C ALA A 92 -7.448 -8.906 10.084 1.00 1.00

ATOM 662 O ALA A 92 -8.075 -8.815 11.143 1.00 1.00

ATOM 663 CB ALA A 92 -6.108 -11.024 9.943 1.00 1.00

ATOM 664 N TYR A 93 -6.872 -7.861 9.492 1.00 1.00

ATOM 665 CA TYR A 93 -6.898 -6.525 10.093 1.00 1.00

ATOM 666 C TYR A 93 -7.986 -5.624 9.501 1.00 1.00

ATOM 667 O TYR A 93 -7.946 -4.400 9.652 1.00 1.00

ATOM 668 CB TYR A 93 -5.519 -5.855 10.003 1.00 1.00

ATOM 669 CG TYR A 93 -4.407 -6.589 10.732 1.00 1.00

ATOM 670 CD1 TYR A 93 -3.086 -6.492 10.297 1.00 1.00

ATOM 671 CD2 TYR A 93 -4.674 -7.372 11.860 1.00 1.00

ATOM 672 CE1 TYR A 93 -2.058 -7.161 10.960 1.00 1.00

ATOM 673 CE2 TYR A 93 -3.653 -8.044 12.528 1.00 1.00

ATOM 674 CZ TYR A 93 -2.349 -7.934 12.072 1.00 1.00

ATOM 675 OH TYR A 93 -1.337 -8.594 12.729 1.00 1.00

ATOM 676 N ALA A 94 -8.957 -6.239 8.830 1.00 1.00

ATOM 677 CA ALA A 94 -10.111 -5.522 8.292 1.00 1.00

ATOM 678 C ALA A 94 -11.007 -5.008 9.418 1.00 1.00

ATOM 679 O ALA A 94 -11.034 -5.582 10.509 1.00 1.00

ATOM 680 CB ALA A 94 -10.901 -6.423 7.353 1.00 1.00

ATOM 681 N ILE A 95 -11.706 -3.892 9.172 1.00 1.00

ATOM 682 CA ILE A 95 -12.706 -3.378 10.121 1.00 1.00

ATOM 683 C ILE A 95 -13.944 -4.275 10.054 1.00 1.00

ATOM 684 O ILE A 95 -14.535 -4.477 8.985 1.00 1.00

ATOM 685 CB ILE A 95 -13.048 -1.940 9.749 1.00 1.00

ATOM 686 CG1 ILE A 95 -11.900 -0.972 10.085 1.00 1.00

ATOM 687 CG2 ILE A 95 -14.286 -1.418 10.479 1.00 1.00

ATOM 688 CD1 ILE A 95 -11.808 0.223 9.135 1.00 1.00

ATOM 689 N THR A 96 -14.319 -4.802 11.209 1.00 1.00

ATOM 690 CA THR A 96 -15.449 -5.734 11.288 1.00 1.00

ATOM 691 C THR A 96 -16.627 -5.215 12.140 1.00 1.00

ATOM 692 O THR A 96 -17.740 -5.761 12.086 1.00 1.00

ATOM 693 CB THR A 96 -14.965 -7.061 11.893 1.00 1.00

ATOM 694 OG1 THR A 96 -14.261 -6.833 13.105 1.00 1.00

ATOM 695 CG2 THR A 96 -14.013 -7.821 10.963 1.00 1.00

ATOM 696 N LYS A 97 -16.408 -4.169 12.925 1.00 1.00

ATOM 697 CA LYS A 97 -17.497 -3.631 13.782 1.00 1.00

ATOM 698 C LYS A 97 -17.640 -2.103 13.658 1.00 1.00

ATOM 699 O LYS A 97 -16.715 -1.396 13.233 1.00 1.00

ATOM 700 CB LYS A 97 -17.232 -3.974 15.257 1.00 1.00

ATOM 701 CG LYS A 97 -16.122 -3.156 15.887 1.00 1.00

ATOM 702 CD LYS A 97 -16.019 -3.431 17.378 1.00 1.00

ATOM 703 CE LYS A 97 -17.316 -3.091 18.093 1.00 1.00

ATOM 704 NZ LYS A 97 -17.116 -2.933 19.561 1.00 1.00

ATOM 705 N ASP A 98 -18.827 -1.648 14.046 1.00 1.00

ATOM 706 CA ASP A 98 -19.196 -0.223 14.022 1.00 1.00

ATOM 707 C ASP A 98 -18.455 0.521 15.131 1.00 1.00

ATOM 708 O ASP A 98 -18.084 -0.069 16.156 1.00 1.00

ATOM 709 CB ASP A 98 -20.704 -0.062 14.238 1.00 1.00

ATOM 710 CG ASP A 98 -21.518 -0.365 12.982 1.00 1.00

ATOM 711 OD1 ASP A 98 -21.065 -1.194 12.111 1.00 1.00

ATOM 712 OD2 ASP A 98 -22.656 0.207 12.793 1.00 1.00

ATOM 713 N GLY A 99 -18.235 1.812 14.914 1.00 1.00

ATOM 714 CA GLY A 99 -17.588 2.654 15.905 1.00 1.00

ATOM 715 C GLY A 99 -16.713 3.737 15.310 1.00 1.00

ATOM 716 O GLY A 99 -16.900 4.144 14.162 1.00 1.00

ATOM 717 N VAL A 100 -15.750 4.199 16.102 1.00 1.00

ATOM 718 CA VAL A 100 -14.863 5.289 15.703 1.00 1.00

ATOM 719 C VAL A 100 -13.776 4.797 14.747 1.00 1.00

ATOM 720 O VAL A 100 -12.943 3.966 15.112 1.00 1.00

ATOM 721 CB VAL A 100 -14.220 5.985 16.933 1.00 1.00

ATOM 722 CG1 VAL A 100 -13.409 7.206 16.504 1.00 1.00

ATOM 723 CG2 VAL A 100 -15.284 6.383 17.953 1.00 1.00

ATOM 724 N PHE A 101 -13.805 5.315 13.521 1.00 1.00

ATOM 725 CA PHE A 101 -12.786 5.018 12.517 1.00 1.00

ATOM 726 C PHE A 101 -11.543 5.869 12.745 1.00 1.00

ATOM 727 O PHE A 101 -10.421 5.361 12.719 1.00 1.00

ATOM 728 CB PHE A 101 -13.345 5.239 11.104 1.00 1.00

ATOM 729 CG PHE A 101 -12.304 5.184 10.014 1.00 1.00

ATOM 730 CD1 PHE A 101 -11.718 3.974 9.649 1.00 1.00

ATOM 731 CD2 PHE A 101 -11.918 6.344 9.345 1.00 1.00

ATOM 732 CE1 PHE A 101 -10.759 3.922 8.639 1.00 1.00

ATOM 733 CE2 PHE A 101 -10.960 6.300 8.334 1.00 1.00

ATOM 734 CZ PHE A 101 -10.380 5.087 7.979 1.00 1.00

ATOM 735 N ALA A 102 -11.755 7.165 12.969 1.00 1.00

ATOM 736 CA ALA A 102 -10.666 8.112 13.182 1.00 1.00

ATOM 737 C ALA A 102 -11.079 9.253 14.108 1.00 1.00

ATOM 738 O ALA A 102 -12.267 9.545 14.259 1.00 1.00

ATOM 739 CB ALA A 102 -10.169 8.660 11.848 1.00 1.00

ATOM 740 N LYS A 103 -10.087 9.889 14.726 1.00 1.00

ATOM 741 CA LYS A 103 -10.311 11.058 15.570 1.00 1.00

ATOM 742 C LYS A 103 -9.498 12.237 15.042 1.00 1.00

ATOM 743 O LYS A 103 -8.267 12.181 14.999 1.00 1.00

ATOM 744 CB LYS A 103 -9.927 10.764 17.023 1.00 1.00

ATOM 745 CG LYS A 103 -10.616 9.545 17.611 1.00 1.00

ATOM 746 CD LYS A 103 -11.740 9.946 18.551 1.00 1.00

ATOM 747 CE LYS A 103 -12.190 8.773 19.407 1.00 1.00

ATOM 748 NZ LYS A 103 -12.729 9.219 20.721 1.00 1.00

ATOM 749 N ILE A 104 -10.197 13.293 14.634 1.00 1.00

ATOM 750 CA ILE A 104 -9.550 14.505 14.141 1.00 1.00

ATOM 751 C ILE A 104 -9.290 15.462 15.301 1.00 1.00

ATOM 752 O ILE A 104 -10.224 15.918 15.963 1.00 1.00

ATOM 753 CB ILE A 104 -10.406 15.196 13.043 1.00 1.00

ATOM 754 CG1 ILE A 104 -10.749 14.219 11.914 1.00 1.00

ATOM 755 CG2 ILE A 104 -9.679 16.427 12.488 1.00 1.00

ATOM 756 CD1 ILE A 104 -12.005 14.584 11.138 1.00 1.00

ATOM 757 N ARG A 105 -8.016 15.752 15.546 1.00 1.00

ATOM 758 CA ARG A 105 -7.632 16.715 16.572 1.00 1.00

ATOM 759 C ARG A 105 -7.489 18.097 15.945 1.00 1.00

ATOM 760 O ARG A 105 -6.650 18.304 15.063 1.00 1.00

ATOM 761 CB ARG A 105 -6.332 16.289 17.258 1.00 1.00

ATOM 762 CG ARG A 105 -6.524 15.267 18.369 1.00 1.00

ATOM 763 CD ARG A 105 -5.234 14.848 19.059 1.00 1.00

ATOM 764 NE ARG A 105 -4.429 13.962 18.219 1.00 1.00

ATOM 765 CZ ARG A 105 -3.262 14.286 17.673 1.00 1.00

ATOM 766 NH1 ARG A 105 -2.735 15.489 17.871 1.00 1.00

ATOM 767 NH2 ARG A 105 -2.616 13.402 16.925 1.00 1.00

ATOM 768 N ALA A 106 -8.321 19.031 16.396 1.00 1.00

ATOM 769 CA ALA A 106 -8.329 20.386 15.852 1.00 1.00

ATOM 770 C ALA A 106 -8.358 21.449 16.943 1.00 1.00

ATOM 771 O ALA A 106 -9.085 21.323 17.931 1.00 1.00

ATOM 772 CB ALA A 106 -9.505 20.570 14.901 1.00 1.00

ATOM 773 N THR A 107 -7.533 22.468 16.765 1.00 1.00

ATOM 774 CA THR A 107 -7.584 23.629 17.663 1.00 1.00

ATOM 775 C THR A 107 -8.496 24.704 17.049 1.00 1.00

ATOM 776 O THR A 107 -8.288 25.151 15.914 1.00 1.00

ATOM 777 CB THR A 107 -6.164 24.186 17.854 1.00 1.00

ATOM 778 OG1 THR A 107 -5.296 23.159 18.314 1.00 1.00

ATOM 779 CG2 THR A 107 -6.093 25.324 18.874 1.00 1.00

ATOM 780 N VAL A 108 -9.496 25.092 17.826 1.00 1.00

ATOM 781 CA VAL A 108 -10.478 26.112 17.408 1.00 1.00

ATOM 782 C VAL A 108 -9.811 27.493 17.414 1.00 1.00

ATOM 783 O VAL A 108 -9.330 27.968 18.453 1.00 1.00

ATOM 784 CB VAL A 108 -11.668 26.104 18.366 1.00 1.00

ATOM 785 CG1 VAL A 108 -12.824 26.983 17.885 1.00 1.00

ATOM 786 CG2 VAL A 108 -12.261 24.704 18.564 1.00 1.00

ATOM 787 N LYS A 109 -9.803 28.105 16.237 1.00 1.00

ATOM 788 CA LYS A 109 -9.169 29.425 16.028 1.00 1.00

ATOM 789 C LYS A 109 -10.176 30.593 16.048 1.00 1.00

ATOM 790 O LYS A 109 -9.808 31.744 16.314 1.00 1.00

ATOM 791 CB LYS A 109 -8.461 29.482 14.669 1.00 1.00

ATOM 792 CG LYS A 109 -7.489 28.341 14.432 1.00 1.00

ATOM 793 CD LYS A 109 -6.058 28.771 14.708 1.00 1.00

ATOM 794 CE LYS A 109 -5.359 29.221 13.435 1.00 1.00

ATOM 795 NZ LYS A 109 -4.039 29.849 13.718 1.00 1.00

ATOM 796 N SER A 110 -11.436 30.302 15.759 1.00 1.00

ATOM 797 CA SER A 110 -12.482 31.354 15.732 1.00 1.00

ATOM 798 C SER A 110 -13.859 30.775 16.071 1.00 1.00

ATOM 799 O SER A 110 -14.041 29.554 16.157 1.00 1.00

ATOM 800 CB SER A 110 -12.542 31.992 14.336 1.00 1.00

ATOM 801 OG SER A 110 -13.792 31.716 13.720 1.00 1.00

ATOM 802 N SER A 111 -14.793 31.707 16.423 1.00 1.00

ATOM 803 CA SER A 111 -16.133 31.351 16.887 1.00 1.00

ATOM 804 C SER A 111 -17.076 30.911 15.763 1.00 1.00

ATOM 805 O SER A 111 -18.142 30.351 16.027 1.00 1.00

ATOM 806 CB SER A 111 -16.751 32.518 17.660 1.00 1.00

ATOM 807 OG SER A 111 -16.996 33.620 16.805 1.00 1.00

ATOM 808 N ALA A 112 -16.678 31.177 14.521 1.00 1.00

ATOM 809 CA ALA A 112 -17.452 30.804 13.339 1.00 1.00

ATOM 810 C ALA A 112 -17.609 29.283 13.227 1.00 1.00

ATOM 811 O ALA A 112 -16.764 28.540 13.737 1.00 1.00

ATOM 812 CB ALA A 112 -16.796 31.370 12.080 1.00 1.00

ATOM 813 N PRO A 113 -18.684 28.818 12.584 1.00 1.00

ATOM 814 CA PRO A 113 -18.892 27.379 12.372 1.00 1.00

ATOM 815 C PRO A 113 -17.728 26.727 11.626 1.00 1.00

ATOM 816 O PRO A 113 -17.291 27.233 10.588 1.00 1.00

ATOM 817 CB PRO A 113 -20.168 27.331 11.525 1.00 1.00

ATOM 818 CG PRO A 113 -20.876 28.604 11.832 1.00 1.00

ATOM 819 CD PRO A 113 -19.794 29.620 12.033 1.00 1.00

ATOM 820 N GLY A 114 -17.226 25.623 12.175 1.00 1.00

ATOM 821 CA GLY A 114 -16.120 24.891 11.585 1.00 1.00

ATOM 822 C GLY A 114 -16.594 23.625 10.905 1.00 1.00

ATOM 823 O GLY A 114 -16.533 22.541 11.485 1.00 1.00

ATOM 824 N TYR A 115 -17.068 23.772 9.669 1.00 1.00

ATOM 825 CA TYR A 115 -17.593 22.656 8.894 1.00 1.00

ATOM 826 C TYR A 115 -16.494 21.693 8.452 1.00 1.00

ATOM 827 O TYR A 115 -15.465 22.112 7.915 1.00 1.00

ATOM 828 CB TYR A 115 -18.350 23.162 7.659 1.00 1.00

ATOM 829 CG TYR A 115 -19.454 24.156 7.948 1.00 1.00

ATOM 830 CD1 TYR A 115 -19.287 25.512 7.667 1.00 1.00

ATOM 831 CD2 TYR A 115 -20.672 23.741 8.487 1.00 1.00

ATOM 832 CE1 TYR A 115 -20.300 26.433 7.926 1.00 1.00

ATOM 833 CE2 TYR A 115 -21.692 24.651 8.752 1.00 1.00

ATOM 834 CZ TYR A 115 -21.500 25.993 8.465 1.00 1.00

ATOM 835 OH TYR A 115 -22.504 26.897 8.723 1.00 1.00

ATOM 836 N ILE A 116 -16.718 20.405 8.701 1.00 1.00

ATOM 837 CA ILE A 116 -15.913 19.343 8.104 1.00 1.00

ATOM 838 C ILE A 116 -16.845 18.493 7.241 1.00 1.00

ATOM 839 O ILE A 116 -17.669 17.728 7.752 1.00 1.00

ATOM 840 CB ILE A 116 -15.175 18.501 9.178 1.00 1.00

ATOM 841 CG1 ILE A 116 -14.211 19.386 9.981 1.00 1.00

ATOM 842 CG2 ILE A 116 -14.408 17.349 8.528 1.00 1.00

ATOM 843 CD1 ILE A 116 -13.829 18.833 11.341 1.00 1.00

ATOM 844 N THR A 117 -16.721 18.660 5.927 1.00 1.00

ATOM 845 CA THR A 117 -17.668 18.089 4.973 1.00 1.00

ATOM 846 C THR A 117 -17.020 17.068 4.046 1.00 1.00

ATOM 847 O THR A 117 -15.827 17.150 3.749 1.00 1.00

ATOM 848 CB THR A 117 -18.327 19.202 4.130 1.00 1.00

ATOM 849 OG1 THR A 117 -17.313 20.025 3.543 1.00 1.00

ATOM 850 CG2 THR A 117 -19.108 20.171 5.014 1.00 1.00

ATOM 851 N PHE A 118 -17.826 16.113 3.589 1.00 1.00

ATOM 852 CA PHE A 118 -17.391 15.104 2.630 1.00 1.00

ATOM 853 C PHE A 118 -17.104 15.744 1.274 1.00 1.00

ATOM 854 O PHE A 118 -17.915 16.518 0.756 1.00 1.00

ATOM 855 CB PHE A 118 -18.456 14.006 2.510 1.00 1.00

ATOM 856 CG PHE A 118 -18.348 13.172 1.258 1.00 1.00

ATOM 857 CD1 PHE A 118 -17.282 12.295 1.074 1.00 1.00

ATOM 858 CD2 PHE A 118 -19.327 13.253 0.270 1.00 1.00

ATOM 859 CE1 PHE A 118 -17.186 11.522 -0.081 1.00 1.00

ATOM 860 CE2 PHE A 118 -19.240 12.483 -0.886 1.00 1.00

ATOM 861 CZ PHE A 118 -18.166 11.615 -1.061 1.00 1.00

ATOM 862 N ASP A 119 -15.944 15.418 0.713 1.00 1.00

ATOM 863 CA ASP A 119 -15.514 15.978 -0.566 1.00 1.00

ATOM 864 C ASP A 119 -15.462 14.911 -1.662 1.00 1.00

ATOM 865 O ASP A 119 -16.107 15.053 -2.702 1.00 1.00

ATOM 866 CB ASP A 119 -14.158 16.683 -0.409 1.00 1.00

ATOM 867 CG ASP A 119 -13.710 17.398 -1.676 1.00 1.00

ATOM 868 OD1 ASP A 119 -14.570 17.895 -2.438 1.00 1.00

ATOM 869 OD2 ASP A 119 -12.507 17.518 -1.988 1.00 1.00

ATOM 870 N GLU A 120 -14.698 13.847 -1.419 1.00 1.00

ATOM 871 CA GLU A 120 -14.505 12.785 -2.402 1.00 1.00

ATOM 872 C GLU A 120 -14.289 11.436 -1.726 1.00 1.00

ATOM 873 O GLU A 120 -13.698 11.365 -0.651 1.00 1.00

ATOM 874 CB GLU A 120 -13.307 13.111 -3.306 1.00 1.00

ATOM 875 CG GLU A 120 -13.209 12.262 -4.568 1.00 1.00

ATOM 876 CD GLU A 120 -12.208 11.122 -4.448 1.00 1.00

ATOM 877 OE1 GLU A 120 -12.066 10.357 -5.427 1.00 1.00

ATOM 878 OE2 GLU A 120 -11.562 10.982 -3.386 1.00 1.00

ATOM 879 N VAL A 121 -14.787 10.376 -2.359 1.00 1.00

ATOM 880 CA VAL A 121 -14.524 9.006 -1.922 1.00 1.00

ATOM 881 C VAL A 121 -13.907 8.196 -3.072 1.00 1.00

ATOM 882 O VAL A 121 -14.329 8.315 -4.224 1.00 1.00

ATOM 883 CB VAL A 121 -15.801 8.317 -1.341 1.00 1.00

ATOM 884 CG1 VAL A 121 -16.883 8.108 -2.411 1.00 1.00

ATOM 885 CG2 VAL A 121 -15.451 7.003 -0.643 1.00 1.00

ATOM 886 N GLY A 122 -12.891 7.399 -2.752 1.00 1.00

ATOM 887 CA GLY A 122 -12.237 6.552 -3.733 1.00 1.00

ATOM 888 C GLY A 122 -12.717 5.119 -3.629 1.00 1.00

ATOM 889 O GLY A 122 -13.844 4.806 -4.012 1.00 1.00

ATOM 890 N GLY A 123 -11.854 4.253 -3.106 1.00 1.00

ATOM 891 CA GLY A 123 -12.196 2.863 -2.862 1.00 1.00

ATOM 892 C GLY A 123 -12.851 2.669 -1.507 1.00 1.00

ATOM 893 O GLY A 123 -12.618 3.447 -0.577 1.00 1.00

ATOM 894 N PHE A 124 -13.677 1.630 -1.406 1.00 1.00

ATOM 895 CA PHE A 124 -14.381 1.276 -0.174 1.00 1.00

ATOM 896 C PHE A 124 -14.875 -0.163 -0.301 1.00 1.00

ATOM 897 O PHE A 124 -16.054 -0.404 -0.563 1.00 1.00

ATOM 898 CB PHE A 124 -15.558 2.231 0.071 1.00 1.00

ATOM 899 CG PHE A 124 -15.754 2.604 1.516 1.00 1.00

ATOM 900 CD1 PHE A 124 -15.490 3.899 1.956 1.00 1.00

ATOM 901 CD2 PHE A 124 -16.218 1.668 2.438 1.00 1.00

ATOM 902 CE1 PHE A 124 -15.670 4.253 3.290 1.00 1.00

ATOM 903 CE2 PHE A 124 -16.403 2.013 3.777 1.00 1.00

ATOM 904 CZ PHE A 124 -16.128 3.309 4.202 1.00 1.00

ATOM 905 N ALA A 125 -13.962 -1.115 -0.116 1.00 1.00

ATOM 906 CA ALA A 125 -14.236 -2.518 -0.423 1.00 1.00

ATOM 907 C ALA A 125 -14.163 -3.452 0.784 1.00 1.00

ATOM 908 O ALA A 125 -13.469 -3.168 1.764 1.00 1.00

ATOM 909 CB ALA A 125 -13.302 -3.006 -1.531 1.00 1.00

ATOM 910 N ASP A 126 -14.885 -4.570 0.693 1.00 1.00

ATOM 911 CA ASP A 126 -14.852 -5.608 1.721 1.00 1.00

ATOM 912 C ASP A 126 -13.692 -6.586 1.508 1.00 1.00

ATOM 913 O ASP A 126 -12.838 -6.363 0.646 1.00 1.00

ATOM 914 CB ASP A 126 -16.202 -6.346 1.812 1.00 1.00

ATOM 915 CG ASP A 126 -16.616 -7.016 0.502 1.00 1.00

ATOM 916 OD1 ASP A 126 -15.743 -7.434 -0.288 1.00 1.00

ATOM 917 OD2 ASP A 126 -17.813 -7.182 0.189 1.00 1.00

ATOM 918 N ASN A 127 -13.729 -7.721 2.215 1.00 1.00

ATOM 919 CA ASN A 127 -12.597 -8.656 2.197 1.00 1.00

ATOM 920 C ASN A 127 -12.572 -9.449 0.879 1.00 1.00

ATOM 921 O ASN A 127 -11.578 -10.107 0.546 1.00 1.00

ATOM 922 CB ASN A 127 -12.710 -9.640 3.362 1.00 1.00

ATOM 923 CG ASN A 127 -11.971 -9.163 4.614 1.00 1.00

ATOM 924 OD1 ASN A 127 -10.893 -8.580 4.509 1.00 1.00

ATOM 925 ND2 ASN A 127 -12.490 -9.380 5.809 1.00 1.00

ATOM 926 N ASP A 128 -13.677 -9.371 0.147 1.00 1.00

ATOM 927 CA ASP A 128 -13.812 -10.073 -1.147 1.00 1.00

ATOM 928 C ASP A 128 -13.547 -9.089 -2.295 1.00 1.00

ATOM 929 O ASP A 128 -13.827 -9.387 -3.471 1.00 1.00

ATOM 930 CB ASP A 128 -15.210 -10.668 -1.277 1.00 1.00

ATOM 931 CG ASP A 128 -15.409 -11.876 -0.354 1.00 1.00

ATOM 932 OD1 ASP A 128 -14.398 -12.609 -0.024 1.00 1.00

ATOM 933 OD2 ASP A 128 -16.582 -12.160 0.094 1.00 1.00

ATOM 934 N LEU A 129 -13.013 -7.953 -1.885 1.00 1.00

ATOM 935 CA LEU A 129 -12.613 -6.860 -2.785 1.00 1.00

ATOM 936 C LEU A 129 -13.829 -6.298 -3.592 1.00 1.00

ATOM 937 O LEU A 129 -13.677 -5.768 -4.707 1.00 1.00

ATOM 938 CB LEU A 129 -11.535 -7.411 -3.734 1.00 1.00

ATOM 939 CG LEU A 129 -10.090 -7.259 -3.267 1.00 1.00

ATOM 940 CD1 LEU A 129 -9.129 -7.554 -4.408 1.00 1.00

ATOM 941 CD2 LEU A 129 -9.828 -5.843 -2.779 1.00 1.00

ATOM 942 N VAL A 130 -15.018 -6.427 -3.003 1.00 1.00

ATOM 943 CA VAL A 130 -16.273 -5.901 -3.605 1.00 1.00

ATOM 944 C VAL A 130 -16.510 -4.491 -3.051 1.00 1.00

ATOM 945 O VAL A 130 -16.544 -4.283 -1.831 1.00 1.00

ATOM 946 CB VAL A 130 -17.476 -6.791 -3.243 1.00 1.00

ATOM 947 CG1 VAL A 130 -18.807 -6.244 -3.779 1.00 1.00

ATOM 948 CG2 VAL A 130 -17.368 -8.213 -3.793 1.00 1.00

ATOM 949 N GLU A 131 -16.667 -3.540 -3.956 1.00 1.00

ATOM 950 CA GLU A 131 -16.888 -2.128 -3.580 1.00 1.00

ATOM 951 C GLU A 131 -18.280 -1.950 -2.964 1.00 1.00

ATOM 952 O GLU A 131 -19.293 -2.367 -3.540 1.00 1.00

ATOM 953 CB GLU A 131 -16.771 -1.222 -4.810 1.00 1.00

ATOM 954 CG GLU A 131 -15.487 -1.461 -5.615 1.00 1.00

ATOM 955 CD GLU A 131 -15.697 -2.344 -6.850 1.00 1.00

ATOM 956 OE1 GLU A 131 -16.622 -3.243 -6.854 1.00 1.00

ATOM 957 OE2 GLU A 131 -14.947 -2.188 -7.889 1.00 1.00

ATOM 958 N GLN A 132 -18.301 -1.340 -1.786 1.00 1.00

ATOM 959 CA GLN A 132 -19.536 -1.222 -0.996 1.00 1.00

ATOM 960 C GLN A 132 -20.203 0.129 -1.227 1.00 1.00

ATOM 961 O GLN A 132 -19.540 1.126 -1.558 1.00 1.00

ATOM 962 CB GLN A 132 -19.227 -1.317 0.511 1.00 1.00

ATOM 963 CG GLN A 132 -18.485 -2.588 0.913 1.00 1.00

ATOM 964 CD GLN A 132 -19.322 -3.847 0.712 1.00 1.00

ATOM 965 OE1 GLN A 132 -20.295 -4.060 1.434 1.00 1.00

ATOM 966 NE2 GLN A 132 -18.992 -4.700 -0.237 1.00 1.00

ATOM 967 N LYS A 133 -21.514 0.143 -1.053 1.00 1.00

ATOM 968 CA LYS A 133 -22.273 1.384 -1.171 1.00 1.00

ATOM 969 C LYS A 133 -21.985 2.183 0.086 1.00 1.00

ATOM 970 O LYS A 133 -22.031 1.651 1.205 1.00 1.00

ATOM 971 CB LYS A 133 -23.760 1.126 -1.287 1.00 1.00

ATOM 972 CG LYS A 133 -24.208 0.578 -2.639 1.00 1.00

ATOM 973 CD LYS A 133 -23.889 -0.901 -2.784 1.00 1.00

ATOM 974 CE LYS A 133 -23.728 -1.291 -4.245 1.00 1.00

ATOM 975 NZ LYS A 133 -22.477 -2.066 -4.477 1.00 1.00

ATOM 976 N VAL A 134 -21.686 3.451 -0.083 1.00 1.00

ATOM 977 CA VAL A 134 -21.339 4.287 1.069 1.00 1.00

ATOM 978 C VAL A 134 -21.912 5.712 0.946 1.00 1.00

ATOM 979 O VAL A 134 -21.946 6.311 -0.138 1.00 1.00

ATOM 980 CB VAL A 134 -19.801 4.342 1.174 1.00 1.00

ATOM 981 CG1 VAL A 134 -19.138 5.184 0.072 1.00 1.00

ATOM 982 CG2 VAL A 134 -19.309 4.931 2.496 1.00 1.00

ATOM 983 N SER A 135 -22.351 6.210 2.097 1.00 1.00

ATOM 984 CA SER A 135 -22.927 7.559 2.228 1.00 1.00

ATOM 985 C SER A 135 -22.251 8.295 3.389 1.00 1.00

ATOM 986 O SER A 135 -21.669 7.670 4.290 1.00 1.00

ATOM 987 CB SER A 135 -24.439 7.481 2.496 1.00 1.00

ATOM 988 OG SER A 135 -24.694 6.787 3.708 1.00 1.00

ATOM 989 N PHE A 136 -22.236 9.619 3.300 1.00 1.00

ATOM 990 CA PHE A 136 -21.495 10.458 4.233 1.00 1.00

ATOM 991 C PHE A 136 -22.388 11.512 4.879 1.00 1.00

ATOM 992 O PHE A 136 -23.202 12.146 4.206 1.00 1.00

ATOM 993 CB PHE A 136 -20.327 11.143 3.515 1.00 1.00

ATOM 994 CG PHE A 136 -19.212 10.209 3.118 1.00 1.00

ATOM 995 CD1 PHE A 136 -19.315 9.419 1.971 1.00 1.00

ATOM 996 CD2 PHE A 136 -18.050 10.133 3.875 1.00 1.00

ATOM 997 CE1 PHE A 136 -18.286 8.557 1.600 1.00 1.00

ATOM 998 CE2 PHE A 136 -17.012 9.276 3.511 1.00 1.00

ATOM 999 CZ PHE A 136 -17.131 8.486 2.370 1.00 1.00

ATOM 1000 N ILE A 137 -22.228 11.691 6.189 1.00 1.00

ATOM 1001 CA ILE A 137 -22.909 12.761 6.918 1.00 1.00

ATOM 1002 C ILE A 137 -21.886 13.792 7.394 1.00 1.00

ATOM 1003 O ILE A 137 -20.936 13.457 8.103 1.00 1.00

ATOM 1004 CB ILE A 137 -23.743 12.195 8.101 1.00 1.00

ATOM 1005 CG1 ILE A 137 -24.949 11.406 7.577 1.00 1.00

ATOM 1006 CG2 ILE A 137 -24.208 13.318 9.037 1.00 1.00

ATOM 1007 CD1 ILE A 137 -25.541 10.425 8.577 1.00 1.00

ATOM 1008 N ASP A 138 -22.089 15.042 6.984 1.00 1.00

ATOM 1009 CA ASP A 138 -21.186 16.141 7.329 1.00 1.00

ATOM 1010 C ASP A 138 -21.325 16.557 8.791 1.00 1.00

ATOM 1011 O ASP A 138 -22.393 16.404 9.388 1.00 1.00

ATOM 1012 CB ASP A 138 -21.434 17.346 6.413 1.00 1.00

ATOM 1013 CG ASP A 138 -21.171 17.039 4.946 1.00 1.00

ATOM 1014 OD1 ASP A 138 -20.499 16.027 4.644 1.00 1.00

ATOM 1015 OD2 ASP A 138 -21.595 17.761 4.020 1.00 1.00

ATOM 1016 N GLY A 139 -20.236 17.079 9.353 1.00 1.00

ATOM 1017 CA GLY A 139 -20.214 17.534 10.733 1.00 1.00

ATOM 1018 C GLY A 139 -19.223 18.659 10.963 1.00 1.00

ATOM 1019 O GLY A 139 -19.099 19.566 10.135 1.00 1.00

ATOM 1020 N GLY A 140 -18.523 18.600 12.093 1.00 1.00

ATOM 1021 CA GLY A 140 -17.502 19.578 12.418 1.00 1.00

ATOM 1022 C GLY A 140 -17.576 20.128 13.831 1.00 1.00

ATOM 1023 O GLY A 140 -18.039 19.450 14.752 1.00 1.00

ATOM 1024 N VAL A 141 -17.111 21.366 13.993 1.00 1.00

ATOM 1025 CA VAL A 141 -17.039 22.028 15.294 1.00 1.00

ATOM 1026 C VAL A 141 -17.916 23.279 15.305 1.00 1.00

ATOM 1027 O VAL A 141 -17.822 24.117 14.404 1.00 1.00

ATOM 1028 CB VAL A 141 -15.578 22.410 15.664 1.00 1.00

ATOM 1029 CG1 VAL A 141 -15.505 23.043 17.049 1.00 1.00

ATOM 1030 CG2 VAL A 141 -14.665 21.195 15.594 1.00 1.00

ATOM 1031 N ASN A 142 -18.760 23.390 16.332 1.00 1.00

ATOM 1032 CA ASN A 142 -19.681 24.519 16.504 1.00 1.00

ATOM 1033 C ASN A 142 -20.561 24.775 15.277 1.00 1.00

ATOM 1034 O ASN A 142 -20.731 25.919 14.845 1.00 1.00

ATOM 1035 CB ASN A 142 -18.922 25.793 16.910 1.00 1.00

ATOM 1036 CG ASN A 142 -18.133 25.620 18.195 1.00 1.00

ATOM 1037 OD1 ASN A 142 -18.637 25.081 19.181 1.00 1.00

ATOM 1038 ND2 ASN A 142 -16.890 26.085 18.192 1.00 1.00

ATOM 1039 N VAL A 143 -21.113 23.697 14.727 1.00 1.00

ATOM 1040 CA VAL A 143 -21.924 23.769 13.508 1.00 1.00

ATOM 1041 C VAL A 143 -23.428 23.658 13.785 1.00 1.00

ATOM 1042 O VAL A 143 -24.244 23.748 12.863 1.00 1.00

ATOM 1043 CB VAL A 143 -21.485 22.708 12.454 1.00 1.00

ATOM 1044 CG1 VAL A 143 -20.057 22.973 11.987 1.00 1.00

ATOM 1045 CG2 VAL A 143 -21.624 21.288 13.001 1.00 1.00

ATOM 1046 N GLY A 144 -23.785 23.466 15.054 1.00 1.00

ATOM 1047 CA GLY A 144 -25.175 23.346 15.457 1.00 1.00

ATOM 1048 C GLY A 144 -25.799 24.685 15.796 1.00 1.00

ATOM 1049 O GLY A 144 -26.886 24.743 16.374 1.00 1.00

ATOM 1050 OXT GLY A 144 -25.186 25.720 15.442 1.00 1.00

TER

ATOM 1051 N GLY B 1 1.723 -2.365 -11.801 1.00 1.00

ATOM 1052 CA GLY B 1 2.369 -2.329 -10.492 1.00 1.00

ATOM 1053 C GLY B 1 3.667 -1.544 -10.464 1.00 1.00

ATOM 1054 O GLY B 1 4.097 -1.096 -9.405 1.00 1.00

ATOM 1055 N ASP B 2 4.294 -1.378 -11.625 1.00 1.00

ATOM 1056 CA ASP B 2 5.544 -0.628 -11.733 1.00 1.00

ATOM 1057 C ASP B 2 5.267 0.872 -11.839 1.00 1.00

ATOM 1058 O ASP B 2 5.574 1.506 -12.851 1.00 1.00

ATOM 1059 CB ASP B 2 6.371 -1.130 -12.927 1.00 1.00

ATOM 1060 CG ASP B 2 7.707 -0.408 -13.066 1.00 1.00

ATOM 1061 OD1 ASP B 2 8.203 0.155 -12.069 1.00 1.00

ATOM 1062 OD2 ASP B 2 8.266 -0.404 -14.180 1.00 1.00

ATOM 1063 N VAL B 3 4.708 1.434 -10.772 1.00 1.00

ATOM 1064 CA VAL B 3 4.257 2.823 -10.761 1.00 1.00

ATOM 1065 C VAL B 3 5.295 3.805 -11.332 1.00 1.00

ATOM 1066 O VAL B 3 4.979 4.572 -12.239 1.00 1.00

ATOM 1067 CB VAL B 3 3.792 3.269 -9.348 1.00 1.00

ATOM 1068 CG1 VAL B 3 3.229 4.682 -9.391 1.00 1.00

ATOM 1069 CG2 VAL B 3 2.747 2.297 -8.785 1.00 1.00

ATOM 1070 N ASN B 4 6.526 3.762 -10.822 1.00 1.00

ATOM 1071 CA ASN B 4 7.564 4.716 -11.246 1.00 1.00

ATOM 1072 C ASN B 4 8.236 4.386 -12.582 1.00 1.00

ATOM 1073 O ASN B 4 9.063 5.159 -13.078 1.00 1.00

ATOM 1074 CB ASN B 4 8.603 4.941 -10.135 1.00 1.00

ATOM 1075 CG ASN B 4 9.489 3.725 -9.891 1.00 1.00

ATOM 1076 OD1 ASN B 4 9.400 2.718 -10.593 1.00 1.00

ATOM 1077 ND2 ASN B 4 10.363 3.825 -8.894 1.00 1.00

ATOM 1078 N GLY B 5 7.889 3.234 -13.149 1.00 1.00

ATOM 1079 CA GLY B 5 8.367 2.824 -14.467 1.00 1.00

ATOM 1080 C GLY B 5 9.843 2.485 -14.585 1.00 1.00

ATOM 1081 O GLY B 5 10.415 2.617 -15.666 1.00 1.00

ATOM 1082 N ASP B 6 10.465 2.041 -13.492 1.00 1.00

ATOM 1083 CA ASP B 6 11.883 1.660 -13.534 1.00 1.00

ATOM 1084 C ASP B 6 12.122 0.193 -13.897 1.00 1.00

ATOM 1085 O ASP B 6 13.265 -0.283 -13.847 1.00 1.00

ATOM 1086 CB ASP B 6 12.615 2.031 -12.234 1.00 1.00

ATOM 1087 CG ASP B 6 12.147 1.228 -11.036 1.00 1.00

ATOM 1088 OD1 ASP B 6 11.243 0.378 -11.180 1.00 1.00

ATOM 1089 OD2 ASP B 6 12.680 1.460 -9.934 1.00 1.00

ATOM 1090 N GLY B 7 11.051 -0.515 -14.254 1.00 1.00

ATOM 1091 CA GLY B 7 11.161 -1.873 -14.788 1.00 1.00

ATOM 1092 C GLY B 7 11.065 -2.983 -13.759 1.00 1.00

ATOM 1093 O GLY B 7 11.096 -4.162 -14.112 1.00 1.00

ATOM 1094 N THR B 8 10.959 -2.608 -12.488 1.00 1.00

ATOM 1095 CA THR B 8 10.779 -3.579 -11.408 1.00 1.00

ATOM 1096 C THR B 8 9.661 -3.136 -10.468 1.00 1.00

ATOM 1097 O THR B 8 9.462 -1.938 -10.255 1.00 1.00

ATOM 1098 CB THR B 8 12.103 -3.854 -10.632 1.00 1.00

ATOM 1099 OG1 THR B 8 11.888 -4.876 -9.650 1.00 1.00

ATOM 1100 CG2 THR B 8 12.620 -2.597 -9.945 1.00 1.00

ATOM 1101 N ILE B 9 8.921 -4.104 -9.935 1.00 1.00

ATOM 1102 CA ILE B 9 7.832 -3.828 -8.999 1.00 1.00

ATOM 1103 C ILE B 9 8.304 -4.054 -7.566 1.00 1.00

ATOM 1104 O ILE B 9 8.647 -5.175 -7.186 1.00 1.00

ATOM 1105 CB ILE B 9 6.589 -4.707 -9.286 1.00 1.00

ATOM 1106 CG1 ILE B 9 6.167 -4.593 -10.756 1.00 1.00

ATOM 1107 CG2 ILE B 9 5.431 -4.328 -8.348 1.00 1.00

ATOM 1108 CD1 ILE B 9 5.316 -5.751 -11.242 1.00 1.00

ATOM 1109 N ASN B 10 8.328 -2.982 -6.780 1.00 1.00

ATOM 1110 CA ASN B 10 8.749 -3.050 -5.376 1.00 1.00

ATOM 1111 C ASN B 10 8.036 -2.037 -4.474 1.00 1.00

ATOM 1112 O ASN B 10 7.148 -1.302 -4.931 1.00 1.00

ATOM 1113 CB ASN B 10 10.286 -2.969 -5.234 1.00 1.00

ATOM 1114 CG ASN B 10 10.878 -1.679 -5.793 1.00 1.00

ATOM 1115 OD1 ASN B 10 10.275 -0.611 -5.714 1.00 1.00

ATOM 1116 ND2 ASN B 10 12.085 -1.778 -6.347 1.00 1.00

ATOM 1117 N SER B 11 8.431 -2.004 -3.202 1.00 1.00

ATOM 1118 CA SER B 11 7.792 -1.152 -2.197 1.00 1.00

ATOM 1119 C SER B 11 7.920 0.353 -2.455 1.00 1.00

ATOM 1120 O SER B 11 7.185 1.144 -1.865 1.00 1.00

ATOM 1121 CB SER B 11 8.285 -1.501 -0.783 1.00 1.00

ATOM 1122 OG SER B 11 9.696 -1.413 -0.680 1.00 1.00

ATOM 1123 N THR B 12 8.837 0.744 -3.336 1.00 1.00

ATOM 1124 CA THR B 12 9.004 2.161 -3.693 1.00 1.00

ATOM 1125 C THR B 12 7.833 2.614 -4.570 1.00 1.00

ATOM 1126 O THR B 12 7.345 3.743 -4.439 1.00 1.00

ATOM 1127 CB THR B 12 10.375 2.425 -4.361 1.00 1.00

ATOM 1128 OG1 THR B 12 11.409 1.871 -3.537 1.00 1.00

ATOM 1129 CG2 THR B 12 10.636 3.925 -4.541 1.00 1.00

ATOM 1130 N ASP B 13 7.370 1.714 -5.439 1.00 1.00

ATOM 1131 CA ASP B 13 6.128 1.914 -6.187 1.00 1.00

ATOM 1132 C ASP B 13 4.930 2.034 -5.250 1.00 1.00

ATOM 1133 O ASP B 13 4.041 2.851 -5.486 1.00 1.00

ATOM 1134 CB ASP B 13 5.890 0.766 -7.164 1.00 1.00

ATOM 1135 CG ASP B 13 6.989 0.643 -8.188 1.00 1.00

ATOM 1136 OD1 ASP B 13 7.227 1.618 -8.936 1.00 1.00

ATOM 1137 OD2 ASP B 13 7.620 -0.432 -8.248 1.00 1.00

ATOM 1138 N LEU B 14 4.918 1.214 -4.199 1.00 1.00

ATOM 1139 CA LEU B 14 3.864 1.276 -3.181 1.00 1.00

ATOM 1140 C LEU B 14 3.802 2.641 -2.486 1.00 1.00

ATOM 1141 O LEU B 14 2.722 3.219 -2.360 1.00 1.00

ATOM 1142 CB LEU B 14 3.998 0.138 -2.160 1.00 1.00

ATOM 1143 CG LEU B 14 2.877 -0.013 -1.123 1.00 1.00

ATOM 1144 CD1 LEU B 14 1.498 -0.157 -1.782 1.00 1.00

ATOM 1145 CD2 LEU B 14 3.155 -1.175 -0.201 1.00 1.00

ATOM 1146 N THR B 15 4.953 3.149 -2.042 1.00 1.00

ATOM 1147 CA THR B 15 5.024 4.487 -1.447 1.00 1.00

ATOM 1148 C THR B 15 4.490 5.546 -2.415 1.00 1.00

ATOM 1149 O THR B 15 3.681 6.399 -2.034 1.00 1.00

ATOM 1150 CB THR B 15 6.456 4.851 -1.006 1.00 1.00

ATOM 1151 OG1 THR B 15 6.832 4.023 0.094 1.00 1.00

ATOM 1152 CG2 THR B 15 6.539 6.319 -0.563 1.00 1.00

ATOM 1153 N MET B 16 4.938 5.469 -3.667 1.00 1.00

ATOM 1154 CA MET B 16 4.482 6.383 -4.710 1.00 1.00

ATOM 1155 C MET B 16 2.972 6.296 -4.912 1.00 1.00

ATOM 1156 O MET B 16 2.302 7.319 -5.023 1.00 1.00

ATOM 1157 CB MET B 16 5.212 6.107 -6.024 1.00 1.00

ATOM 1158 CG MET B 16 5.335 7.333 -6.892 1.00 1.00

ATOM 1159 SD MET B 16 6.143 7.024 -8.462 1.00 1.00

ATOM 1160 CE MET B 16 6.777 8.646 -8.818 1.00 1.00

ATOM 1161 N LEU B 17 2.448 5.073 -4.944 1.00 1.00

ATOM 1162 CA LEU B 17 1.015 4.843 -5.081 1.00 1.00

ATOM 1163 C LEU B 17 0.205 5.437 -3.922 1.00 1.00

ATOM 1164 O LEU B 17 -0.775 6.151 -4.149 1.00 1.00

ATOM 1165 CB LEU B 17 0.711 3.348 -5.246 1.00 1.00

ATOM 1166 CG LEU B 17 -0.739 2.931 -5.527 1.00 1.00

ATOM 1167 CD1 LEU B 17 -1.328 3.662 -6.729 1.00 1.00

ATOM 1168 CD2 LEU B 17 -0.827 1.422 -5.720 1.00 1.00

ATOM 1169 N LYS B 18 0.608 5.153 -2.684 1.00 1.00

ATOM 1170 CA LYS B 18 -0.158 5.659 -1.542 1.00 1.00

ATOM 1171 C LYS B 18 -0.076 7.180 -1.369 1.00 1.00

ATOM 1172 O LYS B 18 -1.079 7.815 -1.035 1.00 1.00

ATOM 1173 CB LYS B 18 0.136 4.892 -0.245 1.00 1.00

ATOM 1174 CG LYS B 18 1.582 4.716 0.136 1.00 1.00

ATOM 1175 CD LYS B 18 1.782 3.382 0.872 1.00 1.00

ATOM 1176 CE LYS B 18 1.004 3.298 2.182 1.00 1.00

ATOM 1177 NZ LYS B 18 1.048 1.908 2.756 1.00 1.00

ATOM 1178 N ARG B 19 1.093 7.756 -1.637 1.00 1.00

ATOM 1179 CA ARG B 19 1.242 9.217 -1.632 1.00 1.00

ATOM 1180 C ARG B 19 0.413 9.905 -2.725 1.00 1.00

ATOM 1181 O ARG B 19 -0.094 11.006 -2.519 1.00 1.00

ATOM 1182 CB ARG B 19 2.718 9.628 -1.690 1.00 1.00

ATOM 1183 CG ARG B 19 3.437 9.383 -0.367 1.00 1.00

ATOM 1184 CD ARG B 19 4.910 9.760 -0.384 1.00 1.00

ATOM 1185 NE ARG B 19 5.532 9.415 0.896 1.00 1.00

ATOM 1186 CZ ARG B 19 6.800 9.644 1.220 1.00 1.00

ATOM 1187 NH1 ARG B 19 7.626 10.227 0.363 1.00 1.00

ATOM 1188 NH2 ARG B 19 7.245 9.286 2.420 1.00 1.00

ATOM 1189 N SER B 20 0.250 9.252 -3.870 1.00 1.00

ATOM 1190 CA SER B 20 -0.612 9.790 -4.926 1.00 1.00

ATOM 1191 C SER B 20 -2.102 9.693 -4.559 1.00 1.00

ATOM 1192 O SER B 20 -2.888 10.582 -4.893 1.00 1.00

ATOM 1193 CB SER B 20 -0.337 9.109 -6.269 1.00 1.00

ATOM 1194 OG SER B 20 -0.987 7.852 -6.352 1.00 1.00

ATOM 1195 N VAL B 21 -2.483 8.613 -3.880 1.00 1.00

ATOM 1196 CA VAL B 21 -3.871 8.426 -3.439 1.00 1.00

ATOM 1197 C VAL B 21 -4.230 9.456 -2.359 1.00 1.00

ATOM 1198 O VAL B 21 -5.362 9.944 -2.308 1.00 1.00

ATOM 1199 CB VAL B 21 -4.150 6.955 -2.989 1.00 1.00

ATOM 1200 CG1 VAL B 21 -5.490 6.824 -2.258 1.00 1.00

ATOM 1201 CG2 VAL B 21 -4.128 6.025 -4.193 1.00 1.00

ATOM 1202 N LEU B 22 -3.254 9.801 -1.525 1.00 1.00

ATOM 1203 CA LEU B 22 -3.420 10.855 -0.521 1.00 1.00

ATOM 1204 C LEU B 22 -3.172 12.249 -1.101 1.00 1.00

ATOM 1205 O LEU B 22 -3.215 13.247 -0.373 1.00 1.00

ATOM 1206 CB LEU B 22 -2.494 10.605 0.673 1.00 1.00

ATOM 1207 CG LEU B 22 -2.841 9.421 1.584 1.00 1.00

ATOM 1208 CD1 LEU B 22 -1.607 8.922 2.327 1.00 1.00

ATOM 1209 CD2 LEU B 22 -3.962 9.768 2.562 1.00 1.00

ATOM 1210 N ARG B 23 -2.916 12.300 -2.412 1.00 1.00

ATOM 1211 CA ARG B 23 -2.594 13.534 -3.143 1.00 1.00

ATOM 1212 C ARG B 23 -1.361 14.284 -2.616 1.00 1.00

ATOM 1213 O ARG B 23 -1.247 15.499 -2.775 1.00 1.00

ATOM 1214 CB ARG B 23 -3.814 14.470 -3.242 1.00 1.00

ATOM 1215 CG ARG B 23 -5.018 13.841 -3.918 1.00 1.00

ATOM 1216 CD ARG B 23 -5.823 14.866 -4.702 1.00 1.00

ATOM 1217 NE ARG B 23 -7.061 14.290 -5.231 1.00 1.00

ATOM 1218 CZ ARG B 23 -7.158 13.590 -6.360 1.00 1.00

ATOM 1219 NH1 ARG B 23 -8.338 13.113 -6.736 1.00 1.00

ATOM 1220 NH2 ARG B 23 -6.088 13.359 -7.115 1.00 1.00

ATOM 1221 N ALA B 24 -0.440 13.543 -1.998 1.00 1.00

ATOM 1222 CA ALA B 24 0.842 14.098 -1.563 1.00 1.00

ATOM 1223 C ALA B 24 1.799 14.201 -2.750 1.00 1.00

ATOM 1224 O ALA B 24 2.749 14.984 -2.721 1.00 1.00

ATOM 1225 CB ALA B 24 1.445 13.256 -0.453 1.00 1.00

ATOM 1226 N ILE B 25 1.548 13.396 -3.782 1.00 1.00

ATOM 1227 CA ILE B 25 2.251 13.511 -5.059 1.00 1.00

ATOM 1228 C ILE B 25 1.275 13.408 -6.234 1.00 1.00

ATOM 1229 O ILE B 25 0.130 12.977 -6.078 1.00 1.00

ATOM 1230 CB ILE B 25 3.383 12.450 -5.240 1.00 1.00

ATOM 1231 CG1 ILE B 25 2.817 11.034 -5.304 1.00 1.00

ATOM 1232 CG2 ILE B 25 4.461 12.576 -4.158 1.00 1.00

ATOM 1233 CD1 ILE B 25 3.496 10.168 -6.335 1.00 1.00

ATOM 1234 N THR B 26 1.739 13.812 -7.412 1.00 1.00

ATOM 1235 CA THR B 26 0.969 13.655 -8.642 1.00 1.00

ATOM 1236 C THR B 26 1.689 12.705 -9.601 1.00 1.00

ATOM 1237 O THR B 26 2.912 12.778 -9.761 1.00 1.00

ATOM 1238 CB THR B 26 0.636 15.023 -9.307 1.00 1.00

ATOM 1239 OG1 THR B 26 0.357 14.836 -10.702 1.00 1.00

ATOM 1240 CG2 THR B 26 1.784 16.013 -9.150 1.00 1.00

ATOM 1241 N LEU B 27 0.925 11.803 -10.212 1.00 1.00

ATOM 1242 CA LEU B 27 1.476 10.817 -11.135 1.00 1.00

ATOM 1243 C LEU B 27 1.465 11.350 -12.563 1.00 1.00

ATOM 1244 O LEU B 27 0.580 12.125 -12.942 1.00 1.00

ATOM 1245 CB LEU B 27 0.694 9.495 -11.066 1.00 1.00

ATOM 1246 CG LEU B 27 0.630 8.697 -9.754 1.00 1.00

ATOM 1247 CD1 LEU B 27 -0.330 7.517 -9.888 1.00 1.00

ATOM 1248 CD2 LEU B 27 1.998 8.211 -9.291 1.00 1.00

ATOM 1249 N THR B 28 2.455 10.937 -13.351 1.00 1.00

ATOM 1250 CA THR B 28 2.443 11.186 -14.787 1.00 1.00

ATOM 1251 C THR B 28 1.417 10.251 -15.405 1.00 1.00

ATOM 1252 O THR B 28 1.066 9.229 -14.810 1.00 1.00

ATOM 1253 CB THR B 28 3.828 10.953 -15.452 1.00 1.00

ATOM 1254 OG1 THR B 28 4.097 9.545 -15.540 1.00 1.00

ATOM 1255 CG2 THR B 28 4.945 11.654 -14.675 1.00 1.00

ATOM 1256 N ASP B 29 0.945 10.589 -16.598 1.00 1.00

ATOM 1257 CA ASP B 29 -0.077 9.793 -17.266 1.00 1.00

ATOM 1258 C ASP B 29 0.356 8.343 -17.521 1.00 1.00

ATOM 1259 O ASP B 29 -0.454 7.423 -17.370 1.00 1.00

ATOM 1260 CB ASP B 29 -0.540 10.478 -18.553 1.00 1.00

ATOM 1261 CG ASP B 29 -1.295 11.778 -18.288 1.00 1.00

ATOM 1262 OD1 ASP B 29 -1.912 11.920 -17.210 1.00 1.00

ATOM 1263 OD2 ASP B 29 -1.271 12.664 -19.168 1.00 1.00

ATOM 1264 N ASP B 30 1.623 8.139 -17.895 1.00 1.00

ATOM 1265 CA ASP B 30 2.154 6.781 -18.073 1.00 1.00

ATOM 1266 C ASP B 30 2.124 5.994 -16.757 1.00 1.00

ATOM 1267 O ASP B 30 1.841 4.799 -16.752 1.00 1.00

ATOM 1268 CB ASP B 30 3.590 6.803 -18.615 1.00 1.00

ATOM 1269 CG ASP B 30 3.677 7.256 -20.067 1.00 1.00

ATOM 1270 OD1 ASP B 30 2.642 7.482 -20.722 1.00 1.00

ATOM 1271 OD2 ASP B 30 4.815 7.385 -20.555 1.00 1.00

ATOM 1272 N ALA B 31 2.429 6.678 -15.655 1.00 1.00

ATOM 1273 CA ALA B 31 2.429 6.068 -14.324 1.00 1.00

ATOM 1274 C ALA B 31 1.026 5.678 -13.862 1.00 1.00

ATOM 1275 O ALA B 31 0.862 4.683 -13.156 1.00 1.00

ATOM 1276 CB ALA B 31 3.081 6.996 -13.312 1.00 1.00

ATOM 1277 N LYS B 32 0.028 6.471 -14.254 1.00 1.00

ATOM 1278 CA LYS B 32 -1.371 6.202 -13.902 1.00 1.00

ATOM 1279 C LYS B 32 -1.843 4.856 -14.457 1.00 1.00

ATOM 1280 O LYS B 32 -2.548 4.111 -13.771 1.00 1.00

ATOM 1281 CB LYS B 32 -2.289 7.331 -14.390 1.00 1.00

ATOM 1282 CG LYS B 32 -2.096 8.660 -13.674 1.00 1.00

ATOM 1283 CD LYS B 32 -2.977 9.741 -14.291 1.00 1.00

ATOM 1284 CE LYS B 32 -2.566 11.133 -13.820 1.00 1.00

ATOM 1285 NZ LYS B 32 -3.381 12.210 -14.463 1.00 1.00

ATOM 1286 N ALA B 33 -1.441 4.547 -15.688 1.00 1.00

ATOM 1287 CA ALA B 33 -1.758 3.256 -16.299 1.00 1.00

ATOM 1288 C ALA B 33 -1.097 2.094 -15.549 1.00 1.00

ATOM 1289 O ALA B 33 -1.688 1.024 -15.401 1.00 1.00

ATOM 1290 CB ALA B 33 -1.362 3.248 -17.770 1.00 1.00

ATOM 1291 N ARG B 34 0.124 2.312 -15.069 1.00 1.00

ATOM 1292 CA ARG B 34 0.839 1.289 -14.306 1.00 1.00

ATOM 1293 C ARG B 34 0.290 1.154 -12.877 1.00 1.00

ATOM 1294 O ARG B 34 0.346 0.075 -12.286 1.00 1.00

ATOM 1295 CB ARG B 34 2.350 1.573 -14.304 1.00 1.00

ATOM 1296 CG ARG B 34 2.941 1.727 -15.706 1.00 1.00

ATOM 1297 CD ARG B 34 4.460 1.839 -15.706 1.00 1.00

ATOM 1298 NE ARG B 34 4.927 3.055 -15.041 1.00 1.00

ATOM 1299 CZ ARG B 34 5.313 4.161 -15.669 1.00 1.00

ATOM 1300 NH1 ARG B 34 5.303 4.221 -16.999 1.00 1.00

ATOM 1301 NH2 ARG B 34 5.719 5.209 -14.967 1.00 1.00

ATOM 1302 N ALA B 35 -0.258 2.253 -12.352 1.00 1.00

ATOM 1303 CA ALA B 35 -0.780 2.327 -10.976 1.00 1.00

ATOM 1304 C ALA B 35 -2.135 1.643 -10.785 1.00 1.00

ATOM 1305 O ALA B 35 -2.430 1.126 -9.702 1.00 1.00

ATOM 1306 CB ALA B 35 -0.862 3.777 -10.521 1.00 1.00

ATOM 1307 N ASP B 36 -2.961 1.656 -11.831 1.00 1.00

ATOM 1308 CA ASP B 36 -4.286 1.040 -11.788 1.00 1.00

ATOM 1309 C ASP B 36 -4.151 -0.476 -11.889 1.00 1.00

ATOM 1310 O ASP B 36 -4.424 -1.074 -12.932 1.00 1.00

ATOM 1311 CB ASP B 36 -5.171 1.589 -12.912 1.00 1.00

ATOM 1312 CG ASP B 36 -6.624 1.146 -12.790 1.00 1.00

ATOM 1313 OD1 ASP B 36 -6.987 0.510 -11.773 1.00 1.00

ATOM 1314 OD2 ASP B 36 -7.408 1.437 -13.715 1.00 1.00

ATOM 1315 N VAL B 37 -3.738 -1.087 -10.780 1.00 1.00

ATOM 1316 CA VAL B 37 -3.351 -2.498 -10.760 1.00 1.00

ATOM 1317 C VAL B 37 -4.508 -3.486 -10.940 1.00 1.00

ATOM 1318 O VAL B 37 -4.292 -4.599 -11.424 1.00 1.00

ATOM 1319 CB VAL B 37 -2.513 -2.851 -9.504 1.00 1.00

ATOM 1320 CG1 VAL B 37 -1.142 -2.196 -9.587 1.00 1.00

ATOM 1321 CG2 VAL B 37 -3.227 -2.431 -8.225 1.00 1.00

ATOM 1322 N ASP B 38 -5.723 -3.082 -10.559 1.00 1.00

ATOM 1323 CA ASP B 38 -6.911 -3.918 -10.774 1.00 1.00

ATOM 1324 C ASP B 38 -7.675 -3.551 -12.056 1.00 1.00

ATOM 1325 O ASP B 38 -8.692 -4.174 -12.385 1.00 1.00

ATOM 1326 CB ASP B 38 -7.831 -3.944 -9.539 1.00 1.00

ATOM 1327 CG ASP B 38 -8.338 -2.571 -9.140 1.00 1.00

ATOM 1328 OD1 ASP B 38 -8.297 -1.632 -9.962 1.00 1.00

ATOM 1329 OD2 ASP B 38 -8.788 -2.431 -7.982 1.00 1.00

ATOM 1330 N LYS B 39 -7.166 -2.531 -12.751 1.00 1.00

ATOM 1331 CA LYS B 39 -7.622 -2.118 -14.090 1.00 1.00

ATOM 1332 C LYS B 39 -9.109 -1.759 -14.162 1.00 1.00

ATOM 1333 O LYS B 39 -9.781 -2.056 -15.152 1.00 1.00

ATOM 1334 CB LYS B 39 -7.267 -3.181 -15.140 1.00 1.00

ATOM 1335 CG LYS B 39 -5.845 -3.733 -15.013 1.00 1.00

ATOM 1336 CD LYS B 39 -5.585 -4.862 -15.994 1.00 1.00

ATOM 1337 CE LYS B 39 -5.019 -4.338 -17.305 1.00 1.00

ATOM 1338 NZ LYS B 39 -4.419 -5.435 -18.120 1.00 1.00

ATOM 1339 N ASN B 40 -9.615 -1.107 -13.118 1.00 1.00

ATOM 1340 CA ASN B 40 -11.026 -0.713 -13.081 1.00 1.00

ATOM 1341 C ASN B 40 -11.280 0.740 -13.506 1.00 1.00

ATOM 1342 O ASN B 40 -12.429 1.189 -13.540 1.00 1.00

ATOM 1343 CB ASN B 40 -11.636 -1.009 -11.704 1.00 1.00

ATOM 1344 CG ASN B 40 -11.018 -0.182 -10.591 1.00 1.00

ATOM 1345 OD1 ASN B 40 -10.113 0.629 -10.815 1.00 1.00

ATOM 1346 ND2 ASN B 40 -11.506 -0.386 -9.374 1.00 1.00

ATOM 1347 N GLY B 41 -10.206 1.463 -13.821 1.00 1.00

ATOM 1348 CA GLY B 41 -10.307 2.828 -14.335 1.00 1.00

ATOM 1349 C GLY B 41 -10.059 3.922 -13.314 1.00 1.00

ATOM 1350 O GLY B 41 -10.080 5.106 -13.654 1.00 1.00

ATOM 1351 N SER B 42 -9.820 3.523 -12.066 1.00 1.00

ATOM 1352 CA SER B 42 -9.590 4.457 -10.967 1.00 1.00

ATOM 1353 C SER B 42 -8.337 4.099 -10.170 1.00 1.00

ATOM 1354 O SER B 42 -7.957 2.930 -10.086 1.00 1.00

ATOM 1355 CB SER B 42 -10.803 4.481 -10.028 1.00 1.00

ATOM 1356 OG SER B 42 -11.950 5.001 -10.684 1.00 1.00

ATOM 1357 N ILE B 43 -7.700 5.109 -9.583 1.00 1.00

ATOM 1358 CA ILE B 43 -6.587 4.892 -8.666 1.00 1.00

ATOM 1359 C ILE B 43 -7.060 5.190 -7.250 1.00 1.00

ATOM 1360 O ILE B 43 -7.402 6.329 -6.938 1.00 1.00

ATOM 1361 CB ILE B 43 -5.366 5.797 -8.985 1.00 1.00

ATOM 1362 CG1 ILE B 43 -5.054 5.823 -10.490 1.00 1.00

ATOM 1363 CG2 ILE B 43 -4.162 5.412 -8.121 1.00 1.00

ATOM 1364 CD1 ILE B 43 -4.593 4.521 -11.070 1.00 1.00

ATOM 1365 N ASN B 44 -7.071 4.169 -6.400 1.00 1.00

ATOM 1366 CA ASN B 44 -7.588 4.315 -5.039 1.00 1.00

ATOM 1367 C ASN B 44 -6.936 3.379 -4.025 1.00 1.00

ATOM 1368 O ASN B 44 -5.961 2.691 -4.339 1.00 1.00

ATOM 1369 CB ASN B 44 -9.119 4.157 -5.031 1.00 1.00

ATOM 1370 CG ASN B 44 -9.584 2.847 -5.638 1.00 1.00

ATOM 1371 OD1 ASN B 44 -8.871 1.840 -5.618 1.00 1.00

ATOM 1372 ND2 ASN B 44 -10.800 2.850 -6.177 1.00 1.00

ATOM 1373 N SER B 45 -7.489 3.358 -2.812 1.00 1.00

ATOM 1374 CA SER B 45 -7.005 2.499 -1.724 1.00 1.00

ATOM 1375 C SER B 45 -6.999 1.011 -2.094 1.00 1.00

ATOM 1376 O SER B 45 -6.136 0.259 -1.641 1.00 1.00

ATOM 1377 CB SER B 45 -7.830 2.732 -0.463 1.00 1.00

ATOM 1378 OG SER B 45 -9.195 2.406 -0.673 1.00 1.00

ATOM 1379 N THR B 46 -7.960 0.593 -2.917 1.00 1.00

ATOM 1380 CA THR B 46 -8.029 -0.795 -3.383 1.00 1.00

ATOM 1381 C THR B 46 -6.802 -1.188 -4.218 1.00 1.00

ATOM 1382 O THR B 46 -6.340 -2.328 -4.147 1.00 1.00

ATOM 1383 CB THR B 46 -9.302 -1.034 -4.215 1.00 1.00

ATOM 1384 OG1 THR B 46 -10.447 -0.572 -3.486 1.00 1.00

ATOM 1385 CG2 THR B 46 -9.474 -2.515 -4.514 1.00 1.00

ATOM 1386 N ASP B 47 -6.278 -0.242 -4.998 1.00 1.00

ATOM 1387 CA ASP B 47 -5.022 -0.458 -5.730 1.00 1.00

ATOM 1388 C ASP B 47 -3.826 -0.606 -4.793 1.00 1.00

ATOM 1389 O ASP B 47 -2.953 -1.454 -5.016 1.00 1.00

ATOM 1390 CB ASP B 47 -4.767 0.678 -6.723 1.00 1.00

ATOM 1391 CG ASP B 47 -5.769 0.695 -7.852 1.00 1.00

ATOM 1392 OD1 ASP B 47 -5.836 -0.286 -8.624 1.00 1.00

ATOM 1393 OD2 ASP B 47 -6.502 1.692 -7.977 1.00 1.00

ATOM 1394 N VAL B 48 -3.797 0.223 -3.751 1.00 1.00

ATOM 1395 CA VAL B 48 -2.746 0.173 -2.733 1.00 1.00

ATOM 1396 C VAL B 48 -2.701 -1.215 -2.092 1.00 1.00

ATOM 1397 O VAL B 48 -1.631 -1.819 -1.972 1.00 1.00

ATOM 1398 CB VAL B 48 -2.951 1.269 -1.655 1.00 1.00

ATOM 1399 CG1 VAL B 48 -2.025 1.049 -0.461 1.00 1.00

ATOM 1400 CG2 VAL B 48 -2.737 2.661 -2.253 1.00 1.00

ATOM 1401 N LEU B 49 -3.872 -1.718 -1.713 1.00 1.00

ATOM 1402 CA LEU B 49 -3.992 -3.008 -1.046 1.00 1.00

ATOM 1403 C LEU B 49 -3.641 -4.185 -1.958 1.00 1.00

ATOM 1404 O LEU B 49 -3.024 -5.155 -1.513 1.00 1.00

ATOM 1405 CB LEU B 49 -5.388 -3.171 -0.438 1.00 1.00

ATOM 1406 CG LEU B 49 -5.723 -4.608 0.000 1.00 1.00

ATOM 1407 CD1 LEU B 49 -5.223 -4.873 1.401 1.00 1.00

ATOM 1408 CD2 LEU B 49 -7.219 -4.907 -0.106 1.00 1.00

ATOM 1409 N LEU B 50 -4.021 -4.084 -3.233 1.00 1.00

ATOM 1410 CA LEU B 50 -3.675 -5.107 -4.214 1.00 1.00

ATOM 1411 C LEU B 50 -2.163 -5.170 -4.448 1.00 1.00

ATOM 1412 O LEU B 50 -1.577 -6.257 -4.486 1.00 1.00

ATOM 1413 CB LEU B 50 -4.426 -4.895 -5.531 1.00 1.00

ATOM 1414 CG LEU B 50 -4.221 -5.971 -6.611 1.00 1.00

ATOM 1415 CD1 LEU B 50 -4.634 -7.344 -6.092 1.00 1.00

ATOM 1416 CD2 LEU B 50 -4.996 -5.634 -7.862 1.00 1.00

ATOM 1417 N LEU B 51 -1.534 -4.006 -4.594 1.00 1.00

ATOM 1418 CA LEU B 51 -0.081 -3.954 -4.758 1.00 1.00

ATOM 1419 C LEU B 51 0.646 -4.505 -3.528 1.00 1.00

ATOM 1420 O LEU B 51 1.656 -5.199 -3.663 1.00 1.00

ATOM 1421 CB LEU B 51 0.395 -2.532 -5.104 1.00 1.00

ATOM 1422 CG LEU B 51 1.902 -2.347 -5.365 1.00 1.00

ATOM 1423 CD1 LEU B 51 2.426 -3.307 -6.440 1.00 1.00

ATOM 1424 CD2 LEU B 51 2.241 -0.911 -5.720 1.00 1.00

ATOM 1425 N SER B 52 0.122 -4.202 -2.338 1.00 1.00

ATOM 1426 CA SER B 52 0.680 -4.721 -1.082 1.00 1.00

ATOM 1427 C SER B 52 0.622 -6.246 -1.009 1.00 1.00

ATOM 1428 O SER B 52 1.579 -6.887 -0.575 1.00 1.00

ATOM 1429 CB SER B 52 -0.037 -4.118 0.124 1.00 1.00

ATOM 1430 OG SER B 52 0.170 -2.724 0.182 1.00 1.00

ATOM 1431 N ARG B 53 -0.509 -6.815 -1.425 1.00 1.00

ATOM 1432 CA ARG B 53 -0.688 -8.264 -1.456 1.00 1.00

ATOM 1433 C ARG B 53 0.297 -8.924 -2.416 1.00 1.00

ATOM 1434 O ARG B 53 0.827 -9.997 -2.124 1.00 1.00

ATOM 1435 CB ARG B 53 -2.126 -8.632 -1.843 1.00 1.00

ATOM 1436 CG ARG B 53 -3.160 -8.458 -0.730 1.00 1.00

ATOM 1437 CD ARG B 53 -4.379 -9.357 -0.966 1.00 1.00

ATOM 1438 NE ARG B 53 -4.946 -9.170 -2.301 1.00 1.00

ATOM 1439 CZ ARG B 53 -5.433 -10.155 -3.060 1.00 1.00

ATOM 1440 NH1 ARG B 53 -5.420 -11.405 -2.627 1.00 1.00

ATOM 1441 NH2 ARG B 53 -5.923 -9.887 -4.262 1.00 1.00

ATOM 1442 N TYR B 54 0.537 -8.271 -3.553 1.00 1.00

ATOM 1443 CA TYR B 54 1.503 -8.747 -4.551 1.00 1.00

ATOM 1444 C TYR B 54 2.920 -8.751 -3.985 1.00 1.00

ATOM 1445 O TYR B 54 3.663 -9.720 -4.161 1.00 1.00

ATOM 1446 CB TYR B 54 1.424 -7.909 -5.837 1.00 1.00

ATOM 1447 CG TYR B 54 2.473 -8.262 -6.880 1.00 1.00

ATOM 1448 CD1 TYR B 54 2.256 -9.287 -7.801 1.00 1.00

ATOM 1449 CD2 TYR B 54 3.689 -7.572 -6.937 1.00 1.00

ATOM 1450 CE1 TYR B 54 3.220 -9.616 -8.755 1.00 1.00

ATOM 1451 CE2 TYR B 54 4.658 -7.894 -7.885 1.00 1.00

ATOM 1452 CZ TYR B 54 4.416 -8.915 -8.788 1.00 1.00

ATOM 1453 OH TYR B 54 5.374 -9.234 -9.725 1.00 1.00

ATOM 1454 N LEU B 55 3.289 -7.668 -3.301 1.00 1.00

ATOM 1455 CA LEU B 55 4.623 -7.546 -2.703 1.00 1.00

ATOM 1456 C LEU B 55 4.828 -8.527 -1.551 1.00 1.00

ATOM 1457 O LEU B 55 5.945 -8.995 -1.319 1.00 1.00

ATOM 1458 CB LEU B 55 4.901 -6.103 -2.264 1.00 1.00

ATOM 1459 CG LEU B 55 4.904 -5.062 -3.395 1.00 1.00

ATOM 1460 CD1 LEU B 55 4.947 -3.650 -2.845 1.00 1.00

ATOM 1461 CD2 LEU B 55 6.047 -5.293 -4.382 1.00 1.00

ATOM 1462 N LEU B 56 3.749 -8.851 -0.844 1.00 1.00

ATOM 1463 CA LEU B 56 3.790 -9.867 0.212 1.00 1.00

ATOM 1464 C LEU B 56 3.709 -11.288 -0.348 1.00 1.00

ATOM 1465 O LEU B 56 3.816 -12.266 0.402 1.00 1.00

ATOM 1466 CB LEU B 56 2.699 -9.617 1.254 1.00 1.00

ATOM 1467 CG LEU B 56 3.001 -8.440 2.187 1.00 1.00

ATOM 1468 CD1 LEU B 56 1.724 -7.906 2.813 1.00 1.00

ATOM 1469 CD2 LEU B 56 4.016 -8.819 3.253 1.00 1.00

ATOM 1470 OXT LEU B 56 3.562 -11.419 -1.587 1.00 1.00

ATOM 1471 CA CA C 160 9.159 0.333 -9.973 1.00 1.00

ATOM 1472 CA CA D 161 -7.994 0.737 -9.667 1.00 1.00

TER

ENDMDL

MODEL 4

ATOM 0 N GLY A 5 -25.852 0.602 2.639 1.00 1.00

ATOM 1 CA GLY A 5 -24.451 0.107 2.790 1.00 1.00

ATOM 2 C GLY A 5 -23.739 0.723 3.982 1.00 1.00

ATOM 3 O GLY A 5 -24.291 0.780 5.087 1.00 1.00

ATOM 4 N VAL A 6 -22.509 1.183 3.762 1.00 1.00

ATOM 5 CA VAL A 6 -21.710 1.797 4.825 1.00 1.00

ATOM 6 C VAL A 6 -22.110 3.262 5.034 1.00 1.00

ATOM 7 O VAL A 6 -22.338 3.994 4.072 1.00 1.00

ATOM 8 CB VAL A 6 -20.180 1.692 4.547 1.00 1.00

ATOM 9 CG1 VAL A 6 -19.371 2.339 5.668 1.00 1.00

ATOM 10 CG2 VAL A 6 -19.759 0.237 4.365 1.00 1.00

ATOM 11 N VAL A 7 -22.207 3.674 6.297 1.00 1.00

ATOM 12 CA VAL A 7 -22.502 5.062 6.636 1.00 1.00

ATOM 13 C VAL A 7 -21.323 5.650 7.406 1.00 1.00

ATOM 14 O VAL A 7 -20.906 5.114 8.430 1.00 1.00

ATOM 15 CB VAL A 7 -23.820 5.205 7.453 1.00 1.00

ATOM 16 CG1 VAL A 7 -24.054 6.664 7.860 1.00 1.00

ATOM 17 CG2 VAL A 7 -25.017 4.688 6.653 1.00 1.00

ATOM 18 N VAL A 8 -20.778 6.747 6.892 1.00 1.00

ATOM 19 CA VAL A 8 -19.680 7.436 7.559 1.00 1.00

ATOM 20 C VAL A 8 -20.211 8.748 8.139 1.00 1.00

ATOM 21 O VAL A 8 -20.645 9.633 7.395 1.00 1.00

ATOM 22 CB VAL A 8 -18.502 7.705 6.593 1.00 1.00

ATOM 23 CG1 VAL A 8 -17.332 8.347 7.328 1.00 1.00

ATOM 24 CG2 VAL A 8 -18.062 6.416 5.891 1.00 1.00

ATOM 25 N GLU A 9 -20.183 8.862 9.465 1.00 1.00

ATOM 26 CA GLU A 9 -20.681 10.061 10.127 1.00 1.00

ATOM 27 C GLU A 9 -19.566 10.845 10.807 1.00 1.00

ATOM 28 O GLU A 9 -18.832 10.305 11.637 1.00 1.00

ATOM 29 CB GLU A 9 -21.795 9.731 11.128 1.00 1.00

ATOM 30 CG GLU A 9 -22.374 10.973 11.812 1.00 1.00

ATOM 31 CD GLU A 9 -23.679 10.722 12.555 1.00 1.00

ATOM 32 OE1 GLU A 9 -24.263 9.619 12.432 1.00 1.00

ATOM 33 OE2 GLU A 9 -24.125 11.648 13.266 1.00 1.00

ATOM 34 N ILE A 10 -19.448 12.118 10.430 1.00 1.00

ATOM 35 CA ILE A 10 -18.557 13.059 11.101 1.00 1.00

ATOM 36 C ILE A 10 -19.280 13.611 12.331 1.00 1.00

ATOM 37 O ILE A 10 -20.375 14.163 12.220 1.00 1.00

ATOM 38 CB ILE A 10 -18.131 14.217 10.151 1.00 1.00

ATOM 39 CG1 ILE A 10 -17.372 13.661 8.938 1.00 1.00

ATOM 40 CG2 ILE A 10 -17.282 15.264 10.896 1.00 1.00

ATOM 41 CD1 ILE A 10 -17.525 14.487 7.668 1.00 1.00

ATOM 42 N GLY A 11 -18.665 13.438 13.498 1.00 1.00

ATOM 43 CA GLY A 11 -19.239 13.905 14.765 1.00 1.00

ATOM 44 C GLY A 11 -19.423 15.414 14.823 1.00 1.00

ATOM 45 O GLY A 11 -18.806 16.158 14.053 1.00 1.00

ATOM 46 N LYS A 12 -20.287 15.859 15.729 1.00 1.00

ATOM 47 CA LYS A 12 -20.515 17.283 15.951 1.00 1.00

ATOM 48 C LYS A 12 -20.124 17.627 17.379 1.00 1.00

ATOM 49 O LYS A 12 -20.629 17.028 18.328 1.00 1.00

ATOM 50 CB LYS A 12 -21.972 17.665 15.679 1.00 1.00

ATOM 51 CG LYS A 12 -22.398 17.517 14.222 1.00 1.00

ATOM 52 CD LYS A 12 -23.900 17.714 14.077 1.00 1.00

ATOM 53 CE LYS A 12 -24.371 17.459 12.652 1.00 1.00

ATOM 54 NZ LYS A 12 -24.175 16.040 12.233 1.00 1.00

ATOM 55 N VAL A 13 -19.197 18.573 17.521 1.00 1.00

ATOM 56 CA VAL A 13 -18.660 18.944 18.830 1.00 1.00

ATOM 57 C VAL A 13 -18.688 20.459 19.028 1.00 1.00

ATOM 58 O VAL A 13 -18.928 21.216 18.085 1.00 1.00

ATOM 59 CB VAL A 13 -17.206 18.413 19.052 1.00 1.00

ATOM 60 CG1 VAL A 13 -17.164 16.886 19.002 1.00 1.00

ATOM 61 CG2 VAL A 13 -16.240 19.016 18.037 1.00 1.00

ATOM 62 N THR A 14 -18.454 20.886 20.263 1.00 1.00

ATOM 63 CA THR A 14 -18.413 22.299 20.604 1.00 1.00

ATOM 64 C THR A 14 -17.088 22.604 21.300 1.00 1.00

ATOM 65 O THR A 14 -16.442 21.700 21.838 1.00 1.00

ATOM 66 CB THR A 14 -19.595 22.709 21.522 1.00 1.00

ATOM 67 OG1 THR A 14 -19.572 21.918 22.715 1.00 1.00

ATOM 68 CG2 THR A 14 -20.933 22.510 20.814 1.00 1.00

ATOM 69 N GLY A 15 -16.687 23.869 21.282 1.00 1.00

ATOM 70 CA GLY A 15 -15.458 24.294 21.947 1.00 1.00

ATOM 71 C GLY A 15 -15.190 25.774 21.783 1.00 1.00

ATOM 72 O GLY A 15 -15.469 26.354 20.730 1.00 1.00

ATOM 73 N SER A 16 -14.649 26.382 22.837 1.00 1.00

ATOM 74 CA SER A 16 -14.279 27.794 22.822 1.00 1.00

ATOM 75 C SER A 16 -13.024 28.039 21.986 1.00 1.00

ATOM 76 O SER A 16 -12.210 27.134 21.788 1.00 1.00

ATOM 77 CB SER A 16 -14.056 28.297 24.253 1.00 1.00

ATOM 78 OG SER A 16 -15.227 28.129 25.033 1.00 1.00

ATOM 79 N VAL A 17 -12.886 29.271 21.492 1.00 1.00

ATOM 80 CA VAL A 17 -11.693 29.701 20.764 1.00 1.00

ATOM 81 C VAL A 17 -10.432 29.445 21.591 1.00 1.00

ATOM 82 O VAL A 17 -10.373 29.786 22.777 1.00 1.00

ATOM 83 CB VAL A 17 -11.780 31.205 20.371 1.00 1.00

ATOM 84 CG1 VAL A 17 -10.460 31.712 19.793 1.00 1.00

ATOM 85 CG2 VAL A 17 -12.918 31.435 19.384 1.00 1.00

ATOM 86 N GLY A 18 -9.435 28.829 20.961 1.00 1.00

ATOM 87 CA GLY A 18 -8.157 28.551 21.612 1.00 1.00

ATOM 88 C GLY A 18 -8.031 27.170 22.223 1.00 1.00

ATOM 89 O GLY A 18 -6.936 26.771 22.621 1.00 1.00

ATOM 90 N THR A 19 -9.143 26.441 22.308 1.00 1.00

ATOM 91 CA THR A 19 -9.137 25.084 22.864 1.00 1.00

ATOM 92 C THR A 19 -8.964 24.024 21.773 1.00 1.00

ATOM 93 O THR A 19 -9.195 24.288 20.590 1.00 1.00

ATOM 94 CB THR A 19 -10.419 24.762 23.682 1.00 1.00

ATOM 95 OG1 THR A 19 -11.545 24.653 22.803 1.00 1.00

ATOM 96 CG2 THR A 19 -10.688 25.826 24.737 1.00 1.00

ATOM 97 N THR A 20 -8.552 22.829 22.188 1.00 1.00

ATOM 98 CA THR A 20 -8.439 21.692 21.284 1.00 1.00

ATOM 99 C THR A 20 -9.671 20.806 21.440 1.00 1.00

ATOM 100 O THR A 20 -10.119 20.538 22.558 1.00 1.00

ATOM 101 CB THR A 20 -7.155 20.868 21.552 1.00 1.00

ATOM 102 OG1 THR A 20 -6.028 21.747 21.657 1.00 1.00

ATOM 103 CG2 THR A 20 -6.900 19.888 20.421 1.00 1.00

ATOM 104 N VAL A 21 -10.220 20.377 20.307 1.00 1.00

ATOM 105 CA VAL A 21 -11.368 19.470 20.281 1.00 1.00

ATOM 106 C VAL A 21 -11.039 18.205 19.490 1.00 1.00

ATOM 107 O VAL A 21 -10.125 18.207 18.662 1.00 1.00

ATOM 108 CB VAL A 21 -12.647 20.149 19.688 1.00 1.00

ATOM 109 CG1 VAL A 21 -13.181 21.228 20.625 1.00 1.00

ATOM 110 CG2 VAL A 21 -12.377 20.723 18.297 1.00 1.00

ATOM 111 N GLU A 22 -11.775 17.129 19.764 1.00 1.00

ATOM 112 CA GLU A 22 -11.669 15.892 18.997 1.00 1.00

ATOM 113 C GLU A 22 -12.958 15.610 18.233 1.00 1.00

ATOM 114 O GLU A 22 -14.026 15.480 18.834 1.00 1.00

ATOM 115 CB GLU A 22 -11.328 14.697 19.895 1.00 1.00

ATOM 116 CG GLU A 22 -9.859 14.566 20.219 1.00 1.00

ATOM 117 CD GLU A 22 -9.403 13.118 20.381 1.00 1.00

ATOM 118 OE1 GLU A 22 -10.206 12.263 20.816 1.00 1.00

ATOM 119 OE2 GLU A 22 -8.224 12.842 20.075 1.00 1.00

ATOM 120 N ILE A 23 -12.842 15.518 16.912 1.00 1.00

ATOM 121 CA ILE A 23 -13.987 15.227 16.051 1.00 1.00

ATOM 122 C ILE A 23 -13.899 13.777 15.570 1.00 1.00

ATOM 123 O ILE A 23 -13.076 13.457 14.712 1.00 1.00

ATOM 124 CB ILE A 23 -14.085 16.186 14.828 1.00 1.00

ATOM 125 CG1 ILE A 23 -13.954 17.658 15.247 1.00 1.00

ATOM 126 CG2 ILE A 23 -15.390 15.958 14.068 1.00 1.00

ATOM 127 CD1 ILE A 23 -12.546 18.217 15.119 1.00 1.00

ATOM 128 N PRO A 24 -14.742 12.894 16.134 1.00 1.00

ATOM 129 CA PRO A 24 -14.700 11.486 15.737 1.00 1.00

ATOM 130 C PRO A 24 -15.362 11.231 14.386 1.00 1.00

ATOM 131 O PRO A 24 -16.301 11.941 14.003 1.00 1.00

ATOM 132 CB PRO A 24 -15.472 10.777 16.853 1.00 1.00

ATOM 133 CG PRO A 24 -16.382 11.800 17.393 1.00 1.00

ATOM 134 CD PRO A 24 -15.767 13.150 17.163 1.00 1.00

ATOM 135 N VAL A 25 -14.857 10.231 13.671 1.00 1.00

ATOM 136 CA VAL A 25 -15.447 9.799 12.413 1.00 1.00

ATOM 137 C VAL A 25 -15.990 8.392 12.635 1.00 1.00

ATOM 138 O VAL A 25 -15.219 7.452 12.855 1.00 1.00

ATOM 139 CB VAL A 25 -14.418 9.826 11.249 1.00 1.00

ATOM 140 CG1 VAL A 25 -15.070 9.399 9.942 1.00 1.00

ATOM 141 CG2 VAL A 25 -13.794 11.217 11.101 1.00 1.00

ATOM 142 N TYR A 26 -17.319 8.265 12.603 1.00 1.00

ATOM 143 CA TYR A 26 -17.997 7.005 12.904 1.00 1.00

ATOM 144 C TYR A 26 -18.279 6.204 11.649 1.00 1.00

ATOM 145 O TYR A 26 -18.656 6.763 10.618 1.00 1.00

ATOM 146 CB TYR A 26 -19.331 7.247 13.623 1.00 1.00

ATOM 147 CG TYR A 26 -19.241 7.980 14.942 1.00 1.00

ATOM 148 CD1 TYR A 26 -19.472 9.352 15.007 1.00 1.00

ATOM 149 CD2 TYR A 26 -18.947 7.303 16.123 1.00 1.00

ATOM 150 CE1 TYR A 26 -19.403 10.037 16.214 1.00 1.00

ATOM 151 CE2 TYR A 26 -18.874 7.977 17.338 1.00 1.00

ATOM 152 CZ TYR A 26 -19.105 9.345 17.374 1.00 1.00

ATOM 153 OH TYR A 26 -19.042 10.027 18.566 1.00 1.00

ATOM 154 N PHE A 27 -18.104 4.891 11.753 1.00 1.00

ATOM 155 CA PHE A 27 -18.497 3.962 10.701 1.00 1.00

ATOM 156 C PHE A 27 -19.699 3.165 11.187 1.00 1.00

ATOM 157 O PHE A 27 -19.677 2.616 12.289 1.00 1.00

ATOM 158 CB PHE A 27 -17.349 2.992 10.385 1.00 1.00

ATOM 159 CG PHE A 27 -16.347 3.512 9.381 1.00 1.00

ATOM 160 CD1 PHE A 27 -16.293 4.860 9.033 1.00 1.00

ATOM 161 CD2 PHE A 27 -15.426 2.639 8.810 1.00 1.00

ATOM 162 CE1 PHE A 27 -15.352 5.324 8.114 1.00 1.00

ATOM 163 CE2 PHE A 27 -14.481 3.093 7.892 1.00 1.00

ATOM 164 CZ PHE A 27 -14.445 4.440 7.545 1.00 1.00

ATOM 165 N ARG A 28 -20.745 3.117 10.368 1.00 1.00

ATOM 166 CA ARG A 28 -21.869 2.205 10.597 1.00 1.00

ATOM 167 C ARG A 28 -22.107 1.369 9.345 1.00 1.00

ATOM 168 O ARG A 28 -21.578 1.679 8.277 1.00 1.00

ATOM 169 CB ARG A 28 -23.141 2.965 10.995 1.00 1.00

ATOM 170 CG ARG A 28 -23.101 3.584 12.395 1.00 1.00

ATOM 171 CD ARG A 28 -24.470 4.115 12.829 1.00 1.00

ATOM 172 NE ARG A 28 -24.997 5.157 11.939 1.00 1.00

ATOM 173 CZ ARG A 28 -24.716 6.456 12.029 1.00 1.00

ATOM 174 NH1 ARG A 28 -23.892 6.911 12.963 1.00 1.00

ATOM 175 NH2 ARG A 28 -25.259 7.309 11.170 1.00 1.00

ATOM 176 N GLY A 29 -22.896 0.306 9.493 1.00 1.00

ATOM 177 CA GLY A 29 -23.212 -0.596 8.397 1.00 1.00

ATOM 178 C GLY A 29 -22.004 -1.327 7.850 1.00 1.00

ATOM 179 O GLY A 29 -21.932 -1.610 6.656 1.00 1.00

ATOM 180 N VAL A 30 -21.049 -1.627 8.729 1.00 1.00

ATOM 181 CA VAL A 30 -19.859 -2.385 8.352 1.00 1.00

ATOM 182 C VAL A 30 -20.292 -3.712 7.698 1.00 1.00

ATOM 183 O VAL A 30 -21.150 -4.414 8.233 1.00 1.00

ATOM 184 CB VAL A 30 -18.918 -2.609 9.572 1.00 1.00

ATOM 185 CG1 VAL A 30 -17.822 -3.621 9.257 1.00 1.00

ATOM 186 CG2 VAL A 30 -18.297 -1.281 10.012 1.00 1.00

ATOM 187 N PRO A 31 -19.716 -4.033 6.522 1.00 1.00

ATOM 188 CA PRO A 31 -20.104 -5.205 5.737 1.00 1.00

ATOM 189 C PRO A 31 -19.880 -6.525 6.473 1.00 1.00

ATOM 190 O PRO A 31 -19.088 -6.581 7.421 1.00 1.00

ATOM 191 CB PRO A 31 -19.178 -5.124 4.517 1.00 1.00

ATOM 192 CG PRO A 31 -18.779 -3.700 4.438 1.00 1.00

ATOM 193 CD PRO A 31 -18.650 -3.271 5.853 1.00 1.00

ATOM 194 N SER A 32 -20.568 -7.574 6.023 1.00 1.00

ATOM 195 CA SER A 32 -20.443 -8.905 6.620 1.00 1.00

ATOM 196 C SER A 32 -19.067 -9.522 6.367 1.00 1.00

ATOM 197 O SER A 32 -18.652 -10.449 7.074 1.00 1.00

ATOM 198 CB SER A 32 -21.545 -9.832 6.103 1.00 1.00

ATOM 199 OG SER A 32 -21.382 -10.078 4.719 1.00 1.00

ATOM 200 N LYS A 33 -18.364 -9.010 5.358 1.00 1.00

ATOM 201 CA LYS A 33 -17.000 -9.453 5.064 1.00 1.00

ATOM 202 C LYS A 33 -15.987 -8.342 5.355 1.00 1.00

ATOM 203 O LYS A 33 -14.885 -8.330 4.813 1.00 1.00

ATOM 204 CB LYS A 33 -16.886 -9.975 3.626 1.00 1.00

ATOM 205 CG LYS A 33 -17.821 -11.145 3.326 1.00 1.00

ATOM 206 CD LYS A 33 -17.475 -11.812 2.008 1.00 1.00

ATOM 207 CE LYS A 33 -18.575 -12.753 1.551 1.00 1.00

ATOM 208 NZ LYS A 33 -19.801 -11.998 1.151 1.00 1.00

ATOM 209 N GLY A 34 -16.385 -7.417 6.224 1.00 1.00

ATOM 210 CA GLY A 34 -15.506 -6.371 6.734 1.00 1.00

ATOM 211 C GLY A 34 -15.196 -5.240 5.776 1.00 1.00

ATOM 212 O GLY A 34 -15.730 -5.177 4.667 1.00 1.00

ATOM 213 N ILE A 35 -14.341 -4.329 6.225 1.00 1.00

ATOM 214 CA ILE A 35 -13.742 -3.330 5.350 1.00 1.00

ATOM 215 C ILE A 35 -12.246 -3.610 5.254 1.00 1.00

ATOM 216 O ILE A 35 -11.521 -3.474 6.237 1.00 1.00

ATOM 217 CB ILE A 35 -13.993 -1.883 5.849 1.00 1.00

ATOM 218 CG1 ILE A 35 -15.501 -1.597 5.906 1.00 1.00

ATOM 219 CG2 ILE A 35 -13.276 -0.869 4.947 1.00 1.00

ATOM 220 CD1 ILE A 35 -15.879 -0.342 6.677 1.00 1.00

ATOM 221 N ALA A 36 -11.807 -4.020 4.065 1.00 1.00

ATOM 222 CA ALA A 36 -10.390 -4.254 3.782 1.00 1.00

ATOM 223 C ALA A 36 -9.657 -2.957 3.457 1.00 1.00

ATOM 224 O ALA A 36 -8.468 -2.813 3.754 1.00 1.00

ATOM 225 CB ALA A 36 -10.231 -5.250 2.641 1.00 1.00

ATOM 226 N ASN A 37 -10.370 -2.017 2.837 1.00 1.00

ATOM 227 CA ASN A 37 -9.788 -0.731 2.462 1.00 1.00

ATOM 228 C ASN A 37 -10.820 0.366 2.296 1.00 1.00

ATOM 229 O ASN A 37 -11.979 0.099 1.959 1.00 1.00

ATOM 230 CB ASN A 37 -8.953 -0.854 1.175 1.00 1.00

ATOM 231 CG ASN A 37 -9.789 -1.253 -0.026 1.00 1.00

ATOM 232 OD1 ASN A 37 -10.403 -0.405 -0.681 1.00 1.00

ATOM 233 ND2 ASN A 37 -9.812 -2.546 -0.328 1.00 1.00

ATOM 234 N CYS A 38 -10.378 1.598 2.533 1.00 1.00

ATOM 235 CA CYS A 38 -11.169 2.783 2.239 1.00 1.00

ATOM 236 C CYS A 38 -10.275 4.002 2.065 1.00 1.00

ATOM 237 O CYS A 38 -9.263 4.153 2.753 1.00 1.00

ATOM 238 CB CYS A 38 -12.245 3.027 3.314 1.00 1.00

ATOM 239 SG CYS A 38 -11.654 3.302 4.988 1.00 1.00

ATOM 240 N ASP A 39 -10.644 4.857 1.118 1.00 1.00

ATOM 241 CA ASP A 39 -9.971 6.134 0.929 1.00 1.00

ATOM 242 C ASP A 39 -11.003 7.217 0.637 1.00 1.00

ATOM 243 O ASP A 39 -11.888 7.043 -0.203 1.00 1.00

ATOM 244 CB ASP A 39 -8.889 6.059 -0.166 1.00 1.00

ATOM 245 CG ASP A 39 -9.457 5.767 -1.554 1.00 1.00

ATOM 246 OD1 ASP A 39 -10.029 4.674 -1.771 1.00 1.00

ATOM 247 OD2 ASP A 39 -9.321 6.633 -2.438 1.00 1.00

ATOM 248 N PHE A 40 -10.907 8.319 1.373 1.00 1.00

ATOM 249 CA PHE A 40 -11.807 9.452 1.177 1.00 1.00

ATOM 250 C PHE A 40 -11.163 10.764 1.616 1.00 1.00

ATOM 251 O PHE A 40 -10.140 10.768 2.307 1.00 1.00

ATOM 252 CB PHE A 40 -13.160 9.227 1.878 1.00 1.00

ATOM 253 CG PHE A 40 -13.050 8.792 3.314 1.00 1.00

ATOM 254 CD1 PHE A 40 -13.014 9.731 4.340 1.00 1.00

ATOM 255 CD2 PHE A 40 -13.008 7.438 3.644 1.00 1.00

ATOM 256 CE1 PHE A 40 -12.924 9.327 5.669 1.00 1.00

ATOM 257 CE2 PHE A 40 -12.915 7.028 4.975 1.00 1.00

ATOM 258 CZ PHE A 40 -12.875 7.973 5.985 1.00 1.00

ATOM 259 N VAL A 41 -11.769 11.869 1.190 1.00 1.00

ATOM 260 CA VAL A 41 -11.240 13.206 1.440 1.00 1.00

ATOM 261 C VAL A 41 -12.330 14.062 2.092 1.00 1.00

ATOM 262 O VAL A 41 -13.472 14.076 1.624 1.00 1.00

ATOM 263 CB VAL A 41 -10.760 13.872 0.108 1.00 1.00

ATOM 264 CG1 VAL A 41 -10.305 15.307 0.344 1.00 1.00

ATOM 265 CG2 VAL A 41 -9.640 13.055 -0.540 1.00 1.00

ATOM 266 N PHE A 42 -11.982 14.737 3.189 1.00 1.00

ATOM 267 CA PHE A 42 -12.872 15.723 3.816 1.00 1.00

ATOM 268 C PHE A 42 -12.344 17.138 3.588 1.00 1.00

ATOM 269 O PHE A 42 -11.141 17.342 3.448 1.00 1.00

ATOM 270 CB PHE A 42 -13.017 15.507 5.331 1.00 1.00

ATOM 271 CG PHE A 42 -13.733 14.233 5.725 1.00 1.00

ATOM 272 CD1 PHE A 42 -14.516 13.519 4.820 1.00 1.00

ATOM 273 CD2 PHE A 42 -13.641 13.774 7.038 1.00 1.00

ATOM 274 CE1 PHE A 42 -15.170 12.352 5.205 1.00 1.00

ATOM 275 CE2 PHE A 42 -14.292 12.612 7.442 1.00 1.00

ATOM 276 CZ PHE A 42 -15.065 11.897 6.522 1.00 1.00

ATOM 277 N ARG A 43 -13.255 18.107 3.571 1.00 1.00

ATOM 278 CA ARG A 43 -12.890 19.516 3.487 1.00 1.00

ATOM 279 C ARG A 43 -13.072 20.202 4.829 1.00 1.00

ATOM 280 O ARG A 43 -13.918 19.803 5.631 1.00 1.00

ATOM 281 CB ARG A 43 -13.723 20.239 2.431 1.00 1.00

ATOM 282 CG ARG A 43 -13.233 20.015 1.025 1.00 1.00

ATOM 283 CD ARG A 43 -13.784 21.051 0.068 1.00 1.00

ATOM 284 NE ARG A 43 -13.323 20.777 -1.287 1.00 1.00

ATOM 285 CZ ARG A 43 -12.280 21.361 -1.863 1.00 1.00

ATOM 286 NH1 ARG A 43 -11.585 22.293 -1.219 1.00 1.00

ATOM 287 NH2 ARG A 43 -11.941 21.023 -3.100 1.00 1.00

ATOM 288 N TYR A 44 -12.269 21.237 5.057 1.00 1.00

ATOM 289 CA TYR A 44 -12.376 22.054 6.260 1.00 1.00

ATOM 290 C TYR A 44 -11.860 23.462 5.993 1.00 1.00

ATOM 291 O TYR A 44 -11.144 23.696 5.014 1.00 1.00

ATOM 292 CB TYR A 44 -11.609 21.413 7.423 1.00 1.00

ATOM 293 CG TYR A 44 -10.106 21.598 7.378 1.00 1.00

ATOM 294 CD1 TYR A 44 -9.475 22.518 8.217 1.00 1.00

ATOM 295 CD2 TYR A 44 -9.310 20.844 6.507 1.00 1.00

ATOM 296 CE1 TYR A 44 -8.101 22.688 8.192 1.00 1.00

ATOM 297 CE2 TYR A 44 -7.928 21.005 6.475 1.00 1.00

ATOM 298 CZ TYR A 44 -7.330 21.930 7.320 1.00 1.00

ATOM 299 OH TYR A 44 -5.964 22.097 7.297 1.00 1.00

ATOM 300 N ASP A 45 -12.224 24.395 6.864 1.00 1.00

ATOM 301 CA ASP A 45 -11.783 25.772 6.723 1.00 1.00

ATOM 302 C ASP A 45 -10.691 26.064 7.751 1.00 1.00

ATOM 303 O ASP A 45 -10.970 26.126 8.952 1.00 1.00

ATOM 304 CB ASP A 45 -12.974 26.732 6.868 1.00 1.00

ATOM 305 CG ASP A 45 -12.636 28.156 6.475 1.00 1.00

ATOM 306 OD1 ASP A 45 -11.469 28.571 6.640 1.00 1.00

ATOM 307 OD2 ASP A 45 -13.547 28.872 5.999 1.00 1.00

ATOM 308 N PRO A 46 -9.440 26.240 7.277 1.00 1.00

ATOM 309 CA PRO A 46 -8.282 26.456 8.148 1.00 1.00

ATOM 310 C PRO A 46 -8.304 27.793 8.901 1.00 1.00

ATOM 311 O PRO A 46 -7.526 27.975 9.834 1.00 1.00

ATOM 312 CB PRO A 46 -7.093 26.390 7.182 1.00 1.00

ATOM 313 CG PRO A 46 -7.654 26.761 5.861 1.00 1.00

ATOM 314 CD PRO A 46 -9.060 26.238 5.850 1.00 1.00

ATOM 315 N ASN A 47 -9.184 28.710 8.494 1.00 1.00

ATOM 316 CA ASN A 47 -9.424 29.949 9.236 1.00 1.00

ATOM 317 C ASN A 47 -10.141 29.685 10.554 1.00 1.00

ATOM 318 O ASN A 47 -9.995 30.444 11.515 1.00 1.00

ATOM 319 CB ASN A 47 -10.263 30.932 8.412 1.00 1.00

ATOM 320 CG ASN A 47 -9.490 31.561 7.274 1.00 1.00

ATOM 321 OD1 ASN A 47 -8.267 31.675 7.324 1.00 1.00

ATOM 322 ND2 ASN A 47 -10.209 31.989 6.239 1.00 1.00

ATOM 323 N VAL A 48 -10.923 28.608 10.578 1.00 1.00

ATOM 324 CA VAL A 48 -11.731 28.253 11.740 1.00 1.00

ATOM 325 C VAL A 48 -11.065 27.147 12.558 1.00 1.00

ATOM 326 O VAL A 48 -11.102 27.169 13.794 1.00 1.00

ATOM 327 CB VAL A 48 -13.158 27.804 11.315 1.00 1.00

ATOM 328 CG1 VAL A 48 -14.020 27.501 12.530 1.00 1.00

ATOM 329 CG2 VAL A 48 -13.823 28.868 10.439 1.00 1.00

ATOM 330 N LEU A 49 -10.459 26.185 11.865 1.00 1.00

ATOM 331 CA LEU A 49 -9.901 25.000 12.509 1.00 1.00

ATOM 332 C LEU A 49 -8.456 24.760 12.092 1.00 1.00

ATOM 333 O LEU A 49 -8.156 24.657 10.903 1.00 1.00

ATOM 334 CB LEU A 49 -10.750 23.755 12.191 1.00 1.00

ATOM 335 CG LEU A 49 -12.220 23.687 12.636 1.00 1.00

ATOM 336 CD1 LEU A 49 -12.843 22.346 12.251 1.00 1.00

ATOM 337 CD2 LEU A 49 -12.380 23.937 14.133 1.00 1.00

ATOM 338 N GLU A 50 -7.566 24.689 13.079 1.00 1.00

ATOM 339 CA GLU A 50 -6.204 24.235 12.842 1.00 1.00

ATOM 340 C GLU A 50 -6.115 22.755 13.195 1.00 1.00

ATOM 341 O GLU A 50 -6.076 22.386 14.372 1.00 1.00

ATOM 342 CB GLU A 50 -5.192 25.036 13.664 1.00 1.00

ATOM 343 CG GLU A 50 -3.741 24.630 13.405 1.00 1.00

ATOM 344 CD GLU A 50 -2.751 25.280 14.353 1.00 1.00

ATOM 345 OE1 GLU A 50 -3.175 25.852 15.384 1.00 1.00

ATOM 346 OE2 GLU A 50 -1.538 25.207 14.068 1.00 1.00

ATOM 347 N ILE A 51 -6.087 21.912 12.169 1.00 1.00

ATOM 348 CA ILE A 51 -6.007 20.471 12.371 1.00 1.00

ATOM 349 C ILE A 51 -4.571 20.076 12.711 1.00 1.00

ATOM 350 O ILE A 51 -3.655 20.237 11.896 1.00 1.00

ATOM 351 CB ILE A 51 -6.561 19.686 11.158 1.00 1.00

ATOM 352 CG1 ILE A 51 -8.013 20.111 10.893 1.00 1.00

ATOM 353 CG2 ILE A 51 -6.438 18.178 11.402 1.00 1.00

ATOM 354 CD1 ILE A 51 -8.740 19.316 9.832 1.00 1.00

ATOM 355 N ILE A 52 -4.388 19.572 13.926 1.00 1.00

ATOM 356 CA ILE A 52 -3.055 19.254 14.438 1.00 1.00

ATOM 357 C ILE A 52 -2.692 17.773 14.299 1.00 1.00

ATOM 358 O ILE A 52 -1.512 17.415 14.308 1.00 1.00

ATOM 359 CB ILE A 52 -2.847 19.768 15.891 1.00 1.00

ATOM 360 CG1 ILE A 52 -3.823 19.103 16.865 1.00 1.00

ATOM 361 CG2 ILE A 52 -2.968 21.304 15.936 1.00 1.00

ATOM 362 CD1 ILE A 52 -3.498 19.357 18.333 1.00 1.00

ATOM 363 N GLY A 53 -3.699 16.922 14.145 1.00 1.00

ATOM 364 CA GLY A 53 -3.460 15.498 13.937 1.00 1.00

ATOM 365 C GLY A 53 -4.724 14.681 13.776 1.00 1.00

ATOM 366 O GLY A 53 -5.817 15.135 14.119 1.00 1.00

ATOM 367 N ILE A 54 -4.563 13.477 13.233 1.00 1.00

ATOM 368 CA ILE A 54 -5.647 12.511 13.108 1.00 1.00

ATOM 369 C ILE A 54 -5.165 11.162 13.652 1.00 1.00

ATOM 370 O ILE A 54 -4.171 10.608 13.176 1.00 1.00

ATOM 371 CB ILE A 54 -6.137 12.372 11.641 1.00 1.00

ATOM 372 CG1 ILE A 54 -6.542 13.742 11.071 1.00 1.00

ATOM 373 CG2 ILE A 54 -7.305 11.394 11.553 1.00 1.00

ATOM 374 CD1 ILE A 54 -6.518 13.825 9.547 1.00 1.00

ATOM 375 N ASP A 55 -5.863 10.651 14.662 1.00 1.00

ATOM 376 CA ASP A 55 -5.512 9.369 15.265 1.00 1.00

ATOM 377 C ASP A 55 -6.453 8.279 14.775 1.00 1.00

ATOM 378 O ASP A 55 -7.631 8.551 14.535 1.00 1.00

ATOM 379 CB ASP A 55 -5.585 9.455 16.791 1.00 1.00

ATOM 380 CG ASP A 55 -4.680 10.532 17.358 1.00 1.00

ATOM 381 OD1 ASP A 55 -3.547 10.696 16.860 1.00 1.00

ATOM 382 OD2 ASP A 55 -5.108 11.217 18.309 1.00 1.00

ATOM 383 N PRO A 56 -5.939 7.042 14.622 1.00 1.00

ATOM 384 CA PRO A 56 -6.809 5.920 14.262 1.00 1.00

ATOM 385 C PRO A 56 -7.776 5.572 15.396 1.00 1.00

ATOM 386 O PRO A 56 -7.406 5.646 16.571 1.00 1.00

ATOM 387 CB PRO A 56 -5.824 4.769 14.015 1.00 1.00

ATOM 388 CG PRO A 56 -4.608 5.127 14.805 1.00 1.00

ATOM 389 CD PRO A 56 -4.528 6.627 14.764 1.00 1.00

ATOM 390 N GLY A 57 -9.007 5.220 15.041 1.00 1.00

ATOM 391 CA GLY A 57 -10.006 4.802 16.020 1.00 1.00

ATOM 392 C GLY A 57 -9.790 3.362 16.454 1.00 1.00

ATOM 393 O GLY A 57 -8.947 2.655 15.893 1.00 1.00

ATOM 394 N ASP A 58 -10.563 2.917 17.440 1.00 1.00

ATOM 395 CA ASP A 58 -10.371 1.577 17.995 1.00 1.00

ATOM 396 C ASP A 58 -10.884 0.424 17.119 1.00 1.00

ATOM 397 O ASP A 58 -10.655 -0.744 17.442 1.00 1.00

ATOM 398 CB ASP A 58 -10.904 1.482 19.437 1.00 1.00

ATOM 399 CG ASP A 58 -12.396 1.749 19.546 1.00 1.00

ATOM 400 OD1 ASP A 58 -13.017 2.216 18.566 1.00 1.00

ATOM 401 OD2 ASP A 58 -12.950 1.500 20.637 1.00 1.00

ATOM 402 N ILE A 59 -11.555 0.743 16.010 1.00 1.00

ATOM 403 CA ILE A 59 -11.946 -0.287 15.031 1.00 1.00

ATOM 404 C ILE A 59 -10.793 -0.644 14.082 1.00 1.00

ATOM 405 O ILE A 59 -10.927 -1.528 13.230 1.00 1.00

ATOM 406 CB ILE A 59 -13.239 0.077 14.230 1.00 1.00

ATOM 407 CG1 ILE A 59 -12.989 1.236 13.248 1.00 1.00

ATOM 408 CG2 ILE A 59 -14.405 0.337 15.190 1.00 1.00

ATOM 409 CD1 ILE A 59 -14.217 1.656 12.437 1.00 1.00

ATOM 410 N ILE A 60 -9.671 0.060 14.238 1.00 1.00

ATOM 411 CA ILE A 60 -8.428 -0.266 13.551 1.00 1.00

ATOM 412 C ILE A 60 -7.673 -1.236 14.455 1.00 1.00

ATOM 413 O ILE A 60 -7.156 -0.840 15.504 1.00 1.00

ATOM 414 CB ILE A 60 -7.564 1.003 13.275 1.00 1.00

ATOM 415 CG1 ILE A 60 -8.377 2.102 12.561 1.00 1.00

ATOM 416 CG2 ILE A 60 -6.279 0.652 12.510 1.00 1.00

ATOM 417 CD1 ILE A 60 -9.014 1.695 11.227 1.00 1.00

ATOM 418 N VAL A 61 -7.622 -2.504 14.051 1.00 1.00

ATOM 419 CA VAL A 61 -7.140 -3.583 14.926 1.00 1.00

ATOM 420 C VAL A 61 -5.694 -4.023 14.689 1.00 1.00

ATOM 421 O VAL A 61 -5.146 -4.786 15.490 1.00 1.00

ATOM 422 CB VAL A 61 -8.084 -4.822 14.917 1.00 1.00

ATOM 423 CG1 VAL A 61 -9.456 -4.455 15.489 1.00 1.00

ATOM 424 CG2 VAL A 61 -8.207 -5.422 13.510 1.00 1.00

ATOM 425 N ASP A 62 -5.084 -3.553 13.601 1.00 1.00

ATOM 426 CA ASP A 62 -3.665 -3.810 13.324 1.00 1.00

ATOM 427 C ASP A 62 -2.839 -3.368 14.543 1.00 1.00

ATOM 428 O ASP A 62 -2.880 -2.195 14.913 1.00 1.00

ATOM 429 CB ASP A 62 -3.233 -3.058 12.054 1.00 1.00

ATOM 430 CG ASP A 62 -1.855 -3.485 11.531 1.00 1.00

ATOM 431 OD1 ASP A 62 -1.028 -4.009 12.309 1.00 1.00

ATOM 432 OD2 ASP A 62 -1.591 -3.279 10.325 1.00 1.00

ATOM 433 N PRO A 63 -2.114 -4.315 15.186 1.00 1.00

ATOM 434 CA PRO A 63 -1.308 -3.997 16.378 1.00 1.00

ATOM 435 C PRO A 63 -0.228 -2.938 16.130 1.00 1.00

ATOM 436 O PRO A 63 0.291 -2.346 17.085 1.00 1.00

ATOM 437 CB PRO A 63 -0.673 -5.344 16.750 1.00 1.00

ATOM 438 CG PRO A 63 -0.741 -6.162 15.511 1.00 1.00

ATOM 439 CD PRO A 63 -2.016 -5.742 14.839 1.00 1.00

ATOM 440 N ASN A 64 0.112 -2.730 14.858 1.00 1.00

ATOM 441 CA ASN A 64 0.863 -1.556 14.421 1.00 1.00

ATOM 442 C ASN A 64 -0.026 -0.763 13.466 1.00 1.00

ATOM 443 O ASN A 64 0.013 -0.989 12.255 1.00 1.00

ATOM 444 CB ASN A 64 2.166 -1.958 13.728 1.00 1.00

ATOM 445 CG ASN A 64 3.038 -2.853 14.587 1.00 1.00

ATOM 446 OD1 ASN A 64 3.329 -3.986 14.214 1.00 1.00

ATOM 447 ND2 ASN A 64 3.450 -2.349 15.746 1.00 1.00

ATOM 448 N PRO A 65 -0.847 0.154 14.010 1.00 1.00

ATOM 449 CA PRO A 65 -1.883 0.851 13.235 1.00 1.00

ATOM 450 C PRO A 65 -1.380 1.569 11.979 1.00 1.00

ATOM 451 O PRO A 65 -2.113 1.633 10.995 1.00 1.00

ATOM 452 CB PRO A 65 -2.454 1.855 14.239 1.00 1.00

ATOM 453 CG PRO A 65 -2.173 1.249 15.570 1.00 1.00

ATOM 454 CD PRO A 65 -0.846 0.583 15.421 1.00 1.00

ATOM 455 N THR A 66 -0.148 2.082 12.015 1.00 1.00

ATOM 456 CA THR A 66 0.414 2.869 10.904 1.00 1.00

ATOM 457 C THR A 66 0.711 2.039 9.652 1.00 1.00

ATOM 458 O THR A 66 0.895 2.591 8.564 1.00 1.00

ATOM 459 CB THR A 66 1.682 3.668 11.322 1.00 1.00

ATOM 460 OG1 THR A 66 2.739 2.766 11.675 1.00 1.00

ATOM 461 CG2 THR A 66 1.377 4.591 12.500 1.00 1.00

ATOM 462 N LYS A 67 0.757 0.717 9.816 1.00 1.00

ATOM 463 CA LYS A 67 0.872 -0.213 8.692 1.00 1.00

ATOM 464 C LYS A 67 -0.420 -0.272 7.870 1.00 1.00

ATOM 465 O LYS A 67 -0.380 -0.495 6.655 1.00 1.00

ATOM 466 CB LYS A 67 1.235 -1.616 9.189 1.00 1.00

ATOM 467 CG LYS A 67 2.642 -1.753 9.772 1.00 1.00

ATOM 468 CD LYS A 67 3.694 -1.939 8.689 1.00 1.00

ATOM 469 CE LYS A 67 5.101 -1.965 9.284 1.00 1.00

ATOM 470 NZ LYS A 67 6.143 -2.112 8.226 1.00 1.00

ATOM 471 N SER A 68 -1.558 -0.077 8.533 1.00 1.00

ATOM 472 CA SER A 68 -2.866 -0.144 7.874 1.00 1.00

ATOM 473 C SER A 68 -3.509 1.224 7.651 1.00 1.00

ATOM 474 O SER A 68 -4.315 1.389 6.740 1.00 1.00

ATOM 475 CB SER A 68 -3.831 -1.028 8.675 1.00 1.00

ATOM 476 OG SER A 68 -3.453 -2.391 8.626 1.00 1.00

ATOM 477 N PHE A 69 -3.144 2.195 8.483 1.00 1.00

ATOM 478 CA PHE A 69 -3.851 3.471 8.563 1.00 1.00

ATOM 479 C PHE A 69 -2.884 4.640 8.403 1.00 1.00

ATOM 480 O PHE A 69 -1.877 4.716 9.102 1.00 1.00

ATOM 481 CB PHE A 69 -4.571 3.549 9.917 1.00 1.00

ATOM 482 CG PHE A 69 -5.376 4.809 10.133 1.00 1.00

ATOM 483 CD1 PHE A 69 -6.734 4.836 9.848 1.00 1.00

ATOM 484 CD2 PHE A 69 -4.784 5.952 10.668 1.00 1.00

ATOM 485 CE1 PHE A 69 -7.490 5.990 10.065 1.00 1.00

ATOM 486 CE2 PHE A 69 -5.525 7.113 10.886 1.00 1.00

ATOM 487 CZ PHE A 69 -6.880 7.131 10.584 1.00 1.00

ATOM 488 N ASP A 70 -3.196 5.546 7.478 1.00 1.00

ATOM 489 CA ASP A 70 -2.423 6.777 7.305 1.00 1.00

ATOM 490 C ASP A 70 -3.351 7.918 6.903 1.00 1.00

ATOM 491 O ASP A 70 -4.422 7.690 6.324 1.00 1.00

ATOM 492 CB ASP A 70 -1.315 6.590 6.250 1.00 1.00

ATOM 493 CG ASP A 70 -0.162 7.592 6.408 1.00 1.00

ATOM 494 OD1 ASP A 70 -0.284 8.571 7.176 1.00 1.00

ATOM 495 OD2 ASP A 70 0.889 7.385 5.770 1.00 1.00

ATOM 496 N THR A 71 -2.943 9.140 7.229 1.00 1.00

ATOM 497 CA THR A 71 -3.674 10.340 6.833 1.00 1.00

ATOM 498 C THR A 71 -2.712 11.412 6.315 1.00 1.00

ATOM 499 O THR A 71 -1.494 11.303 6.491 1.00 1.00

ATOM 500 CB THR A 71 -4.499 10.931 8.007 1.00 1.00

ATOM 501 OG1 THR A 71 -3.630 11.235 9.100 1.00 1.00

ATOM 502 CG2 THR A 71 -5.590 9.966 8.472 1.00 1.00

ATOM 503 N ALA A 72 -3.270 12.444 5.684 1.00 1.00

ATOM 504 CA ALA A 72 -2.500 13.610 5.240 1.00 1.00

ATOM 505 C ALA A 72 -3.314 14.893 5.414 1.00 1.00

ATOM 506 O ALA A 72 -4.512 14.924 5.121 1.00 1.00

ATOM 507 CB ALA A 72 -2.051 13.441 3.790 1.00 1.00

ATOM 508 N ILE A 73 -2.658 15.943 5.906 1.00 1.00

ATOM 509 CA ILE A 73 -3.309 17.235 6.151 1.00 1.00

ATOM 510 C ILE A 73 -2.742 18.301 5.202 1.00 1.00

ATOM 511 O ILE A 73 -1.524 18.502 5.140 1.00 1.00

ATOM 512 CB ILE A 73 -3.158 17.674 7.644 1.00 1.00

ATOM 513 CG1 ILE A 73 -3.815 16.648 8.576 1.00 1.00

ATOM 514 CG2 ILE A 73 -3.743 19.074 7.874 1.00 1.00

ATOM 515 CD1 ILE A 73 -3.249 16.608 9.996 1.00 1.00

ATOM 516 N TYR A 74 -3.634 18.967 4.465 1.00 1.00

ATOM 517 CA TYR A 74 -3.256 20.028 3.519 1.00 1.00

ATOM 518 C TYR A 74 -3.990 21.344 3.795 1.00 1.00

ATOM 519 O TYR A 74 -5.051 21.594 3.220 1.00 1.00

ATOM 520 CB TYR A 74 -3.529 19.597 2.074 1.00 1.00

ATOM 521 CG TYR A 74 -2.895 18.294 1.665 1.00 1.00

ATOM 522 CD1 TYR A 74 -3.638 17.116 1.647 1.00 1.00

ATOM 523 CD2 TYR A 74 -1.553 18.236 1.291 1.00 1.00

ATOM 524 CE1 TYR A 74 -3.059 15.914 1.271 1.00 1.00

ATOM 525 CE2 TYR A 74 -0.967 17.033 0.912 1.00 1.00

ATOM 526 CZ TYR A 74 -1.728 15.882 0.903 1.00 1.00

ATOM 527 OH TYR A 74 -1.154 14.698 0.531 1.00 1.00

ATOM 528 N PRO A 75 -3.426 22.197 4.670 1.00 1.00

ATOM 529 CA PRO A 75 -4.018 23.507 4.973 1.00 1.00

ATOM 530 C PRO A 75 -4.176 24.403 3.737 1.00 1.00

ATOM 531 O PRO A 75 -5.226 25.029 3.573 1.00 1.00

ATOM 532 CB PRO A 75 -3.025 24.121 5.969 1.00 1.00

ATOM 533 CG PRO A 75 -2.319 22.958 6.562 1.00 1.00

ATOM 534 CD PRO A 75 -2.197 21.967 5.448 1.00 1.00

ATOM 535 N ASP A 76 -3.156 24.444 2.877 1.00 1.00

ATOM 536 CA ASP A 76 -3.205 25.231 1.635 1.00 1.00

ATOM 537 C ASP A 76 -4.327 24.754 0.705 1.00 1.00

ATOM 538 O ASP A 76 -4.958 25.558 0.018 1.00 1.00

ATOM 539 CB ASP A 76 -1.859 25.175 0.893 1.00 1.00

ATOM 540 CG ASP A 76 -0.707 25.793 1.686 1.00 1.00

ATOM 541 OD1 ASP A 76 -0.662 25.653 2.931 1.00 1.00

ATOM 542 OD2 ASP A 76 0.176 26.408 1.051 1.00 1.00

ATOM 543 N ARG A 77 -4.563 23.445 0.695 1.00 1.00

ATOM 544 CA ARG A 77 -5.591 22.833 -0.143 1.00 1.00

ATOM 545 C ARG A 77 -6.939 22.708 0.571 1.00 1.00

ATOM 546 O ARG A 77 -7.957 22.414 -0.063 1.00 1.00

ATOM 547 CB ARG A 77 -5.134 21.452 -0.613 1.00 1.00

ATOM 548 CG ARG A 77 -4.158 21.479 -1.768 1.00 1.00

ATOM 549 CD ARG A 77 -3.869 20.072 -2.252 1.00 1.00

ATOM 550 NE ARG A 77 -2.506 19.644 -1.946 1.00 1.00

ATOM 551 CZ ARG A 77 -1.988 18.476 -2.318 1.00 1.00

ATOM 552 NH1 ARG A 77 -2.724 17.607 -3.000 1.00 1.00

ATOM 553 NH2 ARG A 77 -0.733 18.177 -2.008 1.00 1.00

ATOM 554 N LYS A 78 -6.930 22.920 1.885 1.00 1.00

ATOM 555 CA LYS A 78 -8.135 22.888 2.726 1.00 1.00

ATOM 556 C LYS A 78 -8.792 21.505 2.781 1.00 1.00

ATOM 557 O LYS A 78 -10.015 21.390 2.880 1.00 1.00

ATOM 558 CB LYS A 78 -9.151 23.946 2.274 1.00 1.00

ATOM 559 CG LYS A 78 -8.625 25.360 2.285 1.00 1.00

ATOM 560 CD LYS A 78 -9.634 26.321 1.688 1.00 1.00

ATOM 561 CE LYS A 78 -9.181 27.763 1.868 1.00 1.00

ATOM 562 NZ LYS A 78 -7.787 27.984 1.368 1.00 1.00

ATOM 563 N ILE A 79 -7.970 20.461 2.720 1.00 1.00

ATOM 564 CA ILE A 79 -8.463 19.086 2.764 1.00 1.00

ATOM 565 C ILE A 79 -7.667 18.209 3.729 1.00 1.00

ATOM 566 O ILE A 79 -6.512 18.503 4.059 1.00 1.00

ATOM 567 CB ILE A 79 -8.482 18.409 1.351 1.00 1.00

ATOM 568 CG1 ILE A 79 -7.081 18.402 0.714 1.00 1.00

ATOM 569 CG2 ILE A 79 -9.541 19.057 0.447 1.00 1.00

ATOM 570 CD1 ILE A 79 -6.961 17.600 -0.579 1.00 1.00

ATOM 571 N ILE A 80 -8.315 17.138 4.178 1.00 1.00

ATOM 572 CA ILE A 80 -7.661 16.054 4.909 1.00 1.00

ATOM 573 C ILE A 80 -7.984 14.735 4.212 1.00 1.00

ATOM 574 O ILE A 80 -9.098 14.538 3.721 1.00 1.00

ATOM 575 CB ILE A 80 -8.052 16.015 6.417 1.00 1.00

ATOM 576 CG1 ILE A 80 -9.573 16.031 6.606 1.00 1.00

ATOM 577 CG2 ILE A 80 -7.394 17.178 7.165 1.00 1.00

ATOM 578 CD1 ILE A 80 -10.036 15.752 8.044 1.00 1.00

ATOM 579 N VAL A 81 -7.002 13.844 4.145 1.00 1.00

ATOM 580 CA VAL A 81 -7.142 12.609 3.375 1.00 1.00

ATOM 581 C VAL A 81 -6.954 11.393 4.284 1.00 1.00

ATOM 582 O VAL A 81 -6.085 11.394 5.155 1.00 1.00

ATOM 583 CB VAL A 81 -6.133 12.565 2.186 1.00 1.00

ATOM 584 CG1 VAL A 81 -6.481 11.432 1.205 1.00 1.00

ATOM 585 CG2 VAL A 81 -6.092 13.909 1.456 1.00 1.00

ATOM 586 N PHE A 82 -7.787 10.375 4.085 1.00 1.00

ATOM 587 CA PHE A 82 -7.749 9.151 4.880 1.00 1.00

ATOM 588 C PHE A 82 -7.448 7.960 3.982 1.00 1.00

ATOM 589 O PHE A 82 -8.071 7.800 2.927 1.00 1.00

ATOM 590 CB PHE A 82 -9.093 8.913 5.587 1.00 1.00

ATOM 591 CG PHE A 82 -9.513 10.022 6.525 1.00 1.00

ATOM 592 CD1 PHE A 82 -10.103 11.189 6.034 1.00 1.00

ATOM 593 CD2 PHE A 82 -9.348 9.885 7.903 1.00 1.00

ATOM 594 CE1 PHE A 82 -10.502 12.204 6.899 1.00 1.00

ATOM 595 CE2 PHE A 82 -9.750 10.897 8.776 1.00 1.00

ATOM 596 CZ PHE A 82 -10.326 12.058 8.276 1.00 1.00

ATOM 597 N LEU A 83 -6.500 7.127 4.400 1.00 1.00

ATOM 598 CA LEU A 83 -6.200 5.882 3.695 1.00 1.00

ATOM 599 C LEU A 83 -6.121 4.697 4.664 1.00 1.00

ATOM 600 O LEU A 83 -5.244 4.641 5.526 1.00 1.00

ATOM 601 CB LEU A 83 -4.905 6.001 2.875 1.00 1.00

ATOM 602 CG LEU A 83 -4.393 4.762 2.118 1.00 1.00

ATOM 603 CD1 LEU A 83 -5.351 4.314 0.996 1.00 1.00

ATOM 604 CD2 LEU A 83 -3.008 5.007 1.557 1.00 1.00

ATOM 605 N PHE A 84 -7.066 3.772 4.518 1.00 1.00

ATOM 606 CA PHE A 84 -7.031 2.506 5.235 1.00 1.00

ATOM 607 C PHE A 84 -6.895 1.361 4.237 1.00 1.00

ATOM 608 O PHE A 84 -7.620 1.299 3.248 1.00 1.00

ATOM 609 CB PHE A 84 -8.284 2.313 6.099 1.00 1.00

ATOM 610 CG PHE A 84 -8.361 0.959 6.762 1.00 1.00

ATOM 611 CD1 PHE A 84 -7.604 0.681 7.897 1.00 1.00

ATOM 612 CD2 PHE A 84 -9.182 -0.038 6.244 1.00 1.00

ATOM 613 CE1 PHE A 84 -7.666 -0.576 8.514 1.00 1.00

ATOM 614 CE2 PHE A 84 -9.253 -1.296 6.850 1.00 1.00

ATOM 615 CZ PHE A 84 -8.493 -1.564 7.988 1.00 1.00

ATOM 616 N ALA A 85 -5.942 0.471 4.500 1.00 1.00

ATOM 617 CA ALA A 85 -5.773 -0.748 3.723 1.00 1.00

ATOM 618 C ALA A 85 -5.183 -1.789 4.658 1.00 1.00

ATOM 619 O ALA A 85 -3.995 -1.720 5.003 1.00 1.00

ATOM 620 CB ALA A 85 -4.854 -0.516 2.518 1.00 1.00

ATOM 621 N GLU A 86 -6.024 -2.724 5.092 1.00 1.00

ATOM 622 CA GLU A 86 -5.623 -3.719 6.083 1.00 1.00

ATOM 623 C GLU A 86 -4.335 -4.419 5.631 1.00 1.00

ATOM 624 O GLU A 86 -4.236 -4.867 4.486 1.00 1.00

ATOM 625 CB GLU A 86 -6.780 -4.690 6.378 1.00 1.00

ATOM 626 CG GLU A 86 -7.037 -5.778 5.339 1.00 1.00

ATOM 627 CD GLU A 86 -6.175 -7.011 5.572 1.00 1.00

ATOM 628 OE1 GLU A 86 -5.709 -7.195 6.715 1.00 1.00

ATOM 629 OE2 GLU A 86 -5.959 -7.788 4.617 1.00 1.00

ATOM 630 N ASP A 87 -3.346 -4.474 6.522 1.00 1.00

ATOM 631 CA ASP A 87 -1.975 -4.830 6.125 1.00 1.00

ATOM 632 C ASP A 87 -1.632 -6.324 6.181 1.00 1.00

ATOM 633 O ASP A 87 -0.612 -6.738 5.624 1.00 1.00

ATOM 634 CB ASP A 87 -0.941 -4.011 6.925 1.00 1.00

ATOM 635 CG ASP A 87 0.443 -4.001 6.266 1.00 1.00

ATOM 636 OD1 ASP A 87 0.548 -3.685 5.066 1.00 1.00

ATOM 637 OD2 ASP A 87 1.436 -4.305 6.960 1.00 1.00

ATOM 638 N SER A 88 -2.478 -7.127 6.823 1.00 1.00

ATOM 639 CA SER A 88 -2.232 -8.574 6.936 1.00 1.00

ATOM 640 C SER A 88 -2.112 -9.263 5.571 1.00 1.00

ATOM 641 O SER A 88 -1.280 -10.152 5.387 1.00 1.00

ATOM 642 CB SER A 88 -3.323 -9.254 7.771 1.00 1.00

ATOM 643 OG SER A 88 -4.476 -9.523 6.991 1.00 1.00

ATOM 644 N GLY A 89 -2.946 -8.840 4.621 1.00 1.00

ATOM 645 CA GLY A 89 -3.019 -9.473 3.307 1.00 1.00

ATOM 646 C GLY A 89 -3.995 -10.636 3.261 1.00 1.00

ATOM 647 O GLY A 89 -4.243 -11.203 2.193 1.00 1.00

ATOM 648 N THR A 90 -4.548 -10.984 4.421 1.00 1.00

ATOM 649 CA THR A 90 -5.435 -12.142 4.559 1.00 1.00

ATOM 650 C THR A 90 -6.845 -11.740 4.997 1.00 1.00

ATOM 651 O THR A 90 -7.757 -12.572 5.021 1.00 1.00

ATOM 652 CB THR A 90 -4.871 -13.185 5.561 1.00 1.00

ATOM 653 OG1 THR A 90 -4.949 -12.668 6.896 1.00 1.00

ATOM 654 CG2 THR A 90 -3.424 -13.540 5.230 1.00 1.00

ATOM 655 N GLY A 91 -7.012 -10.465 5.331 1.00 1.00

ATOM 656 CA GLY A 91 -8.272 -9.948 5.855 1.00 1.00

ATOM 657 C GLY A 91 -8.318 -9.870 7.371 1.00 1.00

ATOM 658 O GLY A 91 -9.315 -9.429 7.941 1.00 1.00

ATOM 659 N ALA A 92 -7.234 -10.294 8.020 1.00 1.00

ATOM 660 CA ALA A 92 -7.168 -10.347 9.486 1.00 1.00

ATOM 661 C ALA A 92 -7.287 -8.978 10.163 1.00 1.00

ATOM 662 O ALA A 92 -7.823 -8.876 11.273 1.00 1.00

ATOM 663 CB ALA A 92 -5.898 -11.055 9.942 1.00 1.00

ATOM 664 N TYR A 93 -6.799 -7.933 9.496 1.00 1.00

ATOM 665 CA TYR A 93 -6.823 -6.585 10.071 1.00 1.00

ATOM 666 C TYR A 93 -7.925 -5.704 9.490 1.00 1.00

ATOM 667 O TYR A 93 -7.917 -4.483 9.677 1.00 1.00

ATOM 668 CB TYR A 93 -5.454 -5.899 9.943 1.00 1.00

ATOM 669 CG TYR A 93 -4.315 -6.594 10.667 1.00 1.00

ATOM 670 CD1 TYR A 93 -4.547 -7.401 11.782 1.00 1.00

ATOM 671 CD2 TYR A 93 -2.996 -6.419 10.248 1.00 1.00

ATOM 672 CE1 TYR A 93 -3.496 -8.034 12.442 1.00 1.00

ATOM 673 CE2 TYR A 93 -1.944 -7.038 10.906 1.00 1.00

ATOM 674 CZ TYR A 93 -2.198 -7.841 11.997 1.00 1.00

ATOM 675 OH TYR A 93 -1.149 -8.451 12.645 1.00 1.00

ATOM 676 N ALA A 94 -8.872 -6.326 8.789 1.00 1.00

ATOM 677 CA ALA A 94 -10.048 -5.623 8.281 1.00 1.00

ATOM 678 C ALA A 94 -10.872 -5.046 9.428 1.00 1.00

ATOM 679 O ALA A 94 -10.768 -5.506 10.569 1.00 1.00

ATOM 680 CB ALA A 94 -10.909 -6.555 7.428 1.00 1.00

ATOM 681 N ILE A 95 -11.675 -4.032 9.116 1.00 1.00

ATOM 682 CA ILE A 95 -12.630 -3.463 10.059 1.00 1.00

ATOM 683 C ILE A 95 -13.841 -4.394 10.109 1.00 1.00

ATOM 684 O ILE A 95 -14.432 -4.697 9.071 1.00 1.00

ATOM 685 CB ILE A 95 -13.034 -2.017 9.642 1.00 1.00

ATOM 686 CG1 ILE A 95 -11.823 -1.080 9.746 1.00 1.00

ATOM 687 CG2 ILE A 95 -14.201 -1.505 10.484 1.00 1.00

ATOM 688 CD1 ILE A 95 -11.958 0.230 8.982 1.00 1.00

ATOM 689 N THR A 96 -14.195 -4.857 11.306 1.00 1.00

ATOM 690 CA THR A 96 -15.245 -5.879 11.459 1.00 1.00

ATOM 691 C THR A 96 -16.461 -5.416 12.270 1.00 1.00

ATOM 692 O THR A 96 -17.512 -6.063 12.242 1.00 1.00

ATOM 693 CB THR A 96 -14.685 -7.189 12.084 1.00 1.00

ATOM 694 OG1 THR A 96 -13.990 -6.884 13.298 1.00 1.00

ATOM 695 CG2 THR A 96 -13.738 -7.884 11.127 1.00 1.00

ATOM 696 N LYS A 97 -16.320 -4.300 12.980 1.00 1.00

ATOM 697 CA LYS A 97 -17.417 -3.756 13.774 1.00 1.00

ATOM 698 C LYS A 97 -17.630 -2.267 13.506 1.00 1.00

ATOM 699 O LYS A 97 -16.707 -1.576 13.062 1.00 1.00

ATOM 700 CB LYS A 97 -17.171 -4.004 15.271 1.00 1.00

ATOM 701 CG LYS A 97 -15.984 -3.236 15.865 1.00 1.00

ATOM 702 CD LYS A 97 -15.708 -3.586 17.338 1.00 1.00

ATOM 703 CE LYS A 97 -16.844 -3.183 18.285 1.00 1.00

ATOM 704 NZ LYS A 97 -17.207 -1.741 18.195 1.00 1.00

ATOM 705 N ASP A 98 -18.845 -1.789 13.777 1.00 1.00

ATOM 706 CA ASP A 98 -19.152 -0.358 13.794 1.00 1.00

ATOM 707 C ASP A 98 -18.367 0.313 14.914 1.00 1.00

ATOM 708 O ASP A 98 -18.039 -0.326 15.914 1.00 1.00

ATOM 709 CB ASP A 98 -20.635 -0.118 14.081 1.00 1.00

ATOM 710 CG ASP A 98 -21.562 -0.618 12.982 1.00 1.00

ATOM 711 OD1 ASP A 98 -21.103 -1.090 11.916 1.00 1.00

ATOM 712 OD2 ASP A 98 -22.784 -0.518 13.203 1.00 1.00

ATOM 713 N GLY A 99 -18.092 1.606 14.761 1.00 1.00

ATOM 714 CA GLY A 99 -17.406 2.379 15.799 1.00 1.00

ATOM 715 C GLY A 99 -16.602 3.536 15.236 1.00 1.00

ATOM 716 O GLY A 99 -16.848 3.989 14.118 1.00 1.00

ATOM 717 N VAL A 100 -15.638 4.014 16.017 1.00 1.00

ATOM 718 CA VAL A 100 -14.830 5.166 15.621 1.00 1.00

ATOM 719 C VAL A 100 -13.690 4.717 14.712 1.00 1.00

ATOM 720 O VAL A 100 -12.858 3.891 15.101 1.00 1.00

ATOM 721 CB VAL A 100 -14.284 5.943 16.855 1.00 1.00

ATOM 722 CG1 VAL A 100 -13.367 7.079 16.423 1.00 1.00

ATOM 723 CG2 VAL A 100 -15.432 6.483 17.696 1.00 1.00

ATOM 724 N PHE A 101 -13.682 5.258 13.495 1.00 1.00

ATOM 725 CA PHE A 101 -12.631 5.001 12.510 1.00 1.00

ATOM 726 C PHE A 101 -11.405 5.892 12.738 1.00 1.00

ATOM 727 O PHE A 101 -10.267 5.439 12.611 1.00 1.00

ATOM 728 CB PHE A 101 -13.189 5.214 11.096 1.00 1.00

ATOM 729 CG PHE A 101 -12.143 5.214 10.003 1.00 1.00

ATOM 730 CD1 PHE A 101 -11.512 4.033 9.620 1.00 1.00

ATOM 731 CD2 PHE A 101 -11.826 6.390 9.330 1.00 1.00

ATOM 732 CE1 PHE A 101 -10.562 4.027 8.593 1.00 1.00

ATOM 733 CE2 PHE A 101 -10.876 6.395 8.304 1.00 1.00

ATOM 734 CZ PHE A 101 -10.243 5.211 7.937 1.00 1.00

ATOM 735 N ALA A 102 -11.655 7.158 13.057 1.00 1.00

ATOM 736 CA ALA A 102 -10.586 8.135 13.274 1.00 1.00

ATOM 737 C ALA A 102 -11.052 9.241 14.205 1.00 1.00

ATOM 738 O ALA A 102 -12.255 9.473 14.349 1.00 1.00

ATOM 739 CB ALA A 102 -10.110 8.725 11.940 1.00 1.00

ATOM 740 N LYS A 103 -10.091 9.907 14.841 1.00 1.00

ATOM 741 CA LYS A 103 -10.359 11.081 15.665 1.00 1.00

ATOM 742 C LYS A 103 -9.519 12.249 15.160 1.00 1.00

ATOM 743 O LYS A 103 -8.285 12.196 15.179 1.00 1.00

ATOM 744 CB LYS A 103 -10.069 10.795 17.141 1.00 1.00

ATOM 745 CG LYS A 103 -10.962 9.709 17.754 1.00 1.00

ATOM 746 CD LYS A 103 -10.514 9.288 19.162 1.00 1.00

ATOM 747 CE LYS A 103 -9.187 8.521 19.159 1.00 1.00

ATOM 748 NZ LYS A 103 -9.169 7.361 18.225 1.00 1.00

ATOM 749 N ILE A 104 -10.195 13.289 14.686 1.00 1.00

ATOM 750 CA ILE A 104 -9.529 14.478 14.160 1.00 1.00

ATOM 751 C ILE A 104 -9.290 15.454 15.307 1.00 1.00

ATOM 752 O ILE A 104 -10.241 15.879 15.970 1.00 1.00

ATOM 753 CB ILE A 104 -10.373 15.171 13.058 1.00 1.00

ATOM 754 CG1 ILE A 104 -10.782 14.169 11.970 1.00 1.00

ATOM 755 CG2 ILE A 104 -9.607 16.357 12.452 1.00 1.00

ATOM 756 CD1 ILE A 104 -12.005 14.589 11.162 1.00 1.00

ATOM 757 N ARG A 105 -8.023 15.786 15.548 1.00 1.00

ATOM 758 CA ARG A 105 -7.664 16.752 16.583 1.00 1.00

ATOM 759 C ARG A 105 -7.484 18.133 15.972 1.00 1.00

ATOM 760 O ARG A 105 -6.692 18.318 15.043 1.00 1.00

ATOM 761 CB ARG A 105 -6.400 16.325 17.334 1.00 1.00

ATOM 762 CG ARG A 105 -6.659 15.427 18.535 1.00 1.00

ATOM 763 CD ARG A 105 -5.368 14.962 19.208 1.00 1.00

ATOM 764 NE ARG A 105 -4.574 14.072 18.358 1.00 1.00

ATOM 765 CZ ARG A 105 -3.483 14.436 17.685 1.00 1.00

ATOM 766 NH1 ARG A 105 -3.028 15.682 17.753 1.00 1.00

ATOM 767 NH2 ARG A 105 -2.840 13.547 16.939 1.00 1.00

ATOM 768 N ALA A 106 -8.231 19.102 16.495 1.00 1.00

ATOM 769 CA ALA A 106 -8.243 20.448 15.935 1.00 1.00

ATOM 770 C ALA A 106 -8.298 21.529 17.010 1.00 1.00

ATOM 771 O ALA A 106 -9.077 21.433 17.964 1.00 1.00

ATOM 772 CB ALA A 106 -9.416 20.602 14.975 1.00 1.00

ATOM 773 N THR A 107 -7.471 22.556 16.839 1.00 1.00

ATOM 774 CA THR A 107 -7.539 23.757 17.667 1.00 1.00

ATOM 775 C THR A 107 -8.529 24.750 17.046 1.00 1.00

ATOM 776 O THR A 107 -8.474 25.031 15.846 1.00 1.00

ATOM 777 CB THR A 107 -6.148 24.403 17.835 1.00 1.00

ATOM 778 OG1 THR A 107 -5.219 23.420 18.299 1.00 1.00

ATOM 779 CG2 THR A 107 -6.194 25.560 18.834 1.00 1.00

ATOM 780 N VAL A 108 -9.436 25.259 17.874 1.00 1.00

ATOM 781 CA VAL A 108 -10.471 26.198 17.436 1.00 1.00

ATOM 782 C VAL A 108 -9.882 27.606 17.283 1.00 1.00

ATOM 783 O VAL A 108 -9.389 28.195 18.250 1.00 1.00

ATOM 784 CB VAL A 108 -11.667 26.209 18.424 1.00 1.00

ATOM 785 CG1 VAL A 108 -12.794 27.124 17.923 1.00 1.00

ATOM 786 CG2 VAL A 108 -12.188 24.793 18.650 1.00 1.00

ATOM 787 N LYS A 109 -9.926 28.131 16.062 1.00 1.00

ATOM 788 CA LYS A 109 -9.315 29.428 15.754 1.00 1.00

ATOM 789 C LYS A 109 -10.315 30.582 15.751 1.00 1.00

ATOM 790 O LYS A 109 -9.933 31.740 15.952 1.00 1.00

ATOM 791 CB LYS A 109 -8.566 29.372 14.420 1.00 1.00

ATOM 792 CG LYS A 109 -7.273 28.570 14.462 1.00 1.00

ATOM 793 CD LYS A 109 -6.663 28.407 13.065 1.00 1.00

ATOM 794 CE LYS A 109 -6.249 29.739 12.459 1.00 1.00

ATOM 795 NZ LYS A 109 -5.496 29.572 11.190 1.00 1.00

ATOM 796 N SER A 110 -11.584 30.272 15.504 1.00 1.00

ATOM 797 CA SER A 110 -12.647 31.273 15.587 1.00 1.00

ATOM 798 C SER A 110 -13.962 30.637 16.032 1.00 1.00

ATOM 799 O SER A 110 -14.119 29.416 15.972 1.00 1.00

ATOM 800 CB SER A 110 -12.817 32.014 14.249 1.00 1.00

ATOM 801 OG SER A 110 -13.853 31.460 13.462 1.00 1.00

ATOM 802 N SER A 111 -14.893 31.477 16.475 1.00 1.00

ATOM 803 CA SER A 111 -16.203 31.031 16.957 1.00 1.00

ATOM 804 C SER A 111 -17.192 30.711 15.832 1.00 1.00

ATOM 805 O SER A 111 -18.275 30.181 16.095 1.00 1.00

ATOM 806 CB SER A 111 -16.810 32.074 17.909 1.00 1.00

ATOM 807 OG SER A 111 -17.113 33.281 17.223 1.00 1.00

ATOM 808 N ALA A 112 -16.825 31.042 14.593 1.00 1.00

ATOM 809 CA ALA A 112 -17.637 30.685 13.425 1.00 1.00

ATOM 810 C ALA A 112 -17.697 29.159 13.297 1.00 1.00

ATOM 811 O ALA A 112 -16.781 28.471 13.748 1.00 1.00

ATOM 812 CB ALA A 112 -17.058 31.313 12.165 1.00 1.00

ATOM 813 N PRO A 113 -18.788 28.619 12.717 1.00 1.00

ATOM 814 CA PRO A 113 -18.859 27.163 12.563 1.00 1.00

ATOM 815 C PRO A 113 -17.661 26.597 11.804 1.00 1.00

ATOM 816 O PRO A 113 -17.212 27.187 10.814 1.00 1.00

ATOM 817 CB PRO A 113 -20.142 26.948 11.756 1.00 1.00

ATOM 818 CG PRO A 113 -20.961 28.146 12.006 1.00 1.00

ATOM 819 CD PRO A 113 -19.999 29.284 12.200 1.00 1.00

ATOM 820 N GLY A 114 -17.149 25.472 12.289 1.00 1.00

ATOM 821 CA GLY A 114 -16.038 24.778 11.659 1.00 1.00

ATOM 822 C GLY A 114 -16.499 23.461 11.076 1.00 1.00

ATOM 823 O GLY A 114 -16.257 22.401 11.650 1.00 1.00

ATOM 824 N TYR A 115 -17.175 23.535 9.934 1.00 1.00

ATOM 825 CA TYR A 115 -17.709 22.338 9.291 1.00 1.00

ATOM 826 C TYR A 115 -16.616 21.480 8.665 1.00 1.00

ATOM 827 O TYR A 115 -15.709 21.988 7.998 1.00 1.00

ATOM 828 CB TYR A 115 -18.755 22.707 8.238 1.00 1.00

ATOM 829 CG TYR A 115 -19.941 23.477 8.786 1.00 1.00

ATOM 830 CD1 TYR A 115 -20.360 24.667 8.189 1.00 1.00

ATOM 831 CD2 TYR A 115 -20.639 23.018 9.903 1.00 1.00

ATOM 832 CE1 TYR A 115 -21.455 25.372 8.684 1.00 1.00

ATOM 833 CE2 TYR A 115 -21.731 23.720 10.405 1.00 1.00

ATOM 834 CZ TYR A 115 -22.135 24.890 9.793 1.00 1.00

ATOM 835 OH TYR A 115 -23.215 25.572 10.294 1.00 1.00

ATOM 836 N ILE A 116 -16.697 20.180 8.919 1.00 1.00

ATOM 837 CA ILE A 116 -15.856 19.208 8.249 1.00 1.00

ATOM 838 C ILE A 116 -16.785 18.428 7.320 1.00 1.00

ATOM 839 O ILE A 116 -17.769 17.841 7.766 1.00 1.00

ATOM 840 CB ILE A 116 -15.125 18.285 9.247 1.00 1.00

ATOM 841 CG1 ILE A 116 -14.244 19.126 10.186 1.00 1.00

ATOM 842 CG2 ILE A 116 -14.308 17.223 8.498 1.00 1.00

ATOM 843 CD1 ILE A 116 -13.553 18.340 11.301 1.00 1.00

ATOM 844 N THR A 117 -16.478 18.453 6.027 1.00 1.00

ATOM 845 CA THR A 117 -17.430 17.993 5.021 1.00 1.00

ATOM 846 C THR A 117 -16.856 16.944 4.080 1.00 1.00

ATOM 847 O THR A 117 -15.689 17.015 3.690 1.00 1.00

ATOM 848 CB THR A 117 -17.948 19.174 4.165 1.00 1.00

ATOM 849 OG1 THR A 117 -16.836 19.932 3.689 1.00 1.00

ATOM 850 CG2 THR A 117 -18.861 20.092 4.975 1.00 1.00

ATOM 851 N PHE A 118 -17.700 15.988 3.699 1.00 1.00

ATOM 852 CA PHE A 118 -17.349 15.009 2.675 1.00 1.00

ATOM 853 C PHE A 118 -17.029 15.698 1.347 1.00 1.00

ATOM 854 O PHE A 118 -17.840 16.465 0.821 1.00 1.00

ATOM 855 CB PHE A 118 -18.483 13.993 2.493 1.00 1.00

ATOM 856 CG PHE A 118 -18.403 13.221 1.204 1.00 1.00

ATOM 857 CD1 PHE A 118 -17.368 12.313 0.983 1.00 1.00

ATOM 858 CD2 PHE A 118 -19.358 13.406 0.210 1.00 1.00

ATOM 859 CE1 PHE A 118 -17.287 11.602 -0.206 1.00 1.00

ATOM 860 CE2 PHE A 118 -19.285 12.693 -0.989 1.00 1.00

ATOM 861 CZ PHE A 118 -18.247 11.792 -1.196 1.00 1.00

ATOM 862 N ASP A 119 -15.841 15.418 0.815 1.00 1.00

ATOM 863 CA ASP A 119 -15.395 16.004 -0.450 1.00 1.00

ATOM 864 C ASP A 119 -15.404 14.950 -1.561 1.00 1.00

ATOM 865 O ASP A 119 -16.088 15.107 -2.566 1.00 1.00

ATOM 866 CB ASP A 119 -13.996 16.620 -0.284 1.00 1.00

ATOM 867 CG ASP A 119 -13.480 17.304 -1.551 1.00 1.00

ATOM 868 OD1 ASP A 119 -14.240 17.499 -2.525 1.00 1.00

ATOM 869 OD2 ASP A 119 -12.287 17.661 -1.569 1.00 1.00

ATOM 870 N GLU A 120 -14.648 13.875 -1.360 1.00 1.00

ATOM 871 CA GLU A 120 -14.507 12.826 -2.365 1.00 1.00

ATOM 872 C GLU A 120 -14.316 11.469 -1.696 1.00 1.00

ATOM 873 O GLU A 120 -13.770 11.387 -0.598 1.00 1.00

ATOM 874 CB GLU A 120 -13.312 13.136 -3.273 1.00 1.00

ATOM 875 CG GLU A 120 -13.398 12.531 -4.668 1.00 1.00

ATOM 876 CD GLU A 120 -12.481 13.222 -5.674 1.00 1.00

ATOM 877 OE1 GLU A 120 -11.446 13.791 -5.255 1.00 1.00

ATOM 878 OE2 GLU A 120 -12.801 13.192 -6.888 1.00 1.00

ATOM 879 N VAL A 121 -14.786 10.414 -2.356 1.00 1.00

ATOM 880 CA VAL A 121 -14.528 9.044 -1.920 1.00 1.00

ATOM 881 C VAL A 121 -13.950 8.235 -3.080 1.00 1.00

ATOM 882 O VAL A 121 -14.392 8.368 -4.222 1.00 1.00

ATOM 883 CB VAL A 121 -15.800 8.355 -1.313 1.00 1.00

ATOM 884 CG1 VAL A 121 -16.993 8.387 -2.286 1.00 1.00

ATOM 885 CG2 VAL A 121 -15.500 6.923 -0.863 1.00 1.00

ATOM 886 N GLY A 122 -12.937 7.426 -2.783 1.00 1.00

ATOM 887 CA GLY A 122 -12.387 6.494 -3.756 1.00 1.00

ATOM 888 C GLY A 122 -13.150 5.186 -3.705 1.00 1.00

ATOM 889 O GLY A 122 -14.222 5.058 -4.300 1.00 1.00

ATOM 890 N GLY A 123 -12.600 4.217 -2.979 1.00 1.00

ATOM 891 CA GLY A 123 -13.261 2.926 -2.810 1.00 1.00

ATOM 892 C GLY A 123 -13.375 2.480 -1.366 1.00 1.00

ATOM 893 O GLY A 123 -12.483 2.735 -0.567 1.00 1.00

ATOM 894 N PHE A 124 -14.497 1.836 -1.049 1.00 1.00

ATOM 895 CA PHE A 124 -14.733 1.161 0.227 1.00 1.00

ATOM 896 C PHE A 124 -14.984 -0.294 -0.138 1.00 1.00

ATOM 897 O PHE A 124 -16.048 -0.611 -0.666 1.00 1.00

ATOM 898 CB PHE A 124 -16.011 1.700 0.892 1.00 1.00

ATOM 899 CG PHE A 124 -15.783 2.676 2.014 1.00 1.00

ATOM 900 CD1 PHE A 124 -15.476 4.009 1.753 1.00 1.00

ATOM 901 CD2 PHE A 124 -15.939 2.275 3.340 1.00 1.00

ATOM 902 CE1 PHE A 124 -15.292 4.920 2.790 1.00 1.00

ATOM 903 CE2 PHE A 124 -15.765 3.177 4.390 1.00 1.00

ATOM 904 CZ PHE A 124 -15.441 4.505 4.112 1.00 1.00

ATOM 905 N ALA A 125 -14.027 -1.180 0.121 1.00 1.00

ATOM 906 CA ALA A 125 -14.181 -2.583 -0.273 1.00 1.00

ATOM 907 C ALA A 125 -14.137 -3.552 0.904 1.00 1.00

ATOM 908 O ALA A 125 -13.491 -3.282 1.916 1.00 1.00

ATOM 909 CB ALA A 125 -13.135 -2.964 -1.323 1.00 1.00

ATOM 910 N ASP A 126 -14.832 -4.678 0.765 1.00 1.00

ATOM 911 CA ASP A 126 -14.754 -5.751 1.759 1.00 1.00

ATOM 912 C ASP A 126 -13.566 -6.668 1.476 1.00 1.00

ATOM 913 O ASP A 126 -12.788 -6.411 0.548 1.00 1.00

ATOM 914 CB ASP A 126 -16.082 -6.531 1.867 1.00 1.00

ATOM 915 CG ASP A 126 -16.420 -7.340 0.609 1.00 1.00

ATOM 916 OD1 ASP A 126 -15.562 -7.536 -0.277 1.00 1.00

ATOM 917 OD2 ASP A 126 -17.575 -7.798 0.518 1.00 1.00

ATOM 918 N ASN A 127 -13.436 -7.736 2.260 1.00 1.00

ATOM 919 CA ASN A 127 -12.351 -8.701 2.079 1.00 1.00

ATOM 920 C ASN A 127 -12.382 -9.498 0.770 1.00 1.00

ATOM 921 O ASN A 127 -11.392 -10.132 0.416 1.00 1.00

ATOM 922 CB ASN A 127 -12.252 -9.646 3.280 1.00 1.00

ATOM 923 CG ASN A 127 -11.527 -9.019 4.449 1.00 1.00

ATOM 924 OD1 ASN A 127 -10.492 -8.370 4.276 1.00 1.00

ATOM 925 ND2 ASN A 127 -12.060 -9.212 5.653 1.00 1.00

ATOM 926 N ASP A 128 -13.512 -9.458 0.062 1.00 1.00

ATOM 927 CA ASP A 128 -13.612 -10.085 -1.265 1.00 1.00

ATOM 928 C ASP A 128 -13.403 -9.069 -2.389 1.00 1.00

ATOM 929 O ASP A 128 -13.613 -9.378 -3.564 1.00 1.00

ATOM 930 CB ASP A 128 -14.957 -10.797 -1.432 1.00 1.00

ATOM 931 CG ASP A 128 -15.024 -12.116 -0.674 1.00 1.00

ATOM 932 OD1 ASP A 128 -14.061 -12.457 0.041 1.00 1.00

ATOM 933 OD2 ASP A 128 -16.054 -12.813 -0.798 1.00 1.00

ATOM 934 N LEU A 129 -12.986 -7.862 -2.005 1.00 1.00

ATOM 935 CA LEU A 129 -12.761 -6.736 -2.923 1.00 1.00

ATOM 936 C LEU A 129 -14.049 -6.224 -3.571 1.00 1.00

ATOM 937 O LEU A 129 -14.009 -5.524 -4.587 1.00 1.00

ATOM 938 CB LEU A 129 -11.709 -7.073 -3.998 1.00 1.00

ATOM 939 CG LEU A 129 -10.308 -7.519 -3.571 1.00 1.00

ATOM 940 CD1 LEU A 129 -9.348 -7.364 -4.743 1.00 1.00

ATOM 941 CD2 LEU A 129 -9.803 -6.741 -2.379 1.00 1.00

ATOM 942 N VAL A 130 -15.186 -6.571 -2.977 1.00 1.00

ATOM 943 CA VAL A 130 -16.475 -6.066 -3.432 1.00 1.00

ATOM 944 C VAL A 130 -16.655 -4.655 -2.888 1.00 1.00

ATOM 945 O VAL A 130 -16.685 -4.448 -1.667 1.00 1.00

ATOM 946 CB VAL A 130 -17.651 -6.977 -2.992 1.00 1.00

ATOM 947 CG1 VAL A 130 -18.992 -6.409 -3.464 1.00 1.00

ATOM 948 CG2 VAL A 130 -17.459 -8.395 -3.520 1.00 1.00

ATOM 949 N GLU A 131 -16.755 -3.690 -3.801 1.00 1.00

ATOM 950 CA GLU A 131 -16.960 -2.284 -3.454 1.00 1.00

ATOM 951 C GLU A 131 -18.334 -2.067 -2.836 1.00 1.00

ATOM 952 O GLU A 131 -19.321 -2.636 -3.302 1.00 1.00

ATOM 953 CB GLU A 131 -16.811 -1.399 -4.697 1.00 1.00

ATOM 954 CG GLU A 131 -15.383 -1.288 -5.225 1.00 1.00

ATOM 955 CD GLU A 131 -14.572 -0.214 -4.528 1.00 1.00

ATOM 956 OE1 GLU A 131 -15.038 0.340 -3.512 1.00 1.00

ATOM 957 OE2 GLU A 131 -13.459 0.085 -5.008 1.00 1.00

ATOM 958 N GLN A 132 -18.389 -1.235 -1.804 1.00 1.00

ATOM 959 CA GLN A 132 -19.613 -1.016 -1.054 1.00 1.00

ATOM 960 C GLN A 132 -20.286 0.298 -1.429 1.00 1.00

ATOM 961 O GLN A 132 -19.629 1.255 -1.850 1.00 1.00

ATOM 962 CB GLN A 132 -19.306 -1.033 0.449 1.00 1.00

ATOM 963 CG GLN A 132 -18.658 -2.322 0.947 1.00 1.00

ATOM 964 CD GLN A 132 -19.629 -3.486 0.990 1.00 1.00

ATOM 965 OE1 GLN A 132 -20.667 -3.415 1.646 1.00 1.00

ATOM 966 NE2 GLN A 132 -19.293 -4.564 0.297 1.00 1.00

ATOM 967 N LYS A 133 -21.610 0.321 -1.289 1.00 1.00

ATOM 968 CA LYS A 133 -22.364 1.561 -1.326 1.00 1.00

ATOM 969 C LYS A 133 -21.980 2.319 -0.058 1.00 1.00

ATOM 970 O LYS A 133 -21.901 1.728 1.021 1.00 1.00

ATOM 971 CB LYS A 133 -23.862 1.264 -1.338 1.00 1.00

ATOM 972 CG LYS A 133 -24.697 2.225 -2.171 1.00 1.00

ATOM 973 CD LYS A 133 -26.099 1.669 -2.435 1.00 1.00

ATOM 974 CE LYS A 133 -26.077 0.505 -3.435 1.00 1.00

ATOM 975 NZ LYS A 133 -27.450 0.050 -3.807 1.00 1.00

ATOM 976 N VAL A 134 -21.706 3.611 -0.193 1.00 1.00

ATOM 977 CA VAL A 134 -21.279 4.418 0.951 1.00 1.00

ATOM 978 C VAL A 134 -21.918 5.808 0.936 1.00 1.00

ATOM 979 O VAL A 134 -22.017 6.446 -0.115 1.00 1.00

ATOM 980 CB VAL A 134 -19.716 4.472 1.064 1.00 1.00

ATOM 981 CG1 VAL A 134 -19.077 4.979 -0.228 1.00 1.00

ATOM 982 CG2 VAL A 134 -19.262 5.287 2.281 1.00 1.00

ATOM 983 N SER A 135 -22.389 6.253 2.099 1.00 1.00

ATOM 984 CA SER A 135 -22.933 7.599 2.244 1.00 1.00

ATOM 985 C SER A 135 -22.335 8.307 3.460 1.00 1.00

ATOM 986 O SER A 135 -21.944 7.667 4.440 1.00 1.00

ATOM 987 CB SER A 135 -24.465 7.582 2.306 1.00 1.00

ATOM 988 OG SER A 135 -24.934 6.811 3.396 1.00 1.00

ATOM 989 N PHE A 136 -22.262 9.633 3.379 1.00 1.00

ATOM 990 CA PHE A 136 -21.585 10.431 4.393 1.00 1.00

ATOM 991 C PHE A 136 -22.536 11.395 5.081 1.00 1.00

ATOM 992 O PHE A 136 -23.462 11.912 4.459 1.00 1.00

ATOM 993 CB PHE A 136 -20.425 11.210 3.766 1.00 1.00

ATOM 994 CG PHE A 136 -19.297 10.338 3.280 1.00 1.00

ATOM 995 CD1 PHE A 136 -19.380 9.681 2.051 1.00 1.00

ATOM 996 CD2 PHE A 136 -18.151 10.179 4.047 1.00 1.00

ATOM 997 CE1 PHE A 136 -18.339 8.872 1.605 1.00 1.00

ATOM 998 CE2 PHE A 136 -17.105 9.377 3.611 1.00 1.00

ATOM 999 CZ PHE A 136 -17.197 8.720 2.388 1.00 1.00

ATOM 1000 N ILE A 137 -22.303 11.611 6.372 1.00 1.00

ATOM 1001 CA ILE A 137 -23.021 12.611 7.152 1.00 1.00

ATOM 1002 C ILE A 137 -21.992 13.619 7.661 1.00 1.00

ATOM 1003 O ILE A 137 -21.074 13.257 8.396 1.00 1.00

ATOM 1004 CB ILE A 137 -23.799 11.984 8.339 1.00 1.00

ATOM 1005 CG1 ILE A 137 -24.829 10.962 7.835 1.00 1.00

ATOM 1006 CG2 ILE A 137 -24.477 13.070 9.177 1.00 1.00

ATOM 1007 CD1 ILE A 137 -25.422 10.084 8.928 1.00 1.00

ATOM 1008 N ASP A 138 -22.141 14.875 7.243 1.00 1.00

ATOM 1009 CA ASP A 138 -21.204 15.931 7.607 1.00 1.00

ATOM 1010 C ASP A 138 -21.381 16.394 9.055 1.00 1.00

ATOM 1011 O ASP A 138 -22.447 16.219 9.645 1.00 1.00

ATOM 1012 CB ASP A 138 -21.329 17.115 6.637 1.00 1.00

ATOM 1013 CG ASP A 138 -20.879 16.768 5.220 1.00 1.00

ATOM 1014 OD1 ASP A 138 -20.219 15.727 5.031 1.00 1.00

ATOM 1015 OD2 ASP A 138 -21.174 17.551 4.294 1.00 1.00

ATOM 1016 N GLY A 139 -20.324 16.983 9.614 1.00 1.00

ATOM 1017 CA GLY A 139 -20.358 17.509 10.974 1.00 1.00

ATOM 1018 C GLY A 139 -19.297 18.567 11.197 1.00 1.00

ATOM 1019 O GLY A 139 -19.075 19.430 10.338 1.00 1.00

ATOM 1020 N GLY A 140 -18.635 18.498 12.347 1.00 1.00

ATOM 1021 CA GLY A 140 -17.575 19.448 12.680 1.00 1.00

ATOM 1022 C GLY A 140 -17.788 20.154 14.008 1.00 1.00

ATOM 1023 O GLY A 140 -18.326 19.576 14.950 1.00 1.00

ATOM 1024 N VAL A 141 -17.375 21.417 14.069 1.00 1.00

ATOM 1025 CA VAL A 141 -17.278 22.137 15.338 1.00 1.00

ATOM 1026 C VAL A 141 -18.228 23.338 15.397 1.00 1.00

ATOM 1027 O VAL A 141 -18.346 24.095 14.427 1.00 1.00

ATOM 1028 CB VAL A 141 -15.809 22.584 15.607 1.00 1.00

ATOM 1029 CG1 VAL A 141 -15.664 23.217 16.989 1.00 1.00

ATOM 1030 CG2 VAL A 141 -14.852 21.394 15.469 1.00 1.00

ATOM 1031 N ASN A 142 -18.903 23.495 16.539 1.00 1.00

ATOM 1032 CA ASN A 142 -19.809 24.625 16.777 1.00 1.00

ATOM 1033 C ASN A 142 -20.743 24.868 15.592 1.00 1.00

ATOM 1034 O ASN A 142 -20.788 25.974 15.042 1.00 1.00

ATOM 1035 CB ASN A 142 -19.010 25.901 17.092 1.00 1.00

ATOM 1036 CG ASN A 142 -18.152 25.774 18.341 1.00 1.00

ATOM 1037 OD1 ASN A 142 -18.587 25.231 19.354 1.00 1.00

ATOM 1038 ND2 ASN A 142 -16.933 26.298 18.276 1.00 1.00

ATOM 1039 N VAL A 143 -21.488 23.834 15.210 1.00 1.00

ATOM 1040 CA VAL A 143 -22.252 23.845 13.956 1.00 1.00

ATOM 1041 C VAL A 143 -23.422 24.837 13.932 1.00 1.00

ATOM 1042 O VAL A 143 -23.884 25.222 12.858 1.00 1.00

ATOM 1043 CB VAL A 143 -22.746 22.417 13.540 1.00 1.00

ATOM 1044 CG1 VAL A 143 -21.553 21.473 13.311 1.00 1.00

ATOM 1045 CG2 VAL A 143 -23.732 21.837 14.556 1.00 1.00

ATOM 1046 N GLY A 144 -23.885 25.243 15.111 1.00 1.00

ATOM 1047 CA GLY A 144 -25.041 26.131 15.223 1.00 1.00

ATOM 1048 C GLY A 144 -24.716 27.585 15.511 1.00 1.00

ATOM 1049 O GLY A 144 -25.623 28.397 15.683 1.00 1.00

ATOM 1050 OXT GLY A 144 -23.507 27.919 15.535 1.00 1.00

TER

ATOM 1051 N GLY B 1 -1.476 2.526 -11.801 1.00 1.00

ATOM 1052 CA GLY B 1 -2.123 2.556 -10.492 1.00 1.00

ATOM 1053 C GLY B 1 -3.493 1.905 -10.464 1.00 1.00

ATOM 1054 O GLY B 1 -3.966 1.503 -9.405 1.00 1.00

ATOM 1055 N ASP B 2 -4.134 1.803 -11.625 1.00 1.00

ATOM 1056 CA ASP B 2 -5.453 1.183 -11.733 1.00 1.00

ATOM 1057 C ASP B 2 -5.328 -0.338 -11.839 1.00 1.00

ATOM 1058 O ASP B 2 -5.697 -0.937 -12.851 1.00 1.00

ATOM 1059 CB ASP B 2 -6.225 1.765 -12.927 1.00 1.00

ATOM 1060 CG ASP B 2 -7.627 1.182 -13.066 1.00 1.00

ATOM 1061 OD1 ASP B 2 -8.177 0.671 -12.069 1.00 1.00

ATOM 1062 OD2 ASP B 2 -8.183 1.234 -14.180 1.00 1.00

ATOM 1063 N VAL B 3 -4.828 -0.953 -10.772 1.00 1.00

ATOM 1064 CA VAL B 3 -4.519 -2.380 -10.761 1.00 1.00

ATOM 1065 C VAL B 3 -5.651 -3.253 -11.332 1.00 1.00

ATOM 1066 O VAL B 3 -5.414 -4.048 -12.239 1.00 1.00

ATOM 1067 CB VAL B 3 -4.102 -2.871 -9.348 1.00 1.00

ATOM 1068 CG1 VAL B 3 -3.684 -4.333 -9.391 1.00 1.00

ATOM 1069 CG2 VAL B 3 -2.964 -2.009 -8.785 1.00 1.00

ATOM 1070 N ASN B 4 -6.871 -3.086 -10.822 1.00 1.00

ATOM 1071 CA ASN B 4 -8.000 -3.931 -11.246 1.00 1.00

ATOM 1072 C ASN B 4 -8.636 -3.535 -12.582 1.00 1.00

ATOM 1073 O ASN B 4 -9.536 -4.221 -13.078 1.00 1.00

ATOM 1074 CB ASN B 4 -9.057 -4.050 -10.135 1.00 1.00

ATOM 1075 CG ASN B 4 -9.816 -2.751 -9.891 1.00 1.00

ATOM 1076 OD1 ASN B 4 -9.626 -1.758 -10.593 1.00 1.00

ATOM 1077 ND2 ASN B 4 -10.695 -2.763 -8.894 1.00 1.00

ATOM 1078 N GLY B 5 -8.174 -2.424 -13.149 1.00 1.00

ATOM 1079 CA GLY B 5 -8.609 -1.968 -14.467 1.00 1.00

ATOM 1080 C GLY B 5 -10.043 -1.482 -14.585 1.00 1.00

ATOM 1081 O GLY B 5 -10.626 -1.556 -15.666 1.00 1.00

ATOM 1082 N ASP B 6 -10.617 -0.978 -13.492 1.00 1.00

ATOM 1083 CA ASP B 6 -11.990 -0.456 -13.534 1.00 1.00

ATOM 1084 C ASP B 6 -12.080 1.028 -13.897 1.00 1.00

ATOM 1085 O ASP B 6 -13.169 1.616 -13.847 1.00 1.00

ATOM 1086 CB ASP B 6 -12.755 -0.751 -12.234 1.00 1.00

ATOM 1087 CG ASP B 6 -12.209 0.001 -11.036 1.00 1.00

ATOM 1088 OD1 ASP B 6 -11.224 0.755 -11.180 1.00 1.00

ATOM 1089 OD2 ASP B 6 -12.763 -0.176 -9.934 1.00 1.00

ATOM 1090 N GLY B 7 -10.943 1.625 -14.254 1.00 1.00

ATOM 1091 CA GLY B 7 -10.916 2.987 -14.788 1.00 1.00

ATOM 1092 C GLY B 7 -10.709 4.081 -13.759 1.00 1.00

ATOM 1093 O GLY B 7 -10.621 5.258 -14.112 1.00 1.00

ATOM 1094 N THR B 8 -10.641 3.698 -12.488 1.00 1.00

ATOM 1095 CA THR B 8 -10.364 4.646 -11.408 1.00 1.00

ATOM 1096 C THR B 8 -9.296 4.092 -10.468 1.00 1.00

ATOM 1097 O THR B 8 -9.219 2.880 -10.255 1.00 1.00

ATOM 1098 CB THR B 8 -11.654 5.052 -10.632 1.00 1.00

ATOM 1099 OG1 THR B 8 -11.337 6.048 -9.650 1.00 1.00

ATOM 1100 CG2 THR B 8 -12.295 3.854 -9.945 1.00 1.00

ATOM 1101 N ILE B 9 -8.463 4.981 -9.935 1.00 1.00

ATOM 1102 CA ILE B 9 -7.407 4.597 -8.999 1.00 1.00

ATOM 1103 C ILE B 9 -7.854 4.869 -7.566 1.00 1.00

ATOM 1104 O ILE B 9 -8.082 6.019 -7.186 1.00 1.00

ATOM 1105 CB ILE B 9 -6.082 5.346 -9.286 1.00 1.00

ATOM 1106 CG1 ILE B 9 -5.673 5.190 -10.756 1.00 1.00

ATOM 1107 CG2 ILE B 9 -4.968 4.853 -8.348 1.00 1.00

ATOM 1108 CD1 ILE B 9 -4.710 6.257 -11.242 1.00 1.00

ATOM 1109 N ASN B 10 -7.986 3.805 -6.780 1.00 1.00

ATOM 1110 CA ASN B 10 -8.398 3.915 -5.376 1.00 1.00

ATOM 1111 C ASN B 10 -7.790 2.835 -4.474 1.00 1.00

ATOM 1112 O ASN B 10 -6.981 2.015 -4.931 1.00 1.00

ATOM 1113 CB ASN B 10 -9.935 3.989 -5.234 1.00 1.00

ATOM 1114 CG ASN B 10 -10.654 2.765 -5.793 1.00 1.00

ATOM 1115 OD1 ASN B 10 -10.161 1.642 -5.714 1.00 1.00

ATOM 1116 ND2 ASN B 10 -11.845 2.985 -6.347 1.00 1.00

ATOM 1117 N SER B 11 -8.187 2.842 -3.202 1.00 1.00

ATOM 1118 CA SER B 11 -7.637 1.930 -2.197 1.00 1.00

ATOM 1119 C SER B 11 -7.915 0.446 -2.455 1.00 1.00

ATOM 1120 O SER B 11 -7.264 -0.415 -1.865 1.00 1.00

ATOM 1121 CB SER B 11 -8.092 2.327 -0.783 1.00 1.00

ATOM 1122 OG SER B 11 -9.505 2.382 -0.680 1.00 1.00

ATOM 1123 N THR B 12 -8.867 0.149 -3.336 1.00 1.00

ATOM 1124 CA THR B 12 -9.176 -1.244 -3.693 1.00 1.00

ATOM 1125 C THR B 12 -8.056 -1.812 -4.570 1.00 1.00

ATOM 1126 O THR B 12 -7.684 -2.985 -4.439 1.00 1.00

ATOM 1127 CB THR B 12 -10.566 -1.369 -4.361 1.00 1.00

ATOM 1128 OG1 THR B 12 -11.539 -0.713 -3.537 1.00 1.00

ATOM 1129 CG2 THR B 12 -10.977 -2.835 -4.541 1.00 1.00

ATOM 1130 N ASP B 13 -7.505 -0.964 -5.439 1.00 1.00

ATOM 1131 CA ASP B 13 -6.290 -1.288 -6.187 1.00 1.00

ATOM 1132 C ASP B 13 -5.110 -1.528 -5.250 1.00 1.00

ATOM 1133 O ASP B 13 -4.307 -2.430 -5.486 1.00 1.00

ATOM 1134 CB ASP B 13 -5.937 -0.169 -7.164 1.00 1.00

ATOM 1135 CG ASP B 13 -7.018 0.064 -8.188 1.00 1.00

ATOM 1136 OD1 ASP B 13 -7.353 -0.882 -8.936 1.00 1.00

ATOM 1137 OD2 ASP B 13 -7.538 1.197 -8.248 1.00 1.00

ATOM 1138 N LEU B 14 -5.015 -0.713 -4.199 1.00 1.00

ATOM 1139 CA LEU B 14 -3.973 -0.881 -3.181 1.00 1.00

ATOM 1140 C LEU B 14 -4.048 -2.245 -2.486 1.00 1.00

ATOM 1141 O LEU B 14 -3.032 -2.929 -2.360 1.00 1.00

ATOM 1142 CB LEU B 14 -3.992 0.265 -2.160 1.00 1.00

ATOM 1143 CG LEU B 14 -2.861 0.302 -1.123 1.00 1.00

ATOM 1144 CD1 LEU B 14 -1.475 0.307 -1.782 1.00 1.00

ATOM 1145 CD2 LEU B 14 -3.021 1.487 -0.201 1.00 1.00

ATOM 1146 N THR B 15 -5.245 -2.635 -2.042 1.00 1.00

ATOM 1147 CA THR B 15 -5.450 -3.959 -1.447 1.00 1.00

ATOM 1148 C THR B 15 -5.025 -5.066 -2.415 1.00 1.00

ATOM 1149 O THR B 15 -4.306 -5.996 -2.034 1.00 1.00

ATOM 1150 CB THR B 15 -6.911 -4.177 -1.006 1.00 1.00

ATOM 1151 OG1 THR B 15 -7.202 -3.315 0.094 1.00 1.00

ATOM 1152 CG2 THR B 15 -7.142 -5.629 -0.563 1.00 1.00

ATOM 1153 N MET B 16 -5.463 -4.944 -3.667 1.00 1.00

ATOM 1154 CA MET B 16 -5.102 -5.899 -4.710 1.00 1.00

ATOM 1155 C MET B 16 -3.591 -5.965 -4.912 1.00 1.00

ATOM 1156 O MET B 16 -3.027 -7.050 -5.023 1.00 1.00

ATOM 1157 CB MET B 16 -5.800 -5.551 -6.024 1.00 1.00

ATOM 1158 CG MET B 16 -6.046 -6.759 -6.892 1.00 1.00

ATOM 1159 SD MET B 16 -6.819 -6.370 -8.462 1.00 1.00

ATOM 1160 CE MET B 16 -7.613 -7.920 -8.818 1.00 1.00

ATOM 1161 N LEU B 17 -2.946 -4.801 -4.944 1.00 1.00

ATOM 1162 CA LEU B 17 -1.497 -4.716 -5.081 1.00 1.00

ATOM 1163 C LEU B 17 -0.751 -5.389 -3.922 1.00 1.00

ATOM 1164 O LEU B 17 0.152 -6.198 -4.149 1.00 1.00

ATOM 1165 CB LEU B 17 -1.044 -3.259 -5.246 1.00 1.00

ATOM 1166 CG LEU B 17 0.440 -2.990 -5.527 1.00 1.00

ATOM 1167 CD1 LEU B 17 0.953 -3.777 -6.729 1.00 1.00

ATOM 1168 CD2 LEU B 17 0.680 -1.498 -5.720 1.00 1.00

ATOM 1169 N LYS B 18 -1.124 -5.066 -2.684 1.00 1.00

ATOM 1170 CA LYS B 18 -0.412 -5.646 -1.542 1.00 1.00

ATOM 1171 C LYS B 18 -0.647 -7.151 -1.369 1.00 1.00

ATOM 1172 O LYS B 18 0.287 -7.884 -1.035 1.00 1.00

ATOM 1173 CB LYS B 18 -0.628 -4.853 -0.245 1.00 1.00

ATOM 1174 CG LYS B 18 -2.049 -4.533 0.136 1.00 1.00

ATOM 1175 CD LYS B 18 -2.113 -3.186 0.872 1.00 1.00

ATOM 1176 CE LYS B 18 -1.331 -3.180 2.182 1.00 1.00

ATOM 1177 NZ LYS B 18 -1.235 -1.793 2.756 1.00 1.00

ATOM 1178 N ARG B 19 -1.868 -7.607 -1.637 1.00 1.00

ATOM 1179 CA ARG B 19 -2.163 -9.045 -1.632 1.00 1.00

ATOM 1180 C ARG B 19 -1.408 -9.813 -2.725 1.00 1.00

ATOM 1181 O ARG B 19 -1.014 -10.960 -2.519 1.00 1.00

ATOM 1182 CB ARG B 19 -3.673 -9.306 -1.690 1.00 1.00

ATOM 1183 CG ARG B 19 -4.364 -8.989 -0.367 1.00 1.00

ATOM 1184 CD ARG B 19 -5.867 -9.216 -0.384 1.00 1.00

ATOM 1185 NE ARG B 19 -6.451 -8.811 0.896 1.00 1.00

ATOM 1186 CZ ARG B 19 -7.736 -8.911 1.220 1.00 1.00

ATOM 1187 NH1 ARG B 19 -8.617 -9.408 0.363 1.00 1.00

ATOM 1188 NH2 ARG B 19 -8.143 -8.510 2.420 1.00 1.00

ATOM 1189 N SER B 20 -1.180 -9.180 -3.870 1.00 1.00

ATOM 1190 CA SER B 20 -0.376 -9.802 -4.926 1.00 1.00

ATOM 1191 C SER B 20 1.116 -9.855 -4.559 1.00 1.00

ATOM 1192 O SER B 20 1.808 -10.819 -4.893 1.00 1.00

ATOM 1193 CB SER B 20 -0.581 -9.097 -6.269 1.00 1.00

ATOM 1194 OG SER B 20 0.192 -7.911 -6.352 1.00 1.00

ATOM 1195 N VAL B 21 1.604 -8.819 -3.880 1.00 1.00

ATOM 1196 CA VAL B 21 3.003 -8.773 -3.439 1.00 1.00

ATOM 1197 C VAL B 21 3.257 -9.834 -2.359 1.00 1.00

ATOM 1198 O VAL B 21 4.334 -10.433 -2.308 1.00 1.00

ATOM 1199 CB VAL B 21 3.429 -7.337 -2.989 1.00 1.00

ATOM 1200 CG1 VAL B 21 4.873 -7.302 -2.478 1.00 1.00

ATOM 1201 CG2 VAL B 21 3.267 -6.357 -4.141 1.00 1.00

ATOM 1202 N LEU B 22 2.251 -10.079 -1.525 1.00 1.00

ATOM 1203 CA LEU B 22 2.310 -11.144 -0.521 1.00 1.00

ATOM 1204 C LEU B 22 1.923 -12.506 -1.101 1.00 1.00

ATOM 1205 O LEU B 22 1.866 -13.503 -0.373 1.00 1.00

ATOM 1206 CB LEU B 22 1.414 -10.802 0.673 1.00 1.00

ATOM 1207 CG LEU B 22 1.878 -9.659 1.584 1.00 1.00

ATOM 1208 CD1 LEU B 22 0.701 -9.038 2.327 1.00 1.00

ATOM 1209 CD2 LEU B 22 2.959 -10.117 2.562 1.00 1.00

ATOM 1210 N ARG B 23 1.663 -12.531 -2.412 1.00 1.00

ATOM 1211 CA ARG B 23 1.219 -13.726 -3.143 1.00 1.00

ATOM 1212 C ARG B 23 -0.083 -14.348 -2.616 1.00 1.00

ATOM 1213 O ARG B 23 -0.319 -15.546 -2.775 1.00 1.00

ATOM 1214 CB ARG B 23 2.338 -14.780 -3.242 1.00 1.00

ATOM 1215 CG ARG B 23 3.600 -14.276 -3.918 1.00 1.00

ATOM 1216 CD ARG B 23 4.297 -15.377 -4.702 1.00 1.00

ATOM 1217 NE ARG B 23 5.587 -14.928 -5.231 1.00 1.00

ATOM 1218 CZ ARG B 23 5.754 -14.241 -6.360 1.00 1.00

ATOM 1219 NH1 ARG B 23 6.976 -13.886 -6.736 1.00 1.00

ATOM 1220 NH2 ARG B 23 4.713 -13.904 -7.115 1.00 1.00

ATOM 1221 N ALA B 24 -0.925 -13.519 -1.998 1.00 1.00

ATOM 1222 CA ALA B 24 -2.257 -13.942 -1.563 1.00 1.00

ATOM 1223 C ALA B 24 -3.219 -13.948 -2.750 1.00 1.00

ATOM 1224 O ALA B 24 -4.243 -14.631 -2.721 1.00 1.00

ATOM 1225 CB ALA B 24 -2.772 -13.043 -0.453 1.00 1.00

ATOM 1226 N ILE B 25 -2.888 -13.172 -3.782 1.00 1.00

ATOM 1227 CA ILE B 25 -3.599 -13.216 -5.059 1.00 1.00

ATOM 1228 C ILE B 25 -2.618 -13.212 -6.234 1.00 1.00

ATOM 1229 O ILE B 25 -1.435 -12.898 -6.078 1.00 1.00

ATOM 1230 CB ILE B 25 -4.619 -12.046 -5.240 1.00 1.00

ATOM 1231 CG1 ILE B 25 -3.913 -10.694 -5.304 1.00 1.00

ATOM 1232 CG2 ILE B 25 -5.704 -12.063 -4.158 1.00 1.00

ATOM 1233 CD1 ILE B 25 -4.502 -9.765 -6.335 1.00 1.00

ATOM 1234 N THR B 26 -3.120 -13.567 -7.412 1.00 1.00

ATOM 1235 CA THR B 26 -2.338 -13.488 -8.642 1.00 1.00

ATOM 1236 C THR B 26 -2.959 -12.471 -9.601 1.00 1.00

ATOM 1237 O THR B 26 -4.183 -12.420 -9.761 1.00 1.00

ATOM 1238 CB THR B 26 -2.145 -14.883 -9.307 1.00 1.00

ATOM 1239 OG1 THR B 26 -1.848 -14.725 -10.702 1.00 1.00

ATOM 1240 CG2 THR B 26 -3.386 -15.752 -9.150 1.00 1.00

ATOM 1241 N LEU B 27 -2.108 -11.650 -10.212 1.00 1.00

ATOM 1242 CA LEU B 27 -2.557 -10.614 -11.135 1.00 1.00

ATOM 1243 C LEU B 27 -2.600 -11.145 -12.563 1.00 1.00

ATOM 1244 O LEU B 27 -1.797 -12.005 -12.942 1.00 1.00

ATOM 1245 CB LEU B 27 -1.646 -9.377 -11.066 1.00 1.00

ATOM 1246 CG LEU B 27 -1.502 -8.589 -9.754 1.00 1.00

ATOM 1247 CD1 LEU B 27 -0.428 -7.512 -9.888 1.00 1.00

ATOM 1248 CD2 LEU B 27 -2.814 -7.968 -9.291 1.00 1.00

ATOM 1249 N THR B 28 -3.543 -10.634 -13.351 1.00 1.00

ATOM 1250 CA THR B 28 -3.556 -10.883 -14.787 1.00 1.00

ATOM 1251 C THR B 28 -2.441 -10.056 -15.405 1.00 1.00

ATOM 1252 O THR B 28 -1.989 -9.075 -14.810 1.00 1.00

ATOM 1253 CB THR B 28 -4.911 -10.512 -15.452 1.00 1.00

ATOM 1254 OG1 THR B 28 -5.037 -9.084 -15.540 1.00 1.00

ATOM 1255 CG2 THR B 28 -6.093 -11.097 -14.675 1.00 1.00

ATOM 1256 N ASP B 29 -2.006 -10.440 -16.598 1.00 1.00

ATOM 1257 CA ASP B 29 -0.909 -9.751 -17.266 1.00 1.00

ATOM 1258 C ASP B 29 -1.194 -8.265 -17.521 1.00 1.00

ATOM 1259 O ASP B 29 -0.295 -7.431 -17.370 1.00 1.00

ATOM 1260 CB ASP B 29 -0.517 -10.479 -18.553 1.00 1.00

ATOM 1261 CG ASP B 29 0.103 -11.849 -18.288 1.00 1.00

ATOM 1262 OD1 ASP B 29 0.703 -12.052 -17.210 1.00 1.00

ATOM 1263 OD2 ASP B 29 -0.010 -12.728 -19.168 1.00 1.00

ATOM 1264 N ASP B 30 -2.434 -7.934 -17.895 1.00 1.00

ATOM 1265 CA ASP B 30 -2.826 -6.530 -18.073 1.00 1.00

ATOM 1266 C ASP B 30 -2.716 -5.750 -16.757 1.00 1.00

ATOM 1267 O ASP B 30 -2.315 -4.589 -16.752 1.00 1.00

ATOM 1268 CB ASP B 30 -4.256 -6.407 -18.615 1.00 1.00

ATOM 1269 CG ASP B 30 -4.389 -6.849 -20.067 1.00 1.00

ATOM 1270 OD1 ASP B 30 -3.382 -7.178 -20.722 1.00 1.00

ATOM 1271 OD2 ASP B 30 -5.534 -6.863 -20.555 1.00 1.00

ATOM 1272 N ALA B 31 -3.089 -6.400 -15.655 1.00 1.00

ATOM 1273 CA ALA B 31 -3.027 -5.793 -14.324 1.00 1.00

ATOM 1274 C ALA B 31 -1.592 -5.546 -13.862 1.00 1.00

ATOM 1275 O ALA B 31 -1.329 -4.572 -13.156 1.00 1.00

ATOM 1276 CB ALA B 31 -3.769 -6.650 -13.312 1.00 1.00

ATOM 1277 N LYS B 32 -0.679 -6.435 -14.254 1.00 1.00

ATOM 1278 CA LYS B 32 0.740 -6.309 -13.902 1.00 1.00

ATOM 1279 C LYS B 32 1.345 -5.017 -14.457 1.00 1.00

ATOM 1280 O LYS B 32 2.121 -4.347 -13.771 1.00 1.00

ATOM 1281 CB LYS B 32 1.540 -7.524 -14.390 1.00 1.00

ATOM 1282 CG LYS B 32 1.214 -8.827 -13.674 1.00 1.00

ATOM 1283 CD LYS B 32 1.982 -9.991 -14.291 1.00 1.00

ATOM 1284 CE LYS B 32 1.433 -11.335 -13.820 1.00 1.00

ATOM 1285 NZ LYS B 32 2.135 -12.488 -14.463 1.00 1.00

ATOM 1286 N ALA B 33 0.976 -4.669 -15.688 1.00 1.00

ATOM 1287 CA ALA B 33 1.421 -3.416 -16.299 1.00 1.00

ATOM 1288 C ALA B 33 0.881 -2.194 -15.549 1.00 1.00

ATOM 1289 O ALA B 33 1.576 -1.189 -15.401 1.00 1.00

ATOM 1290 CB ALA B 33 1.028 -3.369 -17.770 1.00 1.00

ATOM 1291 N ARG B 34 -0.356 -2.288 -15.069 1.00 1.00

ATOM 1292 CA ARG B 34 -0.964 -1.198 -14.306 1.00 1.00

ATOM 1293 C ARG B 34 -0.405 -1.119 -12.877 1.00 1.00

ATOM 1294 O ARG B 34 -0.352 -0.040 -12.286 1.00 1.00

ATOM 1295 CB ARG B 34 -2.496 -1.329 -14.304 1.00 1.00

ATOM 1296 CG ARG B 34 -3.100 -1.422 -15.706 1.00 1.00

ATOM 1297 CD ARG B 34 -4.622 -1.381 -15.706 1.00 1.00

ATOM 1298 NE ARG B 34 -5.209 -2.544 -15.041 1.00 1.00

ATOM 1299 CZ ARG B 34 -5.705 -3.605 -15.669 1.00 1.00

ATOM 1300 NH1 ARG B 34 -5.701 -3.666 -16.999 1.00 1.00

ATOM 1301 NH2 ARG B 34 -6.214 -4.607 -14.967 1.00 1.00

ATOM 1302 N ALA B 35 0.030 -2.268 -12.352 1.00 1.00

ATOM 1303 CA ALA B 35 0.542 -2.394 -10.976 1.00 1.00

ATOM 1304 C ALA B 35 1.959 -1.850 -10.785 1.00 1.00

ATOM 1305 O ALA B 35 2.304 -1.365 -9.702 1.00 1.00

ATOM 1306 CB ALA B 35 0.477 -3.845 -10.521 1.00 1.00

ATOM 1307 N ASP B 36 2.779 -1.946 -11.831 1.00 1.00

ATOM 1308 CA ASP B 36 4.160 -1.466 -11.788 1.00 1.00

ATOM 1309 C ASP B 36 4.178 0.056 -11.889 1.00 1.00

ATOM 1310 O ASP B 36 4.510 0.623 -12.932 1.00 1.00

ATOM 1311 CB ASP B 36 4.985 -2.101 -12.912 1.00 1.00

ATOM 1312 CG ASP B 36 6.475 -1.807 -12.790 1.00 1.00

ATOM 1313 OD1 ASP B 36 6.900 -1.211 -11.773 1.00 1.00

ATOM 1314 OD2 ASP B 36 7.226 -2.175 -13.715 1.00 1.00

ATOM 1315 N VAL B 37 3.828 0.705 -10.780 1.00 1.00

ATOM 1316 CA VAL B 37 3.585 2.148 -10.760 1.00 1.00

ATOM 1317 C VAL B 37 4.836 3.015 -10.940 1.00 1.00

ATOM 1318 O VAL B 37 4.733 4.144 -11.424 1.00 1.00

ATOM 1319 CB VAL B 37 2.787 2.584 -9.504 1.00 1.00

ATOM 1320 CG1 VAL B 37 1.357 2.070 -9.587 1.00 1.00

ATOM 1321 CG2 VAL B 37 3.455 2.094 -8.225 1.00 1.00

ATOM 1322 N ASP B 38 6.004 2.490 -10.559 1.00 1.00

ATOM 1323 CA ASP B 38 7.270 3.203 -10.774 1.00 1.00

ATOM 1324 C ASP B 38 7.993 2.761 -12.056 1.00 1.00

ATOM 1325 O ASP B 38 9.068 3.278 -12.385 1.00 1.00

ATOM 1326 CB ASP B 38 8.188 3.136 -9.539 1.00 1.00

ATOM 1327 CG ASP B 38 8.895 4.446 -9.245 1.00 1.00

ATOM 1328 OD1 ASP B 38 8.284 5.525 -9.390 1.00 1.00

ATOM 1329 OD2 ASP B 38 10.082 4.392 -8.858 1.00 1.00

ATOM 1330 N LYS B 39 7.384 1.797 -12.751 1.00 1.00

ATOM 1331 CA LYS B 39 7.796 1.340 -14.090 1.00 1.00

ATOM 1332 C LYS B 39 9.240 0.833 -14.162 1.00 1.00

ATOM 1333 O LYS B 39 9.938 1.061 -15.152 1.00 1.00

ATOM 1334 CB LYS B 39 7.550 2.434 -15.140 1.00 1.00

ATOM 1335 CG LYS B 39 6.191 3.126 -15.013 1.00 1.00

ATOM 1336 CD LYS B 39 6.046 4.275 -15.994 1.00 1.00

ATOM 1337 CE LYS B 39 5.430 3.811 -17.305 1.00 1.00

ATOM 1338 NZ LYS B 39 4.944 4.963 -18.120 1.00 1.00

ATOM 1339 N ASN B 40 9.678 0.134 -13.118 1.00 1.00

ATOM 1340 CA ASN B 40 11.042 -0.400 -13.081 1.00 1.00

ATOM 1341 C ASN B 40 11.148 -1.871 -13.506 1.00 1.00

ATOM 1342 O ASN B 40 12.246 -2.434 -13.540 1.00 1.00

ATOM 1343 CB ASN B 40 11.678 -0.167 -11.704 1.00 1.00

ATOM 1344 CG ASN B 40 10.980 -0.928 -10.591 1.00 1.00

ATOM 1345 OD1 ASN B 40 9.998 -1.644 -10.815 1.00 1.00

ATOM 1346 ND2 ASN B 40 11.486 -0.774 -9.374 1.00 1.00

ATOM 1347 N GLY B 41 10.007 -2.483 -13.821 1.00 1.00

ATOM 1348 CA GLY B 41 9.970 -3.851 -14.335 1.00 1.00

ATOM 1349 C GLY B 41 9.613 -4.914 -13.314 1.00 1.00

ATOM 1350 O GLY B 41 9.515 -6.094 -13.654 1.00 1.00

ATOM 1351 N SER B 42 9.416 -4.493 -12.066 1.00 1.00

ATOM 1352 CA SER B 42 9.093 -5.400 -10.967 1.00 1.00

ATOM 1353 C SER B 42 7.882 -4.917 -10.170 1.00 1.00

ATOM 1354 O SER B 42 7.622 -3.716 -10.086 1.00 1.00

ATOM 1355 CB SER B 42 10.297 -5.545 -10.028 1.00 1.00

ATOM 1356 OG SER B 42 11.386 -6.178 -10.684 1.00 1.00

ATOM 1357 N ILE B 43 7.147 -5.858 -9.583 1.00 1.00

ATOM 1358 CA ILE B 43 6.061 -5.530 -8.666 1.00 1.00

ATOM 1359 C ILE B 43 6.502 -5.874 -7.250 1.00 1.00

ATOM 1360 O ILE B 43 6.727 -7.042 -6.938 1.00 1.00

ATOM 1361 CB ILE B 43 4.755 -6.308 -8.985 1.00 1.00

ATOM 1362 CG1 ILE B 43 4.442 -6.302 -10.490 1.00 1.00

ATOM 1363 CG2 ILE B 43 3.596 -5.803 -8.121 1.00 1.00

ATOM 1364 CD1 ILE B 43 4.115 -4.960 -11.070 1.00 1.00

ATOM 1365 N ASN B 44 6.616 -4.859 -6.400 1.00 1.00

ATOM 1366 CA ASN B 44 7.115 -5.057 -5.039 1.00 1.00

ATOM 1367 C ASN B 44 6.561 -4.060 -4.025 1.00 1.00

ATOM 1368 O ASN B 44 5.660 -3.277 -4.339 1.00 1.00

ATOM 1369 CB ASN B 44 8.654 -5.054 -5.031 1.00 1.00

ATOM 1370 CG ASN B 44 9.249 -3.797 -5.638 1.00 1.00

ATOM 1371 OD1 ASN B 44 8.641 -2.723 -5.618 1.00 1.00

ATOM 1372 ND2 ASN B 44 10.458 -3.922 -6.177 1.00 1.00

ATOM 1373 N SER B 45 7.113 -4.095 -2.812 1.00 1.00

ATOM 1374 CA SER B 45 6.718 -3.191 -1.724 1.00 1.00

ATOM 1375 C SER B 45 6.862 -1.710 -2.094 1.00 1.00

ATOM 1376 O SER B 45 6.079 -0.875 -1.641 1.00 1.00

ATOM 1377 CB SER B 45 7.515 -3.506 -0.463 1.00 1.00

ATOM 1378 OG SER B 45 7.199 -4.796 0.037 1.00 1.00

ATOM 1379 N THR B 46 7.860 -1.391 -2.917 1.00 1.00

ATOM 1380 CA THR B 46 8.068 -0.017 -3.383 1.00 1.00

ATOM 1381 C THR B 46 6.887 0.497 -4.218 1.00 1.00

ATOM 1382 O THR B 46 6.542 1.678 -4.147 1.00 1.00

ATOM 1383 CB THR B 46 9.359 0.093 -4.215 1.00 1.00

ATOM 1384 OG1 THR B 46 10.476 -0.343 -3.429 1.00 1.00

ATOM 1385 CG2 THR B 46 9.593 1.531 -4.648 1.00 1.00

ATOM 1386 N ASP B 47 6.270 -0.391 -4.998 1.00 1.00

ATOM 1387 CA ASP B 47 5.043 -0.050 -5.730 1.00 1.00

ATOM 1388 C ASP B 47 3.868 0.218 -4.793 1.00 1.00

ATOM 1389 O ASP B 47 3.084 1.149 -5.016 1.00 1.00

ATOM 1390 CB ASP B 47 4.675 -1.154 -6.723 1.00 1.00

ATOM 1391 CG ASP B 47 5.670 -1.272 -7.852 1.00 1.00

ATOM 1392 OD1 ASP B 47 5.835 -0.303 -8.624 1.00 1.00

ATOM 1393 OD2 ASP B 47 6.299 -2.338 -7.977 1.00 1.00

ATOM 1394 N VAL B 48 3.755 -0.604 -3.751 1.00 1.00

ATOM 1395 CA VAL B 48 2.715 -0.449 -2.733 1.00 1.00

ATOM 1396 C VAL B 48 2.810 0.937 -2.092 1.00 1.00

ATOM 1397 O VAL B 48 1.806 1.646 -1.972 1.00 1.00

ATOM 1398 CB VAL B 48 2.808 -1.560 -1.655 1.00 1.00

ATOM 1399 CG1 VAL B 48 1.909 -1.247 -0.461 1.00 1.00

ATOM 1400 CG2 VAL B 48 2.455 -2.923 -2.253 1.00 1.00

ATOM 1401 N LEU B 49 4.025 1.320 -1.713 1.00 1.00

ATOM 1402 CA LEU B 49 4.274 2.591 -1.046 1.00 1.00

ATOM 1403 C LEU B 49 4.044 3.797 -1.958 1.00 1.00

ATOM 1404 O LEU B 49 3.527 4.825 -1.513 1.00 1.00

ATOM 1405 CB LEU B 49 5.680 2.613 -0.438 1.00 1.00

ATOM 1406 CG LEU B 49 5.903 1.576 0.678 1.00 1.00

ATOM 1407 CD1 LEU B 49 7.372 1.447 1.006 1.00 1.00

ATOM 1408 CD2 LEU B 49 5.093 1.900 1.934 1.00 1.00

ATOM 1409 N LEU B 50 4.412 3.659 -3.233 1.00 1.00

ATOM 1410 CA LEU B 50 4.170 4.711 -4.214 1.00 1.00

ATOM 1411 C LEU B 50 2.672 4.926 -4.448 1.00 1.00

ATOM 1412 O LEU B 50 2.199 6.066 -4.486 1.00 1.00

ATOM 1413 CB LEU B 50 4.896 4.425 -5.531 1.00 1.00

ATOM 1414 CG LEU B 50 4.800 5.516 -6.611 1.00 1.00

ATOM 1415 CD1 LEU B 50 5.350 6.840 -6.092 1.00 1.00

ATOM 1416 CD2 LEU B 50 5.538 5.103 -7.862 1.00 1.00

ATOM 1417 N LEU B 51 1.929 3.831 -4.594 1.00 1.00

ATOM 1418 CA LEU B 51 0.479 3.926 -4.758 1.00 1.00

ATOM 1419 C LEU B 51 -0.189 4.547 -3.528 1.00 1.00

ATOM 1420 O LEU B 51 -1.124 5.339 -3.663 1.00 1.00

ATOM 1421 CB LEU B 51 -0.138 2.559 -5.104 1.00 1.00

ATOM 1422 CG LEU B 51 -1.656 2.526 -5.365 1.00 1.00

ATOM 1423 CD1 LEU B 51 -2.081 3.534 -6.440 1.00 1.00

ATOM 1424 CD2 LEU B 51 -2.138 1.132 -5.720 1.00 1.00

ATOM 1425 N SER B 52 0.301 4.193 -2.338 1.00 1.00

ATOM 1426 CA SER B 52 -0.201 4.765 -1.082 1.00 1.00

ATOM 1427 C SER B 52 0.010 6.277 -1.009 1.00 1.00

ATOM 1428 O SER B 52 -0.878 7.011 -0.575 1.00 1.00

ATOM 1429 CB SER B 52 0.451 4.093 0.124 1.00 1.00

ATOM 1430 OG SER B 52 0.105 2.727 0.182 1.00 1.00

ATOM 1431 N ARG B 53 1.192 6.729 -1.425 1.00 1.00

ATOM 1432 CA ARG B 53 1.516 8.153 -1.456 1.00 1.00

ATOM 1433 C ARG B 53 0.603 8.909 -2.416 1.00 1.00

ATOM 1434 O ARG B 53 0.183 10.029 -2.124 1.00 1.00

ATOM 1435 CB ARG B 53 2.984 8.374 -1.843 1.00 1.00

ATOM 1436 CG ARG B 53 3.995 8.097 -0.730 1.00 1.00

ATOM 1437 CD ARG B 53 5.298 8.869 -0.966 1.00 1.00

ATOM 1438 NE ARG B 53 5.844 8.626 -2.301 1.00 1.00

ATOM 1439 CZ ARG B 53 6.427 9.557 -3.060 1.00 1.00

ATOM 1440 NH1 ARG B 53 6.540 10.802 -2.627 1.00 1.00

ATOM 1441 NH2 ARG B 53 6.888 9.241 -4.262 1.00 1.00

ATOM 1442 N TYR B 54 0.298 8.283 -3.553 1.00 1.00

ATOM 1443 CA TYR B 54 -0.615 8.854 -4.551 1.00 1.00

ATOM 1444 C TYR B 54 -2.025 9.000 -3.985 1.00 1.00

ATOM 1445 O TYR B 54 -2.666 10.039 -4.161 1.00 1.00

ATOM 1446 CB TYR B 54 -0.621 8.012 -5.837 1.00 1.00

ATOM 1447 CG TYR B 54 -1.629 8.469 -6.880 1.00 1.00

ATOM 1448 CD1 TYR B 54 -1.310 9.467 -7.801 1.00 1.00

ATOM 1449 CD2 TYR B 54 -2.908 7.905 -6.937 1.00 1.00

ATOM 1450 CE1 TYR B 54 -2.236 9.891 -8.755 1.00 1.00

ATOM 1451 CE2 TYR B 54 -3.840 8.323 -7.885 1.00 1.00

ATOM 1452 CZ TYR B 54 -3.496 9.314 -8.788 1.00 1.00

ATOM 1453 OH TYR B 54 -4.417 9.728 -9.725 1.00 1.00

ATOM 1454 N LEU B 55 -2.501 7.960 -3.301 1.00 1.00

ATOM 1455 CA LEU B 55 -3.840 7.973 -2.703 1.00 1.00

ATOM 1456 C LEU B 55 -3.945 8.970 -1.551 1.00 1.00

ATOM 1457 O LEU B 55 -5.010 9.548 -1.319 1.00 1.00

ATOM 1458 CB LEU B 55 -4.262 6.565 -2.264 1.00 1.00

ATOM 1459 CG LEU B 55 -4.370 5.530 -3.395 1.00 1.00

ATOM 1460 CD1 LEU B 55 -4.555 4.129 -2.845 1.00 1.00

ATOM 1461 CD2 LEU B 55 -5.484 5.875 -4.382 1.00 1.00

ATOM 1462 N LEU B 56 -2.839 9.183 -0.844 1.00 1.00

ATOM 1463 CA LEU B 56 -2.778 10.198 0.212 1.00 1.00

ATOM 1464 C LEU B 56 -2.554 11.604 -0.348 1.00 1.00

ATOM 1465 O LEU B 56 -2.562 12.588 0.402 1.00 1.00

ATOM 1466 CB LEU B 56 -1.717 9.840 1.254 1.00 1.00

ATOM 1467 CG LEU B 56 -2.136 8.699 2.187 1.00 1.00

ATOM 1468 CD1 LEU B 56 -0.920 8.039 2.813 1.00 1.00

ATOM 1469 CD2 LEU B 56 -3.108 9.178 3.253 1.00 1.00

ATOM 1470 OXT LEU B 56 -2.394 11.720 -1.587 1.00 1.00

ATOM 1471 CA CA C 160 -9.146 0.590 -9.973 1.00 1.00

ATOM 1472 CA CA D 161 7.879 -1.538 -9.667 1.00 1.00

TER

ENDMDL

MODEL 5

ATOM 0 N GLY A 5 -26.382 0.617 3.011 1.00 1.00

ATOM 1 CA GLY A 5 -24.952 0.189 3.012 1.00 1.00

ATOM 2 C GLY A 5 -24.188 0.716 4.211 1.00 1.00

ATOM 3 O GLY A 5 -24.665 0.631 5.346 1.00 1.00

ATOM 4 N VAL A 6 -23.001 1.261 3.955 1.00 1.00

ATOM 5 CA VAL A 6 -22.150 1.819 5.004 1.00 1.00

ATOM 6 C VAL A 6 -22.351 3.331 5.108 1.00 1.00

ATOM 7 O VAL A 6 -22.383 4.033 4.095 1.00 1.00

ATOM 8 CB VAL A 6 -20.651 1.494 4.765 1.00 1.00

ATOM 9 CG1 VAL A 6 -19.804 1.881 5.975 1.00 1.00

ATOM 10 CG2 VAL A 6 -20.459 0.014 4.442 1.00 1.00

ATOM 11 N VAL A 7 -22.501 3.818 6.339 1.00 1.00

ATOM 12 CA VAL A 7 -22.658 5.247 6.598 1.00 1.00

ATOM 13 C VAL A 7 -21.448 5.781 7.362 1.00 1.00

ATOM 14 O VAL A 7 -21.121 5.296 8.449 1.00 1.00

ATOM 15 CB VAL A 7 -23.965 5.555 7.384 1.00 1.00

ATOM 16 CG1 VAL A 7 -24.147 7.056 7.568 1.00 1.00

ATOM 17 CG2 VAL A 7 -25.181 4.957 6.678 1.00 1.00

ATOM 18 N VAL A 8 -20.779 6.771 6.778 1.00 1.00

ATOM 19 CA VAL A 8 -19.646 7.428 7.427 1.00 1.00

ATOM 20 C VAL A 8 -20.111 8.746 8.048 1.00 1.00

ATOM 21 O VAL A 8 -20.573 9.648 7.346 1.00 1.00

ATOM 22 CB VAL A 8 -18.471 7.672 6.448 1.00 1.00

ATOM 23 CG1 VAL A 8 -17.296 8.328 7.164 1.00 1.00

ATOM 24 CG2 VAL A 8 -18.031 6.365 5.795 1.00 1.00

ATOM 25 N GLU A 9 -19.990 8.840 9.370 1.00 1.00

ATOM 26 CA GLU A 9 -20.486 9.995 10.110 1.00 1.00

ATOM 27 C GLU A 9 -19.352 10.862 10.640 1.00 1.00

ATOM 28 O GLU A 9 -18.575 10.427 11.494 1.00 1.00

ATOM 29 CB GLU A 9 -21.376 9.544 11.273 1.00 1.00

ATOM 30 CG GLU A 9 -22.802 9.193 10.880 1.00 1.00

ATOM 31 CD GLU A 9 -23.518 8.343 11.918 1.00 1.00

ATOM 32 OE1 GLU A 9 -22.960 8.120 13.016 1.00 1.00

ATOM 33 OE2 GLU A 9 -24.648 7.891 11.632 1.00 1.00

ATOM 34 N ILE A 10 -19.257 12.084 10.125 1.00 1.00

ATOM 35 CA ILE A 10 -18.360 13.082 10.695 1.00 1.00

ATOM 36 C ILE A 10 -19.049 13.666 11.923 1.00 1.00

ATOM 37 O ILE A 10 -20.124 14.261 11.818 1.00 1.00

ATOM 38 CB ILE A 10 -18.010 14.191 9.670 1.00 1.00

ATOM 39 CG1 ILE A 10 -17.315 13.592 8.442 1.00 1.00

ATOM 40 CG2 ILE A 10 -17.127 15.263 10.315 1.00 1.00

ATOM 41 CD1 ILE A 10 -17.570 14.348 7.147 1.00 1.00

ATOM 42 N GLY A 11 -18.427 13.472 13.085 1.00 1.00

ATOM 43 CA GLY A 11 -18.997 13.888 14.355 1.00 1.00

ATOM 44 C GLY A 11 -19.236 15.381 14.481 1.00 1.00

ATOM 45 O GLY A 11 -18.627 16.184 13.768 1.00 1.00

ATOM 46 N LYS A 12 -20.137 15.742 15.390 1.00 1.00

ATOM 47 CA LYS A 12 -20.441 17.138 15.681 1.00 1.00

ATOM 48 C LYS A 12 -20.135 17.435 17.146 1.00 1.00

ATOM 49 O LYS A 12 -20.752 16.861 18.045 1.00 1.00

ATOM 50 CB LYS A 12 -21.905 17.452 15.363 1.00 1.00

ATOM 51 CG LYS A 12 -22.193 17.629 13.884 1.00 1.00

ATOM 52 CD LYS A 12 -23.679 17.769 13.617 1.00 1.00

ATOM 53 CE LYS A 12 -24.036 17.288 12.221 1.00 1.00

ATOM 54 NZ LYS A 12 -25.219 18.006 11.666 1.00 1.00

ATOM 55 N VAL A 13 -19.169 18.321 17.369 1.00 1.00

ATOM 56 CA VAL A 13 -18.741 18.690 18.719 1.00 1.00

ATOM 57 C VAL A 13 -18.770 20.206 18.923 1.00 1.00

ATOM 58 O VAL A 13 -18.858 20.970 17.958 1.00 1.00

ATOM 59 CB VAL A 13 -17.322 18.141 19.059 1.00 1.00

ATOM 60 CG1 VAL A 13 -17.336 16.620 19.168 1.00 1.00

ATOM 61 CG2 VAL A 13 -16.283 18.610 18.037 1.00 1.00

ATOM 62 N THR A 14 -18.711 20.632 20.183 1.00 1.00

ATOM 63 CA THR A 14 -18.613 22.052 20.522 1.00 1.00

ATOM 64 C THR A 14 -17.377 22.309 21.379 1.00 1.00

ATOM 65 O THR A 14 -16.962 21.448 22.158 1.00 1.00

ATOM 66 CB THR A 14 -19.884 22.553 21.251 1.00 1.00

ATOM 67 OG1 THR A 14 -20.230 21.649 22.307 1.00 1.00

ATOM 68 CG2 THR A 14 -21.099 22.514 20.329 1.00 1.00

ATOM 69 N GLY A 15 -16.796 23.496 21.232 1.00 1.00

ATOM 70 CA GLY A 15 -15.604 23.865 21.974 1.00 1.00

ATOM 71 C GLY A 15 -15.295 25.348 21.925 1.00 1.00

ATOM 72 O GLY A 15 -15.709 26.051 20.999 1.00 1.00

ATOM 73 N SER A 16 -14.566 25.819 22.934 1.00 1.00

ATOM 74 CA SER A 16 -14.172 27.220 23.026 1.00 1.00

ATOM 75 C SER A 16 -12.972 27.530 22.134 1.00 1.00

ATOM 76 O SER A 16 -12.187 26.637 21.798 1.00 1.00

ATOM 77 CB SER A 16 -13.848 27.589 24.477 1.00 1.00

ATOM 78 OG SER A 16 -14.997 27.485 25.301 1.00 1.00

ATOM 79 N VAL A 17 -12.843 28.798 21.752 1.00 1.00

ATOM 80 CA VAL A 17 -11.699 29.275 20.979 1.00 1.00

ATOM 81 C VAL A 17 -10.420 29.126 21.807 1.00 1.00

ATOM 82 O VAL A 17 -10.384 29.506 22.980 1.00 1.00

ATOM 83 CB VAL A 17 -11.887 30.751 20.529 1.00 1.00

ATOM 84 CG1 VAL A 17 -10.743 31.207 19.623 1.00 1.00

ATOM 85 CG2 VAL A 17 -13.225 30.939 19.822 1.00 1.00

ATOM 86 N GLY A 18 -9.387 28.551 21.195 1.00 1.00

ATOM 87 CA GLY A 18 -8.115 28.337 21.861 1.00 1.00

ATOM 88 C GLY A 18 -7.946 26.944 22.445 1.00 1.00

ATOM 89 O GLY A 18 -6.852 26.589 22.889 1.00 1.00

ATOM 90 N THR A 19 -9.024 26.163 22.457 1.00 1.00

ATOM 91 CA THR A 19 -8.972 24.788 22.955 1.00 1.00

ATOM 92 C THR A 19 -8.813 23.791 21.809 1.00 1.00

ATOM 93 O THR A 19 -9.215 24.064 20.677 1.00 1.00

ATOM 94 CB THR A 19 -10.228 24.435 23.796 1.00 1.00

ATOM 95 OG1 THR A 19 -11.386 24.399 22.951 1.00 1.00

ATOM 96 CG2 THR A 19 -10.549 25.534 24.807 1.00 1.00

ATOM 97 N THR A 20 -8.199 22.643 22.118 1.00 1.00

ATOM 98 CA THR A 20 -8.110 21.561 21.126 1.00 1.00

ATOM 99 C THR A 20 -9.226 20.537 21.391 1.00 1.00

ATOM 100 O THR A 20 -9.376 20.023 22.507 1.00 1.00

ATOM 101 CB THR A 20 -6.735 20.882 21.230 1.00 1.00

ATOM 102 OG1 THR A 20 -5.700 21.858 21.182 1.00 1.00

ATOM 103 CG2 THR A 20 -6.469 19.888 20.102 1.00 1.00

ATOM 104 N VAL A 21 -9.994 20.261 20.347 1.00 1.00

ATOM 105 CA VAL A 21 -11.122 19.309 20.432 1.00 1.00

ATOM 106 C VAL A 21 -10.900 18.113 19.505 1.00 1.00

ATOM 107 O VAL A 21 -10.069 18.151 18.588 1.00 1.00

ATOM 108 CB VAL A 21 -12.434 19.994 20.031 1.00 1.00

ATOM 109 CG1 VAL A 21 -12.853 21.090 21.014 1.00 1.00

ATOM 110 CG2 VAL A 21 -12.370 20.667 18.655 1.00 1.00

ATOM 111 N GLU A 22 -11.660 17.073 19.780 1.00 1.00

ATOM 112 CA GLU A 22 -11.597 15.833 19.008 1.00 1.00

ATOM 113 C GLU A 22 -12.903 15.602 18.262 1.00 1.00

ATOM 114 O GLU A 22 -13.985 15.572 18.861 1.00 1.00

ATOM 115 CB GLU A 22 -11.336 14.654 19.937 1.00 1.00

ATOM 116 CG GLU A 22 -9.968 14.009 19.761 1.00 1.00

ATOM 117 CD GLU A 22 -9.575 13.137 20.938 1.00 1.00

ATOM 118 OE1 GLU A 22 -10.196 12.069 21.121 1.00 1.00

ATOM 119 OE2 GLU A 22 -8.644 13.522 21.678 1.00 1.00

ATOM 120 N ILE A 23 -12.767 15.448 16.961 1.00 1.00

ATOM 121 CA ILE A 23 -13.914 15.193 16.083 1.00 1.00

ATOM 122 C ILE A 23 -13.847 13.758 15.564 1.00 1.00

ATOM 123 O ILE A 23 -12.950 13.401 14.789 1.00 1.00

ATOM 124 CB ILE A 23 -13.918 16.147 14.886 1.00 1.00

ATOM 125 CG1 ILE A 23 -13.774 17.617 15.290 1.00 1.00

ATOM 126 CG2 ILE A 23 -15.215 16.057 14.073 1.00 1.00

ATOM 127 CD1 ILE A 23 -12.458 18.242 14.820 1.00 1.00

ATOM 128 N PRO A 24 -14.713 12.914 16.117 1.00 1.00

ATOM 129 CA PRO A 24 -14.730 11.494 15.747 1.00 1.00

ATOM 130 C PRO A 24 -15.326 11.272 14.360 1.00 1.00

ATOM 131 O PRO A 24 -16.227 12.002 13.943 1.00 1.00

ATOM 132 CB PRO A 24 -15.617 10.858 16.827 1.00 1.00

ATOM 133 CG PRO A 24 -16.506 11.961 17.304 1.00 1.00

ATOM 134 CD PRO A 24 -15.767 13.249 17.095 1.00 1.00

ATOM 135 N VAL A 25 -14.793 10.279 13.652 1.00 1.00

ATOM 136 CA VAL A 25 -15.353 9.833 12.381 1.00 1.00

ATOM 137 C VAL A 25 -15.895 8.422 12.596 1.00 1.00

ATOM 138 O VAL A 25 -15.135 7.497 12.885 1.00 1.00

ATOM 139 CB VAL A 25 -14.297 9.845 11.244 1.00 1.00

ATOM 140 CG1 VAL A 25 -14.908 9.376 9.929 1.00 1.00

ATOM 141 CG2 VAL A 25 -13.693 11.235 11.080 1.00 1.00

ATOM 142 N TYR A 26 -17.211 8.272 12.466 1.00 1.00

ATOM 143 CA TYR A 26 -17.880 7.006 12.752 1.00 1.00

ATOM 144 C TYR A 26 -18.131 6.175 11.497 1.00 1.00

ATOM 145 O TYR A 26 -18.486 6.711 10.447 1.00 1.00

ATOM 146 CB TYR A 26 -19.218 7.250 13.460 1.00 1.00

ATOM 147 CG TYR A 26 -19.117 7.942 14.802 1.00 1.00

ATOM 148 CD1 TYR A 26 -19.445 9.290 14.937 1.00 1.00

ATOM 149 CD2 TYR A 26 -18.709 7.245 15.942 1.00 1.00

ATOM 150 CE1 TYR A 26 -19.363 9.934 16.170 1.00 1.00

ATOM 151 CE2 TYR A 26 -18.623 7.880 17.180 1.00 1.00

ATOM 152 CZ TYR A 26 -18.952 9.222 17.286 1.00 1.00

ATOM 153 OH TYR A 26 -18.869 9.854 18.508 1.00 1.00

ATOM 154 N PHE A 27 -17.942 4.863 11.623 1.00 1.00

ATOM 155 CA PHE A 27 -18.354 3.914 10.594 1.00 1.00

ATOM 156 C PHE A 27 -19.603 3.184 11.078 1.00 1.00

ATOM 157 O PHE A 27 -19.561 2.457 12.072 1.00 1.00

ATOM 158 CB PHE A 27 -17.235 2.909 10.293 1.00 1.00

ATOM 159 CG PHE A 27 -16.260 3.363 9.231 1.00 1.00

ATOM 160 CD1 PHE A 27 -16.177 4.701 8.847 1.00 1.00

ATOM 161 CD2 PHE A 27 -15.412 2.441 8.624 1.00 1.00

ATOM 162 CE1 PHE A 27 -15.266 5.111 7.873 1.00 1.00

ATOM 163 CE2 PHE A 27 -14.500 2.841 7.647 1.00 1.00

ATOM 164 CZ PHE A 27 -14.428 4.180 7.273 1.00 1.00

ATOM 165 N ARG A 28 -20.715 3.396 10.380 1.00 1.00

ATOM 166 CA ARG A 28 -21.978 2.745 10.717 1.00 1.00

ATOM 167 C ARG A 28 -22.413 1.800 9.602 1.00 1.00

ATOM 168 O ARG A 28 -22.226 2.096 8.421 1.00 1.00

ATOM 169 CB ARG A 28 -23.077 3.777 10.997 1.00 1.00

ATOM 170 CG ARG A 28 -22.707 4.841 12.027 1.00 1.00

ATOM 171 CD ARG A 28 -23.058 4.484 13.463 1.00 1.00

ATOM 172 NE ARG A 28 -22.739 5.572 14.389 1.00 1.00

ATOM 173 CZ ARG A 28 -22.233 5.401 15.608 1.00 1.00

ATOM 174 NH1 ARG A 28 -21.980 6.458 16.372 1.00 1.00

ATOM 175 NH2 ARG A 28 -21.976 4.184 16.070 1.00 1.00

ATOM 176 N GLY A 29 -22.984 0.663 9.992 1.00 1.00

ATOM 177 CA GLY A 29 -23.461 -0.331 9.045 1.00 1.00

ATOM 178 C GLY A 29 -22.346 -1.091 8.348 1.00 1.00

ATOM 179 O GLY A 29 -22.407 -1.318 7.137 1.00 1.00

ATOM 180 N VAL A 30 -21.329 -1.479 9.118 1.00 1.00

ATOM 181 CA VAL A 30 -20.206 -2.268 8.612 1.00 1.00

ATOM 182 C VAL A 30 -20.703 -3.666 8.229 1.00 1.00

ATOM 183 O VAL A 30 -21.301 -4.357 9.059 1.00 1.00

ATOM 184 CB VAL A 30 -19.054 -2.354 9.654 1.00 1.00

ATOM 185 CG1 VAL A 30 -17.937 -3.281 9.179 1.00 1.00

ATOM 186 CG2 VAL A 30 -18.493 -0.968 9.959 1.00 1.00

ATOM 187 N PRO A 31 -20.467 -4.075 6.978 1.00 1.00

ATOM 188 CA PRO A 31 -20.999 -5.343 6.456 1.00 1.00

ATOM 189 C PRO A 31 -20.384 -6.584 7.104 1.00 1.00

ATOM 190 O PRO A 31 -19.391 -6.480 7.830 1.00 1.00

ATOM 191 CB PRO A 31 -20.644 -5.286 4.965 1.00 1.00

ATOM 192 CG PRO A 31 -19.470 -4.378 4.890 1.00 1.00

ATOM 193 CD PRO A 31 -19.678 -3.353 5.964 1.00 1.00

ATOM 194 N SER A 32 -20.949 -7.741 6.756 1.00 1.00

ATOM 195 CA SER A 32 -20.514 -8.969 7.421 1.00 1.00

ATOM 196 C SER A 32 -19.112 -9.385 6.951 1.00 1.00

ATOM 197 O SER A 32 -18.348 -9.998 7.708 1.00 1.00

ATOM 198 CB SER A 32 -21.490 -10.091 7.126 1.00 1.00

ATOM 199 OG SER A 32 -21.773 -10.187 5.735 1.00 1.00

ATOM 200 N LYS A 33 -18.793 -9.042 5.707 1.00 1.00

ATOM 201 CA LYS A 33 -17.474 -9.376 5.118 1.00 1.00

ATOM 202 C LYS A 33 -16.394 -8.386 5.651 1.00 1.00

ATOM 203 O LYS A 33 -15.187 -8.639 5.535 1.00 1.00

ATOM 204 CB LYS A 33 -17.536 -9.322 3.564 1.00 1.00

ATOM 205 CG LYS A 33 -18.515 -10.307 2.974 1.00 1.00

ATOM 206 CD LYS A 33 -18.630 -10.105 1.472 1.00 1.00

ATOM 207 CE LYS A 33 -19.982 -9.522 1.094 1.00 1.00

ATOM 208 NZ LYS A 33 -20.074 -9.233 -0.364 1.00 1.00

ATOM 209 N GLY A 34 -16.862 -7.274 6.229 1.00 1.00

ATOM 210 CA GLY A 34 -15.983 -6.225 6.830 1.00 1.00

ATOM 211 C GLY A 34 -15.553 -5.173 5.798 1.00 1.00

ATOM 212 O GLY A 34 -16.113 -5.079 4.704 1.00 1.00

ATOM 213 N ILE A 35 -14.557 -4.394 6.194 1.00 1.00

ATOM 214 CA ILE A 35 -13.980 -3.345 5.335 1.00 1.00

ATOM 215 C ILE A 35 -12.454 -3.470 5.339 1.00 1.00

ATOM 216 O ILE A 35 -11.798 -3.259 6.371 1.00 1.00

ATOM 217 CB ILE A 35 -14.357 -1.939 5.823 1.00 1.00

ATOM 218 CG1 ILE A 35 -15.885 -1.687 5.838 1.00 1.00

ATOM 219 CG2 ILE A 35 -13.739 -0.834 4.950 1.00 1.00

ATOM 220 CD1 ILE A 35 -16.527 -1.663 4.443 1.00 1.00

ATOM 221 N ALA A 36 -11.917 -3.941 4.220 1.00 1.00

ATOM 222 CA ALA A 36 -10.484 -4.206 4.080 1.00 1.00

ATOM 223 C ALA A 36 -9.691 -2.957 3.695 1.00 1.00

ATOM 224 O ALA A 36 -8.515 -2.829 4.043 1.00 1.00

ATOM 225 CB ALA A 36 -10.249 -5.321 3.066 1.00 1.00

ATOM 226 N ASN A 37 -10.339 -2.048 2.969 1.00 1.00

ATOM 227 CA ASN A 37 -9.706 -0.809 2.520 1.00 1.00

ATOM 228 C ASN A 37 -10.710 0.308 2.257 1.00 1.00

ATOM 229 O ASN A 37 -11.846 0.052 1.846 1.00 1.00

ATOM 230 CB ASN A 37 -8.861 -1.053 1.261 1.00 1.00

ATOM 231 CG ASN A 37 -9.622 -1.801 0.180 1.00 1.00

ATOM 232 OD1 ASN A 37 -9.867 -3.004 0.295 1.00 1.00

ATOM 233 ND2 ASN A 37 -9.996 -1.094 -0.877 1.00 1.00

ATOM 234 N CYS A 38 -10.277 1.544 2.493 1.00 1.00

ATOM 235 CA CYS A 38 -11.066 2.728 2.164 1.00 1.00

ATOM 236 C CYS A 38 -10.207 3.985 2.094 1.00 1.00

ATOM 237 O CYS A 38 -9.181 4.093 2.773 1.00 1.00

ATOM 238 CB CYS A 38 -12.215 2.926 3.160 1.00 1.00

ATOM 239 SG CYS A 38 -11.700 3.240 4.856 1.00 1.00

ATOM 240 N ASP A 39 -10.624 4.923 1.250 1.00 1.00

ATOM 241 CA ASP A 39 -10.000 6.238 1.174 1.00 1.00

ATOM 242 C ASP A 39 -11.036 7.297 0.825 1.00 1.00

ATOM 243 O ASP A 39 -11.946 7.052 0.029 1.00 1.00

ATOM 244 CB ASP A 39 -8.826 6.252 0.182 1.00 1.00

ATOM 245 CG ASP A 39 -9.275 6.340 -1.264 1.00 1.00

ATOM 246 OD1 ASP A 39 -9.508 5.280 -1.880 1.00 1.00

ATOM 247 OD2 ASP A 39 -9.422 7.424 -1.866 1.00 1.00

ATOM 248 N PHE A 40 -10.903 8.462 1.453 1.00 1.00

ATOM 249 CA PHE A 40 -11.802 9.589 1.214 1.00 1.00

ATOM 250 C PHE A 40 -11.191 10.918 1.651 1.00 1.00

ATOM 251 O PHE A 40 -10.274 10.954 2.476 1.00 1.00

ATOM 252 CB PHE A 40 -13.179 9.380 1.880 1.00 1.00

ATOM 253 CG PHE A 40 -13.119 8.748 3.247 1.00 1.00

ATOM 254 CD1 PHE A 40 -12.876 9.524 4.378 1.00 1.00

ATOM 255 CD2 PHE A 40 -13.335 7.383 3.406 1.00 1.00

ATOM 256 CE1 PHE A 40 -12.830 8.944 5.645 1.00 1.00

ATOM 257 CE2 PHE A 40 -13.291 6.794 4.667 1.00 1.00

ATOM 258 CZ PHE A 40 -13.039 7.578 5.789 1.00 1.00

ATOM 259 N VAL A 41 -11.709 12.000 1.077 1.00 1.00

ATOM 260 CA VAL A 41 -11.228 13.352 1.342 1.00 1.00

ATOM 261 C VAL A 41 -12.326 14.172 2.019 1.00 1.00

ATOM 262 O VAL A 41 -13.487 14.127 1.603 1.00 1.00

ATOM 263 CB VAL A 41 -10.783 14.059 0.029 1.00 1.00

ATOM 264 CG1 VAL A 41 -10.251 15.464 0.306 1.00 1.00

ATOM 265 CG2 VAL A 41 -9.737 13.232 -0.713 1.00 1.00

ATOM 266 N PHE A 42 -11.958 14.902 3.071 1.00 1.00

ATOM 267 CA PHE A 42 -12.865 15.842 3.726 1.00 1.00

ATOM 268 C PHE A 42 -12.389 17.281 3.524 1.00 1.00

ATOM 269 O PHE A 42 -11.186 17.543 3.456 1.00 1.00

ATOM 270 CB PHE A 42 -12.987 15.551 5.231 1.00 1.00

ATOM 271 CG PHE A 42 -13.671 14.243 5.565 1.00 1.00

ATOM 272 CD1 PHE A 42 -13.521 13.685 6.831 1.00 1.00

ATOM 273 CD2 PHE A 42 -14.472 13.579 4.636 1.00 1.00

ATOM 274 CE1 PHE A 42 -14.141 12.482 7.164 1.00 1.00

ATOM 275 CE2 PHE A 42 -15.098 12.376 4.960 1.00 1.00

ATOM 276 CZ PHE A 42 -14.932 11.829 6.229 1.00 1.00

ATOM 277 N ARG A 43 -13.341 18.206 3.434 1.00 1.00

ATOM 278 CA ARG A 43 -13.047 19.631 3.299 1.00 1.00

ATOM 279 C ARG A 43 -13.218 20.345 4.642 1.00 1.00

ATOM 280 O ARG A 43 -14.064 19.959 5.452 1.00 1.00

ATOM 281 CB ARG A 43 -13.968 20.257 2.249 1.00 1.00

ATOM 282 CG ARG A 43 -13.427 21.522 1.602 1.00 1.00

ATOM 283 CD ARG A 43 -13.790 21.673 0.133 1.00 1.00

ATOM 284 NE ARG A 43 -12.635 21.456 -0.735 1.00 1.00

ATOM 285 CZ ARG A 43 -12.701 21.194 -2.036 1.00 1.00

ATOM 286 NH1 ARG A 43 -13.878 21.115 -2.651 1.00 1.00

ATOM 287 NH2 ARG A 43 -11.586 21.011 -2.728 1.00 1.00

ATOM 288 N TYR A 44 -12.407 21.377 4.869 1.00 1.00

ATOM 289 CA TYR A 44 -12.500 22.199 6.080 1.00 1.00

ATOM 290 C TYR A 44 -11.967 23.618 5.866 1.00 1.00

ATOM 291 O TYR A 44 -11.327 23.904 4.852 1.00 1.00

ATOM 292 CB TYR A 44 -11.787 21.519 7.261 1.00 1.00

ATOM 293 CG TYR A 44 -10.279 21.686 7.294 1.00 1.00

ATOM 294 CD1 TYR A 44 -9.459 20.984 6.409 1.00 1.00

ATOM 295 CD2 TYR A 44 -9.673 22.535 8.221 1.00 1.00

ATOM 296 CE1 TYR A 44 -8.073 21.129 6.442 1.00 1.00

ATOM 297 CE2 TYR A 44 -8.290 22.687 8.264 1.00 1.00

ATOM 298 CZ TYR A 44 -7.497 21.981 7.371 1.00 1.00

ATOM 299 OH TYR A 44 -6.130 22.126 7.407 1.00 1.00

ATOM 300 N ASP A 45 -12.243 24.498 6.828 1.00 1.00

ATOM 301 CA ASP A 45 -11.743 25.871 6.809 1.00 1.00

ATOM 302 C ASP A 45 -10.557 26.029 7.763 1.00 1.00

ATOM 303 O ASP A 45 -10.722 25.947 8.984 1.00 1.00

ATOM 304 CB ASP A 45 -12.857 26.859 7.174 1.00 1.00

ATOM 305 CG ASP A 45 -12.555 28.285 6.731 1.00 1.00

ATOM 306 OD1 ASP A 45 -11.368 28.671 6.659 1.00 1.00

ATOM 307 OD2 ASP A 45 -13.456 29.101 6.437 1.00 1.00

ATOM 308 N PRO A 46 -9.367 26.260 7.204 1.00 1.00

ATOM 309 CA PRO A 46 -8.139 26.432 7.996 1.00 1.00

ATOM 310 C PRO A 46 -8.123 27.708 8.841 1.00 1.00

ATOM 311 O PRO A 46 -7.372 27.772 9.816 1.00 1.00

ATOM 312 CB PRO A 46 -7.040 26.496 6.928 1.00 1.00

ATOM 313 CG PRO A 46 -7.668 25.946 5.701 1.00 1.00

ATOM 314 CD PRO A 46 -9.100 26.362 5.759 1.00 1.00

ATOM 315 N ASN A 47 -8.927 28.702 8.465 1.00 1.00

ATOM 316 CA ASN A 47 -9.056 29.937 9.241 1.00 1.00

ATOM 317 C ASN A 47 -9.897 29.745 10.502 1.00 1.00

ATOM 318 O ASN A 47 -9.771 30.506 11.464 1.00 1.00

ATOM 319 CB ASN A 47 -9.648 31.064 8.386 1.00 1.00

ATOM 320 CG ASN A 47 -8.770 31.430 7.202 1.00 1.00

ATOM 321 OD1 ASN A 47 -7.541 31.464 7.303 1.00 1.00

ATOM 322 ND2 ASN A 47 -9.400 31.712 6.067 1.00 1.00

ATOM 323 N VAL A 48 -10.757 28.728 10.484 1.00 1.00

ATOM 324 CA VAL A 48 -11.626 28.414 11.614 1.00 1.00

ATOM 325 C VAL A 48 -11.025 27.306 12.481 1.00 1.00

ATOM 326 O VAL A 48 -11.031 27.401 13.710 1.00 1.00

ATOM 327 CB VAL A 48 -13.050 28.008 11.147 1.00 1.00

ATOM 328 CG1 VAL A 48 -13.966 27.782 12.341 1.00 1.00

ATOM 329 CG2 VAL A 48 -13.641 29.062 10.214 1.00 1.00

ATOM 330 N LEU A 49 -10.504 26.263 11.836 1.00 1.00

ATOM 331 CA LEU A 49 -9.929 25.122 12.545 1.00 1.00

ATOM 332 C LEU A 49 -8.503 24.828 12.102 1.00 1.00

ATOM 333 O LEU A 49 -8.210 24.768 10.907 1.00 1.00

ATOM 334 CB LEU A 49 -10.790 23.867 12.357 1.00 1.00

ATOM 335 CG LEU A 49 -12.254 23.862 12.806 1.00 1.00

ATOM 336 CD1 LEU A 49 -12.935 22.585 12.333 1.00 1.00

ATOM 337 CD2 LEU A 49 -12.386 24.016 14.316 1.00 1.00

ATOM 338 N GLU A 50 -7.618 24.650 13.079 1.00 1.00

ATOM 339 CA GLU A 50 -6.256 24.201 12.811 1.00 1.00

ATOM 340 C GLU A 50 -6.145 22.724 13.176 1.00 1.00

ATOM 341 O GLU A 50 -6.047 22.372 14.353 1.00 1.00

ATOM 342 CB GLU A 50 -5.237 25.040 13.591 1.00 1.00

ATOM 343 CG GLU A 50 -3.784 24.689 13.296 1.00 1.00

ATOM 344 CD GLU A 50 -2.800 25.391 14.213 1.00 1.00

ATOM 345 OE1 GLU A 50 -3.032 25.426 15.441 1.00 1.00

ATOM 346 OE2 GLU A 50 -1.782 25.905 13.702 1.00 1.00

ATOM 347 N ILE A 51 -6.181 21.864 12.160 1.00 1.00

ATOM 348 CA ILE A 51 -6.078 20.423 12.374 1.00 1.00

ATOM 349 C ILE A 51 -4.613 20.035 12.560 1.00 1.00

ATOM 350 O ILE A 51 -3.797 20.174 11.645 1.00 1.00

ATOM 351 CB ILE A 51 -6.746 19.630 11.219 1.00 1.00

ATOM 352 CG1 ILE A 51 -8.253 19.920 11.182 1.00 1.00

ATOM 353 CG2 ILE A 51 -6.485 18.127 11.371 1.00 1.00

ATOM 354 CD1 ILE A 51 -8.964 19.434 9.934 1.00 1.00

ATOM 355 N ILE A 52 -4.296 19.556 13.759 1.00 1.00

ATOM 356 CA ILE A 52 -2.916 19.267 14.149 1.00 1.00

ATOM 357 C ILE A 52 -2.565 17.778 14.086 1.00 1.00

ATOM 358 O ILE A 52 -1.395 17.406 14.206 1.00 1.00

ATOM 359 CB ILE A 52 -2.605 19.849 15.556 1.00 1.00

ATOM 360 CG1 ILE A 52 -3.650 19.402 16.585 1.00 1.00

ATOM 361 CG2 ILE A 52 -2.514 21.374 15.495 1.00 1.00

ATOM 362 CD1 ILE A 52 -3.062 19.002 17.928 1.00 1.00

ATOM 363 N GLY A 53 -3.580 16.935 13.897 1.00 1.00

ATOM 364 CA GLY A 53 -3.370 15.502 13.798 1.00 1.00

ATOM 365 C GLY A 53 -4.637 14.686 13.632 1.00 1.00

ATOM 366 O GLY A 53 -5.736 15.142 13.963 1.00 1.00

ATOM 367 N ILE A 54 -4.476 13.476 13.098 1.00 1.00

ATOM 368 CA ILE A 54 -5.569 12.513 12.973 1.00 1.00

ATOM 369 C ILE A 54 -5.101 11.145 13.475 1.00 1.00

ATOM 370 O ILE A 54 -4.070 10.632 13.033 1.00 1.00

ATOM 371 CB ILE A 54 -6.084 12.413 11.509 1.00 1.00

ATOM 372 CG1 ILE A 54 -6.525 13.783 10.978 1.00 1.00

ATOM 373 CG2 ILE A 54 -7.240 11.420 11.413 1.00 1.00

ATOM 374 CD1 ILE A 54 -6.438 13.927 9.466 1.00 1.00

ATOM 375 N ASP A 55 -5.866 10.570 14.399 1.00 1.00

ATOM 376 CA ASP A 55 -5.539 9.276 14.991 1.00 1.00

ATOM 377 C ASP A 55 -6.551 8.209 14.580 1.00 1.00

ATOM 378 O ASP A 55 -7.730 8.517 14.407 1.00 1.00

ATOM 379 CB ASP A 55 -5.497 9.382 16.519 1.00 1.00

ATOM 380 CG ASP A 55 -4.502 10.415 17.010 1.00 1.00

ATOM 381 OD1 ASP A 55 -3.298 10.290 16.699 1.00 1.00

ATOM 382 OD2 ASP A 55 -4.839 11.387 17.718 1.00 1.00

ATOM 383 N PRO A 56 -6.098 6.965 14.416 1.00 1.00

ATOM 384 CA PRO A 56 -7.004 5.843 14.134 1.00 1.00

ATOM 385 C PRO A 56 -7.872 5.490 15.342 1.00 1.00

ATOM 386 O PRO A 56 -7.402 5.562 16.480 1.00 1.00

ATOM 387 CB PRO A 56 -6.048 4.689 13.815 1.00 1.00

ATOM 388 CG PRO A 56 -4.782 5.037 14.515 1.00 1.00

ATOM 389 CD PRO A 56 -4.688 6.533 14.466 1.00 1.00

ATOM 390 N GLY A 57 -9.123 5.120 15.086 1.00 1.00

ATOM 391 CA GLY A 57 -10.039 4.711 16.137 1.00 1.00

ATOM 392 C GLY A 57 -9.835 3.269 16.562 1.00 1.00

ATOM 393 O GLY A 57 -8.948 2.582 16.049 1.00 1.00

ATOM 394 N ASP A 58 -10.667 2.808 17.495 1.00 1.00

ATOM 395 CA ASP A 58 -10.531 1.463 18.059 1.00 1.00

ATOM 396 C ASP A 58 -10.989 0.336 17.127 1.00 1.00

ATOM 397 O ASP A 58 -10.685 -0.834 17.377 1.00 1.00

ATOM 398 CB ASP A 58 -11.233 1.353 19.425 1.00 1.00

ATOM 399 CG ASP A 58 -12.575 2.064 19.464 1.00 1.00

ATOM 400 OD1 ASP A 58 -13.460 1.738 18.643 1.00 1.00

ATOM 401 OD2 ASP A 58 -12.837 2.960 20.296 1.00 1.00

ATOM 402 N ILE A 59 -11.708 0.683 16.062 1.00 1.00

ATOM 403 CA ILE A 59 -12.164 -0.316 15.086 1.00 1.00

ATOM 404 C ILE A 59 -11.050 -0.760 14.128 1.00 1.00

ATOM 405 O ILE A 59 -11.219 -1.716 13.368 1.00 1.00

ATOM 406 CB ILE A 59 -13.439 0.153 14.320 1.00 1.00

ATOM 407 CG1 ILE A 59 -13.137 1.350 13.407 1.00 1.00

ATOM 408 CG2 ILE A 59 -14.582 0.440 15.303 1.00 1.00

ATOM 409 CD1 ILE A 59 -14.219 1.644 12.382 1.00 1.00

ATOM 410 N ILE A 60 -9.917 -0.060 14.176 1.00 1.00

ATOM 411 CA ILE A 60 -8.702 -0.509 13.505 1.00 1.00

ATOM 412 C ILE A 60 -7.897 -1.346 14.499 1.00 1.00

ATOM 413 O ILE A 60 -7.414 -0.831 15.513 1.00 1.00

ATOM 414 CB ILE A 60 -7.894 0.692 12.943 1.00 1.00

ATOM 415 CG1 ILE A 60 -8.543 1.200 11.651 1.00 1.00

ATOM 416 CG2 ILE A 60 -6.437 0.310 12.669 1.00 1.00

ATOM 417 CD1 ILE A 60 -8.824 2.682 11.648 1.00 1.00

ATOM 418 N VAL A 61 -7.776 -2.638 14.206 1.00 1.00

ATOM 419 CA VAL A 61 -7.240 -3.611 15.164 1.00 1.00

ATOM 420 C VAL A 61 -5.775 -3.994 14.933 1.00 1.00

ATOM 421 O VAL A 61 -5.186 -4.716 15.743 1.00 1.00

ATOM 422 CB VAL A 61 -8.123 -4.892 15.247 1.00 1.00

ATOM 423 CG1 VAL A 61 -9.499 -4.565 15.820 1.00 1.00

ATOM 424 CG2 VAL A 61 -8.246 -5.574 13.883 1.00 1.00

ATOM 425 N ASP A 62 -5.196 -3.512 13.832 1.00 1.00

ATOM 426 CA ASP A 62 -3.781 -3.724 13.526 1.00 1.00

ATOM 427 C ASP A 62 -2.908 -3.288 14.711 1.00 1.00

ATOM 428 O ASP A 62 -2.964 -2.126 15.124 1.00 1.00

ATOM 429 CB ASP A 62 -3.388 -2.944 12.260 1.00 1.00

ATOM 430 CG ASP A 62 -2.070 -3.411 11.650 1.00 1.00

ATOM 431 OD1 ASP A 62 -1.118 -3.729 12.396 1.00 1.00

ATOM 432 OD2 ASP A 62 -1.883 -3.479 10.417 1.00 1.00

ATOM 433 N PRO A 63 -2.128 -4.224 15.264 1.00 1.00

ATOM 434 CA PRO A 63 -1.211 -3.931 16.378 1.00 1.00

ATOM 435 C PRO A 63 -0.143 -2.897 16.012 1.00 1.00

ATOM 436 O PRO A 63 0.400 -2.232 16.899 1.00 1.00

ATOM 437 CB PRO A 63 -0.558 -5.289 16.664 1.00 1.00

ATOM 438 CG PRO A 63 -0.753 -6.083 15.418 1.00 1.00

ATOM 439 CD PRO A 63 -2.073 -5.644 14.871 1.00 1.00

ATOM 440 N ASN A 64 0.151 -2.787 14.717 1.00 1.00

ATOM 441 CA ASN A 64 0.983 -1.721 14.174 1.00 1.00

ATOM 442 C ASN A 64 0.116 -0.846 13.259 1.00 1.00

ATOM 443 O ASN A 64 0.188 -0.969 12.034 1.00 1.00

ATOM 444 CB ASN A 64 2.156 -2.315 13.388 1.00 1.00

ATOM 445 CG ASN A 64 3.499 -1.746 13.809 1.00 1.00

ATOM 446 OD1 ASN A 64 4.408 -2.490 14.180 1.00 1.00

ATOM 447 ND2 ASN A 64 3.637 -0.423 13.744 1.00 1.00

ATOM 448 N PRO A 65 -0.703 0.030 13.850 1.00 1.00

ATOM 449 CA PRO A 65 -1.763 0.731 13.108 1.00 1.00

ATOM 450 C PRO A 65 -1.287 1.559 11.913 1.00 1.00

ATOM 451 O PRO A 65 -2.055 1.707 10.960 1.00 1.00

ATOM 452 CB PRO A 65 -2.405 1.634 14.171 1.00 1.00

ATOM 453 CG PRO A 65 -1.394 1.743 15.251 1.00 1.00

ATOM 454 CD PRO A 65 -0.679 0.430 15.269 1.00 1.00

ATOM 455 N THR A 66 -0.057 2.069 11.955 1.00 1.00

ATOM 456 CA THR A 66 0.486 2.892 10.866 1.00 1.00

ATOM 457 C THR A 66 0.716 2.099 9.577 1.00 1.00

ATOM 458 O THR A 66 0.812 2.682 8.494 1.00 1.00

ATOM 459 CB THR A 66 1.794 3.599 11.294 1.00 1.00

ATOM 460 OG1 THR A 66 2.730 2.631 11.784 1.00 1.00

ATOM 461 CG2 THR A 66 1.555 4.519 12.491 1.00 1.00

ATOM 462 N LYS A 67 0.809 0.776 9.701 1.00 1.00

ATOM 463 CA LYS A 67 0.960 -0.108 8.546 1.00 1.00

ATOM 464 C LYS A 67 -0.332 -0.190 7.729 1.00 1.00

ATOM 465 O LYS A 67 -0.290 -0.364 6.510 1.00 1.00

ATOM 466 CB LYS A 67 1.399 -1.510 8.987 1.00 1.00

ATOM 467 CG LYS A 67 2.718 -1.548 9.752 1.00 1.00

ATOM 468 CD LYS A 67 3.770 -2.365 9.017 1.00 1.00

ATOM 469 CE LYS A 67 5.012 -2.564 9.877 1.00 1.00

ATOM 470 NZ LYS A 67 6.200 -1.861 9.313 1.00 1.00

ATOM 471 N SER A 68 -1.468 -0.051 8.412 1.00 1.00

ATOM 472 CA SER A 68 -2.788 -0.138 7.783 1.00 1.00

ATOM 473 C SER A 68 -3.482 1.216 7.613 1.00 1.00

ATOM 474 O SER A 68 -4.354 1.361 6.756 1.00 1.00

ATOM 475 CB SER A 68 -3.696 -1.071 8.590 1.00 1.00

ATOM 476 OG SER A 68 -3.262 -2.417 8.498 1.00 1.00

ATOM 477 N PHE A 69 -3.089 2.198 8.424 1.00 1.00

ATOM 478 CA PHE A 69 -3.785 3.484 8.503 1.00 1.00

ATOM 479 C PHE A 69 -2.818 4.656 8.346 1.00 1.00

ATOM 480 O PHE A 69 -1.797 4.715 9.030 1.00 1.00

ATOM 481 CB PHE A 69 -4.526 3.571 9.848 1.00 1.00

ATOM 482 CG PHE A 69 -5.353 4.820 10.027 1.00 1.00

ATOM 483 CD1 PHE A 69 -4.818 5.944 10.651 1.00 1.00

ATOM 484 CD2 PHE A 69 -6.681 4.857 9.608 1.00 1.00

ATOM 485 CE1 PHE A 69 -5.584 7.092 10.837 1.00 1.00

ATOM 486 CE2 PHE A 69 -7.453 6.002 9.788 1.00 1.00

ATOM 487 CZ PHE A 69 -6.903 7.122 10.406 1.00 1.00

ATOM 488 N ASP A 70 -3.149 5.583 7.444 1.00 1.00

ATOM 489 CA ASP A 70 -2.368 6.806 7.242 1.00 1.00

ATOM 490 C ASP A 70 -3.255 7.972 6.806 1.00 1.00

ATOM 491 O ASP A 70 -4.283 7.768 6.155 1.00 1.00

ATOM 492 CB ASP A 70 -1.247 6.586 6.215 1.00 1.00

ATOM 493 CG ASP A 70 -0.085 7.569 6.380 1.00 1.00

ATOM 494 OD1 ASP A 70 -0.192 8.525 7.181 1.00 1.00

ATOM 495 OD2 ASP A 70 0.985 7.459 5.747 1.00 1.00

ATOM 496 N THR A 71 -2.851 9.187 7.177 1.00 1.00

ATOM 497 CA THR A 71 -3.588 10.408 6.839 1.00 1.00

ATOM 498 C THR A 71 -2.666 11.528 6.350 1.00 1.00

ATOM 499 O THR A 71 -1.437 11.421 6.428 1.00 1.00

ATOM 500 CB THR A 71 -4.417 10.921 8.046 1.00 1.00

ATOM 501 OG1 THR A 71 -3.589 10.970 9.214 1.00 1.00

ATOM 502 CG2 THR A 71 -5.527 9.945 8.418 1.00 1.00

ATOM 503 N ALA A 72 -3.274 12.604 5.854 1.00 1.00

ATOM 504 CA ALA A 72 -2.549 13.800 5.431 1.00 1.00

ATOM 505 C ALA A 72 -3.406 15.051 5.611 1.00 1.00

ATOM 506 O ALA A 72 -4.617 15.022 5.370 1.00 1.00

ATOM 507 CB ALA A 72 -2.097 13.663 3.987 1.00 1.00

ATOM 508 N ILE A 73 -2.772 16.142 6.040 1.00 1.00

ATOM 509 CA ILE A 73 -3.456 17.418 6.268 1.00 1.00

ATOM 510 C ILE A 73 -2.869 18.515 5.372 1.00 1.00

ATOM 511 O ILE A 73 -1.659 18.753 5.390 1.00 1.00

ATOM 512 CB ILE A 73 -3.369 17.834 7.768 1.00 1.00

ATOM 513 CG1 ILE A 73 -3.973 16.756 8.674 1.00 1.00

ATOM 514 CG2 ILE A 73 -4.062 19.176 8.005 1.00 1.00

ATOM 515 CD1 ILE A 73 -3.241 16.574 9.994 1.00 1.00

ATOM 516 N TYR A 74 -3.730 19.175 4.600 1.00 1.00

ATOM 517 CA TYR A 74 -3.309 20.244 3.689 1.00 1.00

ATOM 518 C TYR A 74 -4.097 21.540 3.911 1.00 1.00

ATOM 519 O TYR A 74 -5.121 21.761 3.263 1.00 1.00

ATOM 520 CB TYR A 74 -3.439 19.797 2.226 1.00 1.00

ATOM 521 CG TYR A 74 -2.804 18.461 1.925 1.00 1.00

ATOM 522 CD1 TYR A 74 -3.590 17.332 1.704 1.00 1.00

ATOM 523 CD2 TYR A 74 -1.416 18.324 1.859 1.00 1.00

ATOM 524 CE1 TYR A 74 -3.015 16.103 1.426 1.00 1.00

ATOM 525 CE2 TYR A 74 -0.831 17.097 1.585 1.00 1.00

ATOM 526 CZ TYR A 74 -1.636 15.992 1.368 1.00 1.00

ATOM 527 OH TYR A 74 -1.067 14.772 1.096 1.00 1.00

ATOM 528 N PRO A 75 -3.624 22.388 4.829 1.00 1.00

ATOM 529 CA PRO A 75 -4.261 23.690 5.086 1.00 1.00

ATOM 530 C PRO A 75 -4.358 24.591 3.849 1.00 1.00

ATOM 531 O PRO A 75 -5.389 25.239 3.666 1.00 1.00

ATOM 532 CB PRO A 75 -3.359 24.327 6.156 1.00 1.00

ATOM 533 CG PRO A 75 -2.097 23.540 6.130 1.00 1.00

ATOM 534 CD PRO A 75 -2.470 22.157 5.716 1.00 1.00

ATOM 535 N ASP A 76 -3.307 24.587 2.992 1.00 1.00

ATOM 536 CA ASP A 76 -3.318 25.448 1.799 1.00 1.00

ATOM 537 C ASP A 76 -4.378 24.986 0.784 1.00 1.00

ATOM 538 O ASP A 76 -4.891 25.784 -0.008 1.00 1.00

ATOM 539 CB ASP A 76 -1.963 25.415 1.075 1.00 1.00

ATOM 540 CG ASP A 76 -0.795 25.511 2.033 1.00 1.00

ATOM 541 OD1 ASP A 76 -0.708 26.514 2.772 1.00 1.00

ATOM 542 OD2 ASP A 76 0.037 24.580 2.044 1.00 1.00

ATOM 543 N ARG A 77 -4.694 23.694 0.821 1.00 1.00

ATOM 544 CA ARG A 77 -5.688 23.113 -0.114 1.00 1.00

ATOM 545 C ARG A 77 -7.053 22.902 0.585 1.00 1.00

ATOM 546 O ARG A 77 -8.032 22.493 -0.051 1.00 1.00

ATOM 547 CB ARG A 77 -5.180 21.782 -0.663 1.00 1.00

ATOM 548 CG ARG A 77 -4.321 21.971 -1.922 1.00 1.00

ATOM 549 CD ARG A 77 -4.010 20.666 -2.640 1.00 1.00

ATOM 550 NE ARG A 77 -2.914 19.939 -2.012 1.00 1.00

ATOM 551 CZ ARG A 77 -2.680 18.644 -2.179 1.00 1.00

ATOM 552 NH1 ARG A 77 -3.463 17.898 -2.975 1.00 1.00

ATOM 553 NH2 ARG A 77 -1.670 18.001 -1.584 1.00 1.00

ATOM 554 N LYS A 78 -7.060 23.192 1.880 1.00 1.00

ATOM 555 CA LYS A 78 -8.269 23.113 2.744 1.00 1.00

ATOM 556 C LYS A 78 -8.909 21.706 2.776 1.00 1.00

ATOM 557 O LYS A 78 -10.145 21.564 2.856 1.00 1.00

ATOM 558 CB LYS A 78 -9.328 24.078 2.224 1.00 1.00

ATOM 559 CG LYS A 78 -8.941 25.546 2.326 1.00 1.00

ATOM 560 CD LYS A 78 -8.200 26.015 1.084 1.00 1.00

ATOM 561 CE LYS A 78 -7.708 27.444 1.241 1.00 1.00

ATOM 562 NZ LYS A 78 -6.903 27.886 0.068 1.00 1.00

ATOM 563 N ILE A 79 -8.074 20.679 2.727 1.00 1.00

ATOM 564 CA ILE A 79 -8.564 19.280 2.749 1.00 1.00

ATOM 565 C ILE A 79 -7.718 18.398 3.680 1.00 1.00

ATOM 566 O ILE A 79 -6.561 18.704 3.989 1.00 1.00

ATOM 567 CB ILE A 79 -8.521 18.681 1.342 1.00 1.00

ATOM 568 CG1 ILE A 79 -7.186 18.936 0.618 1.00 1.00

ATOM 569 CG2 ILE A 79 -9.622 19.248 0.436 1.00 1.00

ATOM 570 CD1 ILE A 79 -6.749 17.772 -0.280 1.00 1.00

ATOM 571 N ILE A 80 -8.376 17.313 4.125 1.00 1.00

ATOM 572 CA ILE A 80 -7.708 16.224 4.836 1.00 1.00

ATOM 573 C ILE A 80 -8.018 14.900 4.138 1.00 1.00

ATOM 574 O ILE A 80 -9.115 14.716 3.605 1.00 1.00

ATOM 575 CB ILE A 80 -8.105 16.176 6.343 1.00 1.00

ATOM 576 CG1 ILE A 80 -9.629 16.180 6.523 1.00 1.00

ATOM 577 CG2 ILE A 80 -7.449 17.328 7.109 1.00 1.00

ATOM 578 CD1 ILE A 80 -10.104 15.680 7.884 1.00 1.00

ATOM 579 N VAL A 81 -7.048 13.990 4.125 1.00 1.00

ATOM 580 CA VAL A 81 -7.178 12.733 3.385 1.00 1.00

ATOM 581 C VAL A 81 -6.945 11.520 4.284 1.00 1.00

ATOM 582 O VAL A 81 -6.044 11.524 5.121 1.00 1.00

ATOM 583 CB VAL A 81 -6.216 12.668 2.164 1.00 1.00

ATOM 584 CG1 VAL A 81 -6.644 11.571 1.191 1.00 1.00

ATOM 585 CG2 VAL A 81 -6.138 14.015 1.446 1.00 1.00

ATOM 586 N PHE A 82 -7.763 10.489 4.092 1.00 1.00

ATOM 587 CA PHE A 82 -7.670 9.248 4.856 1.00 1.00

ATOM 588 C PHE A 82 -7.307 8.086 3.937 1.00 1.00

ATOM 589 O PHE A 82 -7.871 7.952 2.851 1.00 1.00

ATOM 590 CB PHE A 82 -9.005 8.942 5.547 1.00 1.00

ATOM 591 CG PHE A 82 -9.433 9.982 6.550 1.00 1.00

ATOM 592 CD1 PHE A 82 -10.060 11.153 6.136 1.00 1.00

ATOM 593 CD2 PHE A 82 -9.226 9.779 7.909 1.00 1.00

ATOM 594 CE1 PHE A 82 -10.462 12.113 7.062 1.00 1.00

ATOM 595 CE2 PHE A 82 -9.626 10.732 8.843 1.00 1.00

ATOM 596 CZ PHE A 82 -10.244 11.901 8.418 1.00 1.00

ATOM 597 N LEU A 83 -6.365 7.252 4.371 1.00 1.00

ATOM 598 CA LEU A 83 -6.029 6.031 3.639 1.00 1.00

ATOM 599 C LEU A 83 -5.953 4.817 4.558 1.00 1.00

ATOM 600 O LEU A 83 -5.056 4.709 5.397 1.00 1.00

ATOM 601 CB LEU A 83 -4.728 6.194 2.837 1.00 1.00

ATOM 602 CG LEU A 83 -4.128 4.965 2.132 1.00 1.00

ATOM 603 CD1 LEU A 83 -5.061 4.393 1.069 1.00 1.00

ATOM 604 CD2 LEU A 83 -2.773 5.292 1.523 1.00 1.00

ATOM 605 N PHE A 84 -6.911 3.910 4.390 1.00 1.00

ATOM 606 CA PHE A 84 -6.908 2.639 5.104 1.00 1.00

ATOM 607 C PHE A 84 -6.803 1.485 4.118 1.00 1.00

ATOM 608 O PHE A 84 -7.534 1.432 3.130 1.00 1.00

ATOM 609 CB PHE A 84 -8.163 2.485 5.972 1.00 1.00

ATOM 610 CG PHE A 84 -8.277 1.143 6.656 1.00 1.00

ATOM 611 CD1 PHE A 84 -7.495 0.843 7.770 1.00 1.00

ATOM 612 CD2 PHE A 84 -9.163 0.178 6.184 1.00 1.00

ATOM 613 CE1 PHE A 84 -7.593 -0.396 8.405 1.00 1.00

ATOM 614 CE2 PHE A 84 -9.272 -1.066 6.812 1.00 1.00

ATOM 615 CZ PHE A 84 -8.483 -1.351 7.925 1.00 1.00

ATOM 616 N ALA A 85 -5.871 0.578 4.395 1.00 1.00

ATOM 617 CA ALA A 85 -5.725 -0.669 3.652 1.00 1.00

ATOM 618 C ALA A 85 -5.080 -1.691 4.577 1.00 1.00

ATOM 619 O ALA A 85 -3.870 -1.642 4.819 1.00 1.00

ATOM 620 CB ALA A 85 -4.886 -0.462 2.394 1.00 1.00

ATOM 621 N GLU A 86 -5.897 -2.603 5.104 1.00 1.00

ATOM 622 CA GLU A 86 -5.441 -3.590 6.085 1.00 1.00

ATOM 623 C GLU A 86 -4.192 -4.332 5.600 1.00 1.00

ATOM 624 O GLU A 86 -4.129 -4.780 4.455 1.00 1.00

ATOM 625 CB GLU A 86 -6.580 -4.544 6.480 1.00 1.00

ATOM 626 CG GLU A 86 -6.933 -5.626 5.468 1.00 1.00

ATOM 627 CD GLU A 86 -6.156 -6.909 5.695 1.00 1.00

ATOM 628 OE1 GLU A 86 -5.828 -7.206 6.861 1.00 1.00

ATOM 629 OE2 GLU A 86 -5.865 -7.618 4.709 1.00 1.00

ATOM 630 N ASP A 87 -3.200 -4.442 6.480 1.00 1.00

ATOM 631 CA ASP A 87 -1.862 -4.884 6.088 1.00 1.00

ATOM 632 C ASP A 87 -1.496 -6.315 6.505 1.00 1.00

ATOM 633 O ASP A 87 -0.317 -6.682 6.516 1.00 1.00

ATOM 634 CB ASP A 87 -0.816 -3.887 6.608 1.00 1.00

ATOM 635 CG ASP A 87 0.461 -3.893 5.790 1.00 1.00

ATOM 636 OD1 ASP A 87 0.380 -3.906 4.543 1.00 1.00

ATOM 637 OD2 ASP A 87 1.595 -3.889 6.314 1.00 1.00

ATOM 638 N SER A 88 -2.501 -7.124 6.835 1.00 1.00

ATOM 639 CA SER A 88 -2.261 -8.515 7.233 1.00 1.00

ATOM 640 C SER A 88 -1.913 -9.402 6.041 1.00 1.00

ATOM 641 O SER A 88 -1.093 -10.314 6.158 1.00 1.00

ATOM 642 CB SER A 88 -3.467 -9.087 7.983 1.00 1.00

ATOM 643 OG SER A 88 -4.495 -9.475 7.088 1.00 1.00

ATOM 644 N GLY A 89 -2.548 -9.129 4.901 1.00 1.00

ATOM 645 CA GLY A 89 -2.381 -9.932 3.702 1.00 1.00

ATOM 646 C GLY A 89 -3.448 -11.002 3.560 1.00 1.00

ATOM 647 O GLY A 89 -3.565 -11.633 2.506 1.00 1.00

ATOM 648 N THR A 90 -4.227 -11.201 4.623 1.00 1.00

ATOM 649 CA THR A 90 -5.229 -12.270 4.684 1.00 1.00

ATOM 650 C THR A 90 -6.643 -11.740 4.925 1.00 1.00

ATOM 651 O THR A 90 -7.622 -12.474 4.766 1.00 1.00

ATOM 652 CB THR A 90 -4.868 -13.289 5.795 1.00 1.00

ATOM 653 OG1 THR A 90 -4.710 -12.603 7.044 1.00 1.00

ATOM 654 CG2 THR A 90 -3.494 -13.912 5.551 1.00 1.00

ATOM 655 N GLY A 91 -6.745 -10.471 5.312 1.00 1.00

ATOM 656 CA GLY A 91 -8.020 -9.869 5.664 1.00 1.00

ATOM 657 C GLY A 91 -8.254 -9.830 7.165 1.00 1.00

ATOM 658 O GLY A 91 -9.351 -9.497 7.618 1.00 1.00

ATOM 659 N ALA A 92 -7.216 -10.163 7.929 1.00 1.00

ATOM 660 CA ALA A 92 -7.306 -10.260 9.387 1.00 1.00

ATOM 661 C ALA A 92 -7.448 -8.906 10.084 1.00 1.00

ATOM 662 O ALA A 92 -8.075 -8.815 11.143 1.00 1.00

ATOM 663 CB ALA A 92 -6.108 -11.024 9.943 1.00 1.00

ATOM 664 N TYR A 93 -6.872 -7.861 9.492 1.00 1.00

ATOM 665 CA TYR A 93 -6.898 -6.525 10.093 1.00 1.00

ATOM 666 C TYR A 93 -7.986 -5.624 9.501 1.00 1.00

ATOM 667 O TYR A 93 -7.946 -4.400 9.652 1.00 1.00

ATOM 668 CB TYR A 93 -5.519 -5.855 10.003 1.00 1.00

ATOM 669 CG TYR A 93 -4.407 -6.589 10.732 1.00 1.00

ATOM 670 CD1 TYR A 93 -3.086 -6.492 10.297 1.00 1.00

ATOM 671 CD2 TYR A 93 -4.674 -7.372 11.860 1.00 1.00

ATOM 672 CE1 TYR A 93 -2.058 -7.161 10.960 1.00 1.00

ATOM 673 CE2 TYR A 93 -3.653 -8.044 12.528 1.00 1.00

ATOM 674 CZ TYR A 93 -2.349 -7.934 12.072 1.00 1.00

ATOM 675 OH TYR A 93 -1.337 -8.594 12.729 1.00 1.00

ATOM 676 N ALA A 94 -8.957 -6.239 8.830 1.00 1.00

ATOM 677 CA ALA A 94 -10.111 -5.522 8.292 1.00 1.00

ATOM 678 C ALA A 94 -11.007 -5.008 9.418 1.00 1.00

ATOM 679 O ALA A 94 -11.034 -5.582 10.509 1.00 1.00

ATOM 680 CB ALA A 94 -10.901 -6.423 7.353 1.00 1.00

ATOM 681 N ILE A 95 -11.706 -3.892 9.172 1.00 1.00

ATOM 682 CA ILE A 95 -12.706 -3.378 10.121 1.00 1.00

ATOM 683 C ILE A 95 -13.944 -4.275 10.054 1.00 1.00

ATOM 684 O ILE A 95 -14.535 -4.477 8.985 1.00 1.00

ATOM 685 CB ILE A 95 -13.048 -1.940 9.749 1.00 1.00

ATOM 686 CG1 ILE A 95 -11.900 -0.972 10.085 1.00 1.00

ATOM 687 CG2 ILE A 95 -14.286 -1.418 10.479 1.00 1.00

ATOM 688 CD1 ILE A 95 -11.808 0.223 9.135 1.00 1.00

ATOM 689 N THR A 96 -14.319 -4.802 11.209 1.00 1.00

ATOM 690 CA THR A 96 -15.449 -5.734 11.288 1.00 1.00

ATOM 691 C THR A 96 -16.627 -5.215 12.140 1.00 1.00

ATOM 692 O THR A 96 -17.740 -5.761 12.086 1.00 1.00

ATOM 693 CB THR A 96 -14.965 -7.061 11.893 1.00 1.00

ATOM 694 OG1 THR A 96 -14.261 -6.833 13.105 1.00 1.00

ATOM 695 CG2 THR A 96 -14.013 -7.821 10.963 1.00 1.00

ATOM 696 N LYS A 97 -16.408 -4.169 12.925 1.00 1.00

ATOM 697 CA LYS A 97 -17.497 -3.631 13.782 1.00 1.00

ATOM 698 C LYS A 97 -17.640 -2.103 13.658 1.00 1.00

ATOM 699 O LYS A 97 -16.715 -1.396 13.233 1.00 1.00

ATOM 700 CB LYS A 97 -17.232 -3.974 15.257 1.00 1.00

ATOM 701 CG LYS A 97 -16.122 -3.156 15.887 1.00 1.00

ATOM 702 CD LYS A 97 -16.019 -3.431 17.378 1.00 1.00

ATOM 703 CE LYS A 97 -17.316 -3.091 18.093 1.00 1.00

ATOM 704 NZ LYS A 97 -17.116 -2.933 19.561 1.00 1.00

ATOM 705 N ASP A 98 -18.827 -1.648 14.046 1.00 1.00

ATOM 706 CA ASP A 98 -19.196 -0.223 14.022 1.00 1.00

ATOM 707 C ASP A 98 -18.455 0.521 15.131 1.00 1.00

ATOM 708 O ASP A 98 -18.084 -0.069 16.156 1.00 1.00

ATOM 709 CB ASP A 98 -20.704 -0.062 14.238 1.00 1.00

ATOM 710 CG ASP A 98 -21.518 -0.365 12.982 1.00 1.00

ATOM 711 OD1 ASP A 98 -21.065 -1.194 12.111 1.00 1.00

ATOM 712 OD2 ASP A 98 -22.656 0.207 12.793 1.00 1.00

ATOM 713 N GLY A 99 -18.235 1.812 14.914 1.00 1.00

ATOM 714 CA GLY A 99 -17.588 2.654 15.905 1.00 1.00

ATOM 715 C GLY A 99 -16.713 3.737 15.310 1.00 1.00

ATOM 716 O GLY A 99 -16.900 4.144 14.162 1.00 1.00

ATOM 717 N VAL A 100 -15.750 4.199 16.102 1.00 1.00

ATOM 718 CA VAL A 100 -14.863 5.289 15.703 1.00 1.00

ATOM 719 C VAL A 100 -13.776 4.797 14.747 1.00 1.00

ATOM 720 O VAL A 100 -12.943 3.966 15.112 1.00 1.00

ATOM 721 CB VAL A 100 -14.220 5.985 16.933 1.00 1.00

ATOM 722 CG1 VAL A 100 -13.409 7.206 16.504 1.00 1.00

ATOM 723 CG2 VAL A 100 -15.284 6.383 17.953 1.00 1.00

ATOM 724 N PHE A 101 -13.805 5.315 13.521 1.00 1.00

ATOM 725 CA PHE A 101 -12.786 5.018 12.517 1.00 1.00

ATOM 726 C PHE A 101 -11.543 5.869 12.745 1.00 1.00

ATOM 727 O PHE A 101 -10.421 5.361 12.719 1.00 1.00

ATOM 728 CB PHE A 101 -13.345 5.239 11.104 1.00 1.00

ATOM 729 CG PHE A 101 -12.304 5.184 10.014 1.00 1.00

ATOM 730 CD1 PHE A 101 -11.718 3.974 9.649 1.00 1.00

ATOM 731 CD2 PHE A 101 -11.918 6.344 9.345 1.00 1.00

ATOM 732 CE1 PHE A 101 -10.759 3.922 8.639 1.00 1.00

ATOM 733 CE2 PHE A 101 -10.960 6.300 8.334 1.00 1.00

ATOM 734 CZ PHE A 101 -10.380 5.087 7.979 1.00 1.00

ATOM 735 N ALA A 102 -11.755 7.165 12.969 1.00 1.00

ATOM 736 CA ALA A 102 -10.666 8.112 13.182 1.00 1.00

ATOM 737 C ALA A 102 -11.079 9.253 14.108 1.00 1.00

ATOM 738 O ALA A 102 -12.267 9.545 14.259 1.00 1.00

ATOM 739 CB ALA A 102 -10.169 8.660 11.848 1.00 1.00

ATOM 740 N LYS A 103 -10.087 9.889 14.726 1.00 1.00

ATOM 741 CA LYS A 103 -10.311 11.058 15.570 1.00 1.00

ATOM 742 C LYS A 103 -9.498 12.237 15.042 1.00 1.00

ATOM 743 O LYS A 103 -8.267 12.181 14.999 1.00 1.00

ATOM 744 CB LYS A 103 -9.927 10.764 17.023 1.00 1.00

ATOM 745 CG LYS A 103 -10.616 9.545 17.611 1.00 1.00

ATOM 746 CD LYS A 103 -11.740 9.946 18.551 1.00 1.00

ATOM 747 CE LYS A 103 -12.190 8.773 19.407 1.00 1.00

ATOM 748 NZ LYS A 103 -12.729 9.219 20.721 1.00 1.00

ATOM 749 N ILE A 104 -10.197 13.293 14.634 1.00 1.00

ATOM 750 CA ILE A 104 -9.550 14.505 14.141 1.00 1.00

ATOM 751 C ILE A 104 -9.290 15.462 15.301 1.00 1.00

ATOM 752 O ILE A 104 -10.224 15.918 15.963 1.00 1.00

ATOM 753 CB ILE A 104 -10.406 15.196 13.043 1.00 1.00

ATOM 754 CG1 ILE A 104 -10.749 14.219 11.914 1.00 1.00

ATOM 755 CG2 ILE A 104 -9.679 16.427 12.488 1.00 1.00

ATOM 756 CD1 ILE A 104 -12.005 14.584 11.138 1.00 1.00

ATOM 757 N ARG A 105 -8.016 15.752 15.546 1.00 1.00

ATOM 758 CA ARG A 105 -7.632 16.715 16.572 1.00 1.00

ATOM 759 C ARG A 105 -7.489 18.097 15.945 1.00 1.00

ATOM 760 O ARG A 105 -6.650 18.304 15.063 1.00 1.00

ATOM 761 CB ARG A 105 -6.332 16.289 17.258 1.00 1.00

ATOM 762 CG ARG A 105 -6.524 15.267 18.369 1.00 1.00

ATOM 763 CD ARG A 105 -5.234 14.848 19.059 1.00 1.00

ATOM 764 NE ARG A 105 -4.429 13.962 18.219 1.00 1.00

ATOM 765 CZ ARG A 105 -3.262 14.286 17.673 1.00 1.00

ATOM 766 NH1 ARG A 105 -2.735 15.489 17.871 1.00 1.00

ATOM 767 NH2 ARG A 105 -2.616 13.402 16.925 1.00 1.00

ATOM 768 N ALA A 106 -8.321 19.031 16.396 1.00 1.00

ATOM 769 CA ALA A 106 -8.329 20.386 15.852 1.00 1.00

ATOM 770 C ALA A 106 -8.358 21.449 16.943 1.00 1.00

ATOM 771 O ALA A 106 -9.085 21.323 17.931 1.00 1.00

ATOM 772 CB ALA A 106 -9.505 20.570 14.901 1.00 1.00

ATOM 773 N THR A 107 -7.533 22.468 16.765 1.00 1.00

ATOM 774 CA THR A 107 -7.584 23.629 17.663 1.00 1.00

ATOM 775 C THR A 107 -8.496 24.704 17.049 1.00 1.00

ATOM 776 O THR A 107 -8.288 25.151 15.914 1.00 1.00

ATOM 777 CB THR A 107 -6.164 24.186 17.854 1.00 1.00

ATOM 778 OG1 THR A 107 -5.296 23.159 18.314 1.00 1.00

ATOM 779 CG2 THR A 107 -6.093 25.324 18.874 1.00 1.00

ATOM 780 N VAL A 108 -9.496 25.092 17.826 1.00 1.00

ATOM 781 CA VAL A 108 -10.478 26.112 17.408 1.00 1.00

ATOM 782 C VAL A 108 -9.811 27.493 17.414 1.00 1.00

ATOM 783 O VAL A 108 -9.330 27.968 18.453 1.00 1.00

ATOM 784 CB VAL A 108 -11.668 26.104 18.366 1.00 1.00

ATOM 785 CG1 VAL A 108 -12.824 26.983 17.885 1.00 1.00

ATOM 786 CG2 VAL A 108 -12.261 24.704 18.564 1.00 1.00

ATOM 787 N LYS A 109 -9.803 28.105 16.237 1.00 1.00

ATOM 788 CA LYS A 109 -9.169 29.425 16.028 1.00 1.00

ATOM 789 C LYS A 109 -10.176 30.593 16.048 1.00 1.00

ATOM 790 O LYS A 109 -9.808 31.744 16.314 1.00 1.00

ATOM 791 CB LYS A 109 -8.461 29.482 14.669 1.00 1.00

ATOM 792 CG LYS A 109 -7.489 28.341 14.432 1.00 1.00

ATOM 793 CD LYS A 109 -6.058 28.771 14.708 1.00 1.00

ATOM 794 CE LYS A 109 -5.359 29.221 13.435 1.00 1.00

ATOM 795 NZ LYS A 109 -4.039 29.849 13.718 1.00 1.00

ATOM 796 N SER A 110 -11.436 30.302 15.759 1.00 1.00

ATOM 797 CA SER A 110 -12.482 31.354 15.732 1.00 1.00

ATOM 798 C SER A 110 -13.859 30.775 16.071 1.00 1.00

ATOM 799 O SER A 110 -14.041 29.554 16.157 1.00 1.00

ATOM 800 CB SER A 110 -12.542 31.992 14.336 1.00 1.00

ATOM 801 OG SER A 110 -13.792 31.716 13.720 1.00 1.00

ATOM 802 N SER A 111 -14.793 31.707 16.423 1.00 1.00

ATOM 803 CA SER A 111 -16.133 31.351 16.887 1.00 1.00

ATOM 804 C SER A 111 -17.076 30.911 15.763 1.00 1.00

ATOM 805 O SER A 111 -18.142 30.351 16.027 1.00 1.00

ATOM 806 CB SER A 111 -16.751 32.518 17.660 1.00 1.00

ATOM 807 OG SER A 111 -16.996 33.620 16.805 1.00 1.00

ATOM 808 N ALA A 112 -16.678 31.177 14.521 1.00 1.00

ATOM 809 CA ALA A 112 -17.452 30.804 13.339 1.00 1.00

ATOM 810 C ALA A 112 -17.609 29.283 13.227 1.00 1.00

ATOM 811 O ALA A 112 -16.764 28.540 13.737 1.00 1.00

ATOM 812 CB ALA A 112 -16.796 31.370 12.080 1.00 1.00

ATOM 813 N PRO A 113 -18.684 28.818 12.584 1.00 1.00

ATOM 814 CA PRO A 113 -18.892 27.379 12.372 1.00 1.00

ATOM 815 C PRO A 113 -17.728 26.727 11.626 1.00 1.00

ATOM 816 O PRO A 113 -17.291 27.233 10.588 1.00 1.00

ATOM 817 CB PRO A 113 -20.168 27.331 11.525 1.00 1.00

ATOM 818 CG PRO A 113 -20.876 28.604 11.832 1.00 1.00

ATOM 819 CD PRO A 113 -19.794 29.620 12.033 1.00 1.00

ATOM 820 N GLY A 114 -17.226 25.623 12.175 1.00 1.00

ATOM 821 CA GLY A 114 -16.120 24.891 11.585 1.00 1.00

ATOM 822 C GLY A 114 -16.594 23.625 10.905 1.00 1.00

ATOM 823 O GLY A 114 -16.533 22.541 11.485 1.00 1.00

ATOM 824 N TYR A 115 -17.068 23.772 9.669 1.00 1.00

ATOM 825 CA TYR A 115 -17.593 22.656 8.894 1.00 1.00

ATOM 826 C TYR A 115 -16.494 21.693 8.452 1.00 1.00

ATOM 827 O TYR A 115 -15.465 22.112 7.915 1.00 1.00

ATOM 828 CB TYR A 115 -18.350 23.162 7.659 1.00 1.00

ATOM 829 CG TYR A 115 -19.454 24.156 7.948 1.00 1.00

ATOM 830 CD1 TYR A 115 -19.287 25.512 7.667 1.00 1.00

ATOM 831 CD2 TYR A 115 -20.672 23.741 8.487 1.00 1.00

ATOM 832 CE1 TYR A 115 -20.300 26.433 7.926 1.00 1.00

ATOM 833 CE2 TYR A 115 -21.692 24.651 8.752 1.00 1.00

ATOM 834 CZ TYR A 115 -21.500 25.993 8.465 1.00 1.00

ATOM 835 OH TYR A 115 -22.504 26.897 8.723 1.00 1.00

ATOM 836 N ILE A 116 -16.718 20.405 8.701 1.00 1.00

ATOM 837 CA ILE A 116 -15.913 19.343 8.104 1.00 1.00

ATOM 838 C ILE A 116 -16.845 18.493 7.241 1.00 1.00

ATOM 839 O ILE A 116 -17.669 17.728 7.752 1.00 1.00

ATOM 840 CB ILE A 116 -15.175 18.501 9.178 1.00 1.00

ATOM 841 CG1 ILE A 116 -14.211 19.386 9.981 1.00 1.00

ATOM 842 CG2 ILE A 116 -14.408 17.349 8.528 1.00 1.00

ATOM 843 CD1 ILE A 116 -13.829 18.833 11.341 1.00 1.00

ATOM 844 N THR A 117 -16.721 18.660 5.927 1.00 1.00

ATOM 845 CA THR A 117 -17.668 18.089 4.973 1.00 1.00

ATOM 846 C THR A 117 -17.020 17.068 4.046 1.00 1.00

ATOM 847 O THR A 117 -15.827 17.150 3.749 1.00 1.00

ATOM 848 CB THR A 117 -18.327 19.202 4.130 1.00 1.00

ATOM 849 OG1 THR A 117 -17.313 20.025 3.543 1.00 1.00

ATOM 850 CG2 THR A 117 -19.108 20.171 5.014 1.00 1.00

ATOM 851 N PHE A 118 -17.826 16.113 3.589 1.00 1.00

ATOM 852 CA PHE A 118 -17.391 15.104 2.630 1.00 1.00

ATOM 853 C PHE A 118 -17.104 15.744 1.274 1.00 1.00

ATOM 854 O PHE A 118 -17.915 16.518 0.756 1.00 1.00

ATOM 855 CB PHE A 118 -18.456 14.006 2.510 1.00 1.00

ATOM 856 CG PHE A 118 -18.348 13.172 1.258 1.00 1.00

ATOM 857 CD1 PHE A 118 -17.282 12.295 1.074 1.00 1.00

ATOM 858 CD2 PHE A 118 -19.327 13.253 0.270 1.00 1.00

ATOM 859 CE1 PHE A 118 -17.186 11.522 -0.081 1.00 1.00

ATOM 860 CE2 PHE A 118 -19.240 12.483 -0.886 1.00 1.00

ATOM 861 CZ PHE A 118 -18.166 11.615 -1.061 1.00 1.00

ATOM 862 N ASP A 119 -15.944 15.418 0.713 1.00 1.00

ATOM 863 CA ASP A 119 -15.514 15.978 -0.566 1.00 1.00

ATOM 864 C ASP A 119 -15.462 14.911 -1.662 1.00 1.00

ATOM 865 O ASP A 119 -16.107 15.053 -2.702 1.00 1.00

ATOM 866 CB ASP A 119 -14.158 16.683 -0.409 1.00 1.00

ATOM 867 CG ASP A 119 -13.710 17.398 -1.676 1.00 1.00

ATOM 868 OD1 ASP A 119 -14.570 17.895 -2.438 1.00 1.00

ATOM 869 OD2 ASP A 119 -12.507 17.518 -1.988 1.00 1.00

ATOM 870 N GLU A 120 -14.698 13.847 -1.419 1.00 1.00

ATOM 871 CA GLU A 120 -14.505 12.785 -2.402 1.00 1.00

ATOM 872 C GLU A 120 -14.289 11.436 -1.726 1.00 1.00

ATOM 873 O GLU A 120 -13.698 11.365 -0.651 1.00 1.00

ATOM 874 CB GLU A 120 -13.307 13.111 -3.306 1.00 1.00

ATOM 875 CG GLU A 120 -13.209 12.262 -4.568 1.00 1.00

ATOM 876 CD GLU A 120 -12.208 11.122 -4.448 1.00 1.00

ATOM 877 OE1 GLU A 120 -12.066 10.357 -5.427 1.00 1.00

ATOM 878 OE2 GLU A 120 -11.562 10.982 -3.386 1.00 1.00

ATOM 879 N VAL A 121 -14.787 10.376 -2.359 1.00 1.00

ATOM 880 CA VAL A 121 -14.524 9.006 -1.922 1.00 1.00

ATOM 881 C VAL A 121 -13.907 8.196 -3.072 1.00 1.00

ATOM 882 O VAL A 121 -14.329 8.315 -4.224 1.00 1.00

ATOM 883 CB VAL A 121 -15.801 8.317 -1.341 1.00 1.00

ATOM 884 CG1 VAL A 121 -16.883 8.108 -2.411 1.00 1.00

ATOM 885 CG2 VAL A 121 -15.451 7.003 -0.643 1.00 1.00

ATOM 886 N GLY A 122 -12.891 7.399 -2.752 1.00 1.00

ATOM 887 CA GLY A 122 -12.237 6.552 -3.733 1.00 1.00

ATOM 888 C GLY A 122 -12.717 5.119 -3.629 1.00 1.00

ATOM 889 O GLY A 122 -13.844 4.806 -4.012 1.00 1.00

ATOM 890 N GLY A 123 -11.854 4.253 -3.106 1.00 1.00

ATOM 891 CA GLY A 123 -12.196 2.863 -2.862 1.00 1.00

ATOM 892 C GLY A 123 -12.851 2.669 -1.507 1.00 1.00

ATOM 893 O GLY A 123 -12.618 3.447 -0.577 1.00 1.00

ATOM 894 N PHE A 124 -13.677 1.630 -1.406 1.00 1.00

ATOM 895 CA PHE A 124 -14.381 1.276 -0.174 1.00 1.00

ATOM 896 C PHE A 124 -14.875 -0.163 -0.301 1.00 1.00

ATOM 897 O PHE A 124 -16.054 -0.404 -0.563 1.00 1.00

ATOM 898 CB PHE A 124 -15.558 2.231 0.071 1.00 1.00

ATOM 899 CG PHE A 124 -15.754 2.604 1.516 1.00 1.00

ATOM 900 CD1 PHE A 124 -15.490 3.899 1.956 1.00 1.00

ATOM 901 CD2 PHE A 124 -16.218 1.668 2.438 1.00 1.00

ATOM 902 CE1 PHE A 124 -15.670 4.253 3.290 1.00 1.00

ATOM 903 CE2 PHE A 124 -16.403 2.013 3.777 1.00 1.00

ATOM 904 CZ PHE A 124 -16.128 3.309 4.202 1.00 1.00

ATOM 905 N ALA A 125 -13.962 -1.115 -0.116 1.00 1.00

ATOM 906 CA ALA A 125 -14.236 -2.518 -0.423 1.00 1.00

ATOM 907 C ALA A 125 -14.163 -3.452 0.784 1.00 1.00

ATOM 908 O ALA A 125 -13.469 -3.168 1.764 1.00 1.00

ATOM 909 CB ALA A 125 -13.302 -3.006 -1.531 1.00 1.00

ATOM 910 N ASP A 126 -14.885 -4.570 0.693 1.00 1.00

ATOM 911 CA ASP A 126 -14.852 -5.608 1.721 1.00 1.00

ATOM 912 C ASP A 126 -13.692 -6.586 1.508 1.00 1.00

ATOM 913 O ASP A 126 -12.838 -6.363 0.646 1.00 1.00

ATOM 914 CB ASP A 126 -16.202 -6.346 1.812 1.00 1.00

ATOM 915 CG ASP A 126 -16.616 -7.016 0.502 1.00 1.00

ATOM 916 OD1 ASP A 126 -15.743 -7.434 -0.288 1.00 1.00

ATOM 917 OD2 ASP A 126 -17.813 -7.182 0.189 1.00 1.00

ATOM 918 N ASN A 127 -13.729 -7.721 2.215 1.00 1.00

ATOM 919 CA ASN A 127 -12.597 -8.656 2.197 1.00 1.00

ATOM 920 C ASN A 127 -12.572 -9.449 0.879 1.00 1.00

ATOM 921 O ASN A 127 -11.578 -10.107 0.546 1.00 1.00

ATOM 922 CB ASN A 127 -12.710 -9.640 3.362 1.00 1.00

ATOM 923 CG ASN A 127 -11.971 -9.163 4.614 1.00 1.00

ATOM 924 OD1 ASN A 127 -10.893 -8.580 4.509 1.00 1.00

ATOM 925 ND2 ASN A 127 -12.490 -9.380 5.809 1.00 1.00

ATOM 926 N ASP A 128 -13.677 -9.371 0.147 1.00 1.00

ATOM 927 CA ASP A 128 -13.812 -10.073 -1.147 1.00 1.00

ATOM 928 C ASP A 128 -13.547 -9.089 -2.295 1.00 1.00

ATOM 929 O ASP A 128 -13.827 -9.387 -3.471 1.00 1.00

ATOM 930 CB ASP A 128 -15.210 -10.668 -1.277 1.00 1.00

ATOM 931 CG ASP A 128 -15.409 -11.876 -0.354 1.00 1.00

ATOM 932 OD1 ASP A 128 -14.398 -12.609 -0.024 1.00 1.00

ATOM 933 OD2 ASP A 128 -16.582 -12.160 0.094 1.00 1.00

ATOM 934 N LEU A 129 -13.013 -7.953 -1.885 1.00 1.00

ATOM 935 CA LEU A 129 -12.613 -6.860 -2.785 1.00 1.00

ATOM 936 C LEU A 129 -13.829 -6.298 -3.592 1.00 1.00

ATOM 937 O LEU A 129 -13.677 -5.768 -4.707 1.00 1.00

ATOM 938 CB LEU A 129 -11.535 -7.411 -3.734 1.00 1.00

ATOM 939 CG LEU A 129 -10.087 -7.149 -3.327 1.00 1.00

ATOM 940 CD1 LEU A 129 -9.789 -5.659 -3.358 1.00 1.00

ATOM 941 CD2 LEU A 129 -9.823 -7.658 -1.919 1.00 1.00

ATOM 942 N VAL A 130 -15.018 -6.427 -3.003 1.00 1.00

ATOM 943 CA VAL A 130 -16.273 -5.901 -3.605 1.00 1.00

ATOM 944 C VAL A 130 -16.510 -4.491 -3.051 1.00 1.00

ATOM 945 O VAL A 130 -16.544 -4.283 -1.831 1.00 1.00

ATOM 946 CB VAL A 130 -17.476 -6.791 -3.243 1.00 1.00

ATOM 947 CG1 VAL A 130 -18.767 -6.373 -3.962 1.00 1.00

ATOM 948 CG2 VAL A 130 -17.271 -8.264 -3.596 1.00 1.00

ATOM 949 N GLU A 131 -16.667 -3.540 -3.956 1.00 1.00

ATOM 950 CA GLU A 131 -16.888 -2.128 -3.580 1.00 1.00

ATOM 951 C GLU A 131 -18.280 -1.950 -2.964 1.00 1.00

ATOM 952 O GLU A 131 -19.293 -2.367 -3.540 1.00 1.00

ATOM 953 CB GLU A 131 -16.771 -1.222 -4.810 1.00 1.00

ATOM 954 CG GLU A 131 -15.461 -1.421 -5.584 1.00 1.00

ATOM 955 CD GLU A 131 -15.604 -2.347 -6.796 1.00 1.00

ATOM 956 OE1 GLU A 131 -16.534 -3.241 -6.823 1.00 1.00

ATOM 957 OE2 GLU A 131 -14.793 -2.233 -7.794 1.00 1.00

ATOM 958 N GLN A 132 -18.301 -1.340 -1.786 1.00 1.00

ATOM 959 CA GLN A 132 -19.536 -1.222 -0.996 1.00 1.00

ATOM 960 C GLN A 132 -20.203 0.129 -1.227 1.00 1.00

ATOM 961 O GLN A 132 -19.540 1.126 -1.558 1.00 1.00

ATOM 962 CB GLN A 132 -19.227 -1.317 0.511 1.00 1.00

ATOM 963 CG GLN A 132 -18.485 -2.588 0.913 1.00 1.00

ATOM 964 CD GLN A 132 -19.322 -3.847 0.712 1.00 1.00

ATOM 965 OE1 GLN A 132 -20.295 -4.060 1.434 1.00 1.00

ATOM 966 NE2 GLN A 132 -18.992 -4.700 -0.237 1.00 1.00

ATOM 967 N LYS A 133 -21.514 0.143 -1.053 1.00 1.00

ATOM 968 CA LYS A 133 -22.273 1.384 -1.171 1.00 1.00

ATOM 969 C LYS A 133 -21.985 2.183 0.086 1.00 1.00

ATOM 970 O LYS A 133 -22.031 1.651 1.205 1.00 1.00

ATOM 971 CB LYS A 133 -23.760 1.126 -1.287 1.00 1.00

ATOM 972 CG LYS A 133 -24.208 0.578 -2.639 1.00 1.00

ATOM 973 CD LYS A 133 -23.889 -0.901 -2.784 1.00 1.00

ATOM 974 CE LYS A 133 -23.728 -1.291 -4.245 1.00 1.00

ATOM 975 NZ LYS A 133 -22.477 -2.066 -4.477 1.00 1.00

ATOM 976 N VAL A 134 -21.686 3.451 -0.083 1.00 1.00

ATOM 977 CA VAL A 134 -21.339 4.287 1.069 1.00 1.00

ATOM 978 C VAL A 134 -21.912 5.712 0.946 1.00 1.00

ATOM 979 O VAL A 134 -21.946 6.311 -0.138 1.00 1.00

ATOM 980 CB VAL A 134 -19.801 4.342 1.174 1.00 1.00

ATOM 981 CG1 VAL A 134 -19.138 5.184 0.072 1.00 1.00

ATOM 982 CG2 VAL A 134 -19.309 4.931 2.496 1.00 1.00

ATOM 983 N SER A 135 -22.351 6.210 2.097 1.00 1.00

ATOM 984 CA SER A 135 -22.927 7.559 2.228 1.00 1.00

ATOM 985 C SER A 135 -22.251 8.295 3.389 1.00 1.00

ATOM 986 O SER A 135 -21.669 7.670 4.290 1.00 1.00

ATOM 987 CB SER A 135 -24.439 7.481 2.496 1.00 1.00

ATOM 988 OG SER A 135 -24.694 6.787 3.708 1.00 1.00

ATOM 989 N PHE A 136 -22.236 9.619 3.300 1.00 1.00

ATOM 990 CA PHE A 136 -21.495 10.458 4.233 1.00 1.00

ATOM 991 C PHE A 136 -22.388 11.512 4.879 1.00 1.00

ATOM 992 O PHE A 136 -23.202 12.146 4.206 1.00 1.00

ATOM 993 CB PHE A 136 -20.327 11.143 3.515 1.00 1.00

ATOM 994 CG PHE A 136 -19.212 10.209 3.118 1.00 1.00

ATOM 995 CD1 PHE A 136 -19.315 9.419 1.971 1.00 1.00

ATOM 996 CD2 PHE A 136 -18.050 10.133 3.875 1.00 1.00

ATOM 997 CE1 PHE A 136 -18.286 8.557 1.600 1.00 1.00

ATOM 998 CE2 PHE A 136 -17.012 9.276 3.511 1.00 1.00

ATOM 999 CZ PHE A 136 -17.131 8.486 2.370 1.00 1.00

ATOM 1000 N ILE A 137 -22.228 11.691 6.189 1.00 1.00

ATOM 1001 CA ILE A 137 -22.909 12.761 6.918 1.00 1.00

ATOM 1002 C ILE A 137 -21.886 13.792 7.394 1.00 1.00

ATOM 1003 O ILE A 137 -20.936 13.457 8.103 1.00 1.00

ATOM 1004 CB ILE A 137 -23.743 12.195 8.101 1.00 1.00

ATOM 1005 CG1 ILE A 137 -24.949 11.406 7.577 1.00 1.00

ATOM 1006 CG2 ILE A 137 -24.208 13.318 9.037 1.00 1.00

ATOM 1007 CD1 ILE A 137 -25.541 10.425 8.577 1.00 1.00

ATOM 1008 N ASP A 138 -22.089 15.042 6.984 1.00 1.00

ATOM 1009 CA ASP A 138 -21.186 16.141 7.329 1.00 1.00

ATOM 1010 C ASP A 138 -21.325 16.557 8.791 1.00 1.00

ATOM 1011 O ASP A 138 -22.393 16.404 9.388 1.00 1.00

ATOM 1012 CB ASP A 138 -21.434 17.346 6.413 1.00 1.00

ATOM 1013 CG ASP A 138 -21.171 17.039 4.946 1.00 1.00

ATOM 1014 OD1 ASP A 138 -20.499 16.027 4.644 1.00 1.00

ATOM 1015 OD2 ASP A 138 -21.595 17.761 4.020 1.00 1.00

ATOM 1016 N GLY A 139 -20.236 17.079 9.353 1.00 1.00

ATOM 1017 CA GLY A 139 -20.214 17.534 10.733 1.00 1.00

ATOM 1018 C GLY A 139 -19.223 18.659 10.963 1.00 1.00

ATOM 1019 O GLY A 139 -19.099 19.566 10.135 1.00 1.00

ATOM 1020 N GLY A 140 -18.523 18.600 12.093 1.00 1.00

ATOM 1021 CA GLY A 140 -17.502 19.578 12.418 1.00 1.00

ATOM 1022 C GLY A 140 -17.576 20.128 13.831 1.00 1.00

ATOM 1023 O GLY A 140 -18.039 19.450 14.752 1.00 1.00

ATOM 1024 N VAL A 141 -17.111 21.366 13.993 1.00 1.00

ATOM 1025 CA VAL A 141 -17.039 22.028 15.294 1.00 1.00

ATOM 1026 C VAL A 141 -17.916 23.279 15.305 1.00 1.00

ATOM 1027 O VAL A 141 -17.822 24.117 14.404 1.00 1.00

ATOM 1028 CB VAL A 141 -15.578 22.410 15.664 1.00 1.00

ATOM 1029 CG1 VAL A 141 -15.505 23.043 17.049 1.00 1.00

ATOM 1030 CG2 VAL A 141 -14.665 21.195 15.594 1.00 1.00

ATOM 1031 N ASN A 142 -18.760 23.390 16.332 1.00 1.00

ATOM 1032 CA ASN A 142 -19.681 24.519 16.504 1.00 1.00

ATOM 1033 C ASN A 142 -20.561 24.775 15.277 1.00 1.00

ATOM 1034 O ASN A 142 -20.731 25.919 14.845 1.00 1.00

ATOM 1035 CB ASN A 142 -18.922 25.793 16.910 1.00 1.00

ATOM 1036 CG ASN A 142 -18.133 25.620 18.195 1.00 1.00

ATOM 1037 OD1 ASN A 142 -18.637 25.081 19.181 1.00 1.00

ATOM 1038 ND2 ASN A 142 -16.890 26.085 18.192 1.00 1.00

ATOM 1039 N VAL A 143 -21.113 23.697 14.727 1.00 1.00

ATOM 1040 CA VAL A 143 -21.924 23.769 13.508 1.00 1.00

ATOM 1041 C VAL A 143 -23.428 23.658 13.785 1.00 1.00

ATOM 1042 O VAL A 143 -24.244 23.748 12.863 1.00 1.00

ATOM 1043 CB VAL A 143 -21.485 22.708 12.454 1.00 1.00

ATOM 1044 CG1 VAL A 143 -20.057 22.973 11.987 1.00 1.00

ATOM 1045 CG2 VAL A 143 -21.624 21.288 13.001 1.00 1.00

ATOM 1046 N GLY A 144 -23.785 23.466 15.054 1.00 1.00

ATOM 1047 CA GLY A 144 -25.175 23.346 15.457 1.00 1.00

ATOM 1048 C GLY A 144 -25.799 24.685 15.796 1.00 1.00

ATOM 1049 O GLY A 144 -26.886 24.743 16.374 1.00 1.00

ATOM 1050 OXT GLY A 144 -25.186 25.720 15.442 1.00 1.00

TER

ATOM 1051 N GLY B 1 1.469 -2.553 -11.497 1.00 1.00

ATOM 1052 CA GLY B 1 2.214 -2.505 -10.205 1.00 1.00

ATOM 1053 C GLY B 1 3.454 -1.629 -10.250 1.00 1.00

ATOM 1054 O GLY B 1 3.822 -1.010 -9.249 1.00 1.00

ATOM 1055 N ASP B 2 4.093 -1.581 -11.418 1.00 1.00

ATOM 1056 CA ASP B 2 5.325 -0.820 -11.620 1.00 1.00

ATOM 1057 C ASP B 2 5.025 0.669 -11.819 1.00 1.00

ATOM 1058 O ASP B 2 5.047 1.174 -12.944 1.00 1.00

ATOM 1059 CB ASP B 2 6.101 -1.393 -12.814 1.00 1.00

ATOM 1060 CG ASP B 2 7.520 -0.853 -12.918 1.00 1.00

ATOM 1061 OD1 ASP B 2 8.028 -0.263 -11.937 1.00 1.00

ATOM 1062 OD2 ASP B 2 8.209 -0.977 -13.950 1.00 1.00

ATOM 1063 N VAL B 3 4.758 1.358 -10.711 1.00 1.00

ATOM 1064 CA VAL B 3 4.333 2.761 -10.722 1.00 1.00

ATOM 1065 C VAL B 3 5.306 3.690 -11.460 1.00 1.00

ATOM 1066 O VAL B 3 4.906 4.377 -12.401 1.00 1.00

ATOM 1067 CB VAL B 3 4.063 3.290 -9.280 1.00 1.00

ATOM 1068 CG1 VAL B 3 3.623 4.747 -9.304 1.00 1.00

ATOM 1069 CG2 VAL B 3 3.011 2.435 -8.584 1.00 1.00

ATOM 1070 N ASN B 4 6.572 3.702 -11.044 1.00 1.00

ATOM 1071 CA ASN B 4 7.570 4.584 -11.654 1.00 1.00

ATOM 1072 C ASN B 4 8.099 4.099 -13.008 1.00 1.00

ATOM 1073 O ASN B 4 8.706 4.869 -13.755 1.00 1.00

ATOM 1074 CB ASN B 4 8.730 4.881 -10.691 1.00 1.00

ATOM 1075 CG ASN B 4 9.326 3.627 -10.070 1.00 1.00

ATOM 1076 OD1 ASN B 4 9.430 2.581 -10.714 1.00 1.00

ATOM 1077 ND2 ASN B 4 9.725 3.733 -8.809 1.00 1.00

ATOM 1078 N GLY B 5 7.866 2.824 -13.310 1.00 1.00

ATOM 1079 CA GLY B 5 8.232 2.244 -14.591 1.00 1.00

ATOM 1080 C GLY B 5 9.713 1.960 -14.779 1.00 1.00

ATOM 1081 O GLY B 5 10.217 2.041 -15.901 1.00 1.00

ATOM 1082 N ASP B 6 10.408 1.618 -13.694 1.00 1.00

ATOM 1083 CA ASP B 6 11.841 1.327 -13.762 1.00 1.00

ATOM 1084 C ASP B 6 12.163 -0.139 -14.070 1.00 1.00

ATOM 1085 O ASP B 6 13.331 -0.505 -14.223 1.00 1.00

ATOM 1086 CB ASP B 6 12.576 1.813 -12.496 1.00 1.00

ATOM 1087 CG ASP B 6 12.130 1.094 -11.226 1.00 1.00

ATOM 1088 OD1 ASP B 6 11.326 0.139 -11.298 1.00 1.00

ATOM 1089 OD2 ASP B 6 12.536 1.425 -10.092 1.00 1.00

ATOM 1090 N GLY B 7 11.126 -0.970 -14.158 1.00 1.00

ATOM 1091 CA GLY B 7 11.276 -2.370 -14.524 1.00 1.00

ATOM 1092 C GLY B 7 11.162 -3.350 -13.369 1.00 1.00

ATOM 1093 O GLY B 7 11.145 -4.567 -13.583 1.00 1.00

ATOM 1094 N THR B 8 11.083 -2.823 -12.149 1.00 1.00

ATOM 1095 CA THR B 8 10.992 -3.647 -10.948 1.00 1.00

ATOM 1096 C THR B 8 9.838 -3.185 -10.062 1.00 1.00

ATOM 1097 O THR B 8 9.636 -1.983 -9.872 1.00 1.00

ATOM 1098 CB THR B 8 12.320 -3.599 -10.152 1.00 1.00

ATOM 1099 OG1 THR B 8 13.430 -3.721 -11.051 1.00 1.00

ATOM 1100 CG2 THR B 8 12.460 -4.826 -9.255 1.00 1.00

ATOM 1101 N ILE B 9 9.085 -4.142 -9.530 1.00 1.00

ATOM 1102 CA ILE B 9 8.012 -3.843 -8.584 1.00 1.00

ATOM 1103 C ILE B 9 8.520 -4.052 -7.158 1.00 1.00

ATOM 1104 O ILE B 9 8.761 -5.184 -6.732 1.00 1.00

ATOM 1105 CB ILE B 9 6.750 -4.701 -8.867 1.00 1.00

ATOM 1106 CG1 ILE B 9 6.267 -4.498 -10.308 1.00 1.00

ATOM 1107 CG2 ILE B 9 5.631 -4.353 -7.888 1.00 1.00

ATOM 1108 CD1 ILE B 9 5.642 -5.732 -10.936 1.00 1.00

ATOM 1109 N ASN B 10 8.693 -2.946 -6.435 1.00 1.00

ATOM 1110 CA ASN B 10 9.181 -2.980 -5.057 1.00 1.00

ATOM 1111 C ASN B 10 8.486 -1.962 -4.147 1.00 1.00

ATOM 1112 O ASN B 10 7.456 -1.392 -4.516 1.00 1.00

ATOM 1113 CB ASN B 10 10.711 -2.814 -5.009 1.00 1.00

ATOM 1114 CG ASN B 10 11.210 -1.619 -5.813 1.00 1.00

ATOM 1115 OD1 ASN B 10 10.558 -0.576 -5.882 1.00 1.00

ATOM 1116 ND2 ASN B 10 12.380 -1.770 -6.421 1.00 1.00

ATOM 1117 N SER B 11 9.058 -1.743 -2.963 1.00 1.00

ATOM 1118 CA SER B 11 8.487 -0.854 -1.948 1.00 1.00

ATOM 1119 C SER B 11 8.354 0.603 -2.405 1.00 1.00

ATOM 1120 O SER B 11 7.458 1.319 -1.950 1.00 1.00

ATOM 1121 CB SER B 11 9.305 -0.927 -0.657 1.00 1.00

ATOM 1122 OG SER B 11 10.648 -0.538 -0.885 1.00 1.00

ATOM 1123 N THR B 12 9.245 1.026 -3.302 1.00 1.00

ATOM 1124 CA THR B 12 9.226 2.381 -3.860 1.00 1.00

ATOM 1125 C THR B 12 7.933 2.666 -4.635 1.00 1.00

ATOM 1126 O THR B 12 7.433 3.793 -4.622 1.00 1.00

ATOM 1127 CB THR B 12 10.464 2.615 -4.761 1.00 1.00

ATOM 1128 OG1 THR B 12 11.654 2.244 -4.051 1.00 1.00

ATOM 1129 CG2 THR B 12 10.676 4.103 -5.033 1.00 1.00

ATOM 1130 N ASP B 13 7.406 1.641 -5.303 1.00 1.00

ATOM 1131 CA ASP B 13 6.145 1.751 -6.040 1.00 1.00

ATOM 1132 C ASP B 13 4.955 1.931 -5.106 1.00 1.00

ATOM 1133 O ASP B 13 4.091 2.774 -5.353 1.00 1.00

ATOM 1134 CB ASP B 13 5.925 0.524 -6.927 1.00 1.00

ATOM 1135 CG ASP B 13 6.930 0.434 -8.052 1.00 1.00

ATOM 1136 OD1 ASP B 13 6.933 1.321 -8.935 1.00 1.00

ATOM 1137 OD2 ASP B 13 7.768 -0.490 -8.140 1.00 1.00

ATOM 1138 N LEU B 14 4.917 1.133 -4.040 1.00 1.00

ATOM 1139 CA LEU B 14 3.846 1.206 -3.050 1.00 1.00

ATOM 1140 C LEU B 14 3.832 2.561 -2.342 1.00 1.00

ATOM 1141 O LEU B 14 2.766 3.141 -2.135 1.00 1.00

ATOM 1142 CB LEU B 14 3.954 0.059 -2.035 1.00 1.00

ATOM 1143 CG LEU B 14 2.844 -0.085 -0.985 1.00 1.00

ATOM 1144 CD1 LEU B 14 1.503 -0.459 -1.616 1.00 1.00

ATOM 1145 CD2 LEU B 14 3.239 -1.102 0.080 1.00 1.00

ATOM 1146 N THR B 15 5.019 3.054 -1.989 1.00 1.00

ATOM 1147 CA THR B 15 5.175 4.364 -1.357 1.00 1.00

ATOM 1148 C THR B 15 4.638 5.486 -2.249 1.00 1.00

ATOM 1149 O THR B 15 3.935 6.375 -1.771 1.00 1.00

ATOM 1150 CB THR B 15 6.657 4.615 -0.990 1.00 1.00

ATOM 1151 OG1 THR B 15 7.117 3.574 -0.122 1.00 1.00

ATOM 1152 CG2 THR B 15 6.803 5.869 -0.127 1.00 1.00

ATOM 1153 N MET B 16 4.969 5.428 -3.538 1.00 1.00

ATOM 1154 CA MET B 16 4.485 6.402 -4.517 1.00 1.00

ATOM 1155 C MET B 16 2.968 6.328 -4.685 1.00 1.00

ATOM 1156 O MET B 16 2.300 7.357 -4.792 1.00 1.00

ATOM 1157 CB MET B 16 5.163 6.186 -5.871 1.00 1.00

ATOM 1158 CG MET B 16 6.501 6.887 -6.018 1.00 1.00

ATOM 1159 SD MET B 16 7.057 6.916 -7.733 1.00 1.00

ATOM 1160 CE MET B 16 8.830 6.803 -7.498 1.00 1.00

ATOM 1161 N LEU B 17 2.439 5.105 -4.705 1.00 1.00

ATOM 1162 CA LEU B 17 1.005 4.870 -4.848 1.00 1.00

ATOM 1163 C LEU B 17 0.220 5.376 -3.635 1.00 1.00

ATOM 1164 O LEU B 17 -0.810 6.034 -3.792 1.00 1.00

ATOM 1165 CB LEU B 17 0.722 3.379 -5.095 1.00 1.00

ATOM 1166 CG LEU B 17 -0.734 2.895 -5.153 1.00 1.00

ATOM 1167 CD1 LEU B 17 -1.489 3.517 -6.319 1.00 1.00

ATOM 1168 CD2 LEU B 17 -0.796 1.379 -5.234 1.00 1.00

ATOM 1169 N LYS B 18 0.719 5.067 -2.440 1.00 1.00

ATOM 1170 CA LYS B 18 0.078 5.482 -1.192 1.00 1.00

ATOM 1171 C LYS B 18 0.088 7.003 -1.026 1.00 1.00

ATOM 1172 O LYS B 18 -0.906 7.589 -0.593 1.00 1.00

ATOM 1173 CB LYS B 18 0.752 4.812 0.010 1.00 1.00

ATOM 1174 CG LYS B 18 0.330 3.361 0.227 1.00 1.00

ATOM 1175 CD LYS B 18 1.168 2.684 1.301 1.00 1.00

ATOM 1176 CE LYS B 18 0.413 2.593 2.617 1.00 1.00

ATOM 1177 NZ LYS B 18 1.189 1.861 3.662 1.00 1.00

ATOM 1178 N ARG B 19 1.209 7.630 -1.380 1.00 1.00

ATOM 1179 CA ARG B 19 1.345 9.085 -1.324 1.00 1.00

ATOM 1180 C ARG B 19 0.444 9.776 -2.348 1.00 1.00

ATOM 1181 O ARG B 19 -0.057 10.873 -2.099 1.00 1.00

ATOM 1182 CB ARG B 19 2.806 9.502 -1.524 1.00 1.00

ATOM 1183 CG ARG B 19 3.659 9.368 -0.266 1.00 1.00

ATOM 1184 CD ARG B 19 5.163 9.563 -0.485 1.00 1.00

ATOM 1185 NE ARG B 19 5.502 10.896 -0.990 1.00 1.00

ATOM 1186 CZ ARG B 19 5.483 12.017 -0.269 1.00 1.00

ATOM 1187 NH1 ARG B 19 5.137 11.995 1.014 1.00 1.00

ATOM 1188 NH2 ARG B 19 5.812 13.169 -0.835 1.00 1.00

ATOM 1189 N SER B 20 0.239 9.117 -3.489 1.00 1.00

ATOM 1190 CA SER B 20 -0.667 9.603 -4.531 1.00 1.00

ATOM 1191 C SER B 20 -2.125 9.614 -4.065 1.00 1.00

ATOM 1192 O SER B 20 -2.843 10.591 -4.291 1.00 1.00

ATOM 1193 CB SER B 20 -0.519 8.760 -5.805 1.00 1.00

ATOM 1194 OG SER B 20 -1.615 8.954 -6.683 1.00 1.00

ATOM 1195 N VAL B 21 -2.549 8.524 -3.427 1.00 1.00

ATOM 1196 CA VAL B 21 -3.907 8.407 -2.885 1.00 1.00

ATOM 1197 C VAL B 21 -4.119 9.413 -1.747 1.00 1.00

ATOM 1198 O VAL B 21 -5.192 10.007 -1.624 1.00 1.00

ATOM 1199 CB VAL B 21 -4.218 6.961 -2.410 1.00 1.00

ATOM 1200 CG1 VAL B 21 -5.623 6.860 -1.819 1.00 1.00

ATOM 1201 CG2 VAL B 21 -4.066 5.971 -3.559 1.00 1.00

ATOM 1202 N LEU B 22 -3.081 9.615 -0.937 1.00 1.00

ATOM 1203 CA LEU B 22 -3.114 10.609 0.134 1.00 1.00

ATOM 1204 C LEU B 22 -2.924 12.037 -0.388 1.00 1.00

ATOM 1205 O LEU B 22 -2.911 12.994 0.391 1.00 1.00

ATOM 1206 CB LEU B 22 -2.074 10.277 1.212 1.00 1.00

ATOM 1207 CG LEU B 22 -2.445 9.139 2.170 1.00 1.00

ATOM 1208 CD1 LEU B 22 -1.199 8.516 2.778 1.00 1.00

ATOM 1209 CD2 LEU B 22 -3.390 9.622 3.263 1.00 1.00

ATOM 1210 N ARG B 23 -2.781 12.163 -1.709 1.00 1.00

ATOM 1211 CA ARG B 23 -2.681 13.452 -2.410 1.00 1.00

ATOM 1212 C ARG B 23 -1.427 14.264 -2.056 1.00 1.00

ATOM 1213 O ARG B 23 -1.411 15.489 -2.205 1.00 1.00

ATOM 1214 CB ARG B 23 -3.957 14.296 -2.221 1.00 1.00

ATOM 1215 CG ARG B 23 -5.262 13.555 -2.502 1.00 1.00

ATOM 1216 CD ARG B 23 -5.913 13.918 -3.823 1.00 1.00

ATOM 1217 NE ARG B 23 -7.170 13.201 -4.035 1.00 1.00

ATOM 1218 CZ ARG B 23 -8.339 13.786 -4.280 1.00 1.00

ATOM 1219 NH1 ARG B 23 -8.430 15.109 -4.342 1.00 1.00

ATOM 1220 NH2 ARG B 23 -9.425 13.045 -4.460 1.00 1.00

ATOM 1221 N ALA B 24 -0.385 13.579 -1.592 1.00 1.00

ATOM 1222 CA ALA B 24 0.895 14.220 -1.298 1.00 1.00

ATOM 1223 C ALA B 24 1.655 14.521 -2.584 1.00 1.00

ATOM 1224 O ALA B 24 2.342 15.537 -2.691 1.00 1.00

ATOM 1225 CB ALA B 24 1.731 13.347 -0.371 1.00 1.00

ATOM 1226 N ILE B 25 1.518 13.625 -3.559 1.00 1.00

ATOM 1227 CA ILE B 25 2.177 13.759 -4.854 1.00 1.00

ATOM 1228 C ILE B 25 1.179 13.585 -5.994 1.00 1.00

ATOM 1229 O ILE B 25 0.068 13.085 -5.794 1.00 1.00

ATOM 1230 CB ILE B 25 3.333 12.724 -5.001 1.00 1.00

ATOM 1231 CG1 ILE B 25 2.809 11.287 -4.903 1.00 1.00

ATOM 1232 CG2 ILE B 25 4.438 12.980 -3.976 1.00 1.00

ATOM 1233 CD1 ILE B 25 3.423 10.340 -5.916 1.00 1.00

ATOM 1234 N THR B 26 1.581 14.011 -7.187 1.00 1.00

ATOM 1235 CA THR B 26 0.848 13.682 -8.404 1.00 1.00

ATOM 1236 C THR B 26 1.665 12.685 -9.213 1.00 1.00

ATOM 1237 O THR B 26 2.900 12.711 -9.190 1.00 1.00

ATOM 1238 CB THR B 26 0.553 14.936 -9.254 1.00 1.00

ATOM 1239 OG1 THR B 26 1.772 15.637 -9.524 1.00 1.00

ATOM 1240 CG2 THR B 26 -0.284 15.946 -8.469 1.00 1.00

ATOM 1241 N LEU B 27 0.969 11.799 -9.913 1.00 1.00

ATOM 1242 CA LEU B 27 1.621 10.874 -10.827 1.00 1.00

ATOM 1243 C LEU B 27 1.461 11.373 -12.256 1.00 1.00

ATOM 1244 O LEU B 27 0.460 12.014 -12.589 1.00 1.00

ATOM 1245 CB LEU B 27 1.039 9.466 -10.686 1.00 1.00

ATOM 1246 CG LEU B 27 1.189 8.761 -9.333 1.00 1.00

ATOM 1247 CD1 LEU B 27 0.281 7.545 -9.277 1.00 1.00

ATOM 1248 CD2 LEU B 27 2.636 8.365 -9.059 1.00 1.00

ATOM 1249 N THR B 28 2.456 11.086 -13.090 1.00 1.00

ATOM 1250 CA THR B 28 2.384 11.406 -14.513 1.00 1.00

ATOM 1251 C THR B 28 1.353 10.499 -15.181 1.00 1.00

ATOM 1252 O THR B 28 0.953 9.481 -14.610 1.00 1.00

ATOM 1253 CB THR B 28 3.763 11.257 -15.188 1.00 1.00

ATOM 1254 OG1 THR B 28 4.217 9.902 -15.073 1.00 1.00

ATOM 1255 CG2 THR B 28 4.826 12.057 -14.437 1.00 1.00

ATOM 1256 N ASP B 29 0.926 10.874 -16.383 1.00 1.00

ATOM 1257 CA ASP B 29 -0.131 10.148 -17.090 1.00 1.00

ATOM 1258 C ASP B 29 0.221 8.689 -17.377 1.00 1.00

ATOM 1259 O ASP B 29 -0.645 7.819 -17.296 1.00 1.00

ATOM 1260 CB ASP B 29 -0.535 10.890 -18.368 1.00 1.00

ATOM 1261 CG ASP B 29 -1.193 12.233 -18.080 1.00 1.00

ATOM 1262 OD1 ASP B 29 -1.850 12.367 -17.026 1.00 1.00

ATOM 1263 OD2 ASP B 29 -1.107 13.216 -18.847 1.00 1.00

ATOM 1264 N ASP B 30 1.491 8.427 -17.691 1.00 1.00

ATOM 1265 CA ASP B 30 1.983 7.059 -17.873 1.00 1.00

ATOM 1266 C ASP B 30 1.934 6.272 -16.567 1.00 1.00

ATOM 1267 O ASP B 30 1.493 5.119 -16.546 1.00 1.00

ATOM 1268 CB ASP B 30 3.423 7.062 -18.391 1.00 1.00

ATOM 1269 CG ASP B 30 3.533 7.487 -19.842 1.00 1.00

ATOM 1270 OD1 ASP B 30 2.500 7.735 -20.500 1.00 1.00

ATOM 1271 OD2 ASP B 30 4.638 7.608 -20.404 1.00 1.00

ATOM 1272 N ALA B 31 2.403 6.903 -15.492 1.00 1.00

ATOM 1273 CA ALA B 31 2.444 6.287 -14.165 1.00 1.00

ATOM 1274 C ALA B 31 1.047 5.999 -13.624 1.00 1.00

ATOM 1275 O ALA B 31 0.845 5.010 -12.918 1.00 1.00

ATOM 1276 CB ALA B 31 3.220 7.168 -13.195 1.00 1.00

ATOM 1277 N LYS B 32 0.092 6.864 -13.966 1.00 1.00

ATOM 1278 CA LYS B 32 -1.305 6.711 -13.560 1.00 1.00

ATOM 1279 C LYS B 32 -1.909 5.387 -14.032 1.00 1.00

ATOM 1280 O LYS B 32 -2.629 4.725 -13.279 1.00 1.00

ATOM 1281 CB LYS B 32 -2.142 7.883 -14.083 1.00 1.00

ATOM 1282 CG LYS B 32 -2.111 9.121 -13.201 1.00 1.00

ATOM 1283 CD LYS B 32 -3.170 10.124 -13.640 1.00 1.00

ATOM 1284 CE LYS B 32 -3.023 11.440 -12.896 1.00 1.00

ATOM 1285 NZ LYS B 32 -3.042 12.602 -13.830 1.00 1.00

ATOM 1286 N ALA B 33 -1.611 5.012 -15.274 1.00 1.00

ATOM 1287 CA ALA B 33 -2.099 3.758 -15.849 1.00 1.00

ATOM 1288 C ALA B 33 -1.502 2.540 -15.145 1.00 1.00

ATOM 1289 O ALA B 33 -2.198 1.551 -14.906 1.00 1.00

ATOM 1290 CB ALA B 33 -1.810 3.710 -17.347 1.00 1.00

ATOM 1291 N ARG B 34 -0.215 2.625 -14.807 1.00 1.00

ATOM 1292 CA ARG B 34 0.493 1.541 -14.127 1.00 1.00

ATOM 1293 C ARG B 34 0.120 1.442 -12.644 1.00 1.00

ATOM 1294 O ARG B 34 0.188 0.360 -12.054 1.00 1.00

ATOM 1295 CB ARG B 34 2.010 1.701 -14.286 1.00 1.00

ATOM 1296 CG ARG B 34 2.501 1.704 -15.735 1.00 1.00

ATOM 1297 CD ARG B 34 4.015 1.806 -15.896 1.00 1.00

ATOM 1298 NE ARG B 34 4.568 2.985 -15.227 1.00 1.00

ATOM 1299 CZ ARG B 34 5.129 4.016 -15.852 1.00 1.00

ATOM 1300 NH1 ARG B 34 5.224 4.032 -17.177 1.00 1.00

ATOM 1301 NH2 ARG B 34 5.600 5.037 -15.150 1.00 1.00

ATOM 1302 N ALA B 35 -0.265 2.570 -12.052 1.00 1.00

ATOM 1303 CA ALA B 35 -0.716 2.614 -10.660 1.00 1.00

ATOM 1304 C ALA B 35 -2.133 2.059 -10.501 1.00 1.00

ATOM 1305 O ALA B 35 -2.518 1.619 -9.415 1.00 1.00

ATOM 1306 CB ALA B 35 -0.642 4.034 -10.121 1.00 1.00

ATOM 1307 N ASP B 36 -2.902 2.094 -11.587 1.00 1.00

ATOM 1308 CA ASP B 36 -4.253 1.542 -11.614 1.00 1.00

ATOM 1309 C ASP B 36 -4.184 0.013 -11.679 1.00 1.00

ATOM 1310 O ASP B 36 -4.362 -0.584 -12.743 1.00 1.00

ATOM 1311 CB ASP B 36 -5.028 2.118 -12.806 1.00 1.00

ATOM 1312 CG ASP B 36 -6.533 1.891 -12.710 1.00 1.00

ATOM 1313 OD1 ASP B 36 -7.005 1.247 -11.747 1.00 1.00

ATOM 1314 OD2 ASP B 36 -7.326 2.326 -13.567 1.00 1.00

ATOM 1315 N VAL B 37 -3.921 -0.604 -10.527 1.00 1.00

ATOM 1316 CA VAL B 37 -3.674 -2.047 -10.427 1.00 1.00

ATOM 1317 C VAL B 37 -4.850 -2.895 -10.931 1.00 1.00

ATOM 1318 O VAL B 37 -4.651 -3.831 -11.708 1.00 1.00

ATOM 1319 CB VAL B 37 -3.281 -2.466 -8.978 1.00 1.00

ATOM 1320 CG1 VAL B 37 -3.135 -3.984 -8.854 1.00 1.00

ATOM 1321 CG2 VAL B 37 -1.991 -1.777 -8.544 1.00 1.00

ATOM 1322 N ASP B 38 -6.063 -2.558 -10.496 1.00 1.00

ATOM 1323 CA ASP B 38 -7.255 -3.324 -10.879 1.00 1.00

ATOM 1324 C ASP B 38 -7.926 -2.816 -12.159 1.00 1.00

ATOM 1325 O ASP B 38 -8.966 -3.333 -12.572 1.00 1.00

ATOM 1326 CB ASP B 38 -8.257 -3.408 -9.715 1.00 1.00

ATOM 1327 CG ASP B 38 -8.930 -4.773 -9.600 1.00 1.00

ATOM 1328 OD1 ASP B 38 -8.383 -5.778 -10.106 1.00 1.00

ATOM 1329 OD2 ASP B 38 -10.020 -4.937 -9.011 1.00 1.00

ATOM 1330 N LYS B 39 -7.374 -1.799 -12.759 1.00 1.00

ATOM 1331 CA LYS B 39 -7.717 -1.190 -14.051 1.00 1.00

ATOM 1332 C LYS B 39 -9.213 -0.828 -14.102 1.00 1.00

ATOM 1333 O LYS B 39 -9.950 -1.259 -15.004 1.00 1.00

ATOM 1334 CB LYS B 39 -7.421 -2.177 -15.191 1.00 1.00

ATOM 1335 CG LYS B 39 -5.930 -2.456 -15.374 1.00 1.00

ATOM 1336 CD LYS B 39 -5.205 -1.333 -16.112 1.00 1.00

ATOM 1337 CE LYS B 39 -3.692 -1.360 -15.900 1.00 1.00

ATOM 1338 NZ LYS B 39 -2.982 -0.392 -16.746 1.00 1.00

ATOM 1339 N ASN B 40 -9.661 -0.031 -13.133 1.00 1.00

ATOM 1340 CA ASN B 40 -11.081 0.372 -13.093 1.00 1.00

ATOM 1341 C ASN B 40 -11.248 1.889 -13.378 1.00 1.00

ATOM 1342 O ASN B 40 -12.346 2.447 -13.248 1.00 1.00

ATOM 1343 CB ASN B 40 -11.726 0.019 -11.741 1.00 1.00

ATOM 1344 CG ASN B 40 -11.184 0.780 -10.536 1.00 1.00

ATOM 1345 OD1 ASN B 40 -10.125 1.396 -10.620 1.00 1.00

ATOM 1346 ND2 ASN B 40 -11.867 0.765 -9.406 1.00 1.00

ATOM 1347 N GLY B 41 -10.149 2.525 -13.770 1.00 1.00

ATOM 1348 CA GLY B 41 -10.149 3.962 -14.163 1.00 1.00

ATOM 1349 C GLY B 41 -9.879 4.943 -13.010 1.00 1.00

ATOM 1350 O GLY B 41 -9.905 6.168 -13.206 1.00 1.00

ATOM 1351 N SER B 42 -9.624 4.408 -11.836 1.00 1.00

ATOM 1352 CA SER B 42 -9.347 5.238 -10.640 1.00 1.00

ATOM 1353 C SER B 42 -8.186 4.691 -9.844 1.00 1.00

ATOM 1354 O SER B 42 -7.961 3.481 -9.800 1.00 1.00

ATOM 1355 CB SER B 42 -10.543 5.251 -9.706 1.00 1.00

ATOM 1356 OG SER B 42 -11.601 6.048 -10.224 1.00 1.00

ATOM 1357 N ILE B 43 -7.465 5.600 -9.219 1.00 1.00

ATOM 1358 CA ILE B 43 -6.325 5.229 -8.378 1.00 1.00

ATOM 1359 C ILE B 43 -6.664 5.506 -6.909 1.00 1.00

ATOM 1360 O ILE B 43 -6.910 6.653 -6.509 1.00 1.00

ATOM 1361 CB ILE B 43 -5.076 6.018 -8.766 1.00 1.00

ATOM 1362 CG1 ILE B 43 -4.526 5.610 -10.137 1.00 1.00

ATOM 1363 CG2 ILE B 43 -3.926 5.823 -7.771 1.00 1.00

ATOM 1364 CD1 ILE B 43 -4.215 6.800 -11.046 1.00 1.00

ATOM 1365 N ASN B 44 -6.864 4.408 -6.167 1.00 1.00

ATOM 1366 CA ASN B 44 -7.379 4.469 -4.799 1.00 1.00

ATOM 1367 C ASN B 44 -6.844 3.377 -3.861 1.00 1.00

ATOM 1368 O ASN B 44 -5.885 2.675 -4.196 1.00 1.00

ATOM 1369 CB ASN B 44 -8.921 4.491 -4.806 1.00 1.00

ATOM 1370 CG ASN B 44 -9.537 3.254 -5.456 1.00 1.00

ATOM 1371 OD1 ASN B 44 -9.050 2.137 -5.288 1.00 1.00

ATOM 1372 ND2 ASN B 44 -10.624 3.453 -6.186 1.00 1.00

ATOM 1373 N ALA B 45 -7.478 3.245 -2.696 1.00 1.00

ATOM 1374 CA ALA B 45 -7.060 2.305 -1.649 1.00 1.00

ATOM 1375 C ALA B 45 -7.169 0.828 -2.041 1.00 1.00

ATOM 1376 O ALA B 45 -6.505 -0.022 -1.439 1.00 1.00

ATOM 1377 CB ALA B 45 -7.846 2.558 -0.357 1.00 1.00

ATOM 1378 N ALA B 46 -8.009 0.521 -3.028 1.00 1.00

ATOM 1379 CA ALA B 46 -8.127 -0.846 -3.540 1.00 1.00

ATOM 1380 C ALA B 46 -6.849 -1.256 -4.275 1.00 1.00

ATOM 1381 O ALA B 46 -6.384 -2.389 -4.125 1.00 1.00

ATOM 1382 CB ALA B 46 -9.374 -1.001 -4.443 1.00 1.00

ATOM 1383 N ASP B 47 -6.287 -0.330 -5.054 1.00 1.00

ATOM 1384 CA ASP B 47 -4.992 -0.531 -5.707 1.00 1.00

ATOM 1385 C ASP B 47 -3.876 -0.692 -4.680 1.00 1.00

ATOM 1386 O ASP B 47 -2.986 -1.529 -4.846 1.00 1.00

ATOM 1387 CB ASP B 47 -4.652 0.642 -6.631 1.00 1.00

ATOM 1388 CG ASP B 47 -5.722 0.900 -7.667 1.00 1.00

ATOM 1389 OD1 ASP B 47 -6.011 -0.007 -8.478 1.00 1.00

ATOM 1390 OD2 ASP B 47 -6.334 1.987 -7.752 1.00 1.00

ATOM 1391 N VAL B 48 -3.937 0.123 -3.625 1.00 1.00

ATOM 1392 CA VAL B 48 -2.979 0.074 -2.521 1.00 1.00

ATOM 1393 C VAL B 48 -2.974 -1.312 -1.870 1.00 1.00

ATOM 1394 O VAL B 48 -1.912 -1.912 -1.692 1.00 1.00

ATOM 1395 CB VAL B 48 -3.275 1.174 -1.460 1.00 1.00

ATOM 1396 CG1 VAL B 48 -2.434 0.970 -0.203 1.00 1.00

ATOM 1397 CG2 VAL B 48 -3.034 2.563 -2.040 1.00 1.00

ATOM 1398 N LEU B 49 -4.162 -1.812 -1.539 1.00 1.00

ATOM 1399 CA LEU B 49 -4.316 -3.128 -0.922 1.00 1.00

ATOM 1400 C LEU B 49 -3.737 -4.241 -1.800 1.00 1.00

ATOM 1401 O LEU B 49 -3.001 -5.101 -1.311 1.00 1.00

ATOM 1402 CB LEU B 49 -5.789 -3.407 -0.602 1.00 1.00

ATOM 1403 CG LEU B 49 -6.116 -4.628 0.266 1.00 1.00

ATOM 1404 CD1 LEU B 49 -5.980 -4.306 1.747 1.00 1.00

ATOM 1405 CD2 LEU B 49 -7.517 -5.141 -0.041 1.00 1.00

ATOM 1406 N LEU B 50 -4.062 -4.202 -3.089 1.00 1.00

ATOM 1407 CA LEU B 50 -3.622 -5.224 -4.040 1.00 1.00

ATOM 1408 C LEU B 50 -2.106 -5.241 -4.243 1.00 1.00

ATOM 1409 O LEU B 50 -1.497 -6.314 -4.276 1.00 1.00

ATOM 1410 CB LEU B 50 -4.340 -5.064 -5.384 1.00 1.00

ATOM 1411 CG LEU B 50 -5.867 -5.201 -5.376 1.00 1.00

ATOM 1412 CD1 LEU B 50 -6.399 -5.283 -6.799 1.00 1.00

ATOM 1413 CD2 LEU B 50 -6.326 -6.407 -4.557 1.00 1.00

ATOM 1414 N LEU B 51 -1.504 -4.060 -4.371 1.00 1.00

ATOM 1415 CA LEU B 51 -0.055 -3.948 -4.544 1.00 1.00

ATOM 1416 C LEU B 51 0.712 -4.379 -3.289 1.00 1.00

ATOM 1417 O LEU B 51 1.734 -5.061 -3.391 1.00 1.00

ATOM 1418 CB LEU B 51 0.350 -2.528 -4.973 1.00 1.00

ATOM 1419 CG LEU B 51 1.833 -2.242 -5.257 1.00 1.00

ATOM 1420 CD1 LEU B 51 2.429 -3.224 -6.265 1.00 1.00

ATOM 1421 CD2 LEU B 51 2.032 -0.814 -5.733 1.00 1.00

ATOM 1422 N SER B 52 0.216 -3.978 -2.118 1.00 1.00

ATOM 1423 CA SER B 52 0.840 -4.342 -0.846 1.00 1.00

ATOM 1424 C SER B 52 0.806 -5.852 -0.598 1.00 1.00

ATOM 1425 O SER B 52 1.757 -6.418 -0.058 1.00 1.00

ATOM 1426 CB SER B 52 0.191 -3.590 0.322 1.00 1.00

ATOM 1427 OG SER B 52 -1.146 -4.010 0.534 1.00 1.00

ATOM 1428 N ARG B 53 -0.293 -6.488 -1.004 1.00 1.00

ATOM 1429 CA ARG B 53 -0.442 -7.940 -0.910 1.00 1.00

ATOM 1430 C ARG B 53 0.484 -8.668 -1.885 1.00 1.00

ATOM 1431 O ARG B 53 1.006 -9.739 -1.569 1.00 1.00

ATOM 1432 CB ARG B 53 -1.897 -8.351 -1.153 1.00 1.00

ATOM 1433 CG ARG B 53 -2.858 -7.847 -0.089 1.00 1.00

ATOM 1434 CD ARG B 53 -4.269 -8.396 -0.205 1.00 1.00

ATOM 1435 NE ARG B 53 -4.371 -9.745 0.353 1.00 1.00

ATOM 1436 CZ ARG B 53 -5.184 -10.692 -0.102 1.00 1.00

ATOM 1437 NH1 ARG B 53 -5.987 -10.457 -1.134 1.00 1.00

ATOM 1438 NH2 ARG B 53 -5.197 -11.883 0.479 1.00 1.00

ATOM 1439 N TYR B 54 0.682 -8.078 -3.064 1.00 1.00

ATOM 1440 CA TYR B 54 1.584 -8.624 -4.077 1.00 1.00

ATOM 1441 C TYR B 54 3.031 -8.651 -3.579 1.00 1.00

ATOM 1442 O TYR B 54 3.756 -9.620 -3.813 1.00 1.00

ATOM 1443 CB TYR B 54 1.480 -7.825 -5.384 1.00 1.00

ATOM 1444 CG TYR B 54 2.460 -8.254 -6.458 1.00 1.00

ATOM 1445 CD1 TYR B 54 2.187 -9.341 -7.287 1.00 1.00

ATOM 1446 CD2 TYR B 54 3.665 -7.569 -6.644 1.00 1.00

ATOM 1447 CE1 TYR B 54 3.087 -9.739 -8.273 1.00 1.00

ATOM 1448 CE2 TYR B 54 4.571 -7.959 -7.626 1.00 1.00

ATOM 1449 CZ TYR B 54 4.274 -9.043 -8.436 1.00 1.00

ATOM 1450 OH TYR B 54 5.167 -9.433 -9.410 1.00 1.00

ATOM 1451 N LEU B 55 3.438 -7.585 -2.893 1.00 1.00

ATOM 1452 CA LEU B 55 4.793 -7.472 -2.357 1.00 1.00

ATOM 1453 C LEU B 55 5.006 -8.358 -1.129 1.00 1.00

ATOM 1454 O LEU B 55 6.132 -8.769 -0.842 1.00 1.00

ATOM 1455 CB LEU B 55 5.127 -6.013 -2.026 1.00 1.00

ATOM 1456 CG LEU B 55 5.140 -5.002 -3.178 1.00 1.00

ATOM 1457 CD1 LEU B 55 5.093 -3.579 -2.643 1.00 1.00

ATOM 1458 CD2 LEU B 55 6.351 -5.196 -4.083 1.00 1.00

ATOM 1459 N LEU B 56 3.921 -8.650 -0.414 1.00 1.00

ATOM 1460 CA LEU B 56 3.974 -9.493 0.779 1.00 1.00

ATOM 1461 C LEU B 56 3.684 -10.955 0.442 1.00 1.00

ATOM 1462 O LEU B 56 4.268 -11.524 -0.482 1.00 1.00

ATOM 1463 CB LEU B 56 2.989 -8.990 1.836 1.00 1.00

ATOM 1464 CG LEU B 56 3.526 -7.952 2.823 1.00 1.00

ATOM 1465 CD1 LEU B 56 4.704 -8.512 3.605 1.00 1.00

ATOM 1466 CD2 LEU B 56 3.993 -6.705 2.089 1.00 1.00

ATOM 1467 OXT LEU B 56 2.810 -11.547 1.119 1.00 1.00

ATOM 1468 CA CA C 160 9.197 0.279 -10.001 1.00 1.00

ATOM 1469 CA CA D 161 -7.996 1.263 -9.567 1.00 1.00

TER

ENDMDL

MODEL 6

ATOM 0 N GLY A 5 -25.852 0.602 2.639 1.00 1.00

ATOM 1 CA GLY A 5 -24.451 0.107 2.790 1.00 1.00

ATOM 2 C GLY A 5 -23.739 0.723 3.982 1.00 1.00

ATOM 3 O GLY A 5 -24.291 0.780 5.087 1.00 1.00

ATOM 4 N VAL A 6 -22.509 1.183 3.762 1.00 1.00

ATOM 5 CA VAL A 6 -21.710 1.797 4.825 1.00 1.00

ATOM 6 C VAL A 6 -22.110 3.262 5.034 1.00 1.00

ATOM 7 O VAL A 6 -22.338 3.994 4.072 1.00 1.00

ATOM 8 CB VAL A 6 -20.180 1.692 4.547 1.00 1.00

ATOM 9 CG1 VAL A 6 -19.371 2.339 5.668 1.00 1.00

ATOM 10 CG2 VAL A 6 -19.759 0.237 4.365 1.00 1.00

ATOM 11 N VAL A 7 -22.207 3.674 6.297 1.00 1.00

ATOM 12 CA VAL A 7 -22.502 5.062 6.636 1.00 1.00

ATOM 13 C VAL A 7 -21.323 5.650 7.406 1.00 1.00

ATOM 14 O VAL A 7 -20.906 5.114 8.430 1.00 1.00

ATOM 15 CB VAL A 7 -23.820 5.205 7.453 1.00 1.00

ATOM 16 CG1 VAL A 7 -24.054 6.664 7.860 1.00 1.00

ATOM 17 CG2 VAL A 7 -25.017 4.688 6.653 1.00 1.00

ATOM 18 N VAL A 8 -20.778 6.747 6.892 1.00 1.00

ATOM 19 CA VAL A 8 -19.680 7.436 7.559 1.00 1.00

ATOM 20 C VAL A 8 -20.211 8.748 8.139 1.00 1.00

ATOM 21 O VAL A 8 -20.645 9.633 7.395 1.00 1.00

ATOM 22 CB VAL A 8 -18.502 7.705 6.593 1.00 1.00

ATOM 23 CG1 VAL A 8 -17.332 8.347 7.328 1.00 1.00

ATOM 24 CG2 VAL A 8 -18.062 6.416 5.891 1.00 1.00

ATOM 25 N GLU A 9 -20.183 8.862 9.465 1.00 1.00

ATOM 26 CA GLU A 9 -20.681 10.061 10.127 1.00 1.00

ATOM 27 C GLU A 9 -19.566 10.845 10.807 1.00 1.00

ATOM 28 O GLU A 9 -18.832 10.305 11.637 1.00 1.00

ATOM 29 CB GLU A 9 -21.795 9.731 11.128 1.00 1.00

ATOM 30 CG GLU A 9 -22.374 10.973 11.812 1.00 1.00

ATOM 31 CD GLU A 9 -23.679 10.722 12.555 1.00 1.00

ATOM 32 OE1 GLU A 9 -24.263 9.619 12.432 1.00 1.00

ATOM 33 OE2 GLU A 9 -24.125 11.648 13.266 1.00 1.00

ATOM 34 N ILE A 10 -19.448 12.118 10.430 1.00 1.00

ATOM 35 CA ILE A 10 -18.557 13.059 11.101 1.00 1.00

ATOM 36 C ILE A 10 -19.280 13.611 12.331 1.00 1.00

ATOM 37 O ILE A 10 -20.375 14.163 12.220 1.00 1.00

ATOM 38 CB ILE A 10 -18.131 14.217 10.151 1.00 1.00

ATOM 39 CG1 ILE A 10 -17.372 13.661 8.938 1.00 1.00

ATOM 40 CG2 ILE A 10 -17.282 15.264 10.896 1.00 1.00

ATOM 41 CD1 ILE A 10 -17.525 14.487 7.668 1.00 1.00

ATOM 42 N GLY A 11 -18.665 13.438 13.498 1.00 1.00

ATOM 43 CA GLY A 11 -19.239 13.905 14.765 1.00 1.00

ATOM 44 C GLY A 11 -19.423 15.414 14.823 1.00 1.00

ATOM 45 O GLY A 11 -18.806 16.158 14.053 1.00 1.00

ATOM 46 N LYS A 12 -20.287 15.859 15.729 1.00 1.00

ATOM 47 CA LYS A 12 -20.515 17.283 15.951 1.00 1.00

ATOM 48 C LYS A 12 -20.124 17.627 17.379 1.00 1.00

ATOM 49 O LYS A 12 -20.629 17.028 18.328 1.00 1.00

ATOM 50 CB LYS A 12 -21.972 17.665 15.679 1.00 1.00

ATOM 51 CG LYS A 12 -22.398 17.517 14.222 1.00 1.00

ATOM 52 CD LYS A 12 -23.900 17.714 14.077 1.00 1.00

ATOM 53 CE LYS A 12 -24.371 17.459 12.652 1.00 1.00

ATOM 54 NZ LYS A 12 -24.175 16.040 12.233 1.00 1.00

ATOM 55 N VAL A 13 -19.197 18.573 17.521 1.00 1.00

ATOM 56 CA VAL A 13 -18.660 18.944 18.830 1.00 1.00

ATOM 57 C VAL A 13 -18.688 20.459 19.028 1.00 1.00

ATOM 58 O VAL A 13 -18.928 21.216 18.085 1.00 1.00

ATOM 59 CB VAL A 13 -17.206 18.413 19.052 1.00 1.00

ATOM 60 CG1 VAL A 13 -17.164 16.886 19.002 1.00 1.00

ATOM 61 CG2 VAL A 13 -16.240 19.016 18.037 1.00 1.00

ATOM 62 N THR A 14 -18.454 20.886 20.263 1.00 1.00

ATOM 63 CA THR A 14 -18.413 22.299 20.604 1.00 1.00

ATOM 64 C THR A 14 -17.088 22.604 21.300 1.00 1.00

ATOM 65 O THR A 14 -16.442 21.700 21.838 1.00 1.00

ATOM 66 CB THR A 14 -19.595 22.709 21.522 1.00 1.00

ATOM 67 OG1 THR A 14 -19.572 21.918 22.715 1.00 1.00

ATOM 68 CG2 THR A 14 -20.933 22.510 20.814 1.00 1.00

ATOM 69 N GLY A 15 -16.687 23.869 21.282 1.00 1.00

ATOM 70 CA GLY A 15 -15.458 24.294 21.947 1.00 1.00

ATOM 71 C GLY A 15 -15.190 25.774 21.783 1.00 1.00

ATOM 72 O GLY A 15 -15.469 26.354 20.730 1.00 1.00

ATOM 73 N SER A 16 -14.649 26.382 22.837 1.00 1.00

ATOM 74 CA SER A 16 -14.279 27.794 22.822 1.00 1.00

ATOM 75 C SER A 16 -13.024 28.039 21.986 1.00 1.00

ATOM 76 O SER A 16 -12.210 27.134 21.788 1.00 1.00

ATOM 77 CB SER A 16 -14.056 28.297 24.253 1.00 1.00

ATOM 78 OG SER A 16 -15.227 28.129 25.033 1.00 1.00

ATOM 79 N VAL A 17 -12.886 29.271 21.492 1.00 1.00

ATOM 80 CA VAL A 17 -11.693 29.701 20.764 1.00 1.00

ATOM 81 C VAL A 17 -10.432 29.445 21.591 1.00 1.00

ATOM 82 O VAL A 17 -10.373 29.786 22.777 1.00 1.00

ATOM 83 CB VAL A 17 -11.780 31.205 20.371 1.00 1.00

ATOM 84 CG1 VAL A 17 -10.460 31.712 19.793 1.00 1.00

ATOM 85 CG2 VAL A 17 -12.918 31.435 19.384 1.00 1.00

ATOM 86 N GLY A 18 -9.435 28.829 20.961 1.00 1.00

ATOM 87 CA GLY A 18 -8.157 28.551 21.612 1.00 1.00

ATOM 88 C GLY A 18 -8.031 27.170 22.223 1.00 1.00

ATOM 89 O GLY A 18 -6.936 26.771 22.621 1.00 1.00

ATOM 90 N THR A 19 -9.143 26.441 22.308 1.00 1.00

ATOM 91 CA THR A 19 -9.137 25.084 22.864 1.00 1.00

ATOM 92 C THR A 19 -8.964 24.024 21.773 1.00 1.00

ATOM 93 O THR A 19 -9.195 24.288 20.590 1.00 1.00

ATOM 94 CB THR A 19 -10.419 24.762 23.682 1.00 1.00

ATOM 95 OG1 THR A 19 -11.545 24.653 22.803 1.00 1.00

ATOM 96 CG2 THR A 19 -10.688 25.826 24.737 1.00 1.00

ATOM 97 N THR A 20 -8.552 22.829 22.188 1.00 1.00

ATOM 98 CA THR A 20 -8.439 21.692 21.284 1.00 1.00

ATOM 99 C THR A 20 -9.671 20.806 21.440 1.00 1.00

ATOM 100 O THR A 20 -10.119 20.538 22.558 1.00 1.00

ATOM 101 CB THR A 20 -7.155 20.868 21.552 1.00 1.00

ATOM 102 OG1 THR A 20 -6.028 21.747 21.657 1.00 1.00

ATOM 103 CG2 THR A 20 -6.900 19.888 20.421 1.00 1.00

ATOM 104 N VAL A 21 -10.220 20.377 20.307 1.00 1.00

ATOM 105 CA VAL A 21 -11.368 19.470 20.281 1.00 1.00

ATOM 106 C VAL A 21 -11.039 18.205 19.490 1.00 1.00

ATOM 107 O VAL A 21 -10.125 18.207 18.662 1.00 1.00

ATOM 108 CB VAL A 21 -12.647 20.149 19.688 1.00 1.00

ATOM 109 CG1 VAL A 21 -13.181 21.228 20.625 1.00 1.00

ATOM 110 CG2 VAL A 21 -12.377 20.723 18.297 1.00 1.00

ATOM 111 N GLU A 22 -11.775 17.129 19.764 1.00 1.00

ATOM 112 CA GLU A 22 -11.669 15.892 18.997 1.00 1.00

ATOM 113 C GLU A 22 -12.958 15.610 18.233 1.00 1.00

ATOM 114 O GLU A 22 -14.026 15.480 18.834 1.00 1.00

ATOM 115 CB GLU A 22 -11.328 14.697 19.895 1.00 1.00

ATOM 116 CG GLU A 22 -9.859 14.566 20.219 1.00 1.00

ATOM 117 CD GLU A 22 -9.403 13.118 20.381 1.00 1.00

ATOM 118 OE1 GLU A 22 -10.206 12.263 20.816 1.00 1.00

ATOM 119 OE2 GLU A 22 -8.224 12.842 20.075 1.00 1.00

ATOM 120 N ILE A 23 -12.842 15.518 16.912 1.00 1.00

ATOM 121 CA ILE A 23 -13.987 15.227 16.051 1.00 1.00

ATOM 122 C ILE A 23 -13.899 13.777 15.570 1.00 1.00

ATOM 123 O ILE A 23 -13.076 13.457 14.712 1.00 1.00

ATOM 124 CB ILE A 23 -14.085 16.186 14.828 1.00 1.00

ATOM 125 CG1 ILE A 23 -13.954 17.658 15.247 1.00 1.00

ATOM 126 CG2 ILE A 23 -15.390 15.958 14.068 1.00 1.00

ATOM 127 CD1 ILE A 23 -12.546 18.217 15.119 1.00 1.00

ATOM 128 N PRO A 24 -14.742 12.894 16.134 1.00 1.00

ATOM 129 CA PRO A 24 -14.700 11.486 15.737 1.00 1.00

ATOM 130 C PRO A 24 -15.362 11.231 14.386 1.00 1.00

ATOM 131 O PRO A 24 -16.301 11.941 14.003 1.00 1.00

ATOM 132 CB PRO A 24 -15.472 10.777 16.853 1.00 1.00

ATOM 133 CG PRO A 24 -16.382 11.800 17.393 1.00 1.00

ATOM 134 CD PRO A 24 -15.767 13.150 17.163 1.00 1.00

ATOM 135 N VAL A 25 -14.857 10.231 13.671 1.00 1.00

ATOM 136 CA VAL A 25 -15.447 9.799 12.413 1.00 1.00

ATOM 137 C VAL A 25 -15.990 8.392 12.635 1.00 1.00

ATOM 138 O VAL A 25 -15.219 7.452 12.855 1.00 1.00

ATOM 139 CB VAL A 25 -14.418 9.826 11.249 1.00 1.00

ATOM 140 CG1 VAL A 25 -15.070 9.399 9.942 1.00 1.00

ATOM 141 CG2 VAL A 25 -13.794 11.217 11.101 1.00 1.00

ATOM 142 N TYR A 26 -17.319 8.265 12.603 1.00 1.00

ATOM 143 CA TYR A 26 -17.997 7.005 12.904 1.00 1.00

ATOM 144 C TYR A 26 -18.279 6.204 11.649 1.00 1.00

ATOM 145 O TYR A 26 -18.656 6.763 10.618 1.00 1.00

ATOM 146 CB TYR A 26 -19.331 7.247 13.623 1.00 1.00

ATOM 147 CG TYR A 26 -19.241 7.980 14.942 1.00 1.00

ATOM 148 CD1 TYR A 26 -19.472 9.352 15.007 1.00 1.00

ATOM 149 CD2 TYR A 26 -18.947 7.303 16.123 1.00 1.00

ATOM 150 CE1 TYR A 26 -19.403 10.037 16.214 1.00 1.00

ATOM 151 CE2 TYR A 26 -18.874 7.977 17.338 1.00 1.00

ATOM 152 CZ TYR A 26 -19.105 9.345 17.374 1.00 1.00

ATOM 153 OH TYR A 26 -19.042 10.027 18.566 1.00 1.00

ATOM 154 N PHE A 27 -18.104 4.891 11.753 1.00 1.00

ATOM 155 CA PHE A 27 -18.497 3.962 10.701 1.00 1.00

ATOM 156 C PHE A 27 -19.699 3.165 11.187 1.00 1.00

ATOM 157 O PHE A 27 -19.677 2.616 12.289 1.00 1.00

ATOM 158 CB PHE A 27 -17.349 2.992 10.385 1.00 1.00

ATOM 159 CG PHE A 27 -16.347 3.512 9.381 1.00 1.00

ATOM 160 CD1 PHE A 27 -16.293 4.860 9.033 1.00 1.00

ATOM 161 CD2 PHE A 27 -15.426 2.639 8.810 1.00 1.00

ATOM 162 CE1 PHE A 27 -15.352 5.324 8.114 1.00 1.00

ATOM 163 CE2 PHE A 27 -14.481 3.093 7.892 1.00 1.00

ATOM 164 CZ PHE A 27 -14.445 4.440 7.545 1.00 1.00

ATOM 165 N ARG A 28 -20.745 3.117 10.368 1.00 1.00

ATOM 166 CA ARG A 28 -21.869 2.205 10.597 1.00 1.00

ATOM 167 C ARG A 28 -22.107 1.369 9.345 1.00 1.00

ATOM 168 O ARG A 28 -21.578 1.679 8.277 1.00 1.00

ATOM 169 CB ARG A 28 -23.141 2.965 10.995 1.00 1.00

ATOM 170 CG ARG A 28 -23.101 3.584 12.395 1.00 1.00

ATOM 171 CD ARG A 28 -24.470 4.115 12.829 1.00 1.00

ATOM 172 NE ARG A 28 -24.997 5.157 11.939 1.00 1.00

ATOM 173 CZ ARG A 28 -24.716 6.456 12.029 1.00 1.00

ATOM 174 NH1 ARG A 28 -23.892 6.911 12.963 1.00 1.00

ATOM 175 NH2 ARG A 28 -25.259 7.309 11.170 1.00 1.00

ATOM 176 N GLY A 29 -22.896 0.306 9.493 1.00 1.00

ATOM 177 CA GLY A 29 -23.212 -0.596 8.397 1.00 1.00

ATOM 178 C GLY A 29 -22.004 -1.327 7.850 1.00 1.00

ATOM 179 O GLY A 29 -21.932 -1.610 6.656 1.00 1.00

ATOM 180 N VAL A 30 -21.049 -1.627 8.729 1.00 1.00

ATOM 181 CA VAL A 30 -19.859 -2.385 8.352 1.00 1.00

ATOM 182 C VAL A 30 -20.292 -3.712 7.698 1.00 1.00

ATOM 183 O VAL A 30 -21.150 -4.414 8.233 1.00 1.00
[truncated: 1,228,532 more chars]
